# Nirmatrelvir–ritonavir targeting viral persistence in post-COVID-19 condition (long COVID) in the USA (RECOVER-VITAL): a randomised, double-blind, placebo-controlled, phase 2 trial

**DOI:** 10.1016/S1473-3099(26)00406-8

**Published:** 2026-08-31

**Authors:** Lindsey R Baden, Nirav S Shah, Sean T H Liu, Jonathan Cohen, James Moy, Andre Kumar, Grace A McComsey, Peter Chen, Michelle Floris-Moore, Nora G Singer, Inti Fernandez, Alex J Slandzicki, Zanthia Wiley, Alexandra Kadl, David A Kaminsky, Harvey Hsu, Tiffany A Walker, Aluko A Hope, Luis Ostrosky-Zeichner, Jason D Goldman, Michael J Peluso, Thomas F Patterson, Sairam Parthasarathy, Janet M Mullington, Paul Bolin, Sarah E Jolley, Jerry A Krishnan, Mario Castro, Sally L Hodder, Priscilla Pemu, Helen Y Chu, Michael G Risbano, Maya R Jerath, Eleftherios Mylonakis, Ryan T Hurt, Radica Alicic, Nabila S Azad, Marc A Sala, Michelle S Harkins, Jeffrey Parsonnet, Neil Stafford, Philip Robinson, Sabiha Hussain, Xian Qiao, Sophie Two Hawk, Michael Lillestol, Nathan Erdmann, Kelly A Gebo, Praveen Sudhindra, Joseph Sassine, Gailen D Marshall, Tulika Chatterjee, Caryn G Morse, Eyal Kedar, William W Stringer, Jennifer A Frontera, Michael Jordan, Caitlin Blaskewicz, Jorge L Santana, Rachel A Foot, Cherry Wongtrakool, Matthew William McCarthy, Alem Mehari, Arch Amon, Alison K Cohen, Nita Jain, Christine Maughan, Doug Lindsay, Rachel Olson, Samuel Broderick, Pearl Rowe, Sean M O’Brien, David R Walt, Bruce D Levy, Leonard A Jason, Phillip A Low, Cyndya A Shibao, Barry Make, Lucinda Bateman, Susan Redline, David Knopman, Adrian F Hernandez, Tracy L Nolen, Craig Reist, Lisa Berdan, Richard Whitley, Kanecia O Zimmerman

**Affiliations:** **Department of Medicine, Brigham and Women’s Hospital, Harvard Medical School, Boston, MA, USA** (Prof L R Baden MD); **Department of Medicine, Endeavor Health, Evanston IL, USA** (N S Shah MD MPH); **Icahn School of Medicine at Mount Sinai, New York, NY, USA** (S T H Liu MD PhD); **Adventist Healthcare White Oak Medical Center, Calverton, MD, USA** (J Cohen MD); **Jadestone Clinical Research, Silver Spring, MD, USA** (J Cohen); **Department of Internal Medicine, Rush Medical College, Chicago, IL, USA** (J Moy MD); **Stanford University School of Medicine, Stanford, CA, USA** (Prof A Kumar MD Med); **Case Western Reserve University and University Hospitals of Cleveland, Cleveland, OH, USA** (Prof G A McComsey); **Department of Medicine, Women’s Guild Lung Institute, Cedars-Sinai Medical Center, Los Angeles, CA, USA** (Prof P Chen MD); **University of North Carolina School of Medicine, Chapel Hill, NC, USA** (M Floris-Moore MD MS); **The MetroHealth System, Case Western Reserve University, Cleveland, OH, USA** (N G Singer MD); **Valencia Medical & Research Center, Miami, FL, USA** (I Fernandez MD); **Clinical Trials Center of Middle Tennessee, Franklin, TN, USA** (A J Slandzicki MD); **Emory University School of Medicine, Atlanta, GA, USA** (Prof Z Wiley MD); **University of Virginia, Charlottesville, VA, USA** (A Kadl MD MS); **Pulmonary and Critical Care, University of Vermont Larner College of Medicine, Burlington, VT, USA** (Prof D A Kaminsky MD); **Banner–University Medical Center, Phoenix, AZ, USA** (Prof H Hsu MD); **University of Arizona College of Medicine, Phoenix, AZ, USA** (Prof H Hsu); **Division of General Internal Medicine, Department of Medicine, Emory University, Atlanta, GA, USA** (T A Walker MD); **Department of Medicine, Division of Pulmonary, Allergy and Critical Care Medicine, Oregon Health & Science University, Portland, OR, USA** (Prof A A Hope MD MSCE); **Division of Infectious Diseases, University of Texas Health Sciences Center at Houston, Houston, TX, USA** (Prof L Ostrosky-Zeichner MD); **Swedish Providence Medical Center, Seattle, WA, USA** (J D Goldman MD MPH); **Division of Allergy and Infectious Diseases, University of Washington, Seattle, WA, USA** (J D Goldman); **Division of HIV, Infectious Diseases, and Global Medicine, Zuckerberg San Francisco General Hospital, University of California, San Francisco, CA, USA** (M J Peluso MD); **University of Texas Health San Antonio, San Antonio, TX, USA** (Prof T F Patterson MD); **University of Arizona Center for Sleep Circadian and Neurosciences, Tucson, AZ, USA** (Prof S Parthasarathy MD); **Beth Israel Deaconess Medical Center, Boston, MA, USA** (Prof J M Mullington PhD); **East Carolina University, Greenville, NC, USA** (P Bolin Jr MD); **Division of Pulmonary, Allergy and Critical Care Medicine, University of Colorado, Aurora, CO, USA** (S E Jolley MD); **Illinois Research Network and University of Illinois Chicago, Chicago, IL, USA** (Prof J A Krishnan MD PhD); **Frontiers Clinical and Translational Science Institute, University of Kansas School of Medicine, Kansas City, KS, USA** (Prof M Castro MD MPH); **WVU Biomedical Research Center, Morgantown, WV, USA** (Prof S L Hodder MD); **Morehouse School of Medicine Department of Medicine and Clinical Research Center, Atlanta, GA, USA** (P Pemu MD); **University of Washington Schools of Medicine & Public Health, Seattle, WA, USA** (Prof H Y Chu MD MPH); **Division of Pulmonary, Allergy and Critical Care Medicine, University of Pittsburgh, Pittsburgh, PA, USA** (M G Risbano MD); **Department of Medicine, Division of Allergy and Immunology, Washington University in Saint Louis, Saint Louis, MO, USA** (Prof M R Jerath MD PhD); **Houston Methodist Hospital, Houston, TX, USA** (Prof E Mylonakis MD); **Weill Cornell Medical College, New York, NY, USA** (Prof E Mylonakis); **Mayo Clinic, Rochester, MN, USA** (R Y Hurt MD PhD); **Providence Medical Research Center, Providence Inland Northwest Health, Spokane, WA, USA** (Prof R Alicic MD); **Department of Medicine, University of Washington School of Medicine, Seattle, WA, USA** (Prof R Alicic); **Hospital Medicine Unit, Boston University Chobanian & Avedisian School of Medicine, Boston, MA, USA** (N S Azad MD); **Northwestern Feinberg School of Medicine, Chicago, IL, USA** (M A Sala MD); **Department of Internal Medicine, University of New Mexico, Albuquerque, NM, USA** (Prof M S Harkins MD); **Department of Medicine, Dartmouth-Hitchcock Medical Center, Lebanon, NH, USA** (Prof J Parsonnet MD); **Department of Medicine, Duke University School of Medicine, Durham, NC, USA** (N Stafford MD); **Hoag Memorial Hospital Presbyterian, Newport Beach, CA, USA** (P Robinson MD); **Robert Wood Johnson Medical School, Rutgers University, New Brunswick, NJ, USA** (Prof S Hussain MD MPH); **SMG Sentara Pulmonary, Critical Care & Sleep Specialists, Norfolk, VA, USA** (X Qiao MD); **Pulmonary and Critical Care Medicine, Eastern Virginia Medical School, Macon & Joan Brock Virginia Health Sciences at Old Dominion University, Norfolk, VA, USA** (X Qiao); **Avera Medical Group, Sioux Falls, SD, USA** (Prof S Two Hawk MD); **University of South Dakota Sanford School of Medicine, Sioux Falls, SD, USA** (Prof S Two Hawk); **Lillestol Research, Fargo, ND, USA** (M Lillestol MD FACP); **Department of Medicine, University of Alabama at Birmingham, Birmingham, AL, USA** (N Erdmann MD PhD); **Milken Institute School of Public Health, George Washington University, Washington, DC, USA** (Prof K A Gebo MD MPH); **Divisions of Infectious Diseases and Critical Care Medicine, Carle Health Methodist Hospital, Peoria, IL, USA** (P Sudhindra MD); **Division of Infectious Diseases, Department of Medicine, College of Medicine, The University of Oklahoma Health Campus, Oklahoma City, OK, USA** (J Sassine MD); **Division of Clinical Immunology, The University of Mississippi Medical Center, Jackson, MS, USA** (Prof G D Marshall MD PhD); **Department of Internal Medicine, University of Illinois College of Medicine at Peoria, IL, USA** (T Chatterjee MD MPH); **Wake Forest School of Medicine, Winston-Salem, NC, USA** (Prof C G Morse MD MPH); **Department of Clinical and Rural Health Research, St Lawrence Health, Potsdam, NY, USA** (E Kedar MD); **David Geffen School of Medicine at UCLA, Lundquist Institute for Biomedical Innovation at Harbor-UCLA Medical Center, Torrance, CA, USA** (Prof W W Stringer MD); **Division of Neurocritical Care and Stroke, NYU Grossman School of Medicine, New York, NY, USA** (Prof J A Frontera MD FNCS); **School of Medicine and Occupational Health Services Tufts University, Medford, MA, USA** (Prof M Jordan MD MPH); **Department of Infectious Disease, Cleveland Clinic Foundation, Cleveland OH, USA** (C Blaskewicz DO PhD); **Department of Internal Medicine, University of Puerto Rico, School of Medicine, San Juan, PR, USA** (Prof J L Santana MD FIDSA); **Division of Pulmonary, Critical Care and Sleep Medicine, University of Cincinnati, Cincinnati, OH, USA** (R A Foot MD); **Department of Medicine, Emory University School of Medicine, Atlanta, GA, USA** (Prof C Wongtrakool MD); **Atlanta VA Healthcare System, Decatur, GA, USA** (Prof C Wongtrakool); **Department of Medicine, Weill Cornell Medicine, New York, NY, USA** (M W McCarthy MD); **Department of Medicine, Howard University College of Medicine, Washington, DC, USA** (A Mehari MD FCCP); **Lakeland Regional Health, Lakeland, FL, USA** (A Amon MD); **Department of Epidemiology & Biostatistics, University of California San Francisco, San Francisco, CA, USA** (A K Cohen PhD MPH); **RECOVER Patient, Caregiver, or Community Representative, Durham, NC, USA** (A K Cohen, N Jain, C Maughan BS, D Lindsay); **Duke Clinical Research Institute, Duke University School of Medicine, Durham, NC, USA** (R Olson RN MS, S Broderick MS, P Rowe MPH, S M O’Brien PhD); **Department of Pathology, Brigham and Women’s Hospital, Boston, MA, USA** (Prof D R Walt PhD); **Harvard Medical School, Boston, MA, USA** (Prof D R Walt); **Wyss Institute for Biologically Inspired Engineering, Boston, MA, USA** (Prof D R Walt); **Mass General Brigham and Harvard Medical School, Boston, MA, USA** (Prof B D Levy MD); **Center for Community Research, DePaul University, Chicago, IL, USA** (Prof L A Jason PhD); **Department of Neurology, Mayo Clinic, Rochester, MN, USA** (P A Low MD); **Division of Genetics and Clinical Pharmacology, Department of Medicine, Vanderbilt University Medical Center, Nashville, TN, USA** (C A Shibao MD MSCI); **Department of Medicine, National Jewish Health, Denver, CO, USA** (Prof B Make MD); **Bateman Horne Center, Salt Lake City, UT, USA** (L Bateman MD MS); **Department of Medicine, Brigham and Women’s Hospital, Harvard Medical School, Boston, MA, USA** (Prof S Redline MD MPH); **Department of Neurology, Mayo Clinic, Rochester, MN, USA** (Prof D Knopman MD); **Duke Clinical Research Institute, Duke University School of Medicine, Durham, NC, USA** (Prof A F Hernandez MD MHS); **Social, Statistical, and Environmental Sciences, RTI International, Research Triangle Park, NC, USA** (T L Nolen DrPh, C Reist PhD); **National Institutes of Health, Bethesda, MD, USA** (L Berdan PA MHS); **Departments of Pediatrics, Microbiology, Medicine, and Neurosurgery, Heersink School of Medicine, The University of Alabama at Birmingham, Birmingham, AL, USA** (Prof R Whitley MD); **Duke Clinical Research Institute, Duke University School of Medicine, Durham, NC, USA** (Prof K O Zimmerman MD PhD)

## Abstract

**Background:**

Post-acute sequelae of SARS-CoV-2 infection, more commonly known as long COVID, has emerged as a major health problem. The pathogenesis of long COVID is unknown, but among the leading hypotheses is viral persistence. We aimed to investigate whether the use of the SARS-CoV-2 antiviral nirmatrelvir–ritonavir improved long COVID symptoms.

**Methods:**

We conducted a double-blind, placebo-controlled, randomised trial involving adults who had developed persistent symptoms (≥12 weeks) associated with three major symptom phenotypes (cognitive, autonomic, or exercise) after acute SARS-CoV-2 infection at 69 US sites. Participants were eligible if they were 18 years or older and had a previous suspected, probable, or confirmed SARS-CoV-2 infection, as defined by the Pan American Health Organization. Eligible participants were also required to have either at least two moderate symptoms from the same phenotype or one severe phenotype-associated symptom, as identified with the Cluster Targeted COVID-19 Symptom Questions. Participants were randomly allocated in a double-blind manner in a 1:1:1 ratio using permuted blocks of size 30 to receive either 15 days of active intervention followed by 10 days of placebo (300 mg nirmatrelvir–100 mg ritonavir twice daily, then 100 mg ritonavir–placebo); 25 days of active intervention (300 mg nirmatrelvir–100 mg ritonavir twice daily); or 25 days of placebo–ritonavir (100 mg ritonavir–placebo). A clinically significant change in patient-reported outcomes at day 90 comprised the primary endpoint: Patient-Reported Outcomes Measurement Information System Cognitive Function Short Form 8a, Orthostatic Hypotension Questionnaire question 1, and a modified version of the DePaul Symptom Questionnaire Post-Exertional Malaise short form. Secondary outcomes were phenotype-specific performance measures. The study was registered at ClinicalTrials.gov (NCT05595369) and is complete.

**Findings:**

Between July 27, 2023, and Sept 6, 2024, 1207 individuals were screened. Of these, 964 were randomly allocated and 959 participants, excluding four participants who were later found ineligible and one who did not initiate treatment, were enrolled in the three phenotypes: 332 to cognitive, 334 to autonomic, and 332 to exercise. In the 959 participants in the mITT population, 643 (67%) self-reported as female, 314 (33%) were male, and two participants had a sex of unknown or undifferentiated; 750 (78%) were White; and 108 (11%) were Hispanic, Latino, or Spanish. The median age was 49 years (IQR 38–59). No statistically significant benefits were observed for any phenotype for primary endpoints. For the cognitive phenotype, adjusted differences compared to placebo were 3·2% (95% CI –10·4 to 16·8, p=0·65) for the 25-day regimen and –2·2% (–15·5 to 11·1, p=0·74) for the 15-day regimen. For the autonomic phenotype, adjusted differences were –6·4% (–18·5 to 5·7, p=0·30) for the 25-day regimen compared to placebo and –0·1% (–12·5 to 12·3, p=0∙99) for the 15-day regimen compared to placebo. For exercise, adjusted differences were –7·8% (–19·5 to 3·8, p=0·19) for the 25-day regimen compared to placebo and 0·9% (–11·4 to 13·2, p=0∙88) for the 15-day regimen compared to placebo. There were no differences in secondary endpoints, and no safety signals were observed; there were no deaths, and 52 serious adverse events occurred in 42 (4%) of 963 participants over the course of the study.

**Interpretation:**

Nirmatrelvir–ritonavir for 15 days or 25 days showed no evidence of benefit in long COVID in any of the three phenotypes studied. These findings suggest additional approaches to measuring the symptom burden and treating Long COVID are needed.

**Funding:**

National Institutes of Health.

## Introduction

Post-acute sequelae of SARS-CoV-2 infection, more commonly known as long COVID, is a chronic condition that affects nearly every organ system, with more than 200 symptoms described, ranging from new-onset cognitive difficulties to dizziness and arrhythmias.^1–5^ Over 5% of US American adults currently experience long COVID, with over 1% experiencing significant activity limitations due to long COVID; spontaneous remission is rare.^6^ The pathogenesis of long COVID is unknown, but several possibilities being investigated include viral persistence, immune dysregulation, endothelial damage, and thrombotic diathesis.^7–11^

The US National Institutes of Health (NIH) RECOVER (Researching COVID to Enhance Recovery) initiative has facilitated an improved but still evolving understanding of long COVID. Research results, including data from the RECOVER initiative, have highlighted substantial heterogeneity in long COVID symptomology and presentation, with scant concordance between clinical findings and reported symptoms. Three predominant phenotypes are frequently reported and are of substantial importance to patients: cognitive dysfunction, autonomic dysfunction manifesting as orthostatic intolerance, and exercise intolerance with post-exertional malaise (PEM).^12–14^ Given the importance of these symptom complexes for a broad array of illnesses beyond long COVID, tools to characterise these symptoms have been developed and validated.

Viral persistence in the form of ongoing low-level or compartmentalised replication is a leading aetiological hypothesis for long COVID based on detection of SARS-CoV-2 years after primary infection, virus detection in some patients with long COVID symptoms,^15–17^ reports of long COVID prevention with antiviral therapy for acute COVID-19,^18–20^ and several anecdotal reports of antiviral therapy improving long COVID symptoms.^21,22^ Given these observations and the logic that persistence of an acute infection might contribute to chronic symptoms, it is crucial to rigourously assess viral persistence as an aetiology for some of the symptomatology in long COVID and to determine whether antiviral therapy can improve long COVID symptoms.

We conducted a randomised, double-blind, placebo-controlled study to investigate whether the use of the SARS-CoV-2 antiviral nirmatrelvir–ritonavir for 15 days or 25 days improved long COVID symptoms in three predominant clinical phenotypes. This phase 2 trial was designed—in conjunction with academic investigators, the NIH, patient representatives, and the US Food and Drug Administration—to also develop endpoints to assess long COVID and define elements crucial to characterising potential response to therapy, including standardised patient-reported outcome measures (PROMs), performance measures, kinetics and durability of improvement, and variability of long COVID symptomatology over time.

## Methods

### Study design and participants

The study recruited from July 27, 2023, to Sept 6, 2024, at 69 US sites across 27 states. Research sites were a mix of large academic centers, health systems, and small independent sites in rural, urban, and suburban outpatient settings, and all had or were associated with clinics that had significant experience in managing long COVID.

Participants were eligible if they were 18 years or older and had a previous suspected, probable, or confirmed SARS-CoV-2 infection, as defined by the Pan American Health Organization.^23^ Ascertainment of the index-COVID illness was determined in discussion with the participant. We documented what type of test or confirmation was done, and then the records to back that up were obtained (eg, a laboratory result). If no records were available, then the site principal investigator provided a narrative explaining the patient-reported situation and symptoms that led to a suspected diagnosis. Eligible participants were also required to have either at least two moderate symptoms from the same phenotype or one severe phenotype-associated symptom, as identified with the Cluster Targeted COVID-19 Symptom Questions (CTCSQ). All symptoms had to be new since the index COVID-19 illness and persistent for at least 12 weeks. After assignment to a phenotype based on the CTCSQ, participants were required to meet the phenotype-specific threshold on a PROM to qualify for enrolment (appendix p 10). Sex and ethnicity data were collected based on participant self-report.

Initially, participants could be enrolled in more than one phenotype; however, this approach proved overly burdensome for participants. In November 2023, the protocol was modified to assign each participant to the phenotype in which the most severe criteria were met. As phenotypes completed enrolment in 2024 (cognitive on June 26, exercise on Aug 9, and autonomic on Sept 6), participants were enrolled in remaining phenotypes as appropriate.

This study in the RECOVER Clinical Trial programme, VITAL (Viral Persistence and Reactivation, and Immune Dysregulation; NCT05595369), was investigator-initiated under US investigational new drug application and funded by the NIH. The protocol was approved by a central institutional review board (Advarra; approval Pro00067170; appendix pp 12–14), and all participants provided written informed consent. Safety oversight was under the direction of the NIH RECOVER Data Safety Monitoring Board. Details of study conduct can be found in the protocol and statistical analysis plan (appendix p 44).

### Randomisation and masking

Participants were randomised in a 1:1:1 ratio using permuted blocks of size 30 through the central Almac randomisation system to receive either 15 days of active intervention followed by 10 days of placebo (300 mg nirmatrelvir–100 mg ritonavir twice daily, then 100 mg ritonavir–placebo); 25 days of active intervention (300 mg nirmatrelvir–100 mg ritonavir twice daily); or 25 days of placebo–ritonavir (100 mg ritonavir–placebo twice daily). Low-dose ritonavir was administered to the placebo group to maintain masking, as it has a known and unique taste. Dose reduction for renal dysfunction was done as appropriate.

All three groups received the same number of tablets per dose, with doses packaged in two kits (15 days followed by 10 days). Participants, investigators, site personnel, data management personnel, and the blinded statistical analysis team were masked to treatment assignment. A separate unmasked biostatistical team prepared closed interim reports for the data and safety monitoring board. Only the central pharmacy for the study and the unmasked biostatistical team had access to unmasked treatment assignment.

### Procedures

Participants were followed up for 6 months, with the primary timepoint assessment conducted at an in-person clinical visit on day 90 (appendix p 11). Following completion of intervention, weekly remote follow-up was performed, and optional in-person visits for blood collection occurred on days 45, 60, and 120. The final study visit was a remote assessment on day 180.

Adverse events, serious adverse events, and events of special interest, including specific abnormalities in liver and thyroid function, were followed up through to day 180. Blood specimens to assess events of special interest were obtained at baseline, dosing midpoint (day 15), end of dosing (day 25), and day 90. Grading of adverse events was by the Common Terminology Criteria for Adverse Events version 5.0, Nov 27, 2017.

### Outcomes

The primary endpoint for each phenotype was the proportion of participants achieving a clinically meaningful improvement as assessed by phenotype-specific PROM at day 90 compared to baseline. Clinically significant improvement for each phenotype was supported by literature in non-long COVID populations^24–27^ and defined as follows: for cognitive, an increase by at least 5 T-score points on the Patient-Reported Outcomes Measurement Information System (PROMIS) Cognitive Function Short Form 8a; for autonomic, a decrease by at least one rating scale category on a modified version of the Orthostatic Hypotension Questionnaire (OHQ) question 1; and for exercise, absence of any moderate, severe, or very severe symptoms occurring 50% or more of the time as assessed by a modified version of the DePaul Symptom Questionnaire Post-Exertional Malaise (DSQ-PEM) short form. To account for within-person variability in cognitive and autonomic symptoms over time, an average of three weekly assessments closest to day 90 was used for these measures.

Secondary endpoints were based on the proportion of participants obtaining clinically meaningful improvement on phenotype-specific performance measures, which was assessed at day 90 compared to baseline and defined as follows: for cognitive, an increase by 0·5 SD in either the WHO–University of California Los Angeles Auditory Verbal Learning Test delayed recall measure or Symbol Digit Modalities Test number of correct substitution measure, and no decrease exceeding 0·15 SD in either of these measures; for autonomic, either a decrease in heart rate by at least 10 beats per minute or an increase in systolic blood pressure by at least 10 mm Hg, or an increase in diastolic blood pressure by at least 5 mm Hg, and no increase in heart rate or decrease in systolic or diastolic blood pressure compared to baseline on the active stand test at 10 min; and for exercise, an increase by at least 3 min in walking time on the Endurance Shuttle Walk Test.

### Statistical analysis

Sample size was chosen to provide 80% power to detect a difference of 25 percentage points in the proportion of participants who met the binary primary outcome for each pairwise comparison between a treatment group and control within each phenotype, assuming a 2-sided test with α=0·0166. A sample size of 82 per group yields 80% power assuming the true outcome probabilities are 37·5% in the control group and 62·5% in the active treatment group. This sample size was increased by 1/0·82=1·22 to account for a potential 18% dropout and loss to follow-up rate.

The primary analysis was based on a separate modified intention-to-treat (mITT) population defined for each phenotype. Each phenotype-specific mITT population consisted of all randomly allocated participants who received at least part of one dose of the study intervention and were eligible, enrolled, and randomised into that phenotype. Participants were analysed according to their randomly assigned intervention group. Participants who were enrolled into more than one phenotype, as allowed under Protocol Version 3.0, were included in the mITT population of each phenotype for which inclusion criteria were met. The overall mITT population consisted of participants included in at least one phenotype-specific mITT population.

The primary analysis was repeated in the population with high study drug adherence, consisting of all mITT participants who missed no more than three planned doses during their entire drug dosing period. Safety analyses were based on a population of all randomly allocated participants who received at least part of one dose of the study intervention or control, irrespective of eligibility for efficacy analysis. Treatment adherence was assessed by participant self-report and pill counts.

Treatment effects were estimated using logistic regression with direct standardisation (g-computation) and reported as the unadjusted and adjusted difference in the percentages improving between each intervention group and the control group. To enhance precision and account for chance imbalances across treatment groups, the primary analysis was adjusted for prespecified baseline covariates, including region (based on US census divisions), race, years of education, and the baseline value of the phenotype-specific PROM. The missing-data approach assumed that outcomes were missing at random, conditional on the model covariates. Participants with missing primary endpoint data did not contribute to model fitting but were retained when predicted outcomes were averaged over the phenotype-specific mITT population. To account for partial knowledge to inform endpoint definitions and timing, outcome measures were also assessed in a continuous manner and at several different time points: dosing midpoint, end of dosing, and day 180.

A Bonferroni–Holm procedure was used to control the family-wise type I error at a two-sided α of 0*·*05 across the three phenotype-specific primary outcomes. Within each phenotype, comparisons of the two active dose regimens with placebo–ritonavir were performed sequentially, with the 25-day regimen tested first, followed by the 15-day regimen if the preceding comparison was significant, according to the prespecified multiplicity strategy. No adjustments were made for other analyses, including secondary endpoints, continuous versions of the primary endpoints, subgroup analyses, or exploratory outcomes. Results are reported as point estimates and 95% CIs. CI widths were not adjusted for multiple comparisons and should not be used in place of hypothesis testing. Additional details are provided in the statistical analysis plan (appendix p 44).

### Role of the funding source

The funder, NIH, played a role in study design and management but had no role in analysis, interpretation, review, or final approval of the manuscript. Pfizer donated study medication and contributed to study design, but did not have a role in study conduct, interpretation of study data, or initial drafting of the manuscript.

## Results

Between July 27, 2023, and Sept 6, 2024, 1207 individuals were screened. Of these, 964 were randomly allocated and 959 participants, excluding four participants who were later found ineligible and one who did not initiate treatment, were enrolled in the three phenotypes: 332 to cognitive, 334 to autonomic, and 332 to exercise (figure 1). In the 959 participants in the mITT population, 643 (67%) self-reported as female, 314 (33%) were male, and two participants had a sex of unknown or undifferentiated; 750 (78%) were White; and 108 (11%) were Hispanic, Latino, or Spanish. The median age was 49 years (IQR 38–59). Similar demographic distributions were seen in the three phenotypes and across randomly allocated treatment groups (table 1). COVID infections were confirmed in 768 (80%) incidences, 96 (10%) were probable, and 45 (5%) were suspected cases of COVID. A total of 50 cases, largely occurring before May 2021, did not have adequate data to rigorously classify. The distribution of COVID diagnosis certainty was similar across treatment groups and the 3 phenotypes. Protocol deviations occurred in approximately 9% of participants and were similar across the three phenotypes, with missed collection of an endpoint being most common (appendix pp 15–18)

Phenotypes were similar in terms of other baseline characteristics including the mean duration between the diagnosis of the first long COVID-related SARS-CoV-2 infection to random allocation (range 28·2–32·0 months). Most participants reported having received a COVID-19 vaccine (range 74% to 83%). The incidence of comorbidities was similar across the three groups. The most frequently reported health conditions in participants across all three groups in the three symptom phenotypes were hypertension (range 19% to 36%), asthma (19% to 29%), major depression (21% to 34%), and anxiety disorder (28% to 49%). Median high-sensitivity C-reactive protein at baseline was between 1·3–2·3 mg/L across groups (table 1).

Treatment adherence was similar for all three treatment groups with 307 (95%) of 323, 293 (93%) of 316, and 306 (96%) of 320 of nirmatrelvir–ritonavir 25-day, nirmatrelvir–ritonavir 15-day, and placebo–ritonavir participants taking the full course, respectively. Study completion through to day 180 was over 90% in each phenotype (appendix p 19).

Unadjusted proportions of participants who met the predefined threshold for improvement in the primary endpoint were similar across treatment groups within each phenotype (table 2). For the cognitive phenotype, improvement occurred in 58% (59 of 102) of participants assigned to nirmatrelvir–ritonavir 25 days, 53% (59 of 111) of participants assigned to nirmatrelvir–ritonavir 15 days, and 55% (53 of 97) of participants assigned to placebo–ritonavir. Relative to placebo, adjusted differences were 3·2% (95% CI –10·4 to 16·8, p=0·65) for the 25-day regimen and –2·2% (–15·5 to 11·1, p=0·74) for the 15-day regimen. The median change from baseline in PROMIS Cognitive 8a T-score at day 90 was 5·9 (IQR 0·0–10·7) for nirmatrelvir–ritonavir 25 days, 6·3 (0·0–11·4) for nirmatrelvir–ritonavir 15 days, and 5·5 (0·0–13·2) for placebo–ritonavir (appendix p 24).

For the autonomic phenotype, improvement was observed in 62% (73 of 117), 70% (67 of 96), and 70% (73 of 104) of participants assigned to nirmatrelvir–ritonavir 25 days, nirmatrelvir–ritonavir 15 days, and placebo–ritonavir, respectively, with adjusted differences of –6·4% (95% CI –18·5 to 5·7, p=0·30) for the 25-day regimen compared to placebo and –0·1% (–12·5 to 12·3, p=0∙99) for the 15-day regimen compared to placebo. These findings indicate clinically meaningful improve ment in all three treatment groups, but no statistical difference between groups. The change in median OHQ question 1 score from baseline to day 90 was –2 (IQR –5 to 0) for nirmatrelvir–ritonavir 25 days, –2 (–4 to 0) for nirmatrelvir–ritonavir 15 days, and –3 (–5 to –1) for placebo–ritonavir (appendix p 24).

For the exercise phenotype, criteria for improvement were met by 25% (27 of 106) of participants assigned to nirmatrelvir–ritonavir 25 days, 34% (34 of 101) of participants assigned to nirmatrelvir–ritonavir 15 days, and 33% (36 of 110) of participants assigned to placebo–ritonavir. Again, comparisons with placebo did not indicate a treatment effect for the 25-day regimen (adjusted difference –7·8% [95% CI –19·5 to 3·8], p=0·19) or the 15-day regimen (0·9% [–11·4 to 13·2], p=0∙88). The median number of moderate, severe, or very severe symptoms with a frequency of 50% or greater as assessed by modified DSQ-PEM at day 90 compared to baseline was 0 (IQR –3 to 0) for nirmatrelvir–ritonavir 25 days, –1 (–3 to 0) for nirmatrelvir–ritonavir 15 days, and –1 (–3 to 0) for placebo–ritonavir (appendix p 24).

The distributions of primary outcomes were similar across treatment groups for each of the three phenotypes, at dosing midpoint (day 15), end of dosing (day 25), and day 180, and in the group with high medication adherence (appendix pp 20–38).

No evidence for an effect was seen in prespecified subgroup analyses by SARS-CoV-2 vaccination, time since first long COVID-related SARS-CoV-2 infection, race, ethnicity, sex, or initial COVID-19 treatment (figure 2). A total of 31 participants were included in multiple clusters. The distribution of treatment groups was 15 (48%) of multiple cluster participants randomly allocated to nirmatrelvir–ritonavir 25 day, ten (32%) allocated to nirmatrelvir–ritonavir 15 day, and six (19%) to placebo. Given the small numbers, no further analyses are possible.

Unadjusted proportions of participants who met the predefined threshold for improvement in the secondary endpoints were similar across treatment groups within each phenotype (table 2). For the cognitive phenotype, improvement occurred in nine (9%) of 97 participants assigned to nirmatrelvir–ritonavir 25 days, ten (9%) of 110 participants assigned to nirmatrelvir–ritonavir 15 days, and 13 (14%) of 94 participants assigned to placebo–ritonavir. Relative to placebo, adjusted differences were –4·5% (95% CI –13·4 to 4·3, p=0·32) for the 25-day regimen and –5·9% (–14·5 to 2·6, p=0∙18) for the 15-day regimen.

For the autonomic phenotype, improvement was observed in 12 (12%) of 101, 13 (14%) of 90, and 13 (14%) of 93 of participants assigned to nirmatrelvir–ritonavir 25 days, nirmatrelvir–ritonavir 15 days, and placebo–ritonavir, respectively. The adjusted differences of –3·6% (95% CI –12·3 to 5·0, p=0·41) for the 25-day regimen compared to placebo and –0·4% (–9·8 to 9·0, p=0∙93) for the 15-day regimen compared to placebo showed no treatment effect.

For the exercise phenotype, criteria for improvement were met by 16 (16%) of 98 participants assigned to nirmatrelvir–ritonavir 25 days, 22 (24%) of 90 participants assigned to nirmatrelvir–ritonavir 15 days, and 34 (33%) of 103 participants assigned to placebo–ritonavir. Comparisons with placebo did not indicate a treatment effect for the 25-day regimen (adjusted difference –13·8% [95% CI –24·9 to 2·8], p=0·014) or the 15-day regimen (adjusted difference –4·9% [–17·2 to 7·3], p=0∙43).

In all three phenotypes, distributions of secondary endpoints at additional timepoints (dosing midpoint, end of dosing, and day 180) were similar, suggesting consistency with the day 90 findings and no benefit of treatment (appendix pp 20–38).

No virological data on previous SARS-CoV2 co-infections or with other viruses have been generated to date.

Over the course of the study, the incidence of any treatment-emergent adverse event ranged from 64% to 66% of participants in nirmatrelvir–ritonavir treatment groups and 58% of participants in the placebo–ritonavir group (table 3, appendix pp 41–43). Study medication was stopped due to an adverse event in 11 (3%) of 323 on the nirmatrelvir–ritonavir 25 days schedule, 19 (6%) of 320 on nirmatrelvir–ritonavir 15 days, and 13 (4%) of 320 on placebo–ritonavir. This pattern was similar across phenotypes, with medication discontinuation in 2% of the cognitive, 3% of the autonomic, and 4% of the exercise phenotypes (table 3, appendix p 41).

There were no deaths, and 52 serious adverse events occurred in 42 (4%) of 963 participants over the course of the study and were similar across treatment groups (appendix pp 41–43). Three serious adverse events were considered by the investigator as related to treatment and recovered in follow-up: suspected drug-induced liver injury (nirmatrelvir–ritonavir 25 days, exercise), elevated liver function tests (nirmatrelvir–ritonavir 15 days, autonomic), and drug intolerance (placebo–ritonavir, exercise).

The most frequently reported adverse events in all three groups by participant were diarrhoea (56 [17%] of 323 in nirmatrelvir–ritonavir 25 days, 58 [18%] of 320 in nirmatrelvir–ritonavir 15 days, and 38 [12%] of 320 in placebo–ritonavir) and nausea (37 [11%] of 323, 44 [14%] of 320, and 23 [7%] of 320, respectively). No pattern of laboratory adverse events was identified.

## Discussion

The results of this randomised, double-blind, placebo-controlled trial showed no evidence that up to 25 days of the antiviral intervention nirmatrelvir–ritonavir provided clinical benefit by PROMs or performance measures for participants living with long COVID with cognitive dysfunction, autonomic dysfunction, or exercise intolerance as dominant symptoms. Benefit was not seen at any assessment timepoints or in either the 15-day or 25-day course of therapy. These findings are consistent with data from smaller studies assessing the role of 15 days of nirmatrelvir–ritonavir for treatment of long COVID in all patients reporting long COVID without careful clinical phenotype assessment and consideration.^28,29^

Previous trials of nirmatrelvir–ritonavir in long COVID and retrospective assessments of these trials have attributed negative findings to potential limitations in design elements (eg, small sample size, disease heterogeneity, and non-specific outcome measures or insensitive endpoints) as well as incongruity between the therapeutic agent and long COVID biology (eg, inaccessible viral reservoirs, non-replicating virus, inadequate duration, and alternative aetiology). RECOVER-VITAL sought to overcome these limitations with focus on sufficient sample sizes for each of three major phenotypes, use of previously validated assessment tools, phenotype-specific assessment with both PROMs and performance measures, assessment at multiple timepoints, binary and continuous endpoints to minimise the risk of missing an effect, and two dosing durations to investigate a potential duration–response relationship. Nevertheless, the optimal timing of endpoints, which will depend on the pathophysiology of the symptom complex and the mechanism of the treatment and healing, remains unknown and could have contributed to negative findings. Phenotypes such as PEM are relapsing and remitting, which could further complicate endpoint assessment, and repeated assess ment might have resulted in regression to the mean. Moreover, due to feasibility, RECOVER-VITAL did not restrict enrolment to those with evidence of persistent virus who are potentially most likely to benefit. The study also enrolled those with previous SARS-CoV-2 vaccination and prolonged long COVID duration, who might have a lower probability of improvement.

An important set of observations from this study is the behaviour of phenotype-specific outcome measures in the placebo–ritonavir group. Across all three phenotypes, substantial percentages of participants showed improvement over time by PROMs and less so by performance measures. Although the Hawthorne effect is commonly seen in clinical trials, perhaps due to attention and care during the course of a trial, the proportion improving in RECOVER-VITAL exceeded assumptions used for initial sample size calculations, perhaps limiting the possibility of detecting effect with the final sample size. Importantly, this background rate of improvement is not believed to be due to low-dose ritonavir administered to each group to maintain masking; previous studies suggested no meaningful biological activity of ritonavir against SARS-CoV-2, especially when used at a low dose.^30^ Nonetheless, the high proportion improving in the placebo–ritonavir group cautions against overinterpretation of uncontrolled observations and highlights the need for placebo control in studies of long COVID to better define therapeutic improvement rather than variation of symptomatology over time.

Nirmatrelvir–ritonavir has been used extensively to treat acute COVID, typically for 5 days. Extending the course to 25 days did not reveal any new safety concerns. A slight increase in transient mild nausea and diarrhoea was observed compared to ritonavir alone. Overall, treatment was well tolerated with few significant adverse events or discontinuations.

In addition to the design elements described above, this study has many strengths, including multicentre and nationally representative enrolment, high adherence to study medication, and high retention. This study also has limitations, including difficulties establishing the diagnosis of long COVID with homogenous specificity, no validated long COVID-specific symptom assessment tools, a study population that reflects most known long COVID populations (but might not reflect the true demographic distribution of long COVID and was largely White [78%], thereby limiting generalisability), potential for unmasking due to change in unpleasant taste for those receiving 15 days of active therapy (though the effect was probably scant as no difference was seen in the other two groups where this was not a consideration), lack of clarity around whether long COVID due to viral persistence would require a longer course of therapy, the fact that the proportion of individuals with viral persistence is unknown, and the fact that the duration of symptoms before enrolment is long, so those with severe symptoms might be beyond the point where antivirals can have an effect. In addition, as long COVID probably has more than one pathogenesis driving symptomatology, the question of whether nirmatrelvir–ritonavir leads to symptom improvement and associated viral clearance among those with evidence of viral persistence at baseline remains unanswered. Although RECOVER-VITAL obtained necessary blood samples to pursue these evaluations, both in terms of evidence for persistent virus at baseline and if viral antigen cleared with treatment, analyses are pending and will be published. Participants with persistent virus and with changes in viral antigen levels with treatment could provide crucial insights into long COVID pathogenesis and viral kinetics, and whether there are subgroups of patients for whom an antiviral could provide benefits.

In conclusion, antiviral treatment with nirmatrelvir–ritonavir in patients with long COVID for up to 25 days did not improve cognitive, autonomic, or exercise symptomatology as measured by symptom-specific PROMs and performance measures. Future directions to test this mechanistic hypothesis that long COVID symptoms relate to viral persistence include biomarker screening for measures of viral persistence at baseline, and possibly longer durations or combinations of antiviral or other therapies.

## Supplementary Material

1

## Figures and Tables

**Figure 1:**
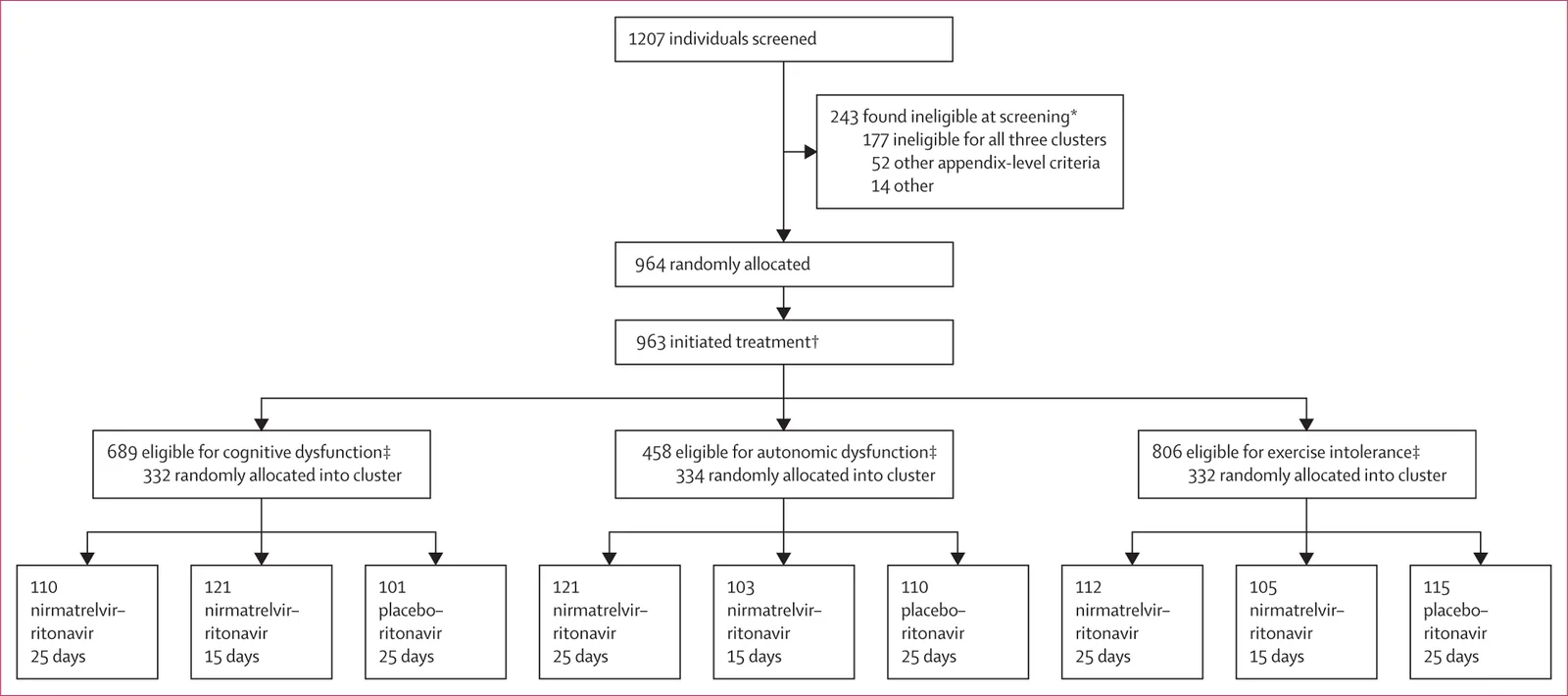
CONSORT diagram *Reasons are presented in a hierarchical manner (ie, ineligibility for at least one phenotype is reported among all those who screen failed before random allocation). “Other appendix-level criteria” comprises all those who were eligible for at least one phenotype. “Other” comprises those who were eligible for at least one phenotype and met appendix-level criteria. Common reasons in the “other” category include potential participants withdrawing their consent and being outside of the window between the screening and baseline visits. †There were four participants who were found to not meet eligibility criteria, but who were randomly allocated and initiated study treatment. These four participants are included in the count of participants who were randomly allocated and initiated study treatment. One participant was randomly allocated but left the clinic before initiating study treatment. ‡Before Protocol version 4.0, participants who were eligible for multiple symptom phenotypes were enrolled into all phenotypes for which eligibility criteria were met. Beginning with Protocol version 4.0, participants who met eligibility criteria for multiple phenotypes were enrolled in the phenotype for which they exhibited the most severe criteria.

**Figure 2:**
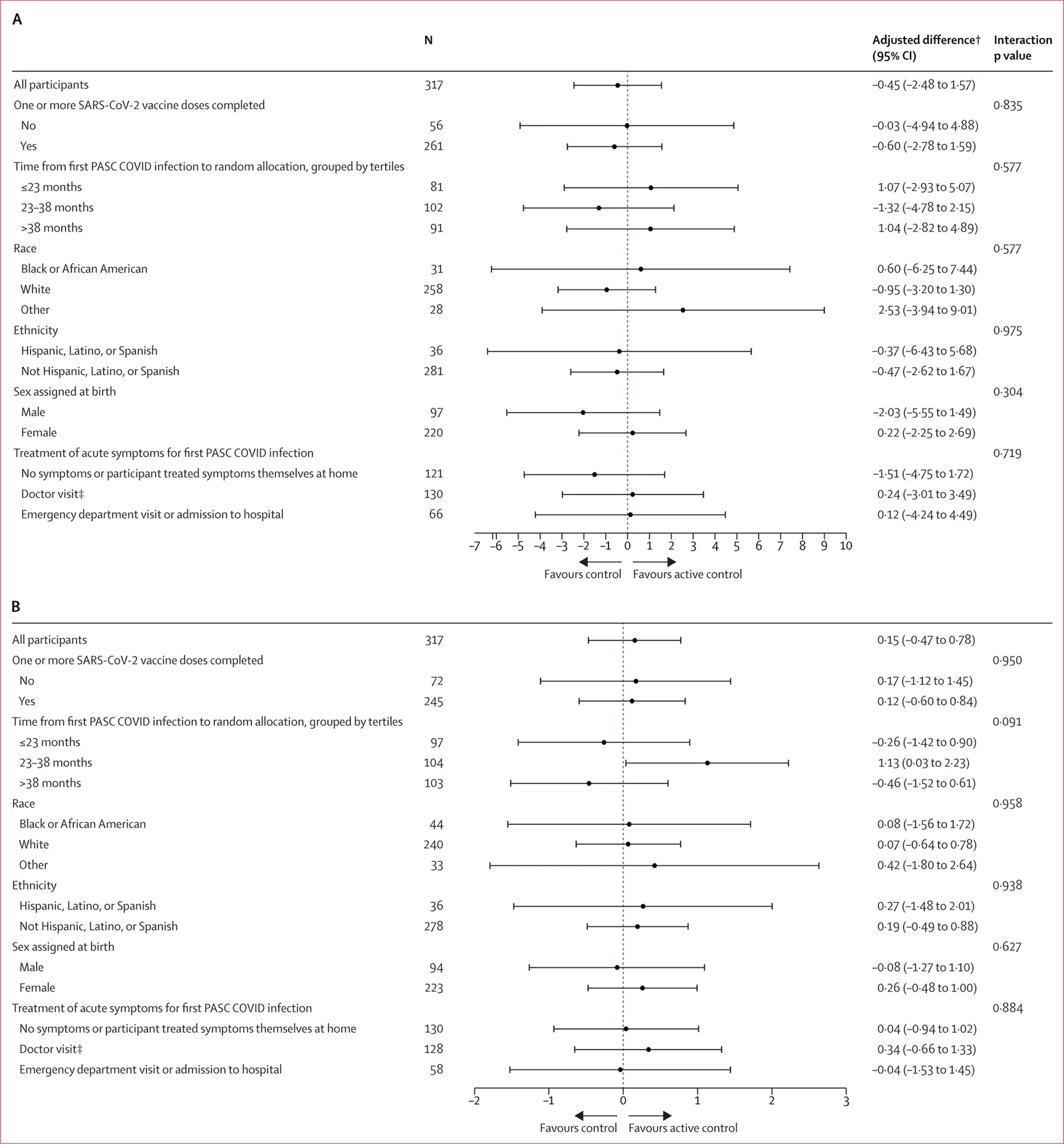

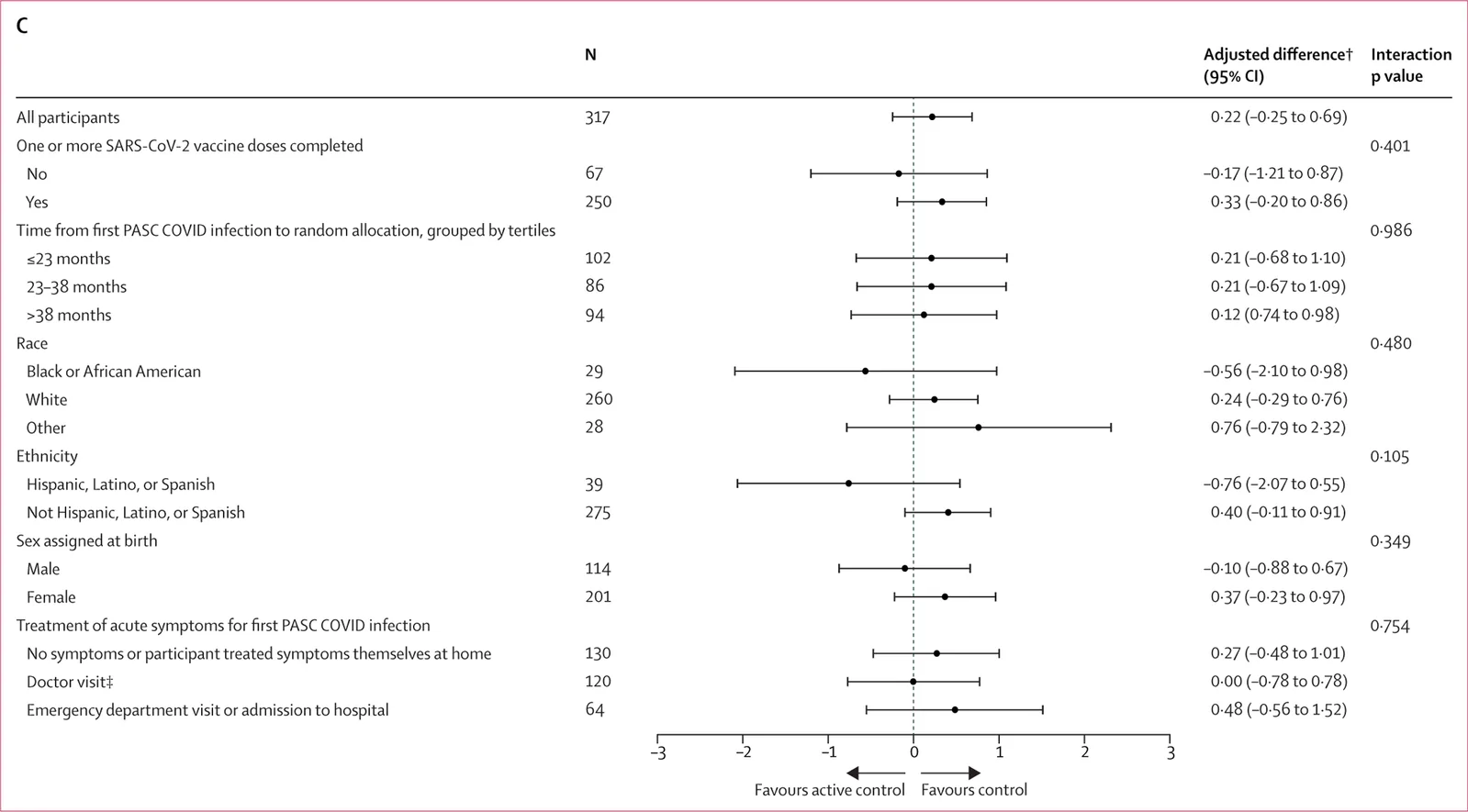
Forest plots of subgroups by the three phenotypes: (A) cognitive, (B) autonomic, and (C) exercise* DSQ-PEM=DePaul Symptom Questionnaire PEM. OHQ=Orthostatic Hypotension Questionnaire. PASC=post-acute sequelae of SARS-CoV-2 infection. PEM=post-exertional malaise. PROMIS=Patient-Reported Outcomes Measurement Information System. *Modified intention-to-treat population. This heterogeneity of treatment effect analysis assesses whether the difference in treatment means of the (A) PROMIS Cognitive 8a T-score, (B) OHQ question 1 score rating, or (C) DSQ-PEM Criteria for PEM at day 90 differs based on the level of various subgroups. The analysis was accomplished by fitting an analysis of covariance linear regression model with adjustment for treatment group (pooled active *vs* control), baseline measurement of the outcome variable, subgroup variable of interest, and the subgroup × treatment interaction (see statistical analysis plan section 8.6, appendix p 44). Participants with missing outcome or specific subgroup data were excluded from the analysis for their subgroup. †Presents the adjusted difference between a pooled active treatment group that pooled 15-day and 25-day dose durations and the control group. Negative values of this difference indicate that the adjusted mean for the pooled active treatment group is lower than the adjusted mean for the control group. ‡In-person clinic visit or telehealth appointment.

**Table 1:** Demographics by symptom phenotype in the modified intention-to-treat population

	Cognitive dysfunction n=332			Autonomic dysfunction n=334			Exercise intolerance n=332		
	Nirmatrelvir–ritonavir 25 days n=110	Nirmatrelvir–ritonavir 15 days n=121	Placebo–ritonavir 25 days n=101	Nirmatrelvir–ritonavir 25 days n=121	Nirmatrelvir–ritonavir 15 days n=103	Placebo–ritonavir 25 days n=110	Nirmatrelvir–ritonavir 25 days n=112	Nirmatrelvir–ritonavir 15 days n=105	Placebo–ritonavir 25 days n=115

**Age category, years**									
18–45	35 (32%)	47 (39%)	45 (45%)	47 (39%)	49 (48%)	58 (53%)	46 (41%)	44 (42%)	43 (37%)
>45–65	60 (55%)	56 (46%)	47 (47%)	62 (51%)	45 (44%)	44 (40%)	51 (46%)	47 (45%)	55 (48%)
>65	15 (14%)	18 (15%)	9 (9%)	12 (10%)	9 (9%)	8 (7%)	15 (13%)	14 (13%)	17 (15%)
**Ethnicity**									
Hispanic, Latino, or Spanish	10/110 (9%)	16/121 (13%)	11/101 (11%)	9/119 (8%)	10/102 (10%)	18/110 (16%)	15/112 (13%)	9/104 (9%)	16/113 (14%)
Not Hispanic, Latino, or Spanish	100/110 (91%)	105/121 (87%)	90/101 (89%)	110/119 (92%)	92/102 (90%)	92/110 (84%)	97/112 (87%)	95/104 (91%)	97/113 (86%)
**Race**									
White	93 (85%)	96 (79%)	82 (81%)	85 (70%)	82 (80%)	84 (76%)	87 (78%)	89 (85%)	90 (78%)
Black or African American	9 (8%)	14 (12%)	8 (8%)	18 (15%)	12 (12%)	17 (15%)	14 (12%)	7 (7%)	13 (11%)
Other	8 (7%)	11 (9%)	11 (11%)	18 (15%)	9 (9%)	9 (8%)	11 (10%)	9 (9%)	12 (10%)
**Sex assigned at birth**									
Male	23/110 (21%)	45/121 (37%)	35/101 (35%)	40/121 (33%)	30/103 (29%)	30/110 (27%)	42/111 (38%)	33/104 (32%)	45/115 (39%)
Female	87/110 (79%)	76/121 (63%)	66/101 (65%)	81/121 (67%)	73/103 (71%)	80/110 (73%)	69/111 (62%)	71/104 (68%)	70/115 (61%)
**Years of education**									
Participants, n	110/110	121/121	101/101	120/121	103/103	110/110	112/112	105/105	115/115
Mean (SD)	15·8 (2·40)	15·7 (2·39)	15·6 (2·61)	15·7 (2·31)	15·9 (2·44)	15·6 (2·56)	15·8 (2·40)	16·2 (2·48)	16·3 (2·45)
Median (IQR)	16 (14–18)	16 (14–18)	16 (14–18)	16 (14–18)	16 (14–18)	16 (14–18)	16 (14–18)	16 (14–18)	16 (14–18)
Time from first PASC COVID infection* to random allocation, months									
Participants, n	94/110	105/121	87/101	114/121	97/103	107/110	99/112	94/105	101/115
Mean (SD)	30·5 (11·61)	32·0 (11·85)	29·5 (11·49)	30·3 (13·30)	29·3 (14·23)	31·2 (13·65)	28·2 (13·58)	28·7 (14·31)	31·7 (11·95)
Median (IQR)	28 (22–40)	32 (23–41)	27 (22–41)	30 (20–42)	28 (19–42)	31 (22–43)	27 (19–39)	28 (18–40)	31 (23–40)
Received any vaccines for COVID-19	90 (82%)	99 (82%)	84 (83%)	95 (79%)	79 (77%)	81 (74%)	90 (80%)	81 (77%)	93 (81%)
**Baseline high-sensitivity C-reactive protein, mg/L**									
Participants, n	103/110	117/121	98/101	117/121	102/103	106/110	108/112	102/105	112/115
Mean (SD)	3·8 (3·96)	3·0 (4·36)	3·4 (4·30)	3·4 (4·33)	5·7 (18·6)	3·3 (5·18)	3·3 (4·13)	3·3 (4·42)	3·4 (5·32)
Median (IQR)	2·3 (0·8–5·2)	1·3 (0·6–3·4)	1·6 (0·7–4·9)	1·8 (0·7–4·4)	2·0 (0·6–4·0)	1·4 (0·5–4·1)	1·9 (0·8–3·8)	1·6 (0·6–3·9)	1·9 (0·8–4·4)
**Baseline PROMIS Cognitive 8a T-score**									
Participants, n	106/110	120/121	99/101	NA	NA	NA	NA	NA	NA
Mean (SD)	29·2 (5·15)	29·6 (4·98)	30·4 (5·03)	NA	NA	NA	NA	NA	NA
Median (IQR)	29·5 (22·4–33·1)	28·9 (25·7–33·9)	30·1 (27·5–33·9)	NA	NA	NA	NA	NA	NA
**Baseline OHQ item number 1 scale rating**									
Participants, n	NA	NA	NA	120/121	103/103	110/110	NA	NA	NA
Mean (SD)	NA	NA	NA	6·3 (1·98)	6·1 (1·90)	6·5 (1·67)	NA	NA	NA
Median (IQR)	NA	NA	NA	6 (5–8)	6 (4–7)	7 (5–8)	NA	NA	NA
**Baseline number of symptoms of moderate or greater severity with 50% or more frequency**									
Participants, n	NA	NA	NA	NA	NA	NA	112/112	105/105	115/115
Mean (SD)	NA	NA	NA	NA	NA	NA	4·0 (1·27)	3·7 (1·36)	3·8 (1·32)
Median (IQR)	NA	NA	NA	NA	NA	NA	5 (3–5)	4 (3–5)	4 (3–5)

Data are n (%) or n/N (%) unless otherwise stated. NA=not applicable. OHQ=Orthostatic Hypotension Questionnaire. PASC=post-acute sequelae of SARS-CoV-2 infection. PROMIS=Patient-Reported Outcomes Measurement Information System.

* First COVID infection that led to current PASC symptoms.

**Table 2:** Primary (PROM) and secondary performance measure endpoints by symptom phenotype in the modified intention-to-treat population

	Unadjusted percentage improving from baseline to day 90*			Adjusted difference (95% CI)^†^	
	Nirmatrelvir–ritonavir 25 days	Nirmatrelvir–ritonavir 15 days	Placebo–ritonavir 25 days	Nirmatrelvir–ritonavir 25 days minus placebo–ritonavir 25 days	Nirmatrelvir–ritonavir 15 days minus placebo–ritonavir 25 days

**Cognitive**					
Primary endpoint	59/102 (58%)	59/111 (53%)	53/97 (55%)	3·2% (−10·4 to 16·8), p=0·65	−2·2% (−15·5 to 11·1)
Secondary endpoint	9/97 (9%)	10/110 (9%)	13/94 (14%)	−4·5% (−13·4 to 4·3), p=0·32	−5·9% (−14·5 to 2·6)
**Autonomic**					
Primary endpoint	73/117 (62%)	67/96 (70%)	73/104 (70%)	−6·4% (−18·5 to 5·7), p=0·30	−0·1% (−12·5 to 12·3)
Secondary endpoint	12/101 (12%)	13/90 (14%)	13/93 (14%)	−3·6% (−12·3 to 5·0), p=0·41	−0·4% (−9·8 to 9·0)
**Exercise**					
Primary endpoint	27/106 (25%)	34/101 (34%)	36/110 (33%)	−7·8% (−19·5 to 3·8), p=0·19	0·9% (−11·4 to 13·2)
Secondary endpoint	16/98 (16%)	22/90 (24%)	34/103 (33%)	−13·8% (−24·9 to −2·8), p=0·01	−4·9% (−17·2 to 7·3)

Data are n/N (%) unless otherwise stated. PROM=patient-reported outcome measure. SAP=statistical analysis plan.

* Each phenotype-specific primary and secondary endpoint corresponds to a predefined level of improvement in a continuous outcome measure that corresponds to the symptom phenotype. Details on the corresponding definitions of improvement can be found in Section 2.2.1 of the SAP in the appendix (p 44). The denominator includes all participants with a non-missing endpoint.

† Estimation of treatment pairwise comparisons of adjusted differences in proportions were performed using the G-computation method that combines logistic regression with direct standardisation (see SAP Section 8.1 for details). After multiplicity adjustment, a p-value <0·01667 for the primary endpoint is considered significant (see SAP Section 5.5 for details). The covariates used in the analyses are as follows: baseline result of continuous outcome measure used to define improvement for the corresponding phenotype-specific primary endpoint, race (Black, White, or other as a categorical variable), years of education (continuous), and US region (northeast, midwest, south, and west, categorical).

**Table 3:** Overview of adverse events in the safety population

	Nirmatrelvir–ritonavir 25 days (n=323)	Nirmatrelvir–ritonavir 15 days(n=320)	Placebo–ritonavir 25 days (n=320)	Risk difference* (95% CI)

Death	0	0	0	
Nirmatrelvir–ritonavir 25 days minus placebo–ritonavir 25 days	··	··	··	NA
Nirmatrelvir–ritonavir 15 days minus placebo–ritonavir 25 days	··	··	··	NA
Any adverse event	212 (66%)	204 (64%)	185 (58%)	
Nirmatrelvir–ritonavir 25 days minus placebo–ritonavir 25 days	··	··	··	7·8% (0·3% to 15·3%)
Nirmatrelvir–ritonavir 15 days minus placebo–ritonavir 25 days				5·9% (−1·6% to 13·4%)
Any suspected unexpected adverse reaction	1 (<1%)	1 (<1%)	0	
Nirmatrelvir–ritonavir 25 days minus placebo–ritonavir 25 days	··	··	··	0·3% (−0·9% to 1·7%)
Nirmatrelvir–ritonavir 15 days minus placebo–ritonavir 25 days	··	··	··	0·3% (−0·9% to 1·8%)
Any event of special interest†	2 (1%)	3 (1%)	4 (1%)	
Nirmatrelvir–ritonavir 25 days minus placebo–ritonavir 25 days	··	··	··	−0·6% (−2·6% to 1·1%)
Nirmatrelvir–ritonavir 15 days minus placebo–ritonavir 25 days	··	··	··	−0·3% (−2·3% to 1·6%)
Any adverse event related to randomly allocated treatment	136 (42%)	132 (41%)	90 (28·1%)	
Nirmatrelvir–ritonavir 25 days minus placebo–ritonavir 25 days	··	··	··	14·0% (6·6% to 21·2%)
Nirmatrelvir–ritonavir 15 days minus placebo–ritonavir 25 days	··	··	··	13·1% (5·8% to 20·4%)
Any serious adverse event related to randomly allocated treatment	1 (<1%)	1 (<1%)	1 (<1%)	
Nirmatrelvir–ritonavir 25 days minus placebo–ritonavir 25 days	··	··	··	−0·0% (−1·5% to 1·4%)
Nirmatrelvir–ritonavir 15 days minus placebo–ritonavir 25 days	··	··	··	0·0% (−1·5% to 1·5%)
Any non-serious adverse event related to randomly allocated treatment	135 (42%)	132 (41%)	89 (28%)	
Nirmatrelvir–ritonavir 25 days minus placebo–ritonavir 25 days	··	··	··	14·0% (6·6% to 21·2%)
Nirmatrelvir–ritonavir 15 days minus placebo–ritonavir 25 days	··	··	··	13·4% (6·1% to 20·7%)
Any action taken with study drug									
Dose not changed	156 (48%)	151 (47%)	131 (41%)	
Nirmatrelvir–ritonavir 25 days minus placebo–ritonavir 25 days	··	··	··	7·4% (−0·3% to 15·0%)
Nirmatrelvir–ritonavir 15 days minus placebo–ritonavir 25 days	··	··	··	6·3% (−1·4% to 13·9%)
Dose reduced	1 (<1%)	3 (1%)	3 (1%)	
Nirmatrelvir–ritonavir 25 days minus placebo–ritonavir 25 days	··	··	··	−0·6% (−2·4% to 0·9%)
Nirmatrelvir–ritonavir 15 days minus placebo–ritonavir 25 days	··	··	··	0·0% (−1·9% to 1·9%)
Dose rate reduced	0	0	0	
Nirmatrelvir–ritonavir 25 days minus placebo–ritonavir 25 days	··	··	··	NA
Nirmatrelvir–ritonavir 15 days minus placebo–ritonavir 25 days	··	··	··	NA
Drug interrupted	8 (2%)	5 (2%)	5 (2%)	
Nirmatrelvir–ritonavir 25 days minus placebo–ritonavir 25 days	··	··	··	0·9% (−1·4% to 3·4%)
Nirmatrelvir–ritonavir 15 days minus placebo–ritonavir 25 days	··	··	··	0·0% (−2·2% to 2·2%)
Drug withdrawn	11 (3%)	19 (6%)	13 (4%)	
Nirmatrelvir–ritonavir 25 days minus placebo–ritonavir 25 days	··	··	··	−0·7% (−3·8% to 2·4%)
Nirmatrelvir–ritonavir 15 days minus placebo–ritonavir 25 days	··	··	··	1·9% (−1·6% to 5·5%)

NA=not applicable.

* 95% CIs for the risk difference were calculated with the use of the Miettinen–Nurminen method. Control is the reference group. Risk difference values smaller than 0 indicate a benefit with the active treatment.

† Events of special interest include alternation in thyroid function and drug–induced liver injury.

## Data Availability

De-identified summary data and participant-level data for the RECOVER-VITAL trial will be available in the National Heart, Lung, and Blood Institute’s BioData Catalyst ecosystem after study completion. The data will be available upon request by qualified researchers per BioData Catalyst requirements and approval. The trial protocol is available on the study website or at ClinicalTrials.gov.

## Research in context

### Evidence before this study

Persistent symptoms post-SARS-CoV-2 infection (long COVID) have emerged as a major cause of morbidity globally. Multiple possible aetiologies of long COVID are being investigated, with viral persistence emerging as a leading possibility based on detection of SARS-CoV-2 years after primary infection, virus detection in some patients with long COVID symptoms, reports of long COVID prevention with antiviral therapy for acute COVID-19, and several anecdotal reports of antiviral therapy improving long COVID symptoms. We searched MEDLINE for studies reported in English from Jan 1, 2020, to June 1, 2026, using the terms “Long COVID”, “PASC”, “antiviral therapy”, “nirmatrelvir–ritonavir”, “randomized controlled trial”, AND “prolonged COVID”. We found 36 trials fitting our criteria. Two randomised trials (STOP-PASC and PAX-LC) using 15 days of nirmatrelvir–ritonavir did not show a benefit of treatment, but are not considered definitive, due to the small number of patients treated (151 randomised to nirmatrelvir–ritonavir), entry criteria that were broad, and the primary outcome assessed—a Likert scale on improvement across six organ symptoms (developed for STOP-PASC) or overall Patient-Reported Outcomes Measurement Information System 29 (PROMIS-29) Physical Health Summary Score (for PAX-LC).

### Added value of this study

Between July 2023 and September 2024, 1207 individuals were screened for inclusion. Of these, 964 were randomised and 959 were enrolled with three major symptom phenotypes: 332 cognitive, 334 autonomic, and 332 exercise. Tools were used which focused on specific clinical phenotype dysfunction including both patient-reported outcome measures (PROMs) and objective performance measures. No significant improvement was observed by cluster, PROMs or performance measures, or by treatment duration. No safety concerns were identified. This trial is substantially larger than previous trials, and the added value of this trial over previous reports includes its treatment duration, double-blind design, reducing heterogeneity by looking at carefully defined phenotypes, and having phenotype-specific outcomes. These data further demonstrate the importance of a double-blind randomised controlled trial design given the significant undulation and improvement in long COVID symptoms in the research setting.

### Implications of all the available evidence

Taken together, treatment of long COVID with nirmatrelvir–ritonavir for up to 25 days did not provide any measurable clinical benefit. More research is needed to better define the pathogenesis of long COVID so treatments can be developed. It is important to assess repurposed medications in trials while pursuing an improved mechanistic understanding. These data add insights into how phenotype-specific measures behave over time in those with long COVID symptoms.
