# Supplementary material for: Nirmatrelvir–ritonavir targeting viral persistence in post-COVID-19 condition (long COVID) in the USA (RECOVER-VITAL): a randomised, double-blind, placebo-controlled, phase 2 trial
Source: Lancet Infect Dis. Author manuscript; Available in PMC 2026 Oct 16. (PMC13673146; doi:10.1016/S1473-3099(26)00406-8)
Supplement: Supplementary file 1 [file NIHMS2208855-supplement-1.pdf]

Supplementary Appendix

Contents

RECOVER-VITAL Collaborators/Study Group 2

Figure S1: Study Schema 10

Figure S2: Study Assessments 11

Table S1: Sites with ID Numbers 12

Table S2. Overview of Protocol Deviations – Overall Modified Intent-to-Treat Population 15

Table S3. Overview of Protocol Deviations by Symptom Cluster - Cognitive Dysfunction Modified Intent-to-Treat Population 16

Table S4. Overview of Protocol Deviations by Symptom Cluster - Autonomic Dysfunction Modified Intent-to-Treat Population 17

Table S5. Overview of Protocol Deviations by Symptom Cluster - Exercise Intolerance Modified Intent-to-Treat Population 18

Table S6: Study Drug Adherence, MITT 19

Table S7: Additional Analysis Results for Primary Endpoints by Symptom Cluster, MITT 20

Table S8: Analyses of Primary Endpoint in a Continuous Manner 22

Table S9: Summary Statistics at Multiple Time Points, MITT 24

Table S10: Main Analysis Results for Primary Endpoints by Symptom Cluster: High Study Drug Adherence Population 33

Table S11: Additional Analyses for Primary Endpoint Symptom Cluster in High Drug Adherence Population 36

Figure S3: Forest Plot of Heterogeneity of Treatment Effect (HTE) by Symptom Cluster, MITT Population 38

Table S12: Serious Adverse Events by System Organ and Preferred Term (MedDRA Version 26.1), Safety Population 41

Protocol and SAP 44

RECOVER-VITAL Collaborators/Study Group

Abdel-Hamid, Khaled Mohammed	Sub-Investigator	Washington University School of Medicine	St Louis, MO, USA
Aberg, Judith	Sub-Investigator	Icahn School of Medicine at Mount Sinai	New York, NY, USA
Adhikaram, Dushmanthi	Sub-Investigator	Saint Francis Medical Center	Peoria, IL, USA
Alicic, Radica	Principal Investigator	Providence Medical Research Center	Spokane, WA, USA
Alsoubani, Majd	Sub-Investigator	Tufts Medical Center	Boston, MA, USA
Amon, Arch	Principal Investigator	Lakeland Regional Medical Center	Lakeland, FL, USA
Anderson, Jordan	Sub-Investigator	Oregon Health and Science University	Portland, OR, USA
Antonelli, Maria	Sub-Investigator	MetroHealth System	Cleveland, OH, USA
Arjun, Arundhati	Sub-Investigator	Washington University School of Medicine	St Louis, MO, USA
Ayache, Mirna	Sub-Investigator	MetroHealth System	Cleveland, OH, USA
Aylward, Laura	Sub-Investigator	West Virginia Clinical and Translational Science Institute	Morgantown, WV, USA
Azad, Nabila	Principal Investigator	Boston Medical Center	Boston, MA, USA
Azola, Alba	Sub-Investigator	Johns Hopkins Hospital	Baltimore, MD, USA
Baden, Lindsey	Principal Investigator	Brigham and Womens Hospital	Boston, MA, USA
Bardes, James	Sub-Investigator	West Virginia Clinical and Translational Science Institute	Morgantown, WV, USA
Biller, Julie	Principal Investigator	Froedtert Hospital - Medical College of Wisconsin	Milwaukee, WI, USA
Blaskewicz, Caitlin	Principal Investigator	Cleveland Clinic	Cleveland, OH, USA
Bolin, Paul	Principal Investigator	East Carolina University	Greenville, NC, USA
Brinkley, Sherilyn	Sub-Investigator	Johns Hopkins Hospital	Baltimore, MD, USA
Broomes, Tarashon	Sub-Investigator	Icahn School of Medicine at Mount Sinai	New York, NY, USA
Brown, Jamie	Sub-Investigator	Lillestol Research LLC	Fargo, ND, USA
Casto, Amanda	Sub-Investigator	University of Washington	Seattle, WA, USA

Cattran, Ashley	Sub-Investigator	University of Cincinnati Medical Center	Cincinnati, OH, USA
Castro, Mario	Principal Investigator	University of Kansas Medical Center	Kansas City, KS, USA
Chand, Arista	Sub-Investigator	Banner University Medical Center Tucson	Tucson, AZ, USA
Chatterjee, Tulika	Principal Investigator	Saint Francis Medical Center	Peoria, IL, USA
Chen, Peter	Principal Investigator	Cedars-Sinai Medical Center	Los Angeles, CA, USA
Chu, Helen	Principal Investigator	University of Washington	Seattle, WA, USA
Cohen, Jonathan	Principal Investigator	Jadestone Clinical Research, LLC	Rockville, MD, USA
Cribbs, Sushma	Sub-Investigator	Atlanta V.A. Medical Center	Atlanta, GA, USA
Croghan, Ivana	Sub-Investigator	Mayo Clinic	Rochester, MN, USA
Culp, Matthew	Sub-Investigator	Methodist Medical Center of Illinois	Peoria, IL, USA
Decato, Thomas	Sub-Investigator	Los Angeles Biomedical Research Institute at Harbor-UCLA Medical Center	Torrance, CA, USA
Dedier, Julien	Sub-Investigator	Boston Medical Center	Boston, MA, USA
Detelich, Joshua	Sub-Investigator	Atlanta V.A. Medical Center	Decatur, GA, USA
Elsayed-Ali, Omar	Sub-Investigator	Washington University School of Medicine	St. Louis, MO, USA
Emerson, Karissa	Sub-Investigator	Lillestol Research LLC	Fargo, ND, USA
Engelson, Cecilia	Sub-Investigator	NYU Langone Health/Brooklyn Hospital	Brooklyn, NY, USA
Erdmann, Nathaniel	Principal Investigator	University of Alabama at Birmingham	Birmingham, AL, USA
Erlandson, Kristine	Sub-Investigator	University of Colorado	Aurora, CO, USA
Fernandez, Inti	Principal Investigator	Valencia Medical and Research Center	Miami, FL, USA
Floris-Moore, Michelle	Principal Investigator	University of North Carolina Hospital	Chapel Hill, NC, USA
Foot, Rachel	Principal Investigator	University of Cincinnati Medical Center	Cincinnati, OH, USA
Frontera, Jennifer	Principal Investigator	NYU Langone Health/Brooklyn Hospital	Brooklyn, NY, USA
Gaffney, Kimberly	Sub-Investigator	Brigham and Womens Hospital	Boston, MA, USA
Gebo, Kelly	Principal Investigator	Johns Hopkins Hospital	Baltimore, MD, USA
Ghauloush, Maher	Sub-Investigator	Tufts Medical Center	Boston, MA, USA

Gibson, Jamie	Sub-Investigator	St Lawrence Health System	Canton, NY, USA
Gilman, Lee	Sub-Investigator	Johns Hopkins Hospital	Baltimore, MD, USA
Gimbel, Alison	Sub-Investigator	UPMC Presbyterian Shadyside	Pittsburgh, PA, USA
Goepfert, Paul	Sub-Investigator	University of Alabama at Birmingham	Birmingham, AL, USA
Goldman, Jason	Principal Investigator	Swedish Health Services	Seattle, WA, USA
Gothing, Jon	Sub-Investigator	Brigham and Womens Hospital	Boston, MA, USA
Han, Jenny	Sub-Investigator	Grady Memorial Hospital	Atlanta, GA, USA
Haney, Elizabeth	Sub-Investigator	Oregon Health and Science University	Portland, OR, USA
Harkins, Michelle	Principal Investigator	University of New Mexico Health Science Center	Albuquerque, NM, USA
Hartwig, Kimberly	Sub-Investigator	Saint Francis Medical Center	Peoria, IL, USA
Hatlen, Timothy	Sub-Investigator	Los Angeles Biomedical Research Institute at Harbor-UCLA Medical Center	Torrance, CA, USA
Hodder, Sally	Principal Investigator	West Virginia Clinical and Translational Science Institute	Morgantown, WV, USA
Hope, Aluko	Principal Investigator	Oregon Health and Science University	Portland, OR, USA
Horrocks, James	Sub-Investigator	St Lawrence Health System	Potsdam, NY, USA
Hsu, Harvey	Principal Investigator	Banner University Medical Center Phoenix	Phoenix, AZ, USA
Humphreys, Sarah	Sub-Investigator	Icahn School of Medicine at Mount Sinai	New York, NY, USA
Hurt, Ryan	Principal Investigator	Mayo Clinic	Rochester, MN, USA
Hussain, Sabiha	Principal Investigator	Rutgers University - Robert Wood Johnson Medical School	New Brunswick, NJ, USA
Ibrahim, Homam	Sub-Investigator	Jadestone Clinical Research, LLC	Silver Spring, MD, USA
Igho-Pemu, Priscilla	Principal Investigator	Morehouse School of Medicine	Atlanta, GA, USA
Isache, Carmen	Principal Investigator	University of Florida College of Medicine Jacksonville	Jacksonville, FL, USA
Jerath, Maya	Principal Investigator	Washington University School of Medicine	St. Louis, MO, USA
Jolley, Sarah	Principal Investigator	University of Colorado	Aurora, CO, USA

Jordan, Michael	Principal Investigator	Tufts Medical Center	Boston, MA, USA
Juskowich, Joy	Sub-Investigator	West Virginia Clinical and Translational Science Institute	Morgantown, WV, USA
Kadl, Alexandra	Principal Investigator	University of Virginia	Charlottesville, VA, USA
Kaminsky, David	Principal Investigator	Vermont Lung Center, University of Vermont	Colchester, VT, USA
Katcher, Jeremy	Sub-Investigator	Washington University School of Medicine	St Louis, MO, USA
Kau, Andrew	Sub-Investigator	Washington University School of Medicine	St. Louis, MO, USA
Kedar, Eyal	Principal Investigator	St Lawrence Health System	Canton, NY, USA
Kim, Christina	Sub-Investigator	Swedish Health Services	Seattle, WA, USA
Knodle, Ryan	Sub-Investigator	University of Illinois at Chicago	Chicago, IL, USA
Koch, Stanley	Principal Investigator	Koch Family Medicine	Morton, IL, USA
Krishnan, Jerry	Principal Investigator	University of Illinois at Chicago	Chicago, IL, USA
Kumar, Andre	Principal Investigator	Stanford University	Stanford, CA, USA
Layton, Alicia	Sub-Investigator	Methodist Medical Center of Illinois	Peoria, IL, USA
Lillestol, Michael	Principal Investigator	Lillestol Research LLC	Fargo, ND, USA
Lillestol, Michael	Sub-Investigator	Lillestol Research LLC	Fargo, ND, USA
Lin, Matthew	Sub-Investigator	University of Texas Health Science Center at Houston	Houston, TX, USA
Liu, Sean	Principal Investigator	Icahn School of Medicine at Mount Sinai	New York, NY, USA
Lozinski, Grace	Sub-Investigator	Hoag Memorial Hospital Presbyterian	Newport Beach, CA, USA
Lyndaker, McKailey	Sub-Investigator	St Lawrence Health System	Canton, NY, USA
Lyons, Maureen	Sub-Investigator	Oregon Health and Science University	Portland, OR, USA
Maini, Nayna	Sub-Investigator	Washington University School of Medicine	St Louis, MO, USA
Malakooti, Shahdi	Sub-Investigator	MetroHealth System	Cleveland, OH, USA
Mantia, Tarisa	Sub-Investigator	Washington University School of Medicine	St Louis, MO, USA
Marathe, Jai	Sub-Investigator	Boston Medical Center	Boston, MA, USA
Marconi, Vincent	Sub-Investigator	Atlanta V.A. Medical Center	Decatur, GA, USA

Marshall, Gailen	Principal Investigator	University of Mississippi Medical Center	Jackson, MS, USA
McCarthy, Matthew	Principal Investigator	Weill Cornell Medicine	New York, NY, USA
McComsey, Grace	Principal Investigator	University Hospitals Cleveland Medical Center	Cleveland, OH, USA
Mehari, Ahem	Principal Investigator	Howard University Hospital	Washington, D.C. USA
Mehta, Sarah	Sub-Investigator	Washington University School of Medicine	St. Louis, MO, USA
Menson, Katherine	Sub-Investigator	Vermont Lung Center, University of Vermont	Burlington, VT, USA
Middleton, Sondra	Sub-Investigator	Icahn School of Medicine at Mount Sinai	New York, NY, USA
Miller, Casi	Sub-Investigator	Clinical Trials Center of Middle Tennessee	Franklin, TN, USA
Monroy, Jennifer	Sub-Investigator	Washington University School of Medicine	St. Louis, MO, USA
Moon, Juhi	Sub-Investigator	Johns Hopkins Hospital	Baltimore, MD, USA
Morse, Caryn	Principal Investigator	Wake Forest University Health Sciences	Winston Salem, NC, USA
Moukabary, Talal	Sub-Investigator	Banner University Medical Center Tucson	Tucson, AZ, USA
Moy, James	Principal Investigator	Rush University Medical Center	Chicago, IL, USA
Mullington, Janet	Principal Investigator	Beth Israel Deaconess Med. Ctr.	Boston, MA, USA
Mylonakis, Eleftherios	Principal Investigator	Houston Methodist Hospital	Houston, TX, USA
Nanchal, Rahul	Sub-Investigator	Froedtert Hospital - Medical College of Wisconsin	Milwaukee, WI, USA
Neuman, Robert	Principal Investigator	Kaiser Permanente Southwood	Atlanta, GA, USA
Nordvig, Anna	Sub-Investigator	Weill Cornell Medicine	New York, NY, USA
Ostrosky-Zeichner, Luis	Principal Investigator	University of Texas Health Science Center at Houston	Houston, TX, USA
Owusu-Asare, Charity	Sub-Investigator	Washington University School of Medicine	St Louis, MO, USA
Parker, Ann	Sub-Investigator	Johns Hopkins Hospital	Baltimore, MD, USA

Parsonnet, Jeffrey	Principal Investigator	Dartmouth Hitchcock Medical Center	Lebanon, NH, USA
Parthasarathy, Sairam	Principal Investigator	Banner University Medical Center Tucson	Tucson, AZ, USA
Patterson, Thomas	Principal Investigator	University of Texas Health Science Center at San Antonio	San Antonio, TX, USA
Patel, Bela	Sub-Investigator	University of Texas Health Science Center at Houston	Houston, TX, USA
Peluso, Michael	Principal Investigator	University of California San Francisco General Hospital	San Francisco, CA, USA
Qiao, Xian	Principal Investigator	Sentara Norfolk General Hospital	Norfolk, VA, USA
Reece, Rebecca	Sub-Investigator	West Virginia Clinical and Translational Science Institute	Morgantown, WV, USA
Risbano, Michael	Principal Investigator	UPMC Presbyterian Shadyside	Pittsburgh, PA, USA
Rischard, Franz	Sub-Investigator	Banner University Medical Center Tucson	Tucson, AZ, USA
Robinson, Philip	Principal Investigator	Hoag Memorial Hospital Presbyterian	Newport Beach, CA, USA
Sala, Marc	Principal Investigator	Northwestern Memorial Hospital	Chicago, IL, USA
Salomone, John	Sub-Investigator	Washington University School of Medicine	St. Louis, MO, USA
Santana, Jorge	Principal Investigator	University of Puerto Rico	San Juan, Puerto Rico
Sanosyan, Armen	Sub-Investigator	Washington University School of Medicine	St. Louis, MO, USA
Sarafian, Farjad	Sub-Investigator	Hoag Memorial Hospital Presbyterian	Newport Beach, CA, USA
Sarwari, Arif	Sub-Investigator	West Virginia Clinical and Translational Science Institute	Morgantown, WV, USA
Sassine, Joseph	Principal Investigator	Oklahoma Clinical and Translational Science Institute	Oklahoma City, OK, USA
Sciurba, Frank	Sub-Investigator	UPMC Presbyterian Shadyside	Pittsburgh, PA, USA
Sender, Abraham	Sub-Investigator	Vermont Lung Center, University of Vermont	Burlington, VT, USA
Shah, Mehr Zahra	Sub-Investigator	Washington University School of Medicine	St Louis, MO, USA
Shah, Nirav	Principal Investigator	North Shore University HealthSystem/Evanston Hospital	Evanston, IL, USA

Sheehan, Elyce	Sub-Investigator	University of New Mexico Health Science Center	Albuquerque, NM, USA
Sherman, Amy	Sub-Investigator	Brigham and Womens Hospital	Boston, MA, USA
Shouman, Kamal	Sub-Investigator	Mayo Clinic	Rochester, MN, USA
Simmons, Christopher	Principal Investigator	University of Kentucky Chandler Medical Center	Lexington, KY, USA
Singer, Wolfgang	Sub-Investigator	Mayo Clinic	Rochester, MN, USA
Singer, Nora	Principal Investigator	MetroHealth System	Cleveland, OH, USA
Skinner, Kristina	Sub-Investigator	Washington University School of Medicine	St. Louis, MO, USA
Slandzicki, Alex	Principal Investigator	Clinical Trials Center of Middle Tennessee	Franklin, TN, USA
Small, Catherine	Sub-Investigator	Weill Cornell Medicine	New York, NY, USA
Smullin, Tanya	Sub-Investigator	Washington University School of Medicine	St Louis, MO, USA
Stafford, Neil	Principal Investigator	Duke Clinical & Translational Science Institute	Durham, NC, USA
Stout, Janice	Sub-Investigator	Sentara Norfolk General Hospital	Norfolk, VA, USA
Stringer, William	Principal Investigator	Los Angeles Biomedical Research Institute at Harbor-UCLA Medical Center	Torrance, CA, USA
Sudhindra, Praveen	Principal Investigator	Methodist Medical Center of Illinois	Peoria, IL, USA
Sukhera, Fatima	Sub-Investigator	Oklahoma Clinical and Translational Science Institute	Oklahoma, OK, USA
Two Hawk, Sophie	Principal Investigator	Avera McKennan Hospital & University Health Center	Sioux Falls, SD, USA
Vanwagoner, Tim	Sub-Investigator	Oklahoma Clinical and Translational Science Institute	Oklahoma City, OK, USA
Verplaetse, Wyatt	Sub-Investigator	Washington University School of Medicine	St. Louis, MO, USA
Walker, Tiffany	Principal Investigator	Grady Memorial Hospital	Atlanta, GA, USA
Walsh, Stephen	Sub-Investigator	Brigham and Womens Hospital	Boston, MA, USA
Wedner, H. James	Sub-Investigator	Washington University School of Medicine	St. Louis, MO, USA
Weinberger, Elisheva	Sub-Investigator	MetroHealth System	Cleveland, OH, USA

Whiteheart, Sidney	Sub-Investigator	University of Kentucky Chandler Medical Center	Lexington, KY, USA
Wiley, Zanthia	Principal Investigator	Emory Hope Clinic	Decatur, GA, USA
Wilkin, Aimee	Sub-Investigator	Wake Forest University Health Sciences	Winston Salem, NC, USA
Wongtrakool, Cherry	Principal Investigator	Atlanta V.A. Medical Center	Decatur, GA, USA
Yadav, Siddhant	Sub-Investigator	Mayo Clinic	Rochester, MN, USA
Yadav, Manajyoti	Sub-Investigator	Saint Francis Medical Center	Peoria, IL, USA
Yee, Nathan	Sub-Investigator	Los Angeles Biomedical Research Institute at Harbor-UCLA Medical Center	Torrance, CA, USA
Yendewa, George	Sub-Investigator	University Hospitals Cleveland Medical Center	Cleveland, OH, USA

Figure S1: Study Schema

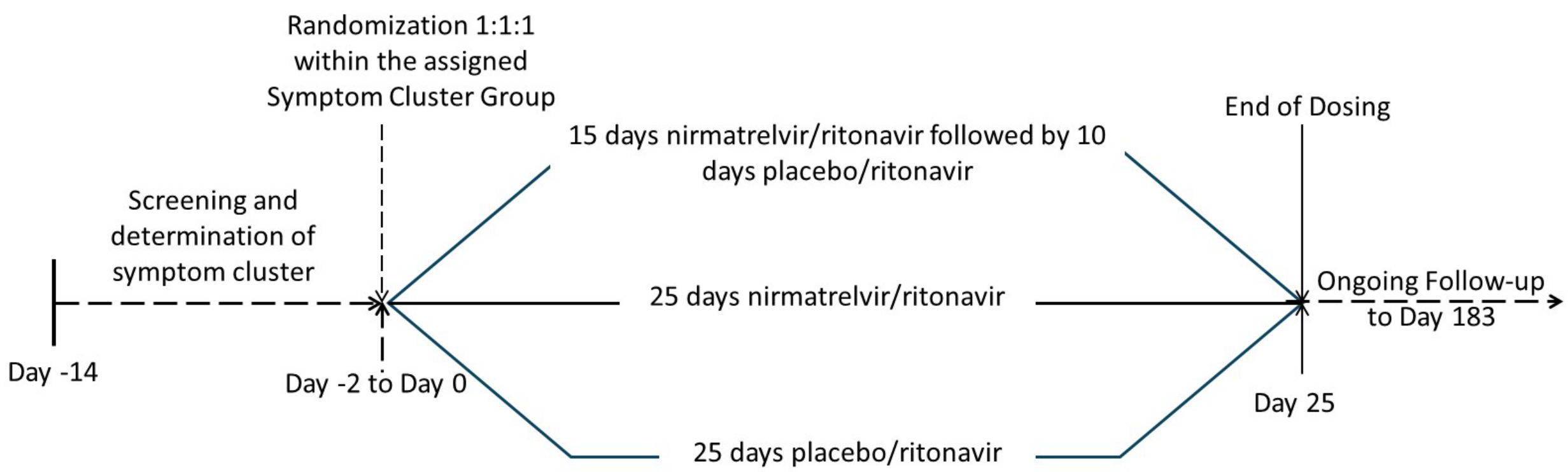

Figure S2: Study Assessments

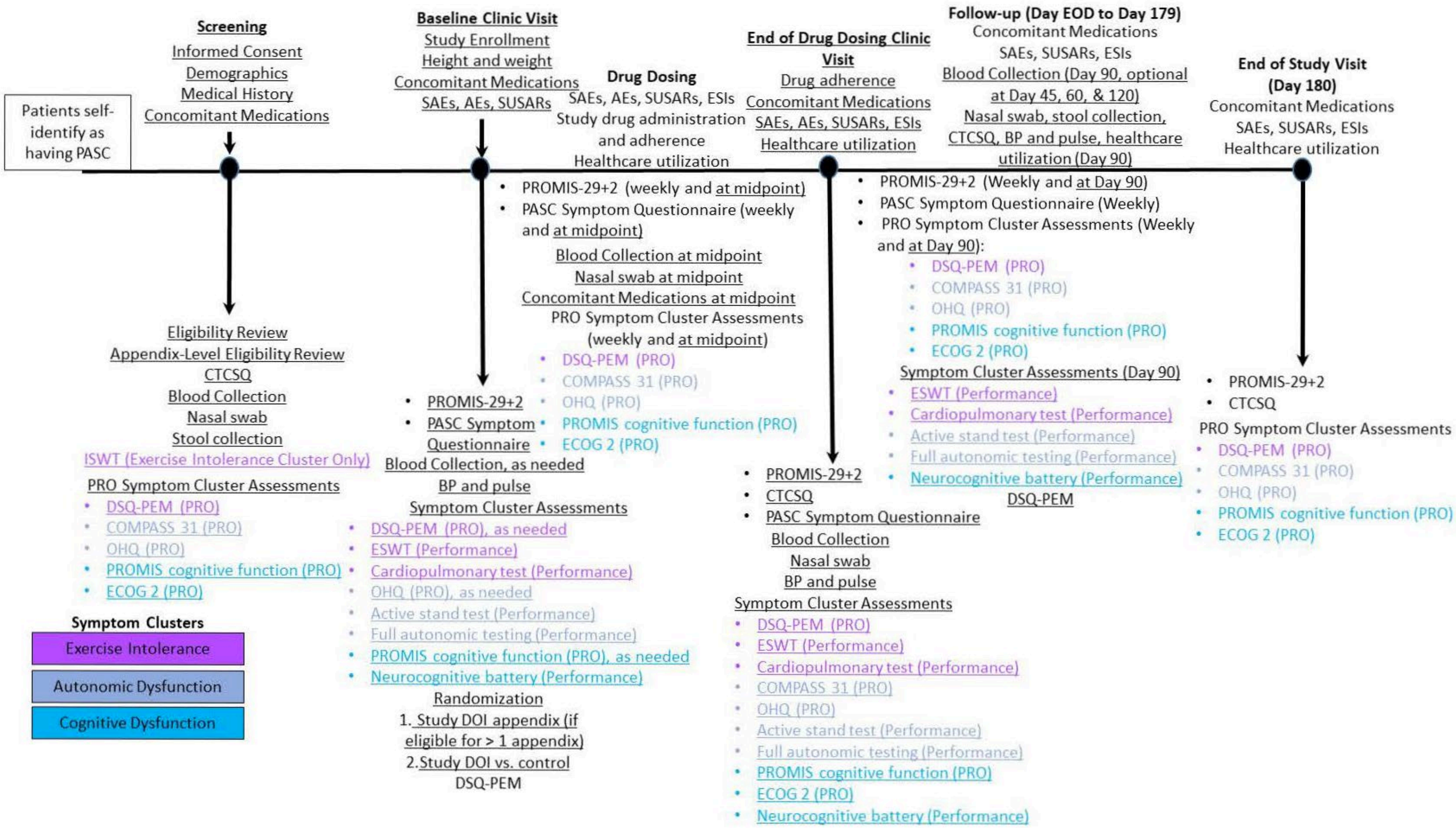

Table S1: Sites with ID Numbers

DCRI Site ID	CIRBI Site ID	Site PI Name	Site Name
VI034	SSU00231108	Radica Alicic, MD	Providence Sacred Heart Medical Center and Children's Hospital-PSHMC
VI136	SSU00223724	Arch Amon, MD	Lakeland Regional Health Hollis Cancer Center
VI005	SSU00224233	Nabila Azad, MD	Boston Medical Center
VI006	SSU00222002	Lindsey Baden, MD	Brigham and Women's Hospital
VI243	SSU00252981	Julie Biller, MD	Medical College of Wisconsin
VI254	SSU00225526	Caitlin Blaskewicz, DO	Cleveland Clinic
VI107	SSU00223440	Paul Bolin, MD	East Carolina University
VI052	SSU00229141	Mario Castro, MD, MPH	University of Kansas Medical Center
VI290	SSU00222471	Tulika Chatterjee, MD, MPH	Saint Francis Medical Center
VI010	SSU00223146	Peter Chen, MD	Cedars-Sinai Medical Center
VI061	SSU00223997	Helen Chu, MD, MPH	University of Washington
VI131	SSU00223145	Jonathan B. Cohen, MD, FACP	Jadestone Clinical Research
VI047	SSU00229251	Nathan Erdmann, MD, PhD	University of Alabama at Birmingham (UAB)
VI312	SSU00241335	Inti Fernandez, MD	Valencia Medical & Research Center
VI226	SSU00224212	Michelle Floris, MD	University of North Carolina at Chapel Hill
V1220	SSU00249076	Rachel Foot, MD	UC Health/University of Cincinnati
SD243	SSU00257925	Rose Franco, MD	Medical College of Wisconsin
VI163	SSU00251798	Jennifer Frontera, MD	NYU Langone/NYU School of Medicine
VI135	SSU00226638	Idania Garcia Del Sol, MD	L&A Morales Healthcare, Inc.
V1065	SSU00223676	Kelly Gebo, MD, MPH	Johns Hopkins
VI040	SSU00226296	Jason Goldman, MD, MPH	Swedish Medical Center
VI057	SSU00227333	Michelle Harkins, MD, FCCP, FACP	University of New Mexico
VI062	SSU00222148	Sally Hodder, MD	West Virginia University
VI169	SSU00222822	Aluko Hope, MD, MSCE	Oregon Health & Science University
VI003	SSU00228205	Harvey Hsu, MD	University of Arizona

VI143	SSU00224302	Ryan Hurt, MD, PhD	Mayo Clinic Health System
VI287	SSU00226178	Sabiha Hussain, M.D.	Rutgers
VI222	SSU00229385	Carmen Isache, MD	University of Florida
VI069	SSU00227275	Maya Jerath, MD, PhD	Washington University
VI050	SSU00223674	Sarah Jolley, MD, MSc	University of Colorado Denver
VI042	SSU00233351	Michael Jordan, MD	Tufts Medical Center & Tufts University Health Sciences
VI232	SSU00223825	Alexandra Kadl, MD, MS	University of Virginia
VI283	SSU00223936	David Kaminsky, M.D.	University of Vermont
VI087	SSU00223769	Eyal Kedar, M.D.	Canton Potsdam Hospital
VI310	SSU00224871	Stanley Koch, MD	Koch Family Medicine
VI060	SSU00225680	John Kriesel, MD	University of Utah
VI045	SSU00222691	Jerry Krishnan, M.D., PhD.	University of Illinois at Chicago
VI038	SSU00224392	Andre Kumar, MD, MEd	Stanford University
VI297	SSU00223841	Michael J. Lillestol, M.D.	Lillestol Research LLC
VI020	SSU00233285	Sean Liu, MD PhD	Mount Sinai (NY) / Icahn School of Medicine at Mount Sinai
V1055	SSU00223202	Gailen Marshall, PhD, MD	University of Mississippi Medical Center
VI248	SSU00227371	Matthew McCarthy, MD, FACP	Weill Cornell Medical College & New York Presbyterian Hospital
VI009	SSU00223523	Grace McComsey, M.D.	University Hospitals Cleveland Medical Center
VI019	SSU00226240	Alem Mehari, MD, FCCP	Howard University
VI068	SSU00227453	Caryn Morse, MD, MPH	Wake Forest University Health Sciences
VI182	SSU00227233	James Moy, M.D.	Rush University Medical Center
VI004	SSU00227152	Janet Mullington, PhD	Beth Israel Deaconess Medical Center
VI278	SSU00228279	Eleftherios Mylonakis, MD, PhD, FIDSA	Houston Methodist Research Institute
VI023	SSU00255640	Robert Neuman, MD	Kaiser Permanente
VI230	SSU00223927	Luis Z. Ostrosky, MD, FACP, FIDSA, FSHEA, FECMM, CMQ	University of Texas Health Science Center Houston (UTHSCH)
VI277	SSU00244587	Jeffrey Parsonnet, MD	Dartmouth-Hitchcock Health
VI002	SSU00228199	Sairam Parthasarathy, MD	University of Arizona

VI059	SSU00222550	Thomas Patterson, M.D., FACP, FIDSA	University of Texas Health Science Center at San Antonio (UTHSCSA)
VI044	SSU00232468	Michael Peluso, MD MPhil MHS DTM&H	University of California San Francisco (UCSF)
VI030	SSU00231903	Priscilla Pemu, MD	Morehouse School of Medicine
VI292	SSU00224849	Xian Qiao, MD	Sentara Healthcare
VI067	SSU00227384	Michael Risbano, MD	University of Pittsburgh Medical Center (UPMC)
VI125	SSU00228183	Philip A. Robinson, M.D., FIDSA	Hoag Memorial Hospital
VI274	SSU00226199	Marc Sala, MD	Northwestern University
VI018	SSU00223564	Jorge L. Santana Bagur, M.D.	University of Puerto Rico
VI058	SSU00228948	Joseph Sassine, MD, FACP	University of Oklahoma Health Sciences Center
VI164	SSU00224517	Nirav Shah, MD, MPH	Endeavor Health (prev Northshore University HealthSystem & Edward-Elmhurst Health)
VI053	SSU00246261	Christopher Simmons, MD, PhD	University of Kentucky
VI041	SSU00223271	Nora Singer, MD	MetroHealth
VI063	SSU00225491	Alex Slandzicki, MD	Clinical Trials Center of Middle Tennessee
VI235	SSU00226972	Neil Stafford, MD	Duke University Health System
VI252	SSU00223360	William Stringer, MD	Lundquist Institute for Biomedical Innovation at Harbor-UCLA Medical Center
VI289	SSU00225300	Praveen Sudhindra, MD	Methodist Medical Center of Illinois
VI285	SSU00222944	Sophie Two Hawk, MD	Avera Research
VI016	SSU00224325	Tiffany Walker, MD	Emory University
VI014	SSU00224256	Zanthia E. Wiley, MD	Emory University
VI0001	SSU00234132	Cherry Wongtrakool, MD	Department of Veterans Affairs (Atlanta)

All sites were overseen by a central IRB (Advarra), approval number Pro00067170.

Table S2. Overview of Protocol Deviations – Overall Modified Intent-to-Treat Population

	PAXLOVID 25 Day N = 323		PAXLOVID 15 Day N = 316		Control N = 320		Overall N = 959	
	Participants n (%)	Deviations n	Participants n (%)	Deviations n	Participants n (%)	Deviations n	Participants n (%)	Deviations n
Any major protocol deviations	30 (9.3%)	33	26 (8.2%)	29	34 (10.6%)	39	90 (9.4%)	101
Medication errors resulting in SAE or exposure events	1 (0.3%)	1	2 (0.6%)	2	1 (0.3%)	1	4 (0.4%)	4
Failure to obtain informed consent or obtaining consent after initiation of study procedures	2 (0.6%)	2	0	0	2 (0.6%)	2	4 (0.4%)	4
Enrollment of an ineligible participant	4 (1.2%)	4	0	0	2 (0.6%)	2	6 (0.6%)	6
Performing a study procedure not approved by the IRB	0	0	2 (0.6%)	2	0	0	2 (0.2%)	2
Dispensing error (wrong study product)	1 (0.3%)	1	0	0	1 (0.3%)	1	2 (0.2%)	2
Enrollment of a participant or participants after IRB-approval has expired	0	0	0	0	0	0	0	0
Use of outdated/expired consent form	2 (0.6%)	2	0	0	0	0	2 (0.2%)	2
Inappropriate documentation of informed consent	0	0	1 (0.3%)	2	0	0	1 (0.1%)	2
Missed collection of the primary outcome measure(s)	8 (2.5%)	8	12 (3.8%)	12	13 (4.1%)	13	33 (3.4%)	33
Key study procedures performed by the study staff without appropriate qualifications or train	0	0	0	0	0	0	0	0
Other	13 (4.0%)	15	11 (3.5%)	11	17 (5.3%)	20	41 (4.3%)	46

Note: An additional 11 participants were found to have an incorrect version of the informed consent form.

Table S3. Overview of Protocol Deviations by Symptom Cluster - Cognitive Dysfunction Modified Intent-to-Treat Population

	PAXLOVID 25 Day N = 110		PAXLOVID 15 Day N = 121		Control N = 101		Overall N = 332	
	Participants n (%)	Deviations n	Participants n (%)	Deviations n	Participants n (%)	Deviations n	Participants n (%)	Deviations n
Any major protocol deviations	13 (11.8%)	16	15 (12.4%)	17	11 (10.9%)	13	39 (11.7%)	46
Medication errors resulting in SAE or exposure events	0	0	2 (1.7%)	2	0	0	2 (0.6%)	2
Failure to obtain informed consent or obtaining consent after initiation of study procedures	1 (0.9%)	1	0	0	2 (2.0%)	2	3 (0.9%)	3
Enrollment of an ineligible participant	2 (1.8%)	2	0	0	2 (2.0%)	2	4 (1.2%)	4
Performing a study procedure not approved by the IRB	0	0	0	0	0	0	0	0
Dispensing error (wrong study product)	0	0	0	0	0	0	0	0
Enrollment of a participant or participants after IRB-approval has expired	0	0	0	0	0	0	0	0
Use of outdated/expired consent form	1 (0.9%)	1	0	0	0	0	1 (0.3%)	1
Inappropriate documentation of informed consent	0	0	1 (0.8%)	2	0	0	1 (0.3%)	2
Missed collection of the primary outcome measure(s)	4 (3.6%)	4	7 (5.8%)	7	3 (3.0%)	3	14 (4.2%)	14
Key study procedures performed by the study staff without appropriate qualifications or train	0	0	0	0	0	0	0	0
Other	6 (5.5%)	8	6 (5.0%)	6	5 (5.0%)	6	17 (5.1%)	20

Table S4. Overview of Protocol Deviations by Symptom Cluster - Autonomic Dysfunction Modified Intent-to-Treat Population

	PAXLOVID 25 Day N = 121		PAXLOVID 15 Day N = 103		Control N = 110		Overall N = 334	
	Participants n (%)	Deviations n	Participants n (%)	Deviations n	Participants n (%)	Deviations n	Participants n (%)	Deviations n
Any major protocol deviations	8 (6.6%)	8	4 (3.9%)	4	9 (8.2%)	11	21 (6.3%)	23
Medication errors resulting in SAE or exposure events	0	0	0	0	1 (0.9%)	1	1 (0.3%)	1
Failure to obtain informed consent or obtaining consent after initiation of study procedures	0	0	0	0	0	0	0	0
Enrollment of an ineligible participant	0	0	0	0	0	0	0	0
Performing a study procedure not approved by the IRB	0	0	0	0	0	0	0	0
Dispensing error (wrong study product)	1 (0.8%)	1	0	0	0	0	1 (0.3%)	1
Enrollment of a participant or participants after IRB-approval has expired	0	0	0	0	0	0	0	0
Use of outdated/expired consent form	1 (0.8%)	1	0	0	0	0	1 (0.3%)	1
Inappropriate documentation of informed consent	0	0	0	0	0	0	0	0
Missed collection of the primary outcome measure(s)	3 (2.5%)	3	2 (1.9%)	2	4 (3.6%)	4	9 (2.7%)	9
Key study procedures performed by the study staff without appropriate qualifications or train	0	0	0	0	0	0	0	0
Other	3 (2.5%)	3	2 (1.9%)	2	5 (4.5%)	6	10 (3.0%)	11

Table S5. Overview of Protocol Deviations by Symptom Cluster - Exercise Intolerance Modified Intent-to-Treat Population

	PAXLOVID 25 Day N = 112		PAXLOVID 15 Day N = 105		Control N = 115		Overall N = 332	
	Participants n (%)	Deviations n	Participants n (%)	Deviations n	Participants n (%)	Deviations n	Participants n (%)	Deviations n
Any major protocol deviations	12 (10.7%)	12	8 (7.6%)	9	16 (13.9%)	17	36 (10.8%)	38
Medication errors resulting in SAE or exposure events	1 (0.9%)	1	0	0	0	0	1 (0.3%)	1
Failure to obtain informed consent or obtaining consent after initiation of study procedures	1 (0.9%)	1	0	0	0	0	1 (0.3%)	1
Enrollment of an ineligible participant	4 (3.6%)	4	0	0	1 (0.9%)	1	5 (1.5%)	5
Performing a study procedure not approved by the IRB	0	0	2 (1.9%)	2	0	0	2 (0.6%)	2
Dispensing error (wrong study product)	0	0	0	0	1 (0.9%)	1	1 (0.3%)	1
Enrollment of a participant or participants after IRB-approval has expired	0	0	0	0	0	0	0	0
Use of outdated/expired consent form	1 (0.9%)	1	0	0	0	0	1 (0.3%)	1
Inappropriate documentation of informed consent	0	0	0	0	0	0	0	0
Missed collection of the primary outcome measure(s)	1 (0.9%)	1	3 (2.9%)	3	6 (5.2%)	6	10 (3.0%)	10
Key study procedures performed by the study staff without appropriate qualifications or train	0	0	0	0	0	0	0	0
Other	4 (3.6%)	4	4 (3.8%)	4	8 (7.0%)	9	16 (4.8%)	17

Table S6: Study Drug Adherence, MITT

	PAXLOVID 25 Day N = 323	PAXLOVID 15 Day N = 316	Control N = 320	Overall N = 959
Study drug administered	323 / 323 (100%)	316 / 316 (100%)	320 / 320 (100%)	959 / 959 (100%)
Half dose due to renal impairment	13 / 323 (4.0%)	9 / 316 (2.8%)	9 / 320 (2.8%)	31 / 959 (3.2%)
Completed full course of treatment*	307 / 323 (95.0%)	293 / 316 (92.7%)	306 / 320 (95.6%)	906 / 959 (94.5%)
Overdose**	0 / 323	0 / 316	0 / 320	0 / 959
Study drug interruption	23 / 323 (7.1%)	33 / 316 (10.4%)	31 / 320 (9.7%)	87 / 959 (9.1%)
Did not complete full course of treatment	16 / 323 (5.0%)	23 / 316 (7.3%)	14 / 320 (4.4%)	53 / 959 (5.5%)
Participant ended study early	0 / 16	2 / 23 (8.7%)	0 / 14	2 / 53 (3.8%)
Non-compliance with study intervention	1 / 16 (6.3%)	1 / 23 (4.3%)	0 / 14	2 / 53 (3.8%)
AE/SAE	12 / 16 (75.0%)	14 / 23 (60.9%)	5 / 14 (35.7%)	31 / 53 (58.5%)
Clinical deterioration	0 / 16	0 / 23	0 / 14	0 / 53
Investigator decision	1 / 16 (6.3%)	1 / 23 (4.3%)	2 / 14 (14.3%)	4 / 53 (7.5%)
Participant concerns	2 / 16 (12.5%)	5 / 23 (21.7%)	3 / 14 (21.4%)	10 / 53 (18.9%)
Confirmed new case of acute SARs-CoV-2	0 / 16	0 / 23	3 / 14 (21.4%)	3 / 53 (5.7%)
Other	0 / 16	0 / 23	1 / 14 (7.1%)	1 / 53 (1.9%)
Unknown/Not reported	0 / 16	0 / 23	0 / 14	0 / 53
Number of completed doses***				
N	323	316	320	959
Mean (SD)	48.7 (4.20)	48.2 (5.01)	48.8 (3.60)	48.6 (4.31)
Median (Q1, Q3)	50 (49, 50)	50 (49, 50)	50 (49, 50)	50 (49, 50)
Min, Max	20, 50	12, 50	25, 50	12, 50
Number of missed doses				
N	323	316	320	959
Mean (SD)	1.3 (4.20)	1.8 (5.01)	1.2 (3.60)	1.4 (4.31)
Median (Q1, Q3)	0 (0, 1)	0 (0, 1)	0 (0, 1)	0 (0, 1)
Min, Max	0, 30	0, 38	0, 25	0, 38

*Participants who did not discontinue treatment are counted as having completed the full course of treatment.

**An overdose is defined as any dose of nirmatrelvir greater than 900 mg (6 nirmatrelvir tablets) or ritonavir greater than 300 mg (3 ritonavir tablets) within a 24-hour time period.

***Each randomized participant was scheduled to take two doses of study drug per day for 25 days. The number of completed doses is calculated as 50 planned doses minus the number of planned missed doses as reported in the 'Study Drug Completion' section of the 'Study Drug Adherence' eCRF.

Table S7: Additional Analysis Results for Primary Endpoints by Symptom Cluster, MITT

Panel A: Cognitive Dysfunction Symptom Cluster

	PAXLOVID 25 Day	PAXLOVID 15 Day	Control
**Cognitive Dysfunction Symptom Cluster - PROMIS Cognitive 8a***	N = 110	N = 121	N = 101
Adjusted T-Score Mean at Day 180 (95% CI) (Exploratory Endpoint)**	37.01 (35.30, 38.71)	36.33 (34.58, 38.08)	37.26 (35.32, 39.20)
vs Control (95% CI), p-value	-0.26 (-2.68, 2.17), 0.835	-0.94 (-3.40, 1.53), 0.456	-
vs PAXLOVID 15 Day (95% CI), p-value	0.68 (-1.64, 3.00), 0.567	-	0.94 (-1.53, 3.40), 0.456
vs PAXLOVID 25 Day (95% CI), p-value	-	-0.68 (-3.00, 1.64), 0.567	0.26 (-2.17, 2.68), 0.835

Panel B: Autonomic Dysfunction Symptom Cluster

	PAXLOVID 25 Day	PAXLOVID 15 Day	Control
**Autonomic Dysfunction Symptom Cluster - Orthostatic Hypotension Questionnaire***	N = 121	N = 103	N = 110
Adjusted Overall OHQ Composite Score Mean at Day 90 (95% CI) (Exploratory Endpoint)**	4.36 (3.87, 4.85)	4.33 (3.86, 4.80)	4.20 (3.72, 4.68)
vs Control (95% CI), p-value	0.16 (-0.50, 0.82), 0.633	0.13 (-0.51, 0.76), 0.692	-
vs PAXLOVID 15 Day (95% CI), p-value	0.03 (-0.62, 0.69), 0.922	-	-0.13 (-0.76, 0.51), 0.692
vs PAXLOVID 25 Day (95% CI), p-value	-	-0.03 (-0.69, 0.62), 0.922	-0.16 (-0.82, 0.50), 0.633
Adjusted Item #1 Scale Rating Mean at Day 180 (95% CI) (Exploratory Endpoint)**	4.04 (3.51, 4.56)	3.84 (3.33, 4.34)	3.72 (3.13, 4.32)
vs Control (95% CI), p-value	0.31 (-0.47, 1.09), 0.435	0.11 (-0.65, 0.87), 0.773	-
vs PAXLOVID 15 Day (95% CI), p-value	0.20 (-0.51, 0.91), 0.583	-	-0.11 (-0.87, 0.65), 0.773
vs PAXLOVID 25 Day (95% CI), p-value	-	-0.20 (-0.91, 0.51), 0.583	-0.31 (-1.09, 0.47), 0.435
Adjusted Overall OHQ Composite Score Mean at Day 180 (95% CI) (Exploratory Endpoint)**	4.31 (3.79, 4.84)	4.30 (3.82, 4.77)	3.78 (3.28, 4.28)
vs Control (95% CI), p-value	0.53 (-0.16, 1.23), 0.131	0.52 (-0.12, 1.16), 0.112	-

	PAXLOVID 25 Day	PAXLOVID 15 Day	Control
vs PAXLOVID 15 Day (95% CI), p-value	0.02 (-0.67, 0.70), 0.965	-	-0.52 (-1.16, 0.12), 0.112
vs PAXLOVID 25 Day (95% CI), p-value	-	-0.02 (-0.70, 0.67), 0.965	-0.53 (-1.23, 0.16), 0.131

Panel C: Exercise Intolerance Symptom Cluster

	PAXLOVID 25 Day	PAXLOVID 15 Day	Control
**Exercise Intolerance Symptom Cluster - Post-Exertional Malaise***	N = 112	N = 105	N = 115
Adjusted Mean number of symptoms of moderate or greater severity with 50% or more frequency at Day 180 (95% CI) (Exploratory Endpoint)**	2.99 (2.58, 3.39)	2.52 (2.12, 2.93)	2.55 (2.15, 2.95)
vs Control (95% CI), p-value	0.44 (-0.12, 1.00), 0.122	-0.03 (-0.58, 0.53), 0.930	-
vs PAXLOVID 15 Day (95% CI), p-value	0.47 (-0.10, 1.03), 0.105	-	0.03 (-0.53, 0.58), 0.930
vs PAXLOVID 25 Day (95% CI), p-value	-	-0.47 (-1.03, 0.10), 0.105	-0.44 (-1.00, 0.12), 0.122

CI: Confidence Interval

*The numbers in this row report the number of participants by treatment that are in the mITT population for the symptom cluster corresponding to the endpoint or endpoints analyzed for the symptom cluster.

**Estimation of adjusted means and corresponding differences were performed using the G-computation method that combines least squares linear regression with direct standardization (See SAP Section 8.4.1 for details). The following covariates used in the analyses are as follows: baseline result of continuous outcome measure used for the comparison of the adjusted mean difference, race (black, white, other) (categorical), years of education (continuous), and US region (Northeast, Midwest, South, West) (categorical).

Table S8: Analyses of Primary Endpoint in a Continuous Manner

Panel A: Cognitive Dysfunction Symptom Cluster

	PAXLOVID 25 Day	PAXLOVID 15 Day	Control
Cognitive Dysfunction Symptom Cluster - PROMIS Cognitive 8a*	N = 110	N = 121	N = 101
Adjusted T-Score Mean at Day 180 (95% CI) (Exploratory Endpoint)**	37.01 (35.30, 38.71)	36.33 (34.58, 38.08)	37.26 (35.32, 39.20)
vs Control (95% CI), p-value	-0.26 (-2.68, 2.17), 0.835	-0.94 (-3.40, 1.53), 0.456	-
vs PAXLOVID 15 Day (95% CI), p-value	0.68 (-1.64, 3.00), 0.567	-	0.94 (-1.53, 3.40), 0.456
vs PAXLOVID 25 Day (95% CI), p-value	-	-0.68 (-3.00, 1.64), 0.567	0.26 (-2.17, 2.68), 0.835

Panel B: Autonomic Dysfunction Symptom Cluster

	PAXLOVID 25 Day	PAXLOVID 15 Day	Control
Autonomic Dysfunction Symptom Cluster - Orthostatic Hypotension Questionnaire*	N = 121	N = 103	N = 110
Adjusted Overall OHQ Composite Score Mean at Day 90 (95% CI) (Exploratory Endpoint)**	4.36 (3.87, 4.85)	4.33 (3.86, 4.80)	4.20 (3.72, 4.68)
vs Control (95% CI), p-value	0.16 (-0.50, 0.82), 0.633	0.13 (-0.51, 0.76), 0.692	-
vs PAXLOVID 15 Day (95% CI), p-value	0.03 (-0.62, 0.69), 0.922	-	-0.13 (-0.76, 0.51), 0.692
vs PAXLOVID 25 Day (95% CI), p-value	-	-0.03 (-0.69, 0.62), 0.922	-0.16 (-0.82, 0.50), 0.633
Adjusted Item #1 Scale Rating Mean at Day 180 (95% CI) (Exploratory Endpoint)**	4.04 (3.51, 4.56)	3.84 (3.33, 4.34)	3.72 (3.13, 4.32)
vs Control (95% CI), p-value	0.31 (-0.47, 1.09), 0.435	0.11 (-0.65, 0.87), 0.773	-
vs PAXLOVID 15 Day (95% CI), p-value	0.20 (-0.51, 0.91), 0.583	-	-0.11 (-0.87, 0.65), 0.773
vs PAXLOVID 25 Day (95% CI), p-value	-	-0.20 (-0.91, 0.51), 0.583	-0.31 (-1.09, 0.47), 0.435
Adjusted Overall OHQ Composite Score Mean at Day 180 (95% CI) (Exploratory Endpoint)**	4.31 (3.79, 4.84)	4.30 (3.82, 4.77)	3.78 (3.28, 4.28)
vs Control (95% CI), p-value	0.53 (-0.16, 1.23), 0.131	0.52 (-0.12, 1.16), 0.112	-

	PAXLOVID 25 Day	PAXLOVID 15 Day	Control
vs PAXLOVID 15 Day (95% CI), p-value	0.02 (-0.67, 0.70), 0.965	-	-0.52 (-1.16, 0.12), 0.112
vs PAXLOVID 25 Day (95% CI), p-value	-	-0.02 (-0.70, 0.67), 0.965	-0.53 (-1.23, 0.16), 0.131

Panel C: Exercise Intolerance Symptom Cluster

	PAXLOVID 25 Day	PAXLOVID 15 Day	Control
Exercise Intolerance Symptom Cluster - Post-Exertional Malaise*	N = 112	N = 105	N = 115
Adjusted Mean number of symptoms of moderate or greater severity with 50% or more frequency at Day 180 (95% CI) (Exploratory Endpoint)**	2.99 (2.58, 3.39)	2.52 (2.12, 2.93)	2.55 (2.15, 2.95)
vs Control (95% CI), p-value	0.44 (-0.12, 1.00), 0.122	-0.03 (-0.58, 0.53), 0.930	-
vs PAXLOVID 15 Day (95% CI), p-value	0.47 (-0.10, 1.03), 0.105	-	0.03 (-0.53, 0.58), 0.930
vs PAXLOVID 25 Day (95% CI), p-value	-	-0.47 (-1.03, 0.10), 0.105	-0.44 (-1.00, 0.12), 0.122

CI: Confidence Interval

*The numbers in this row report the number of participants by treatment that are in the mITT population for the symptom cluster corresponding to the endpoint or endpoints analyzed for the symptom cluster.

**Estimation of adjusted means and corresponding differences were performed using the G-computation method that combines least squares linear regression with direct standardization (See SAP Section 8.4.1 for details). The following covariates used in the analyses are as follows: baseline result of continuous outcome measure used for the comparison of the adjusted mean difference, race (black, white, other) (categorical), years of education (continuous), and US region (Northeast, Midwest, South, West) (categorical).

Table S9: Summary Statistics at Multiple Time Points, MITT

Panel A: PROMISE 8a: Cognitive Dysfunction Symptom Cluster

	PAXLOVID 25 Day N = 110	PAXLOVID 15 Day N = 121	Control N = 101
Change from Baseline in T-Score			
n	91	113	88
Mean (SD)	5.01 (6.111)	4.61 (7.084)	5.25 (6.633)
Median (Q1, Q3)	4.7 (0.0, 8.8)	3.3 (0.0, 8.8)	4.0 (0.0, 9.5)
Min, Max	-5.5, 23.3	-12.3, 21.2	-5.9, 24.7
End of Dosing Clinic Visit			
Total Raw Score			
n	98	114	94
Mean (SD)	18.3 (8.38)	18.7 (8.61)	18.6 (8.04)
Median (Q1, Q3)	17 (11, 24)	18 (10, 25)	18 (12, 24)
Min, Max	8, 40	8, 40	8, 40
T-Score			
n	98	114	95
Mean (SD)	35.11 (8.570)	35.37 (8.519)	35.08 (8.455)
Median (Q1, Q3)	34.8 (28.9, 40.3)	35.6 (27.5, 41.1)	35.6 (28.9, 40.3)
Min, Max	22.4, 63.5	22.4, 63.5	16.0, 63.5
Change from Baseline in T-Score			
n	95	113	93
Mean (SD)	5.92 (6.970)	5.78 (7.830)	5.07 (6.865)
Median (Q1, Q3)	5.0 (0.0, 9.8)	4.4 (0.0, 9.1)	3.3 (0.0, 8.5)
Min, Max	-7.7, 32.3	-9.7, 29.4	-12.9, 24.0

	PAXLOVID 25 Day N = 110	PAXLOVID 15 Day N = 121	Control N = 101
Day 90			
Total Raw Score			
n	106	112	99
Mean (SD)	19.5 (9.29)	20.1 (9.23)	21.1 (9.81)
Median (Q1, Q3)	18 (11, 26)	20 (12, 28)	20 (12, 29)
Min, Max	8, 40	8, 40	8, 40
T-Score			
n	106	112	99
Mean (SD)	36.22 (9.535)	36.57 (9.319)	37.81 (10.717)
Median (Q1, Q3)	35.6 (28.9, 41.9)	37.2 (29.5, 43.6)	37.2 (30.1, 44.4)
Min, Max	22.4, 63.5	22.4, 63.5	22.4, 63.5
Change from Baseline in T-Score			
n	102	111	97
Mean (SD)	6.75 (8.075)	6.96 (8.155)	7.23 (9.113)
Median (Q1, Q3)	5.9 (0.0, 10.7)	6.3 (0.0, 11.4)	5.5 (0.0, 13.2)
Min, Max	-8.1, 31.3	-5.6, 34.6	-7.3, 37.8
Day 180			
Total Raw Score			
n	102	105	92
Mean (SD)	19.6 (9.20)	20.4 (9.48)	21.3 (10.23)
Median (Q1, Q3)	18 (12, 26)	21 (11, 28)	21 (12, 30)
Min, Max	8, 40	8, 40	8, 40
T-Score			
n	102	105	92
Mean (SD)	36.46 (9.785)	36.81 (9.949)	38.25 (11.098)
Median (Q1, Q3)	35.6 (30.1, 41.9)	37.9 (28.9, 43.6)	37.5 (30.1, 44.9)
Min, Max	22.4, 63.5	22.4, 63.5	22.4, 63.5
Change from Baseline in T-Score			
n	98	104	90
Mean (SD)	7.34 (8.233)	6.95 (9.083)	7.38 (8.945)
Median (Q1, Q3)	6.6 (0.9, 12.3)	6.4 (0.0, 11.4)	6.6 (1.4, 10.7)
Min, Max	-8.8, 32.3	-9.7, 34.6	-10.7, 34.6

Panel B: Orthostatic Hypotension Questionnaire: Autonomic Dysfunction Symptom Cluster

	PAXLOVID 25 Day N = 121	PAXLOVID 15 Day N = 103	Control N = 110
Baseline			
Item #1 Scale Rating			
n	120	103	110
Mean (SD)	6.3 (1.98)	6.1 (1.90)	6.5 (1.67)
Median (Q1, Q3)	6 (5, 8)	6 (4, 7)	7 (5, 8)
Min, Max	0, 10	0, 10	3, 10
OHSA Composite Score			
n	120	103	110
Mean (SD)	6.20 (1.929)	5.82 (1.871)	6.22 (1.648)
Median (Q1, Q3)	6.33 (5.09, 7.50)	5.67 (4.50, 7.17)	6.17 (5.17, 7.33)
Min, Max	0.00, 10.00	1.33, 9.67	2.17, 9.67
OHDAS Composite Score			
n	119	103	110
Mean (SD)	5.72 (2.448)	5.71 (2.502)	6.00 (2.230)
Median (Q1, Q3)	6.00 (4.00, 7.50)	6.50 (3.75, 7.75)	6.00 (4.50, 8.00)
Min, Max	0.00, 10.00	0.00, 9.50	0.50, 10.00
Overall OHQ Composite Score			
n	119	103	110
Mean (SD)	5.96 (1.949)	5.76 (1.981)	6.12 (1.709)
Median (Q1, Q3)	6.09 (4.46, 7.42)	6.21 (4.09, 7.38)	5.98 (4.88, 7.21)
Min, Max	0.00, 9.84	0.67, 9.34	2.71, 9.75
Drug Dosing Midpoint Clinic Visit			
Item #1 Scale Rating			
n	108	95	100
Mean (SD)	4.6 (2.45)	4.4 (2.69)	4.7 (2.31)
Median (Q1, Q3)	5 (3, 6)	5 (2, 7)	4 (3, 7)
Min, Max	0, 10	0, 10	0, 10
Change from Baseline in Item #1 Scale Rating			
n	108	95	100
Mean (SD)	-1.7 (2.70)	-1.8 (2.40)	-1.8 (2.58)
Median (Q1, Q3)	-2 (-3, 0)	-2 (-3, 0)	-2 (-3, 0)
Min, Max	-9, 5	-7, 4	-10, 5
OHSA Composite Score			
n	107	95	100
Mean (SD)	4.78 (2.298)	4.40 (2.391)	4.80 (2.142)
Median (Q1, Q3)	5.00 (2.83, 6.50)	4.50 (2.50, 6.17)	5.09 (3.42, 6.19)

	PAXLOVID 25 Day<br>N = 121	PAXLOVID 15 Day<br>N = 103	Control<br>N = 110
Min, Max	0.00, 9.17	0.00, 9.67	0.00, 9.33
Change from Baseline in OHSA Composite Score			
n	107	95	100
Mean (SD)	-1.45 (2.545)	-1.34 (1.963)	-1.38 (1.978)
Median (Q1, Q3)	-1.17 (-2.66, 0.16)	-1.33 (-2.17, 0.00)	-1.25 (-2.42, -0.08)
Min, Max	-8.17, 7.50	-6.67, 3.17	-7.50, 3.33
OHDAS Composite Score			
n	103	93	100
Mean (SD)	4.95 (2.662)	4.39 (2.674)	5.07 (2.590)
Median (Q1, Q3)	5.00 (3.25, 7.25)	4.25 (2.50, 6.50)	5.00 (3.25, 7.50)
Min, Max	0.00, 10.00	0.00, 10.00	0.00, 10.00
Change from Baseline in OHDAS Composite Score			
n	103	93	100
Mean (SD)	-0.95 (2.402)	-1.32 (2.326)	-0.92 (2.196)
Median (Q1, Q3)	-0.50 (-2.50, 0.50)	-0.75 (-2.50, 0.25)	-1.00 (-2.00, 0.25)
Min, Max	-8.00, 5.50	-8.50, 5.00	-8.50, 7.25
Overall OHQ Composite Score			
n	103	93	100
Mean (SD)	4.91 (2.291)	4.41 (2.408)	4.93 (2.166)
Median (Q1, Q3)	5.34 (3.17, 6.63)	4.34 (2.67, 6.34)	4.82 (3.43, 6.71)
Min, Max	0.00, 8.84	0.00, 9.84	0.00, 9.29
Change from Baseline in Overall OHQ Composite Score			
n	103	93	100
Mean (SD)	-1.18 (2.196)	-1.36 (1.937)	-1.15 (1.935)
Median (Q1, Q3)	-0.88 (-2.42, 0.25)	-1.00 (-2.37, 0.00)	-1.23 (-2.25, 0.13)
Min, Max	-8.09, 3.50	-6.34, 3.16	-7.17, 5.21
OHDAS Composite Score			
n	101	93	102
Mean (SD)	4.92 (2.807)	4.47 (2.937)	4.60 (2.642)
Median (Q1, Q3)	5.00 (3.00, 7.25)	4.50 (2.00, 6.75)	4.88 (2.50, 6.75)
Min, Max	0.00, 10.00	0.00, 10.00	0.00, 10.00
Change from Baseline in OHDAS Composite Score			
n	101	93	102
Mean (SD)	-1.05 (2.864)	-1.34 (2.434)	-1.48 (2.367)

	PAXLOVID 25 Day<br>N = 121	PAXLOVID 15 Day<br>N = 103	Control<br>N = 110
Median (Q1, Q3)	-1.00 (-2.75, 0.75)	-1.00 (-2.75, 0.00)	-1.25 (-2.75, 0.00)
Min, Max	-10.00, 4.50	-8.50, 5.50	-8.50, 3.50
Overall OHQ Composite Score			
n	101	93	102
Mean (SD)	4.80 (2.469)	4.46 (2.591)	4.57 (2.342)
Median (Q1, Q3)	5.21 (2.96, 6.71)	4.38 (2.25, 6.45)	4.62 (2.92, 6.63)
Min, Max	0.00, 9.46	0.00, 9.21	0.00, 9.25
Change from Baseline in Overall OHQ Composite Score			
n	101	93	102
Mean (SD)	-1.34 (2.563)	-1.39 (2.085)	-1.57 (2.153)
Median (Q1, Q3)	-0.74 (-2.60, 0.33)	-1.21 (-2.50, -0.12)	-1.36 (-2.79, -0.21)
Min, Max	-9.17, 4.00	-7.33, 3.33	-8.08, 3.04
Day 90			
Item #1 Scale Rating			
n	117	96	104
Mean (SD)	4.0 (2.79)	3.8 (2.68)	3.9 (2.82)
Median (Q1, Q3)	4 (2, 6)	4 (2, 6)	3 (1, 6)
Min, Max	0, 10	0, 9	0, 10
Change from Baseline in Item #1 Scale Rating			
n	117	96	104
Mean (SD)	-2.3 (3.08)	-2.4 (2.62)	-2.6 (2.87)
Median (Q1, Q3)	-2 (-5, 0)	-2 (-4, 0)	-3 (-5, -1)
Min, Max	-10, 4	-9, 3	-10, 3
OHSA Composite Score			
n	116	96	104
Mean (SD)	4.33 (2.704)	4.15 (2.622)	4.12 (2.679)
Median (Q1, Q3)	4.50 (2.00, 6.50)	4.29 (2.00, 6.50)	4.00 (1.92, 6.42)
Min, Max	0.00, 9.17	0.00, 9.17	0.00, 10.00
Change from Baseline in OHSA Composite Score			
n	116	96	104
Mean (SD)	-1.94 (3.026)	-1.66 (2.379)	-2.07 (2.498)
Median (Q1, Q3)	-1.70 (-3.30, 0.00)	-1.50 (-3.00, 0.00)	-1.83 (-3.83, -0.17)
Min, Max	-8.50, 7.50	-7.50, 4.92	-8.00, 3.00
OHDAS Composite Score			
n	112	94	104
Mean (SD)	4.51 (3.115)	4.27 (3.040)	4.43 (3.042)

	PAXLOVID 25 Day N = 121	PAXLOVID 15 Day N = 103	Control N = 110
Median (Q1, Q3)	4.75 (1.38, 7.42)	4.25 (2.00, 7.00)	4.38 (1.88, 7.13)
Min, Max	0.00, 10.00	0.00, 10.00	0.00, 10.00
Change from Baseline in OHDAS Composite Score			
n	112	94	104
Mean (SD)	-1.44 (2.990)	-1.53 (2.711)	-1.54 (2.730)
Median (Q1, Q3)	-1.00 (-3.25, 0.63)	-1.00 (-3.00, 0.00)	-1.25 (-3.25, 0.25)
Min, Max	-8.50, 5.50	-8.25, 5.50	-8.75, 4.25
Overall OHQ Composite Score			
n	112	94	104
Mean (SD)	4.45 (2.745)	4.21 (2.669)	4.28 (2.746)
Median (Q1, Q3)	4.92 (2.07, 6.84)	4.48 (1.85, 6.50)	4.11 (2.09, 6.49)
Min, Max	0.00, 9.00	0.00, 8.92	0.00, 9.79
Change from Baseline in Overall OHQ Composite Score			
n	112	94	104
Mean (SD)	-1.69 (2.826)	-1.63 (2.249)	-1.80 (2.464)
Median (Q1, Q3)	-1.44 (-3.42, 0.33)	-1.31 (-3.16, -0.04)	-1.42 (-3.44, -0.02)
Min, Max	-8.42, 5.75	-7.79, 3.16	-8.30, 3.05
Day 180			
Item #1 Scale Rating			
n	109	90	97
Mean (SD)	4.0 (2.91)	3.6 (2.79)	3.9 (3.13)
Median (Q1, Q3)	4 (1, 6)	3 (1, 6)	3 (1, 7)
Min, Max	0, 10	0, 10	0, 10
Change from Baseline in Item #1 Scale Rating			
n	109	90	97
Mean (SD)	-2.3 (3.20)	-2.5 (2.52)	-2.7 (3.13)
Median (Q1, Q3)	-2 (-5, 0)	-3 (-4, -1)	-3 (-5, 0)
Min, Max	-9, 5	-9, 4	-10, 4
OHSA Composite Score			
n	108	90	97
Mean (SD)	4.19 (2.766)	3.97 (2.670)	3.81 (2.800)
Median (Q1, Q3)	4.42 (1.70, 6.33)	4.00 (1.75, 6.17)	3.60 (1.33, 6.33)
Min, Max	0.00, 9.17	0.00, 9.50	0.00, 9.83
Change from Baseline in OHSA Composite Score			
n	108	90	97
Mean (SD)	-2.09 (3.090)	-1.82 (2.358)	-2.42 (2.517)

	PAXLOVID 25 Day N = 121	PAXLOVID 15 Day N = 103	Control N = 110
Median (Q1, Q3)	-1.68 (-3.84, -0.15)	-1.97 (-3.16, -0.27)	-2.34 (-4.00, -0.50)
Min, Max	-8.50, 8.00	-8.50, 5.17	-8.17, 2.00
OHDAS Composite Score			
n	104	88	97
Mean (SD)	4.56 (3.162)	4.36 (2.933)	4.13 (3.130)
Median (Q1, Q3)	4.88 (1.75, 7.00)	4.88 (1.84, 6.38)	4.25 (1.00, 7.00)
Min, Max	0.00, 10.00	0.00, 9.50	0.00, 10.00
Change from Baseline in OHDAS Composite Score			
n	104	88	97
Mean (SD)	-1.46 (3.067)	-1.41 (2.582)	-1.97 (2.602)
Median (Q1, Q3)	-1.13 (-3.25, 0.50)	-1.13 (-2.63, 0.04)	-1.75 (-3.75, 0.00)
Min, Max	-8.00, 5.42	-8.25, 4.50	-9.00, 4.75
Overall OHQ Composite Score			
n	104	88	97
Mean (SD)	4.39 (2.833)	4.15 (2.694)	3.97 (2.817)
Median (Q1, Q3)	5.19 (1.80, 6.60)	4.45 (1.82, 5.94)	3.84 (1.28, 6.38)
Min, Max	0.00, 9.50	0.00, 8.92	0.00, 9.42
Change from Baseline in Overall OHQ Composite Score			
n	104	88	97
Mean (SD)	-1.78 (2.911)	-1.67 (2.191)	-2.20 (2.389)
Median (Q1, Q3)	-1.46 (-3.21, 0.20)	-1.44 (-2.84, -0.40)	-2.08 (-3.56, -0.41)
Min, Max	-8.25, 6.50	-7.79, 3.33	-8.59, 1.71

Panel C: DePaul Symptom Questionnaire Post Exertional Malaise (DSQ-PEM), Exercise Intolerance Symptom Cluster

	PAXLOVID 25 Day N = 112	PAXLOVID 15 Day N = 105	Control N = 115
Baseline			
Observed number of symptoms of moderate or greater severity with 50% or more frequency			
n	112	105	115
Mean (SD)	4.0 (1.27)	3.7 (1.36)	3.8 (1.32)
Median (Q1, Q3)	5 (3, 5)	4 (3, 5)	4 (3, 5)
Min, Max	1, 5	0, 5	1, 5
At least one moderate or greater severity symptom with 50% or more frequency	112 / 112 (100%)	104 / 104 (100%)	115 / 115 (100%)
Drug Dosing Midpoint Clinic Visit			
Observed number of symptoms of moderate or greater severity with 50% or more frequency			
n	93	92	98
Mean (SD)	3.4 (1.89)	2.7 (2.09)	2.5 (1.99)
Median (Q1, Q3)	4 (2, 5)	3 (0, 5)	3 (0, 4)
Min, Max	0, 5	0, 5	0, 5
Change from Baseline			
n	93	92	98
Mean (SD)	-0.6 (1.87)	-1.0 (2.08)	-1.3 (2.07)
Median (Q1, Q3)	0 (-1, 0)	0 (-2, 0)	-1 (-3, 0)
Min, Max	-5, 4	-5, 3	-5, 4
End of Dosing Clinic Visit			
Observed number of symptoms of moderate or greater severity with 50% or more frequency			
n	105	96	108
Mean (SD)	3.0 (1.93)	2.7 (2.15)	2.5 (2.00)
Median (Q1, Q3)	4 (1, 5)	4 (0, 5)	3 (0, 5)
Min, Max	0, 5	0, 5	0, 5
Change from Baseline			
n	105	96	108
Mean (SD)	-1.1 (2.05)	-1.0 (2.18)	-1.3 (2.14)
Median (Q1, Q3)	0 (-2, 0)	-1 (-2, 0)	-1 (-3, 0)
Min, Max	-5, 3	-5, 3	-5, 4
Day 90			
Observed number of symptoms of moderate or greater severity with 50% or more frequency			

	PAXLOVID 25 Day<br>N = 112	PAXLOVID 15 Day<br>N = 105	Control<br>N = 115
n	106	101	110
Mean (SD)	3.0 (2.11)	2.4 (2.11)	2.5 (2.09)
Median (Q1, Q3)	4 (0, 5)	2 (0, 5)	3 (0, 5)
Min, Max	0, 5	0, 5	0, 5
Change from Baseline			
n	106	101	110
Mean (SD)	-1.0 (2.25)	-1.3 (2.18)	-1.4 (2.09)
Median (Q1, Q3)	0 (-3, 0)	-1 (-3, 0)	-1 (-3, 0)
Min, Max	-5, 3	-5, 3	-5, 3
Day 180			
Observed number of symptoms of moderate or greater severity with 50% or more frequency			
n	103	95	105
Mean (SD)	3.0 (2.17)	2.4 (2.10)	2.6 (2.15)
Median (Q1, Q3)	4 (0, 5)	2 (0, 5)	3 (0, 5)
Min, Max	0, 5	0, 5	0, 5
Change from Baseline			
n	103	95	105
Mean (SD)	-1.0 (2.30)	-1.2 (2.16)	-1.3 (2.18)
Median (Q1, Q3)	-1 (-3, 0)	-1 (-3, 0)	-1 (-3, 0)
Min, Max	-5, 3	-5, 3	-5, 4

OHSA: Orthostatic Hypotension Symptom Assessment; OHDAS: Orthostatic Hypotension Daily Activity Scale

Table S10: Main Analysis Results for Primary Endpoints by Symptom Cluster: High Study Drug Adherence Population

Panel A: Cognitive Dysfunction Symptom Cluster

	PAXLOVID 25 Day	PAXLOVID 15 Day	Control
Cognitive Dysfunction Symptom Cluster - PROMIS Cognitive 8a at Day 90*	N = 106	N = 108	N = 92
Improvement at Day 90 (Primary Endpoint)			
Unadjusted Proportion**	56 / 99 (56.6%)	57 / 103 (55.3%)	51 / 89 (57.3%)
Adjusted Proportion***	56.7% (46.9%, 66.4%)	55.0% (45.5%, 64.4%)	57.2% (47.0%, 67.3%)
vs Control (95% CI), p-value	-0.5% (-14.6%, 13.6%), 0.945	-2.2% (-16.0%, 11.6%), 0.756	-
vs PAXLOVID 15 Day (95% CI), p-value	1.7% (-11.8%, 15.3%), 0.806	-	2.2% (-11.6%, 16.0%), 0.756
vs PAXLOVID 25 Day (95% CI), p-value	-	-1.7% (-15.3%, 11.8%), 0.806	0.5% (-13.6%, 14.6%), 0.945
Adjusted Mean Difference in T-Score (Exploratory Endpoint)***	36.71 (35.04, 38.38)	36.56 (34.98, 38.13)	37.57 (35.74, 39.41)
vs Control (95% CI), p-value	-0.86 (-3.22, 1.49), 0.472	-1.02 (-3.30, 1.27), 0.383	-
vs PAXLOVID 15 Day (95% CI), p-value	0.15 (-2.00, 2.31), 0.890	-	1.02 (-1.27, 3.30), 0.383
vs PAXLOVID 25 Day (95% CI), p-value	-	-0.15 (-2.31, 2.00), 0.890	0.86 (-1.49, 3.22), 0.472

Panel B: Autonomic Dysfunction Symptom Cluster

	PAXLOVID 25 Day	PAXLOVID 15 Day	Control
Autonomic Dysfunction Symptom Cluster - Orthostatic Hypotension Questionnaire Question 1 at Day 90*	N = 112	N = 93	N = 100
Improvement at Day 90 (Primary Endpoint)			
Unadjusted Proportion**	69 / 110 (62.7%)	61 / 88 (69.3%)	70 / 96 (72.9%)
Adjusted Proportion***	63.4% (54.6%, 72.1%)	68.5% (59.2%, 77.7%)	72.8% (63.9%, 81.7%)
vs Control (95% CI), p-value	-9.4% (-21.8%, 3.0%), 0.137	-4.3% (-17.0%, 8.4%), 0.508	-
vs PAXLOVID 15 Day (95% CI), p-value	-5.1% (-17.6%, 7.4%), 0.424	-	4.3% (-8.4%, 17.0%), 0.508
vs PAXLOVID 25 Day (95% CI), p-value	-	5.1% (-7.4%, 17.6%), 0.424	9.4% (-3.0%, 21.8%), 0.137
Adjusted Mean Difference (Exploratory Endpoint)***	4.03 (3.54, 4.53)	3.91 (3.39, 4.44)	3.68 (3.14, 4.22)
vs Control (95% CI), p-value	0.36 (-0.36, 1.07), 0.331	0.23 (-0.50, 0.97), 0.534	-
vs PAXLOVID 15 Day (95% CI), p-value	0.12 (-0.59, 0.83), 0.735	-	-0.23 (-0.97, 0.50), 0.534
vs PAXLOVID 25 Day (95% CI), p-value	-	-0.12 (-0.83, 0.59), 0.735	-0.36 (-1.07, 0.36), 0.331

Panel C: Exercise Intolerance Symptom Cluster

	PAXLOVID 25 Day	PAXLOVID 15 Day	Control
Exercise Intolerance Symptom Cluster - Post-Exertional Malaise at Day 90*	N = 103	N = 92	N = 102
Improvement at Day 90 (Primary Endpoint)			
Unadjusted Proportion**	27 / 99 (27.3%)	30 / 91 (33.0%)	33 / 98 (33.7%)
Adjusted Proportion***	27.2% (18.6%, 35.8%)	32.8% (23.4%, 42.2%)	34.7% (25.8%, 43.6%)
vs Control (95% CI), p-value	-7.5% (-19.6%, 4.7%), 0.229	-1.9% (-14.7%, 10.9%), 0.769	-
vs PAXLOVID 15 Day (95% CI), p-value	-5.5% (-18.1%, 7.1%), 0.389	-	1.9% (-10.9%, 14.7%), 0.769
vs PAXLOVID 25 Day (95% CI), p-value	-	5.5% (-7.1%, 18.1%), 0.389	7.5% (-4.7%, 19.6%), 0.229
Adjusted Mean Difference in number of symptoms of moderate or greater severity with 50% or more frequency (Exploratory Endpoint)***	2.98 (2.58, 3.39)	2.44 (2.05, 2.84)	2.37 (1.97, 2.76)
vs Control (95% CI), p-value	0.61 (0.06, 1.17), 0.030	0.07 (-0.47, 0.62), 0.791	-
vs PAXLOVID 15 Day (95% CI), p-value	0.54 (-0.01, 1.09), 0.056	-	-0.07 (-0.62, 0.47), 0.791
vs PAXLOVID 25 Day (95% CI), p-value	-	-0.54 (-1.09, 0.01), 0.056	-0.61 (-1.17, -0.06), 0.030

CI: Confidence Interval

*The numbers in this row report the number of participants by treatment that are in the mITT population for the symptom cluster corresponding to the endpoint and the high study drug adherence population. Each cluster-specific primary endpoint corresponds to a pre-defined level of improvement in a continuous outcome measure that corresponds to the symptom cluster. Details on the corresponding definitions of improvement can be found in Section 2.2.1 of the study SAP.

**The denominator includes all participants with a non-missing endpoint.

***The treatment pairwise comparisons of adjusted proportions were performed using the G-computation method that combines logistic regression with direct standardization (See SAP Section 8.1 for details). Estimation of adjusted means and corresponding differences were also performed using the G-computation method, but using a least squares linear regression instead of a logistic model (See SAP Section 8.4.1 for details). The following covariates used in the analyses are as follows: baseline result of continuous outcome measure used to define improvement for the corresponding cluster-specific primary endpoint, race (black, white, other) (categorical), years of education (continuous), and US region (Northeast, Midwest, South, West) (categorical).

Table S11: Additional Analyses for Primary Endpoint Symptom Cluster in High Drug Adherence Population

Panel A: Cognitive Dysfunction Symptom Cluster

	PAXLOVID 25 Day	PAXLOVID 15 Day	Control
Cognitive Dysfunction Symptom Cluster - PROMIS Cognitive 8a*	N = 106	N = 108	N = 92
Adjusted T-Score Mean at Day 180 (95% CI) (Exploratory Endpoint)**	36.83 (35.11, 38.55)	36.62 (34.78, 38.45)	37.06 (35.10, 39.03)
vs Control (95% CI), p-value	-0.23 (-2.68, 2.21), 0.852	-0.45 (-2.98, 2.09), 0.729	-
vs PAXLOVID 15 Day (95% CI), p-value	0.22 (-2.16, 2.59), 0.859	-	0.45 (-2.09, 2.98), 0.729
vs PAXLOVID 25 Day (95% CI), p-value	-	-0.22 (-2.59, 2.16), 0.859	0.23 (-2.21, 2.68), 0.852

Panel B: Autonomic Dysfunction Symptom Cluster

	PAXLOVID 25 Day	PAXLOVID 15 Day	Control
Autonomic Dysfunction Symptom Cluster - Orthostatic Hypotension Questionnaire*	N = 112	N = 93	N = 100
Adjusted Overall OHQ Composite Score Mean at Day 90 (95% CI) (Exploratory Endpoint)**	4.29 (3.78, 4.79)	4.37 (3.88, 4.85)	4.07 (3.57, 4.57)
vs Control (95% CI), p-value	0.22 (-0.47, 0.91), 0.533	0.30 (-0.35, 0.95), 0.366	-
vs PAXLOVID 15 Day (95% CI), p-value	-0.08 (-0.76, 0.60), 0.812	-	-0.30 (-0.95, 0.35), 0.366
vs PAXLOVID 25 Day (95% CI), p-value	-	0.08 (-0.60, 0.76), 0.812	-0.22 (-0.91, 0.47), 0.533
Adjusted Item #1 Scale Rating Mean at Day 180 (95% CI) (Exploratory Endpoint)**	4.06 (3.53, 4.60)	3.89 (3.36, 4.43)	3.52 (2.91, 4.12)
vs Control (95% CI), p-value	0.55 (-0.24, 1.34), 0.176	0.38 (-0.40, 1.15), 0.344	-
vs PAXLOVID 15 Day (95% CI), p-value	0.17 (-0.57, 0.91), 0.650	-	-0.38 (-1.15, 0.40), 0.344
vs PAXLOVID 25 Day (95% CI), p-value	-	-0.17 (-0.91, 0.57), 0.650	-0.55 (-1.34, 0.24), 0.176

	PAXLOVID 25 Day	PAXLOVID 15 Day	Control
Adjusted Overall OHQ Composite Score Mean at Day 180 (95% CI) (Exploratory Endpoint)**	4.35 (3.81, 4.89)	4.33 (3.86, 4.81)	3.60 (3.09, 4.10)
vs Control (95% CI), p-value	0.75 (0.04, 1.46), 0.038	0.74 (0.09, 1.38), 0.025	-
vs PAXLOVID 15 Day (95% CI), p-value	0.02 (-0.67, 0.71), 0.964	-	-0.74 (-1.38, -0.09), 0.025
vs PAXLOVID 25 Day (95% CI), p-value	-	-0.02 (-0.71, 0.67), 0.964	-0.75 (-1.46, -0.04), 0.038

Panel C: Exercise Intolerance Symptom Cluster

	PAXLOVID 25 Day	PAXLOVID 15 Day	Control
Exercise Intolerance Symptom Cluster - Post-Exertional Malaise*	N = 103	N = 92	N = 102
Adjusted Mean number of symptoms of moderate or greater severity with 50% or more frequency at Day 180 (95% CI) (Exploratory Endpoint)**	2.96 (2.54, 3.39)	2.62 (2.21, 3.02)	2.45 (2.04, 2.86)
vs Control (95% CI), p-value	0.51 (-0.07, 1.09), 0.082	0.17 (-0.40, 0.73), 0.565	-
vs PAXLOVID 15 Day (95% CI), p-value	0.35 (-0.22, 0.92), 0.233	-	-0.17 (-0.73, 0.40), 0.565
vs PAXLOVID 25 Day (95% CI), p-value	-	-0.35 (-0.92, 0.22), 0.233	-0.51 (-1.09, 0.07), 0.082

CI: Confidence Interval

*The numbers in this row report the number of participants by treatment that are in the mITT population for the symptom cluster corresponding to the endpoint and the high study drug adherence population. Each cluster-specific primary endpoint corresponds to a pre-defined level of improvement in a continuous outcome measure that corresponds to the symptom cluster. Details on the corresponding definitions of improvement can be found in Section 2.2.1 of the study SAP.

**The treatment pairwise comparisons of adjusted proportions were performed using the G-computation method that combines logistic regression with direct standardization (See SAP Section 8.1 for details). Estimation of adjusted means and corresponding differences were also performed using the G-computation method, but using a least squares linear regression instead of a logistic model (See SAP Section 8.4.1 for details). The following covariates used in the analyses are as follows: baseline result of continuous outcome measure used to define improvement for the corresponding cluster-specific primary endpoint, race (black, white, other) (categorical), years of education (continuous), and US region (Northeast, Midwest, South, West) (categorical).

Figure S3: Forest Plot of Heterogeneity of Treatment Effect (HTE) by Symptom Cluster, MITT Population

Panel A: Cognitive Dysfunction Symptom Cluster: PROMIS Cognitive 8a T-score by Subgroup (HTE)*

Sub-Group	Interaction P-Value	N	Adjusted Difference**	95% CI
All Participants		317	-0.45	-2.48, 1.57
One or more SARS-CoV-2 vaccine doses completed	0.835			
No		56	-0.03	-4.94, 4.88
Yes		261	-0.60	-2.78, 1.59
Time from first PASC COVID infection to randomization (grouped by tertiles)	0.577			
<= 23 months		81	1.07	-2.93, 5.07
23 to 38 months		102	-1.32	-4.78, 2.15
> 38 months		91	1.04	-2.82, 4.89
Race	0.577			
Black or African American		31	0.60	-6.25, 7.44
White		258	-0.95	-3.20, 1.30
Other		28	2.53	-3.94, 9.01
Ethnicity	0.975			
Hispanic, Latino or Spanish		36	-0.37	-6.43, 5.68
Not Hispanic, Latino or Spanish		281	-0.47	-2.62, 1.67
Sex assigned at birth	0.304			
Male		97	-2.03	-5.55, 1.49
Female		220	0.22	-2.25, 2.69
Treatment of acute symptoms for first PASC COVID infection	0.719			
No symptoms or participant treated symptoms themselves at home		121	-1.51	-4.75, 1.72
Doctor visit***		130	0.24	-3.01, 3.49
ED visit or hospitalization		66	0.12	-4.24, 4.49

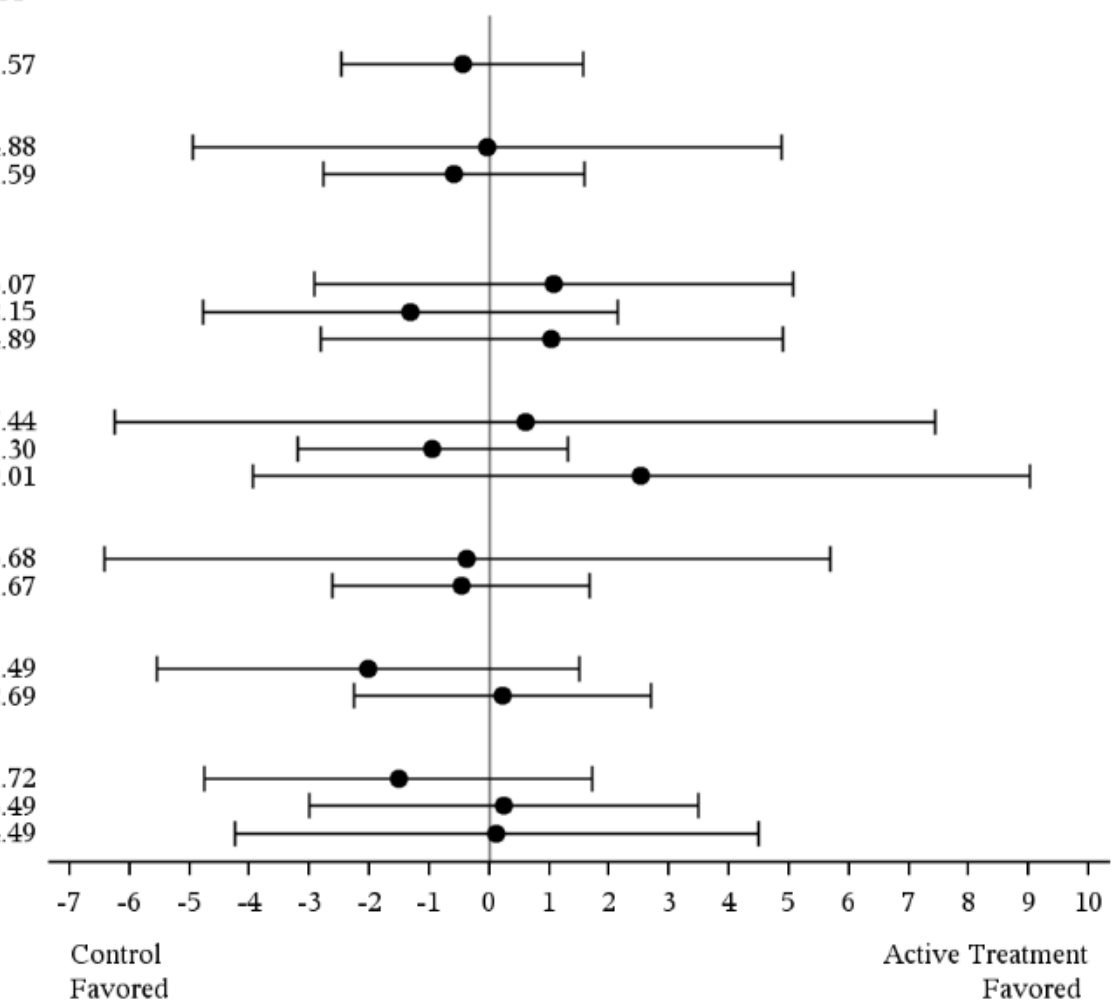

CI: Confidence Interval

*This heterogeneity of treatment effect (HTE) analysis assesses whether the difference in treatment means of the PROMIS Cognitive 8a T-Score at Day 90 differs based on the level of various subgroups. The analysis was accomplished by fitting an ANCOVA linear regression model with adjustment for treatment group (pooled active versus control), baseline measurement of the outcome variable, subgroup variable of interest, and the subgroup × treatment interaction (See SAP Section 8.6 for details). Participants with missing outcome or particular subgroup data were excluded from the analysis for the particular subgroup.

**Presents the adjusted difference between a pooled active treatment group that pooled 15- and 25-day dose durations and the control group. Negative values of this difference indicate that the adjusted mean for the pooled active treatment group is lower than the adjusted mean for the control group.

***In-person clinic visit or telehealth.

Panel B: Autonomic Dysfunction Symptom Cluster: OHQ Question #1 by Subgroup (HTE)*

Sub-Group	Interaction P-Value	N	Adjusted Difference**	95% CI
All Participants		317	0.15	-0.47, 0.78
One or more SARS-CoV-2 vaccine doses completed	0.950			
No		72	0.17	-1.12, 1.45
Yes		245	0.12	-0.60, 0.84
Time from first PASC COVID infection to randomization (grouped by tertiles)	0.091			
<= 23 months		97	-0.26	-1.42, 0.90
23 to 38 months		104	1.13	0.03, 2.23
> 38 months		103	-0.46	-1.52, 0.61
Race	0.958			
Black or African American		44	0.08	-1.56, 1.72
White		240	0.07	-0.64, 0.78
Other		33	0.42	-1.80, 2.64
Ethnicity	0.938			
Hispanic, Latino or Spanish		36	0.27	-1.48, 2.01
Not Hispanic, Latino or Spanish		278	0.19	-0.49, 0.88
Sex assigned at birth	0.627			
Male		94	-0.08	-1.27, 1.10
Female		223	0.26	-0.48, 1.00
Treatment of acute symptoms for first PASC COVID infection	0.884			
No symptoms or participant treated symptoms themselves at home		130	0.04	-0.94, 1.02
Doctor visit***		128	0.34	-0.66, 1.33
ED visit or hospitalization		58	-0.04	-1.53, 1.45

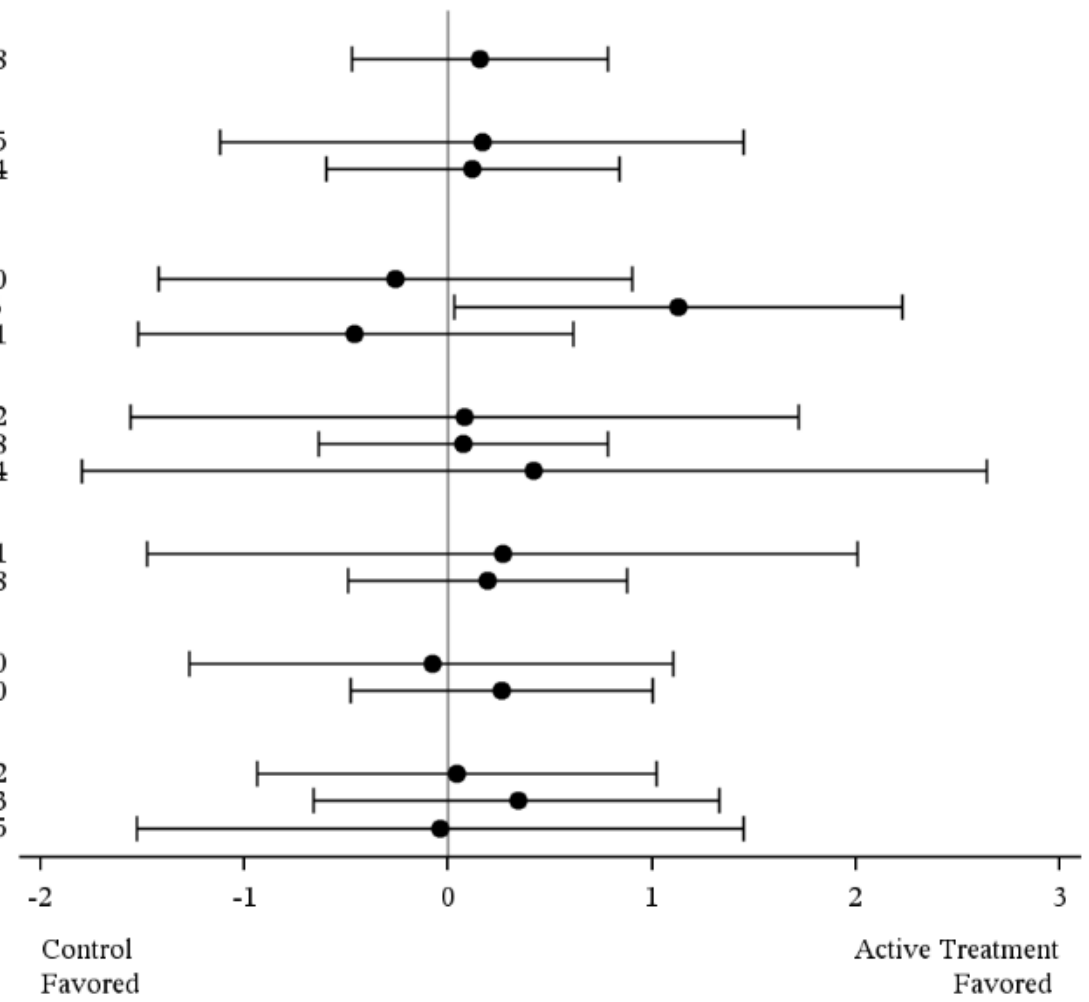

CI: Confidence Interval

*This heterogeneity of treatment effect (HTE) analysis assesses whether the difference in treatment means of the OHQ Item #1 Scale Rating at Day 90 differs based on the level of various subgroups. The analysis was accomplished by fitting an ANCOVA linear regression model with adjustment for treatment group (pooled active versus control), baseline measurement of the outcome variable, subgroup variable of interest, and the subgroup × treatment interaction (See SAP Section 8.6 for details). Participants with missing outcome or particular subgroup data were excluded from the analysis for the particular subgroup.

**Presents the adjusted difference between a pooled active treatment group that pooled 15- and 25-day dose durations and the control group. Negative values of this difference indicate that the adjusted mean for the pooled active treatment group is lower than the adjusted mean for the control group.

***In-person clinic visit or telehealth.

Panel C: Exercise Intolerance Symptom Cluster: DSQ-PEM Criteria for PEM Subgroup (HTE)*

Sub-Group	Interaction P-Value	N	Adjusted Difference**	95% CI
All Participants		317	0.22	-0.25, 0.69
One or more SARS-CoV-2 vaccine doses completed	0.401			
No		67	-0.17	-1.21, 0.87
Yes		250	0.33	-0.20, 0.86
Time from first PASC COVID infection to randomization (grouped by tertiles)	0.986			
<= 23 months		102	0.21	-0.68, 1.10
23 to 38 months		86	0.21	-0.67, 1.09
> 38 months		94	0.12	-0.74, 0.98
Race	0.480			
Black or African American		29	-0.56	-2.10, 0.98
White		260	0.24	-0.29, 0.76
Other		28	0.76	-0.79, 2.32
Ethnicity	0.105			
Hispanic, Latino or Spanish		39	-0.76	-2.07, 0.55
Not Hispanic, Latino or Spanish		275	0.40	-0.11, 0.91
Sex assigned at birth	0.349			
Male		114	-0.10	-0.88, 0.67
Female		201	0.37	-0.23, 0.97
Treatment of acute symptoms for first PASC COVID infection	0.754			
No symptoms or participant treated symptoms themselves at home		130	0.27	-0.48, 1.01
Doctor visit***		120	0.00	-0.78, 0.78
ED visit or hospitalization		64	0.48	-0.56, 1.52

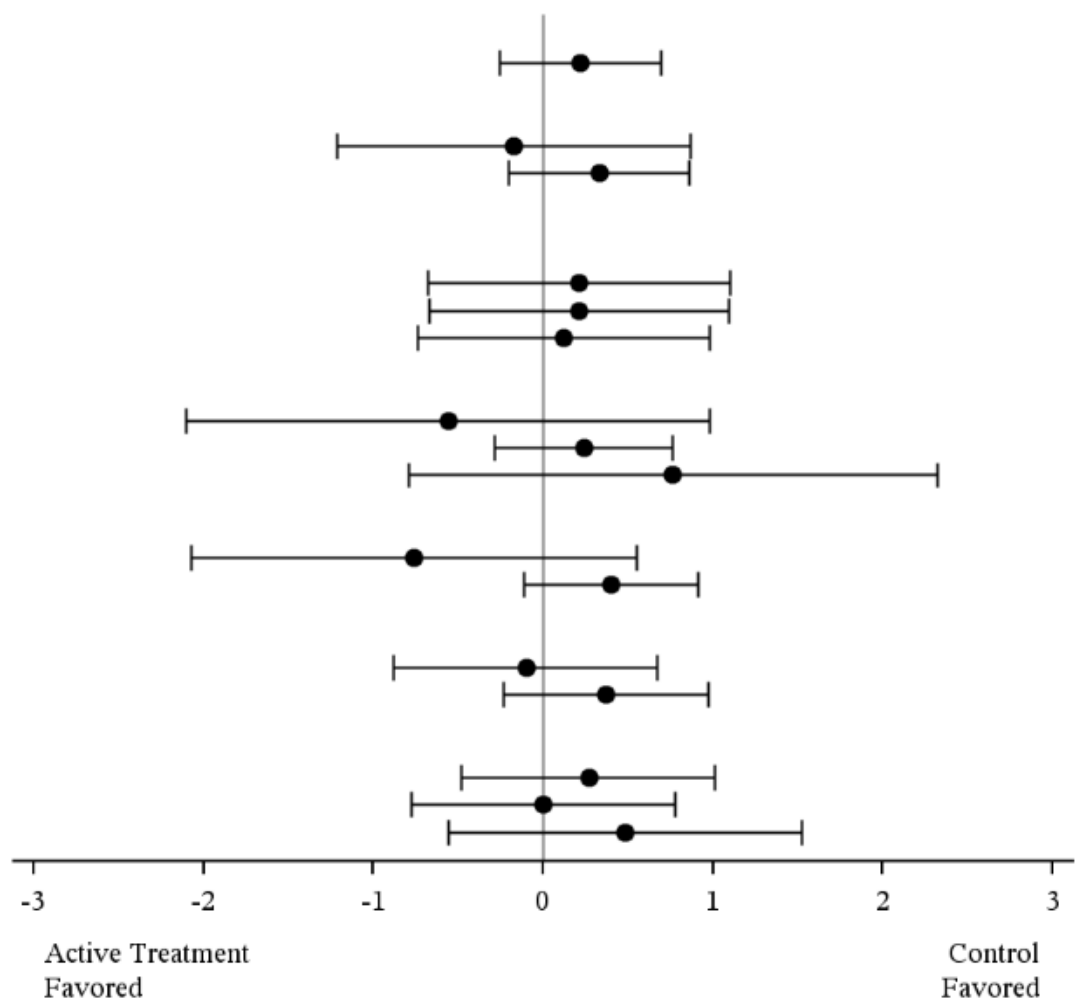

CI: Confidence Interval

*This heterogeneity of treatment effect (HTE) analysis assesses whether the difference in treatment means of the DSQ-PEM Criteria for PEM at Day 90 differs based on the level of various subgroups. The analysis was accomplished by fitting an ANCOVA linear regression model with adjustment for treatment group (pooled active versus control), baseline measurement of the outcome variable, subgroup variable of interest, and the subgroup × treatment interaction (See SAP Section 8.6 for details). Participants with missing outcome or particular subgroup data were excluded from the analysis for the particular subgroup.

**Presents the adjusted difference between a pooled active treatment group that pooled 15- and 25-day dose durations and the control group. Negative values of this difference indicate that the adjusted mean for the pooled active treatment group is lower than the adjusted mean for the control group.

***In-person clinic visit or telehealth.

Table S12: Serious Adverse Events by System Organ and Preferred Term (MedDRA Version 26.1), Safety Population

Subjects with one or more events<br>System Organ Class<br>Preferred Term	PAXLOVID 25 Day<br>N = 323<br>n (%) E	PAXLOVID 15 Day<br>N = 320<br>n (%) E	Control<br>N = 320<br>n (%) E	Overall<br>N = 963<br>n (%) E
Any Serious Adverse Event	13 (4.0%) 17	13 (4.1%) 16	16 (5.0%) 19	42 (4.4%) 52
Infections and infestations	1 (0.3%) 1	3 (0.9%) 3	4 (1.3%) 5	8 (0.8%) 9
Appendicitis	0	1 (0.3%) 1	0	1 (0.1%) 1
COVID-19	0	0	1 (0.3%) 1	1 (0.1%) 1
Cellulitis	0	0	1 (0.3%) 1	1 (0.1%) 1
Diverticulitis	0	1 (0.3%) 1	0	1 (0.1%) 1
Large intestine infection	0	0	1 (0.3%) 1	1 (0.1%) 1
Pneumonia bacterial	0	0	1 (0.3%) 1	1 (0.1%) 1
Sepsis	0	0	1 (0.3%) 1	1 (0.1%) 1
Urosepsis	1 (0.3%) 1	0	0	1 (0.1%) 1
Wound infection	0	1 (0.3%) 1	0	1 (0.1%) 1
Nervous system disorders	4 (1.2%) 5	0	1 (0.3%) 1	5 (0.5%) 6
Cavernous sinus syndrome	1 (0.3%) 1	0	0	1 (0.1%) 1
Cerebrovascular accident	1 (0.3%) 1	0	0	1 (0.1%) 1
Dizziness	1 (0.3%) 1	0	0	1 (0.1%) 1
Facial paralysis	1 (0.3%) 1	0	0	1 (0.1%) 1
Headache	1 (0.3%) 1	0	0	1 (0.1%) 1
Migraine	0	0	1 (0.3%) 1	1 (0.1%) 1
General disorders and administration site conditions	1 (0.3%) 1	1 (0.3%) 1	2 (0.6%) 2	4 (0.4%) 4
Drug intolerance	0	0	1 (0.3%) 1	1 (0.1%) 1
Drug withdrawal syndrome	0	1 (0.3%) 1	0	1 (0.1%) 1
Fatigue	1 (0.3%) 1	0	0	1 (0.1%) 1
Systemic inflammatory response syndrome	0	0	1 (0.3%) 1	1 (0.1%) 1
Hepatobiliary disorders	3 (0.9%) 3	0	1 (0.3%) 1	4 (0.4%) 4
Cholecystitis acute	1 (0.3%) 1	0	1 (0.3%) 1	2 (0.2%) 2
Cholelithiasis	1 (0.3%) 1	0	0	1 (0.1%) 1
Suspected drug-induced liver injury	1 (0.3%) 1	0	0	1 (0.1%) 1
Injury, poisoning and procedural complications	1 (0.3%) 1	1 (0.3%) 1	2 (0.6%) 2	4 (0.4%) 4
Accidental poisoning	0	0	1 (0.3%) 1	1 (0.1%) 1
Hip fracture	0	0	1 (0.3%) 1	1 (0.1%) 1
Post lumbar puncture syndrome	0	1 (0.3%) 1	0	1 (0.1%) 1
Seroma	1 (0.3%) 1	0	0	1 (0.1%) 1

Subjects with one or more events<br>System Organ Class<br>Preferred Term	PAXLOVID 25 Day<br>N = 323<br>n (%) E	PAXLOVID 15 Day<br>N = 320<br>n (%) E	Control<br>N = 320<br>n (%) E	Overall<br>N = 963<br>n (%) E
Psychiatric disorders	1 (0.3%) 2	3 (0.9%) 3	0	4 (0.4%) 5
Suicidal ideation	1 (0.3%) 2	2 (0.6%) 2	0	3 (0.3%) 4
Mental status changes	0	1 (0.3%) 1	0	1 (0.1%) 1
Respiratory, thoracic and mediastinal disorders	0	1 (0.3%) 1	3 (0.9%) 3	4 (0.4%) 4
Acute respiratory failure	0	0	1 (0.3%) 1	1 (0.1%) 1
Dyspnoea	0	0	1 (0.3%) 1	1 (0.1%) 1
Pneumonitis	0	0	1 (0.3%) 1	1 (0.1%) 1
Pulmonary embolism	0	1 (0.3%) 1	0	1 (0.1%) 1
Gastrointestinal disorders	0	1 (0.3%) 2	2 (0.6%) 3	3 (0.3%) 5
Anal fissure	0	0	1 (0.3%) 1	1 (0.1%) 1
Cyclic vomiting syndrome	0	1 (0.3%) 2	0	1 (0.1%) 2
Proctitis	0	0	1 (0.3%) 1	1 (0.1%) 1
Small intestinal perforation	0	0	1 (0.3%) 1	1 (0.1%) 1
Vascular disorders	1 (0.3%) 1	2 (0.6%) 2	0	3 (0.3%) 3
Deep vein thrombosis	0	1 (0.3%) 1	0	1 (0.1%) 1
Hypertension	1 (0.3%) 1	0	0	1 (0.1%) 1
Hypertensive emergency	0	1 (0.3%) 1	0	1 (0.1%) 1
Cardiac disorders	1 (0.3%) 1	1 (0.3%) 1	0	2 (0.2%) 2
Acute myocardial infarction	0	1 (0.3%) 1	0	1 (0.1%) 1
Atrial fibrillation	1 (0.3%) 1	0	0	1 (0.1%) 1
Congenital, familial and genetic disorders	0	1 (0.3%) 1	0	1 (0.1%) 1
Right-to-left cardiac shunt	0	1 (0.3%) 1	0	1 (0.1%) 1
Investigations	0	1 (0.3%) 1	0	1 (0.1%) 1
Transaminases increased	0	1 (0.3%) 1	0	1 (0.1%) 1
Musculoskeletal and connective tissue disorders	1 (0.3%) 1	0	0	1 (0.1%) 1
Muscle spasms	1 (0.3%) 1	0	0	1 (0.1%) 1
Neoplasms benign, malignant and unspecified (incl cysts and polyps)	0	0	1 (0.3%) 1	1 (0.1%) 1
Neoplasm recurrence	0	0	1 (0.3%) 1	1 (0.1%) 1

Subjects with one or more events<br>System Organ Class<br>Preferred Term	PAXLOVID 25 Day<br>N = 323<br>n (%) E	PAXLOVID 15 Day<br>N = 320<br>n (%) E	Control<br>N = 320<br>n (%) E	Overall<br>N = 963<br>n (%) E
Pregnancy, puerperium and perinatal conditions	1 (0.3%) 1	0	0	1 (0.1%) 1
Abortion spontaneous	1 (0.3%) 1	0	0	1 (0.1%) 1
Renal and urinary disorders	0	0	1 (0.3%) 1	1 (0.1%) 1
Acute kidney injury	0	0	1 (0.3%) 1	1 (0.1%) 1

Protocol and SAP

1. Final protocol.

2. Original statistical analysis plan, summary of changes, final statistical analysis plan.

RECOVER-VITAL: A Platform Protocol for Evaluation of Interventions for Viral Persistence, Viral Reactivation, and Immune Dysregulation in Post-Acute Sequelae of SARS-CoV-2 Infection (PASC)

National Clinical Trial (NCT) Identified Number: NCT05595369

Co-Principal Investigators:

Kanecia Zimmerman, MD, PhD, MPH
kanecia.zimmerman@duke.edu

Lindsey Baden, MD
lbaden@bwh.harvard.edu

Richard Whitley, MD
rwhitley@uabmc.edu

IND Sponsor: Kanecia Zimmerman, MD, PhD, MPH

Funded by: National Institutes of Health

Version Number: v4.0

07-NOV-2023

TABLE OF CONTENTS

TITLE PAGE i
TABLE OF CONTENTS ii
LIST OF TABLES v
LIST OF FIGURES v
1 STATEMENT OF COMPLIANCE 1
1.1 Synopsis 1
1.2 Schema for Phase 2 Study Designs 3
2 INTRODUCTION 5
2.1 Study Rationale 5
2.2 Background 5
2.3 Risk/Benefit Assessment 6
2.3.1 Known Potential Risks 6
2.3.2 Known Potential Benefits 7
2.3.3 Assessment of Potential Risks and Benefits 7
3 OBJECTIVES AND ENDPOINTS 8
4 STUDY DESIGN 11
4.1 Overall Design 11
4.2 Scientific Rationale for Study Design 11
4.3 Justification for Dose 12
4.4 End of Study Definition 12
5 STUDY POPULATION 13
5.1 Inclusion Criteria 13
5.2 Exclusion Criteria 13
5.3 Special Considerations for Participant Enrollment 14
5.4 Screen Failures 15
5.5 Study Definition of Enrollment 15
5.6 Strategies for Recruitment and Retention 15
6 STUDY INTERVENTION 16
6.1 Study Intervention(s) Administration 16
6.1.1 Study Intervention Description 16
6.1.2 Dosing and Administration 16
6.2 Preparation/Handling/Storage/Accountability 16
6.2.1 Acquisition and Accountability 16
6.2.2 Formulation, Appearance, Packaging, and Labeling 16
6.2.3 Product Storage and Stability 16
6.2.4 Preparation 16
6.3 Measures to Minimize Bias: Randomization and Blinding 16
6.3.1 Unblinding 17
6.4 Study Intervention Adherence 17
6.5 Concomitant Therapy 17
6.5.1 Symptomatic Therapy 17
6.5.2 Rescue Medicine 18
7 PARTICIPANT DISCONTINUATION/WITHDRAWAL 19
7.1 Participant Discontinuation From Study Drug 19
7.2 Participant Withdrawal from the Study 19
7.3 Lost to Follow-Up 19

7.4 Study Halting Rules 20
8 STUDY ASSESSMENTS AND PROCEDURES FOR PHASE 2 STUDY DESIGNS 21
8.1 Schedule of Procedures 21
8.2 Screening (Day -14 To Day 0) 21
8.3 Baseline (Visit 2, Day -2 to Day 0) 22
8.4 Drug Dosing Midpoint Clinic Visit (Visit 3, DAY 12 to Day 15) 22
8.5 End of Dosing Clinic Visit (Visit 4, DAY 25 to DAY 28) 23
8.6 Follow-Up (End of Dosing [DAY 25 TO DAY 28] THROUGH Day 179) 24
8.7 day 45 optional follow-up (visit 5, visit 45 [day 40 to day 50]) 24
8.8 day 60 optional follow-up (visit 6, day 60 [day 55 to day 65]) 24
8.9 day 90 (visit 7, day 90 [day 85 to day 95]) 24
8.10 DAY 120 optional follow-up (visit 8, Day 120 [day 115 to day 125]) 25
8.11 End of Study Visit (Visit 9, Day 183 ± 7 days) 25
8.12 Optional Blood Collection/Nasal Swab Visits 25
8.13 Clinical Laboratory Assessments 25
8.14 Study Assessments 26
8.14.1 Biospecimen Collection 26
8.14.2 PROMIS-29+2 (PROPr) 27
8.14.3 Cluster Targeted COVID-19 Symptom Questions (CTCSQ) 27
8.14.4 Symptom Cluster Assessment 28
8.14.5 Post-Acute Sequelae of SARS-CoV-2 Infection (PASC) Symptom Questionnaire 31
9 SAFETY ASSESSMENTS AND REPORTING 31
9.1 Adverse Events and Serious Adverse Events 31
9.1.1 Definition of Adverse Events (AE) and Serious Adverse Events (SAE) 31
9.1.2 Collection Period for AE and SAE Information 32
9.1.3 Time Period and Frequency for Event Assessment and Follow-Up 33
9.1.4 Reporting and Monitoring of SAEs 33
9.1.5 Events of Special Interest (ESI) 34
9.1.6 Reporting of Pregnancy 34
10 STATISTICAL CONSIDERATIONS FOR PHASE 2 DESIGN 35
10.1 Statistical Hypotheses 35
10.2 Primary Estimand 35
10.3 Sample Size Determination 36
10.4 Populations for Analyses 37
10.5 Statistical Analyses 37
10.5.1 Primary Efficacy Analysis 38
10.5.2 Analysis of Secondary Endpoints 39
10.5.3 Analysis of Safety Endpoints 40
10.5.4 Analysis of Exploratory Endpoints 40
10.5.5 Exploratory Longitudinal Analyses 40
10.5.6 Exploratory Bayesian Analyses 40
10.5.7 Analysis of Treatment Effect Heterogeneity 41
10.5.8 Missing Data 41
10.5.9 Planned Interim Analysis 41
11 SUPPORTING DOCUMENTATION AND OPERATIONAL CONSIDERATIONS 43
11.1 Regulatory, Ethical, and Study Oversight Considerations 43
11.1.1 Informed Consent Process 43

11.1.2 Study Discontinuation and Closure 43
11.1.3 Confidentiality and Privacy 44
11.1.4 Key Roles and Study Governance 44
11.1.5 Data and Safety Monitoring Board (DSMB) 44
11.1.6 Clinical Monitoring 45
11.1.7 Quality Assurance and Quality Control 45
11.1.8 Data Handling and Record Keeping 45
11.1.9 Protocol Deviations 46
11.1.10 Publication and Data Sharing Policy 46
11.1.11 Conflict of Interest Policy 46
11.2 Abbreviations 47
11.3 Protocol Amendment History 49
12 REFERENCES 52
13 APPENDIX A (PAXLOVID PHASE 2) 55
13.1 Risk Assessment 55
13.2 Additional Appendix-level Exploratory Objective(s) 55
13.3 Additional Appendix-level Exclusion Criteria 56
13.4 Additional Appendix-level Study Assessments 56
13.4.1 Schedule of Procedures 58
13.5 PAXLOVID Information 63
13.5.1 Precautions 63
13.5.2 Formulation, Appearance, Packaging, and Labeling 63
13.5.3 Drug Dispensing, Storage, and Stability 64
13.5.4 Dosing and Administration 64
13.5.5 Rationale for Selection of Dose 65
13.6 Control Information 66
13.6.1 Formulation, Appearance, Packaging, and Labeling 67
13.6.2 Control Dispensing, Storage, and Stability 67
13.6.3 Dosing and Administration 67
13.7 Events of Special Interest (ESI) 67
13.8 Appendix Halting Rules 69

LIST OF TABLES

Table 1. Summary of study objectives, endpoints, and outcome measures. 8
Table 2. CTCSQ items #1-13 and associated symptom cluster(s) 28
Table 3. Minimum treatment effects for 80% or 90% power 37
Table 4. Schedule of Procedures – PAXLOVID, Exercise Intolerance Symptom Cluster 58
Table 5. Schedule of Procedures – PAXLOVID, Cognitive Dysfunction Symptom Cluster 60
Table 6. Schedule of Procedures – PAXLOVID, Autonomic Dysfunction Symptom Cluster 62

LIST OF FIGURES

Figure 1. Study schema for phase 2 study designs 3
Figure 2. Concise study schema 4
Figure 3. Symptom cluster assignment flow. 4
Figure 4. Multiple testing flowchart 38
Figure 5. Effect of PAXLOVID (PF-07321332) on lung viral titers following oral administration in BALB/c mice infected with SARS-COV-2-MA10 66

1 STATEMENT OF COMPLIANCE

The trial will be carried out in accordance with International Conference on Harmonization Good Clinical Practice (ICH GCP) and the following:

- United States (US) Code of Federal Regulations (CFR) applicable to clinical studies (45 CFR Part 46, 21 CFR Part 50, 21 CFR Part 56, 21 CFR Part 312, and/or 21 CFR Part 812)

National Institutes of Health (NIH)-funded investigators and clinical trial site staff who are responsible for the conduct, management, or oversight of NIH-funded clinical trials have completed Human Subjects Protection and ICH GCP Training.

The protocol, informed consent form(s), recruitment materials, and all participant materials will be submitted to the central Institutional Review Board (IRB) for review and approval. Approval of both the protocol and the consent form(s) must be obtained before any participant is enrolled. Any amendment to the protocol will require review and approval by the IRB before the changes are implemented to the study. In addition, all changes to the consent form will be IRB-approved; a determination will be made regarding whether a new consent needs to be obtained from participants who provided consent, using a previously approved consent form.

1.1 SYNOPSIS

**Title:**	RECOVER-VITAL: A Platform Protocol for Evaluation of Interventions for Viral Persistence, Viral Reactivation, and Immune Dysregulation in Post-Acute Sequelae of SARS-CoV-2 Infection (PASC)
**Study Description:**	This study is a platform protocol designed to be flexible so that it is suitable for a wide range of settings within health care systems and community settings where it can be integrated into COVID-19 programs and subsequent treatment plans.<br><br>This protocol describes a prospective, multi-center, multi-arm, double-blind, randomized, controlled, platform trial, with different interventions organized as appendices to the protocol. Each appendix evaluates potential mechanisms of action, efficacy, and safety of antivirals and other therapeutics in individuals with PASC, according to the platform protocol objectives. The hypothesis is that persistent viral infection and/or overactive/chronic immune response and inflammation are underlying contributors to PASC and that antiviral and other applicable therapies may result in viral clearance or decreased inflammation and improvement in PASC symptoms.
**Objectives:**	Primary: Evaluate the effect of intervention(s) versus control on symptom-specific participant-reported outcome (PRO) measures within each PASC symptom cluster at Day 90<br><br>Secondary: Evaluate the effect of intervention(s) versus control on performance-based outcomes within each PASC symptom cluster<br><br>Safety: Characterize the safety and tolerability of intervention(s) in PASC

<table>
<tr><td></td><td>Exploratory:
1. Investigate mechanisms of potential intervention(s) efficacy
2. Compare the effect of intervention(s) on symptom burden and health related quality of life (HRQOL) versus control
3. Evaluate the durability of intervention(s) response
4. Evaluate intervention(s) effect on healthcare utilization
5. Describe the effect of intervention(s) versus control across exploratory performance-based outcomes and PROs within each PASC symptom cluster
6. Determine biomarkers associated with PASC symptom clusters and intervention-associated recovery</td></tr>
<tr><td>Study Population:</td><td>Each study intervention appendix will enroll adult participants who experience at least 1 of 3 predominant symptom clusters: cognitive dysfunction, autonomic dysfunction, or exercise intolerance. Overall enrollment for the platform protocol will depend on the number of screen failures, the number of study intervention appendices that are added, the ability to pool their control groups for analysis, and adjustments to sample size based on study data.

For Phase 2 studies, an estimated sample size of approximately 100 evaluable participants per treatment group per symptom cluster (an appendix with 1 control group and 2 dosing regimens across 3 symptom clusters would have a total of 900 participants) is expected to be sufficient to provide adequate proof-of-concept that will enable optimal design and facilitate rapid transition to phase 3 trials for interventions that demonstrate preliminary evidence of benefit.

The goal is to have a diverse population, including underserved communities and racial/ethnic populations frequently underrepresented in clinical research.</td></tr>
<tr><td>Phase:</td><td>Appendices including phase 2 and phase 3 studies</td></tr>
<tr><td>Description of Sites/Facilities Enrolling Participants:</td><td>Participants may be recruited from acute COVID-19 trials and existing RECOVER initiatives including, but not limited to, the longitudinal cohort, as well as research sites and communities. Approximately 100 sites in the US will participate.</td></tr>
<tr><td>Description of Study Intervention:</td><td>Each study intervention appendix describes a different study drug/drug combination/duration and control. The following arms will be included in each study appendix:
• Study Drug Arm: study drug(s) of interest (intervention(s), see appendices)
• Control Arm: control</td></tr>
<tr><td>Participant Duration:</td><td>The duration of dosing will depend on the intervention. Participants will be in the study for approximately 6 months from the time of initiating study drug administration until their end of study visit.</td></tr>
</table>

1.2 SCHEMA FOR PHASE 2 STUDY DESIGNS

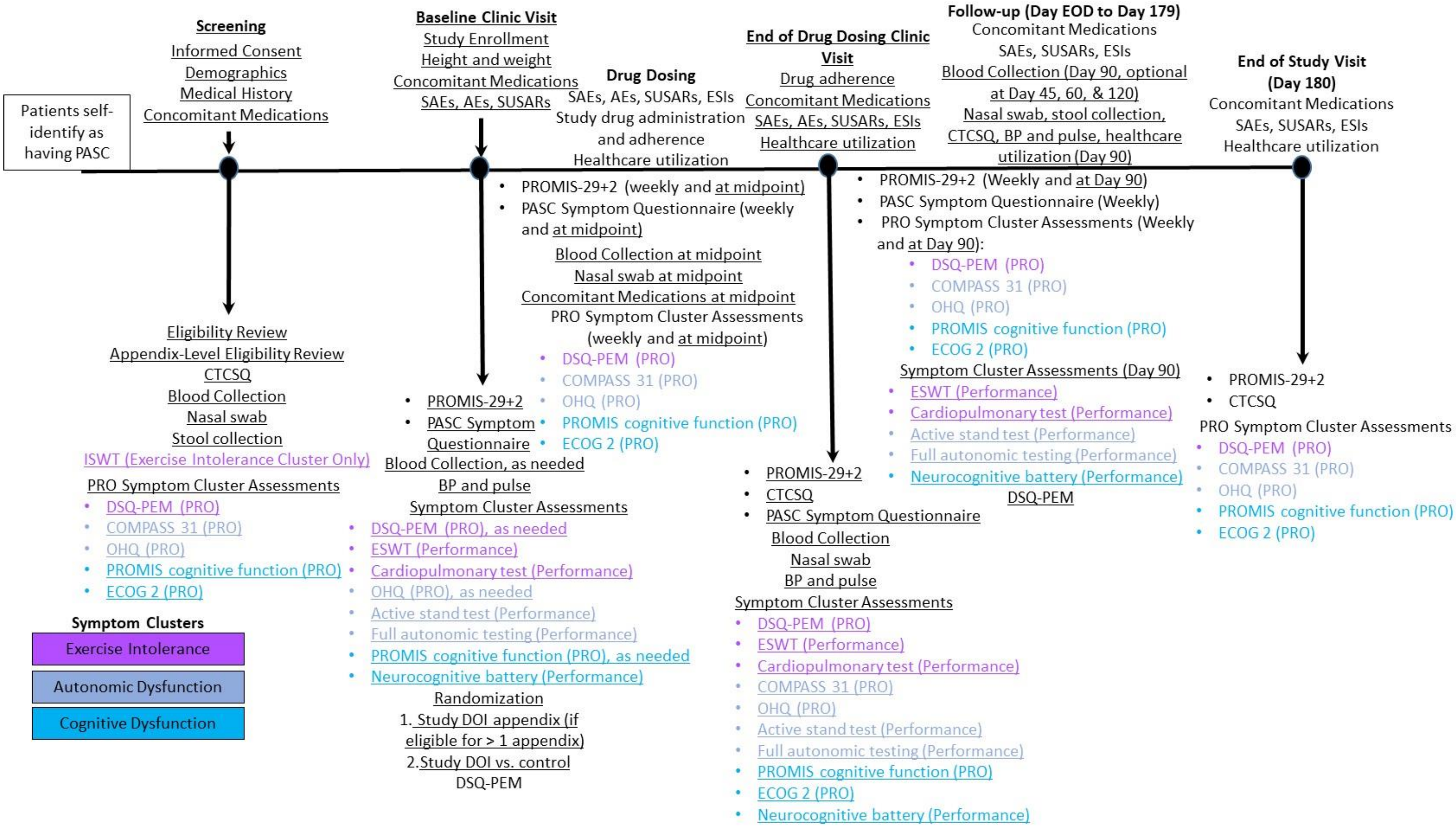

Refer to Section 11.2 for definitions of abbreviations used in Figure 1.

Figure 1. Study schema for phase 2 study designs

Underline in Figure 1 indicates an in-person visit

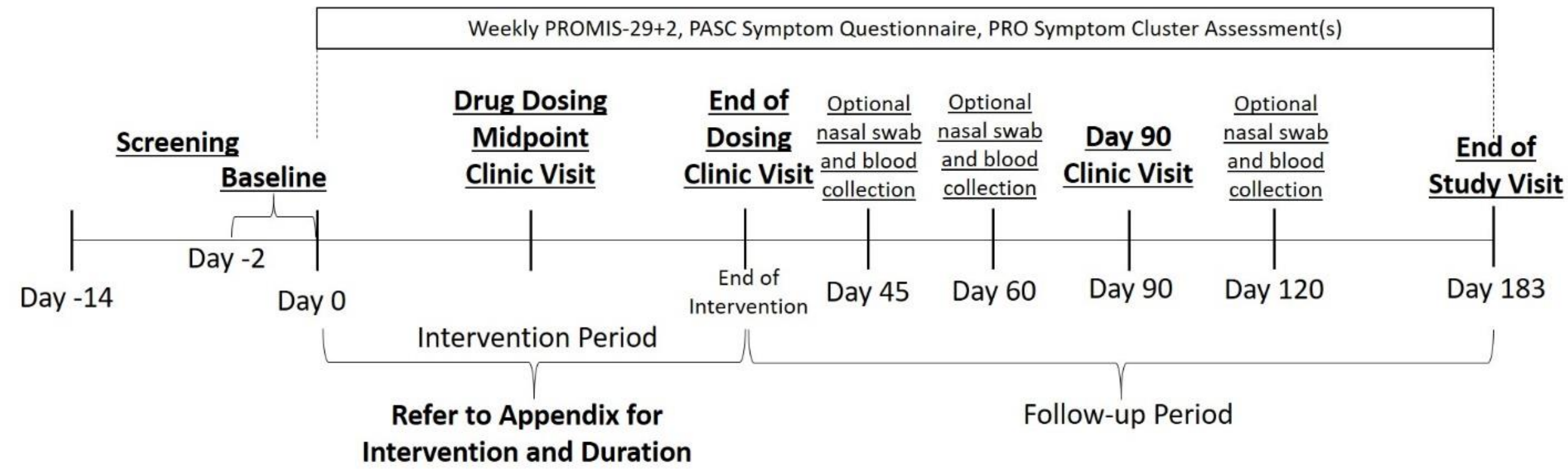

Drug Dosing Midpoint Clinic Visit 3 (Day 15 – 3 days); End of Dosing (EOD) Clinic Visit is Visit 4 (Day 25 + 3 days)

Figure 2. Concise study schema

Underline in Figure 2 indicates and in-person visit where study outcomes and other procedures will be assessed.

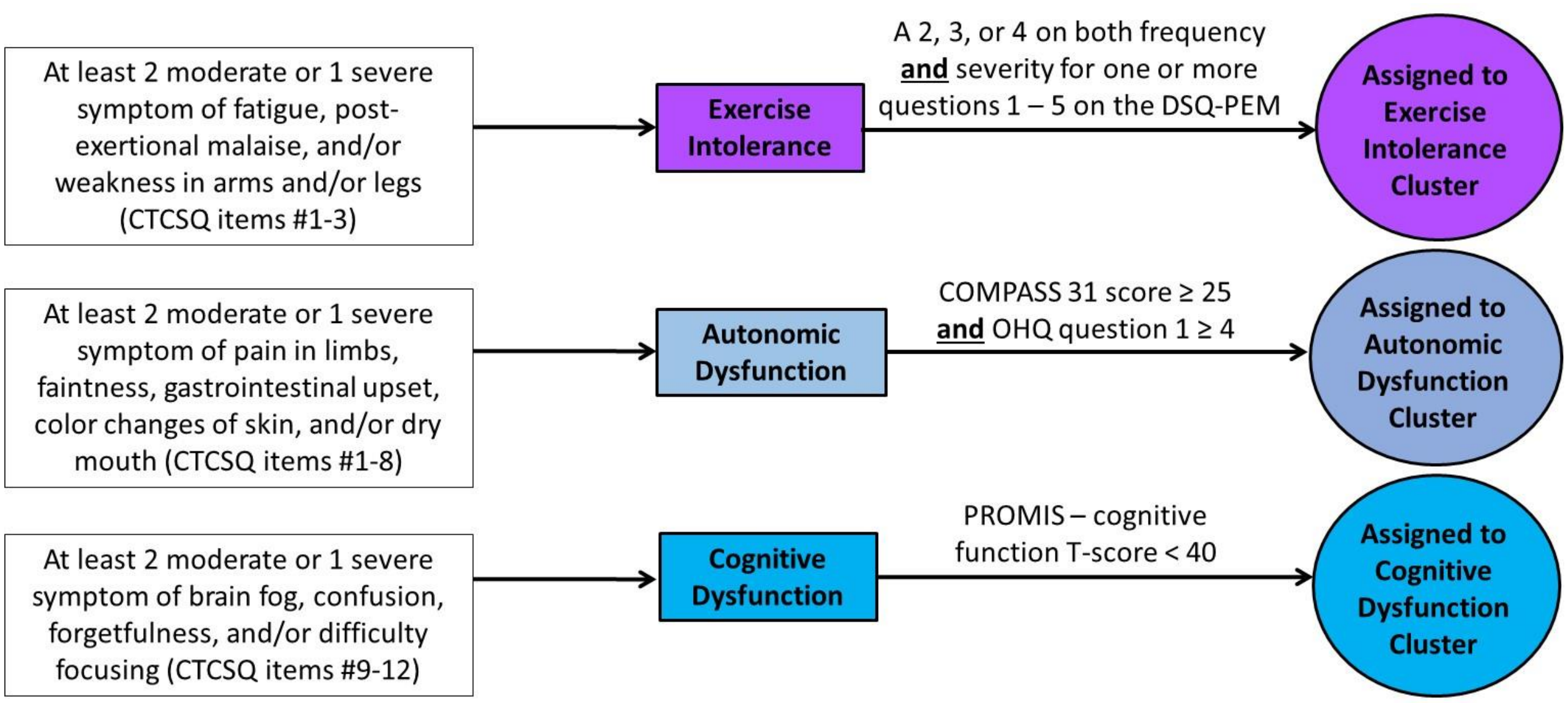

Figure 3. Symptom cluster assignment flow.

Participants who report having at least 2 moderate symptoms from the same symptom cluster or 1 severe symptom from the Cluster Targeted COVID-19 Symptom Questions (CTCSQ) will be screened for symptom clusters based on which symptoms are reported as moderate or severe. Arrows from symptom boxes to cluster boxes indicate which symptom clusters should be assessed based on the moderate or severe symptoms. Arrows from cluster boxes to assignment designation indicate the required scoring of the Patient Reported Outcome (PRO) in order to be assigned to the cluster.

2 INTRODUCTION

2.1 STUDY RATIONALE

Post-acute sequelae of SARS-CoV-2 infection (PASC), also known as Long COVID, is a chronic condition present in up to 80% of SARS-CoV-2-infected, hospitalized patients and 40 to 70% of non-hospitalized patients with COVID-19.[1-4] Symptoms of PASC can be debilitating. The personal and global impact of these long-term symptoms from SARS-CoV-2 infection can be devastating, and the number of Long COVID patients is escalating. With the increasing number of people infected with SARS-CoV-2, an urgent and unmet clinical need exists to better understand the pathophysiology of PASC and to develop targeted therapeutics to resolve the disease more rapidly and restore patients' health. This study aims to investigate various interventions including antivirals, immunomodulatory agents, and other therapeutics or combinations thereof, and evaluate their use in treating PASC, including 1) mechanisms of potential efficacy, 2) safety and tolerability, 3) optimal outcomes and endpoints, and 4) preliminary efficacy. If successful, this trial will rapidly provide the foundational evidence for future phase 3 studies to further establish intervention efficacy in the PASC population.

2.2 BACKGROUND

In 2019, a novel coronavirus-disease (COVID-19) emerged in Wuhan, China. A month later the Chinese Center for Disease Control and Prevention identified a new beta-coronavirus (SARS coronavirus 2, or SARS-CoV-2) as the etiological agent.[5] The clinical manifestations of COVID-19 range from asymptomatic infection or mild, transient symptoms to severe viral pneumonia with respiratory failure.

COVID-19 has led to the death of more than 6 million people worldwide; however, this disease has affected even more lives through often-debilitating symptoms lingering long after acute SARS-CoV-2 infection. Post-acute sequelae of SARS-CoV-2 infection, also known as PASC, is a chronic condition that affects nearly every organ system, with more than 200 individual symptoms, ranging from new-onset anxiety, depression, and cognitive difficulties to shortness of breath, dizziness, and arrhythmias.[6] Moreover, PASC can occur regardless of severity of acute COVID-19 disease, and it impacts across socioeconomic, racial and ethnic, and age strata. These prolonged symptoms open the door for substantial short- and long-term individual and societal costs, including healthcare costs and inability to work. Prolonged symptoms have kept individuals out of work, which has exacerbated poverty in the underserved, historically minoritized populations and worsened a decades-long mental health crisis. Considering these costs, identification of safe and effective methods to treat and prevent the occurrence of PASC represents an urgent, unmet public health need.

To address this need, the NIH has launched the RECOVER initiative across the nation (RECOVER: Researching COVID to Enhance Recovery) to better understand the disease. The RECOVER Initiative brings together patients, caregivers, clinicians, community leaders, and scientists from across the nation to understand, prevent, and treat PASC. The RECOVER Consortium represents and supports researchers who are leading studies on PASC at more than 200 sites around the country. These studies have a diverse group of participants, including adults, pregnant people, and children. Data from the RECOVER initiative, as well as existing literature, have highlighted 3 predominant symptom clusters, including exercise intolerance with post-exertional malaise (PEM), cognitive dysfunction, and autonomic dysfunction, which are frequently reported and of substantial importance to patients. In addition, data have revealed substantial heterogeneity in symptomology and presentation, even among those within a specific symptom cluster, and in many cases the lack of concordance between objective findings and reported symptoms.[6,7]

Emerging research is also beginning to identify risk factors for PASC. Women are disproportionately impacted by PASC relative to men at an approximately 3:1 ratio, with this sex difference appearing to fade in older individuals 60 to 70 years of age.[8-10] Longitudinal, multiomic profiling of COVID patients has revealed several anticipating risk factors for PASC at the time of diagnosis, including type 2 diabetes, SARS-CoV-2 RNAemia, Epstein-Barr Virus viremia, and autoantibodies.[11]

Three etiologies have emerged as leading candidates for instigating the manifestations of PASC: disordered regulation of coagulation, immune dysregulation, and viral persistence/reactivation.[12,13] In particular, prior investigators have demonstrated in preliminary data that SARS-CoV-2 can persist throughout the body for up to 230 days after acute infection[14], and that viral RNA has been identified within the gut mucosa and plasma of the majority of patients with PASC, but not within those without PASC.[15] Direct targeting of viral persistence, coagulation, and immune dysregulation with therapeutic agents warrants further investigation.

Preliminary data suggest that antiviral treatment of those with lingering symptoms of COVID-19 beyond 4 weeks after acute infection can result in symptom improvement. In a cohort study of 29 adults with lingering symptoms ranging from fatigue to inappropriate tachycardia, and many with severely reduced functional status (48%) on a patient reported scale, 18 patients received antiviral therapy (lopinavir/ritonavir and hydroxychloroquine). A statistically significant improvement in functional status, defined as at least a 1-point improvement on the functional scale, was observed at some point over a 6-week period among those receiving treatment compared to those who did not. Half of the patients who received antiviral therapy had SARS-CoV-2 RNA detected at treatment initiation via RT-PCR in plasma; no SARS-CoV-2 was detected in the plasma of these 9 patients during the 1st week following treatment.[16] While these data are promising, the heterogeneity and novelty of PASC, discordance between reported symptoms and objective findings,[6,7,17] and lack of validated measures and endpoints specific to this disease process highlight the importance of studying antiviral and other therapies' effects in the context of well-controlled clinical trials to ensure that optimal and appropriate therapies are made rapidly available for patients. The proposed clinical trials will evaluate potential mechanisms underpinning PASC and PASC improvement and test the utility of endpoints and outcome measures that have been well-established in other disease processes in the PASC population. In addition, collection of key biospecimens can assist in the development of biomarkers associated with PASC symptom clusters and response to therapy allowing a better understanding of pathogenesis and potentially targeted interventions. Phase 3 appendix trials will be sized to build on learnings from phase 2 trials to more definitively define drug efficacy for interventions that show promise in preliminary evaluations.

2.3 RISK/BENEFIT ASSESSMENT

2.3.1 KNOWN POTENTIAL RISKS

Potential risks of this study include those associated with the specific study drug (refer to appendices for details), blood draws, nasal swabs, performance measures, and loss of confidentiality.

Risks associated with blood draws include momentary discomfort and/or bruising. Infection, excess bleeding, clotting, or fainting are also possible, although unlikely.

Risks associated with nasal swabs include mild irritation, insignificant local pain, and minor bleeding.

Risks associated with the incremental and endurance shuttle walk tests (ISWT/ESWT) include tiredness, shortness of breath, or palpitations during or after the test. The active stand test and full autonomic testing may cause some participants to faint or feel lightheaded or weak. The cardiopulmonary exercise

test may result in fatigue, shortness of breath, possible bronchospasm, cardiac arrhythmia, and/or syncope.

There is also a risk of loss of confidentiality. However, coding all participant data with a unique identification number will minimize risk to loss of participant confidentiality.

2.3.2 KNOWN POTENTIAL BENEFITS

Participants may benefit from the potential clearance of the viral infection or reduction in inflammation and improvement in PASC symptoms.

Society in general and future patients infected with SARS-CoV-2 may benefit from the study's results, which aim to provide a better understanding of the benefit/risk of antiviral or anti-inflammatory treatment for PASC and alleviation of PASC symptoms.

2.3.3 ASSESSMENT OF POTENTIAL RISKS AND BENEFITS

Potential benefits may outweigh potential risks. PASC is a significant health issue and can have consequences that impact quality of life. These consequences are extremely important for the individual and also impact the society at large.

3 OBJECTIVES AND ENDPOINTS

The objectives, outcome measures, and endpoints for the study are listed in Table 1, below. Further details on outcome measures and endpoints are provided in Section 10 and the Statistical Analysis Plan (SAP).

Table 1. Summary of study objectives, endpoints, and outcome measures.

OBJECTIVES	OUTCOME MEASURES	ENDPOINTS
Primary		
Evaluate the effect of intervention(s) versus control on symptom-specific participant-reported outcome measures within each Post-acute Sequelae of SARS-CoV-2 (PASC) symptom cluster at Day 90	**Cognitive dysfunction symptom cluster:** Patient-Reported Outcomes Measurement Information System (PROMIS) – cognitive 8a<br>**Autonomic dysfunction symptom cluster:** Orthostatic Hypotension Questionnaire (OHQ)<br>**Exercise intolerance symptom cluster:** Modified DePaul Symptom Questionnaire-Post Exertional Malaise (DSQ-PEM)	Primary endpoint:<br>Meeting pre-specified change from baseline to Day 90 on cluster-specific Participant Reported Outcome (PRO) (yes/no)<br>Exploratory endpoint:<br>PRO outcome as continuous variable at Day 90<br>See Section 10.5.1
Secondary		
Evaluate the effect of intervention(s) versus control on performance-based outcomes within each PASC symptom cluster	**Cognitive dysfunction symptom cluster:** neurocognitive battery<br>**Autonomic dysfunction symptom cluster:** active stand test<br>**Exercise intolerance symptom cluster:** endurance shuttle walk test (ESWT)	Secondary endpoint:<br>Meeting pre-specified change from baseline to Day 90 on cluster-specific performance outcome (yes/no)<br>Exploratory endpoint:<br>Performance outcome as continuous variable at Day 90<br>See Section 10.5.2
Safety		
Characterize the safety and tolerability of intervention(s) in Post-acute Sequelae of SARS-CoV-2 Infection (PASC) Symptom Questionnaire	• Serious adverse events (SAEs)<br>• Events of Special Interest (ESIs)<br>• Adverse events (AEs)<br>• Adherence in intervention versus control	• Occurrence of individual SAEs<br>• Occurrence of any 1 or more SAE(s)<br>• Occurrence of AEs and SAEs leading to discontinuation<br>• Occurrence of ESIs<br>• Number of missed doses (adherence)<br>See Section 10.5.3

OBJECTIVES	OUTCOME MEASURES	ENDPOINTS
Exploratory		
Investigate mechanisms of potential intervention(s) efficacy	• Antigenemia (plasma spike protein antigen and other markers of viral persistence and reactivation)<br>• Viral RNA in nasal swabs<br>• Blood inflammatory markers<br>• Coagulation markers<br>• Endogenous and exogenous metabolites	Change from baseline through end of study / final follow-up of the following:<br>• Viral clearance, persistence, and recurrence<br>• Immune dysfunction<br>• Coagulation<br>• Biologic system functioning<br>See Section 10.5.4
Evaluate intervention(s) effect on healthcare utilization	Healthcare utilization, including medical visits and hospitalizations	• Cumulative medical visits in intervention vs control<br>• Cumulative hospitalizations in intervention vs control<br>See Section 10.5.4
Describe the effect and durability of intervention(s) versus control across performance-based outcomes and participant reported outcomes (PROs) within each PASC symptom cluster	**All symptom clusters:**<br>• PASC Symptom Questionnaire<br>• PROMIS-29+2<br>• Patient global impression of change<br>**Cognitive dysfunction symptom cluster:**<br>• PROMIS - cognitive 8a<br>• Everyday Cognition 2 (ECOG 2)<br>**Autonomic dysfunction symptom cluster:**<br>• Composite Autonomic Symptom Score 31 Form (COMPASS 31)<br>• OHQ<br>• Full autonomic testing (select sites)<br>**Exercise intolerance symptom cluster:**<br>• DePaul Symptom Questionnaire Post Exertional Malaise (DSQ-PEM)<br>• Cardiopulmonary exercise test (select sites)	Outcome at Day 90 and End of Study<br>See Section 10.5.4

OBJECTIVES	OUTCOME MEASURES	ENDPOINTS
Determine biomarkers associated with PASC symptom clusters and intervention-associated recovery	Outcome measures may include:<br>• Active viral replication<br>• Cytokine levels<br>• Immune profiling (B cells, T cells, natural killer cells)<br>• Sanctuary site assessment<br>• Infecting SARS-CoV-2 variant, impact SARS-CoV-2 immune state at time of infection	Change in biomarker levels<br>See Section 10.5.4

4 STUDY DESIGN

4.1 OVERALL DESIGN

This multi-center, multi-arm, master platform clinical trial protocol describes the overall design for prospective, double-blind, randomized, controlled trials that are described in appendices evaluating treatment of PASC in adults previously infected with SARS-CoV-2. Each appendix describes a sub-study that will evaluate interventions for PASC. The interventions may include different dosing durations or drug combinations for phase 2 or 3 investigation. Phase 2 appendices are designed to understand mechanisms of potential efficacy, safety and tolerability, and preliminary efficacy. Phase 3 appendices build upon the knowledge gained in phase 2 to more definitively evaluate the efficacy of interventions that show promise in phase 2 trials. To decrease sample size, control participants may be pooled across appendices when appropriate. Rules for pooling data will be specified for each sub-study.

Within each sub-study, multiple symptom clusters are noted by up to 60% of individuals (unpublished data). Study intervention appendices may be added or removed according to adaptive design and/or emerging evidence; refer to the protocol appendices for further information on each intervention.

All participants will undergo blood draws to assess presence of virus and other biomarkers and report overall global health status and symptomatology as well as undergo organ-specific assessments, including PROs of functional status and objective assessments. Follow-up will be a combination of face-to-face visits when necessary to obtain objective clinical assessments and phone calls to be mindful of the participant burden.

4.2 SCIENTIFIC RATIONALE FOR STUDY DESIGN

Each intervention under this platform protocol will follow a double-blind, randomized trial design to compare 1 or more dose levels of the study intervention to control. Antiviral agents and other therapeutics of interest will be used. Additional interventions can be studied as new agents are available or as the understanding of the viral sanctuary sites and/or immune/inflammatory response is improved and favorable drug properties are discovered. Both phase 2 and phase 3 appendices will be incorporated into this platform to enable a more seamless transition from phase 2 to phase 3 trials for promising interventions by allowing phase 3 trials to take advantage of the phase 2 infrastructure for trial implementation. In some cases, incorporation of phase 2 and phase 3 appendices within a single protocol will also enable shared utilization of data across appendices.

Phase 2 and 3 trials conducted under this platform master protocol will share major design elements such as inclusion criteria, dose levels, follow-up schedule, and study outcomes. Because phase 3 designs are expected to be refined based on phase 2 experience, some implementation differences may occur, for example:

- **Inclusion criteria.** One or more symptom clusters may be dropped from phase 3 if phase 2 data suggest a lack of efficacy, insufficient volumes of eligible participants, or other factors that might impact a successful phase 3 outcome. Target populations may be narrowed to specific subgroups if exploratory phase 2 data reveal participant factors that strongly predict presence or absence of treatment benefit.
- **Dose levels.** In general, each phase 3 study is expected to focus on a single intervention dose level. Phase 2 exploratory analyses will inform the selection of an optimal dose for phase 3 evaluation.

- **Follow-up schedule.** The duration and frequency of participant follow-up may be modified in light of phase 2 data. Factors to be considered include participant burden, patterns of missing data and drop out, fluctuations in symptoms over time, and uncertainty regarding the durability of a hypothesized treatment benefit.
- **Study outcomes.** An outcome designated as secondary in the current protocol may be elevated to primary in phase 3 if phase 2 data suggest that it outperforms a current primary outcome as a meaningful and sensitive measure of treatment benefit.

4.3 JUSTIFICATION FOR DOSE

The dose and duration of the intervention(s) will be based on the specific agent. Refer to the appendices for details.

4.4 END OF STUDY DEFINITION

The End of Study will occur when all participants have completed their End of Study Visit.

5 STUDY POPULATION

5.1 INCLUSION CRITERIA

In order to be eligible to participate in this study, an individual must meet all of the following criteria:

1. ≥ 18 years of age at the time of enrollment
2. Previous suspected, probable, or confirmed SARS-CoV-2 infection, as defined by the Pan American Health Organization[18ɸ]

   ɸ Suspected and probable cases leading to PASC symptoms will only be allowed if they occurred before May 1, 2021, and will be limited to 10% of the study population. Otherwise, confirmed cases leading to PASC symptoms are required. Refer to the MOP for details.

   Suspected case of SARS-CoV-2 infection - Three options, A through C:

   a. *A person who meets the clinical OR epidemiological criteria. Clinical criteria: Acute onset of fever AND cough (influenza-like illness) OR Acute onset of ANY 3 OR MORE of the following signs or symptoms: fever, cough, general, weakness/fatigue, headache, myalgia, sore throat, coryza, dyspnea, nausea, diarrhea, anorexia. Epidemiological criteria: Contact of a probable or confirmed case or linked to a COVID-19 cluster; or*
   b. *Acute respiratory infection with history of fever or measured fever of ≥ 38°C; and cough; with onset within the last 10 days; and who requires hospitalization); or*
   c. *With no clinical signs or symptoms, NOR meeting epidemiologic criteria with a positive professional use or self-test SARS-CoV-2 antigen-Rapid Diagnostic Test.*

   Probable case of SARS-CoV-2 infection:

   a. *A person who meets clinical criteria above AND is a contact of a probable or confirmed case or is linked to a COVID-19 cluster.*

   Confirmed case of SARS-CoV-2 infection - Two options, A through B:

   a. *A person with a positive nucleic acid amplification test, regardless of clinical criteria OR epidemiological criteria; or*
   b. *Meeting clinical criteria AND/OR epidemiological criteria (See suspect case A). With a positive professional use or self-test SARS-CoV-2 Antigen-Rapid Diagnostic Test.*
3. At least 2 moderate symptoms from the same Symptom Cluster or 1 severe cluster-associated symptom identified via the Cluster Targeted COVID-19 Symptom Questions (CTCSQ), see Section 8.14.3, with participant identifying new symptoms since COVID-19 illness and having persisted for at least 12 weeks[19]
4. Meeting PRO Symptom Cluster criteria for 1 Symptom Cluster (See Section 8.14.4)
5. Willing and able to provide informed consent, complete the surveys, clinical assessments, and return for all of the necessary follow-up visits

5.2 EXCLUSION CRITERIA

An individual who meets any of the following criteria will be excluded from participation in this study. Refer to appendices for additional appendix-level criteria:

1. Known active acute SARS-CoV-2 infection ≤ 4 weeks from consent
2. Known severe anemia, defined as < 8 g/dL[20]
3. Meeting the following symptom cluster exclusion for eligible clusters[#]:
    a. Cognitive dysfunction: known stroke that resulted in cognitive impairment within 3 months of enrollment
    b. Autonomic dysfunction: atrial fibrillation or significant cardiac arrhythmia, more than moderate alcohol consumption[ɣ], pre-existing sustained severe hypertension (BP> 180/110 mmHg in the sitting position)
    c. Exercise intolerance: any of the following within 4 weeks of consent - an acute myocardial infarction or unstable angina, uncontrolled arrhythmias causing symptoms or hemodynamic compromise, acute myocarditis or pericarditis, uncontrolled acutely decompensated heart failure (acute pulmonary edema), acute pulmonary embolism, suspected dissecting aneurysm, severe hypoxemia at rest, any acute or chronic disorder that may affect exercise performance, or if they are aggravated by exercise (e.g., infection, thyrotoxicosis, unable to cooperate)

    [ɣ] *Defined as greater than 2 drinks a day for men and 1 drink a day for women. A drink is equivalent to 12 ounces of beer (5% alcohol content), 8 ounces of malt liquor (7% alcohol content), 5 ounces of wine (12% alcohol content), 1.5 ounces or a "shot" of 80-proof (40% alcohol content) distilled spirits or liquor (e.g., gin, rum, vodka, whiskey).* [21]

4. Known diagnosis of chronic Lyme disease with persistent symptoms, sequelae, or related therapy
5. Any non-marijuana illicit drug use within 30 days of informed consent
6. Current or recent use (within the last 14 days) of study intervention*
7. Known allergy/sensitivity or any hypersensitivity to components of the study intervention (s) or control*
8. Known contraindication(s) to study intervention(s),
9. Inability to discontinue symptomatic medications for the identified time periods (See Section 6.5.1)
10. Moderate or severe immunocompromised patients, such as those described in the NIH COVID-19 Treatment Guidelines (*https://www.covid19treatmentguidelines.nih.gov/special-populations/immunocompromised/*)
11. Currently enrolled in another clinical trial outside this platform protocol or another study intervention appendix in this platform protocol[$]
    [$]*Participants may enroll in another clinical trial or re-enroll in this platform protocol for a different study intervention appendix if they have completed a 30-day washout period. For those who wish to re-enroll, re-enrollment can only occur after efficacy (via the primary objective) has been determined for the appendix in which they were previously enrolled.*
12. Any condition that would make the participant, in the opinion of the investigator, unsuitable for the study

**If only 1 study intervention appendix is open at the time of enrollment. If multiple study intervention appendices are open, a participant may be excluded from any study intervention appendix based on contraindications listed in the study intervention appendix, current use of study intervention, or known allergy/sensitivity/hypersensitivity and still remain eligible for the remaining study intervention appendices.*

5.3 SPECIAL CONSIDERATIONS FOR PARTICIPANT ENROLLMENT

Participants will not be enrolled into a symptom cluster cohort for which they also meet symptom cluster specific exclusion criteria. Assessments for a symptom cluster will be performed among

participants who meet the symptom cluster-specific eligibility criteria. The Principal Investigator will decide in which cluster the participant will be enrolled based on severity of symptoms. The most severe cluster is based on the participant's PROs. Additionally, once the cluster is decided and documented in a source document, the study team will document the cluster in which the participant will be enrolled (the cluster in which they will participate) in RAVE EDC. NOTE: the study team will randomize the participant in IXRS only to that cluster. Refer to appendices for details.

5.4 SCREEN FAILURES

Screen failures are defined as participants who consent to participate in the clinical trial but are not subsequently randomly assigned to the study intervention or entered in the study. A minimal set of screen failure information is required to ensure transparent reporting of screen failure participants, to meet the Consolidated Standards of Reporting Trials (CONSORT) publishing requirements and to respond to queries from regulatory authorities. Minimal information includes demography, screen failure details, and eligibility criteria.

5.5 STUDY DEFINITION OF ENROLLMENT

For this study, enrollment is defined as signing consent and completing randomization.

5.6 STRATEGIES FOR RECRUITMENT AND RETENTION

The RECOVER Clinical Trial Data Coordinating Center (CT-DCC) will use an integrated strategy of coordinating with community organizations, the public, and clinical trial sites to identify and retain study participants. To ensure a diverse population is enrolled, strategies from prior successful initiatives will be refined and utilized. The study team will develop a comprehensive communication strategy involving print and social media, as well as leveraging existing organizational structures where possible, to educate the public on concerns about PASC and opportunities for clinical trial participation. Interested members of the public will be provided with information to contact a local site for potential participation.

Participants can be recruited and identified through participating site outreach. Site investigators, or their designee, may contact eligible participants to introduce the study and discuss study participation.

Participants may be recruited from other ongoing COVID-19 trials if they opted-in to be contacted about future research opportunities. Patient advocates that represent a diverse PASC community will be engaged in the study at every step. Patient advocates will serve as consultants to inform study design, protocol development, and recruitment and retention strategies.

During active study, study sites will maintain close connections with study participants.

6 STUDY INTERVENTION

6.1 STUDY INTERVENTION(S) ADMINISTRATION

6.1.1 STUDY INTERVENTION DESCRIPTION

See appendices.

6.1.2 DOSING AND ADMINISTRATION

See appendices.

6.2 PREPARATION/HANDLING/STORAGE/ACCOUNTABILITY

6.2.1 ACQUISITION AND ACCOUNTABILITY

Receipt and use of study drug will be handled, tracked by the sites, and stored in a safe, secure, and temperature-monitored location to which only the investigator and designated personnel have access. Use of study drug will be tracked by the sites. At minimum, the sites will check intervention adherence at the Drug Dosing Midpoint clinic visit (Visit 3/Day 15 -3 days, i.e., Day 12 to Day 15) to monitor compliance with study drug administration and retrain the participant, as necessary. Participants will also be asked to bring all unused drug with them to the clinic during their Midpoint visit and End of Dosing (EOD) clinic visit (Visit 4/Day 25 +3 days, i.e., Day 25 to Day 28).

See appendices for further details.

6.2.2 FORMULATION, APPEARANCE, PACKAGING, AND LABELING

See appendices.

6.2.3 PRODUCT STORAGE AND STABILITY

Upon receipt, study drug should be stored according to the instructions specified on the label. See appendices.

6.2.4 PREPARATION

See appendices.

6.3 MEASURES TO MINIMIZE BIAS: RANDOMIZATION AND BLINDING

To achieve blinding and an equitable randomization probability, a 2-step randomization process will be used.

At the first step, each participant will be assigned with equal probability to 1 of the appendices for which the participant is eligible after applying any appendix-level exclusion criteria. At the second step, if the appendix includes k dose levels of active intervention plus a control group, participants will be assigned with probability $1/(k+1)$ to 1 of the $(k+1)$ available treatment options.

For simplicity, the text below assumes that M_1 active appendices will have no sharing of control participants and that an additional M_2 active appendices each involve a single intervention dose level

and will use a single pooled control group among them. Let k_i be the number of dose levels in the i-th appendix where no restriction is placed on $k_1, \ldots, k_{M_1}$ and $k_{M_1+1} = k_{M_1+2} = \cdots = k_{M_1+M_2} = 1$. Randomization will proceed as follows for a participant who is eligible for m_1 of the M_1 appendices with no sharing of controls and m_2 of the M_2 appendices with sharing of controls. At the first stage, the participant will be assigned with probability $1/(m_1 + m_2)$ to 1 of the appendices for which the participant is eligible. If the randomly assigned intervention is unable to use a pooled control group, the second stage randomization will assign the participant with probability $1/(k_i + 1)$ to 1 of the available intervention groups, where i denotes the randomly assigned appendix. If the randomly assigned intervention is 1 that will use a pooled control group, the second stage randomization will assign the participant to active intervention or control at an m_2: 1 ratio, where m_2 is defined above.

Sites will be informed to which study intervention appendix the participant is randomized, but not whether they are allocated to 1 of the active intervention dose levels or control within that appendix. The participants and investigators will be blinded throughout the study.

6.3.1 UNBLINDING

For each appendix, the participant, treating clinicians, and study personnel will remain blinded to study intervention versus control assignment until after the database is locked and blinded analysis for that sub-study is completed. Blinding may continue beyond the final analysis if the control group data are being used by another ongoing sub-study. Only the biostatistical team who is preparing closed interim reports will be unblinded. Specifically, study intervention/control will be dispensed with packaging and labelling that blinds treatment assignment. Unblinding will occur only if required for participant safety or treatment, at the request of the treating clinician. Refer to the Manual of Procedures (MOP) for further details.

6.4 STUDY INTERVENTION ADHERENCE

Participants will be notified of the importance of completing the full treatment course.

6.5 CONCOMITANT THERAPY

For this protocol, a prescription medication is defined as a medication that can be prescribed only by a properly authorized/licensed clinician. Medications to be reported in the Case Report Form (CRF) are relevant concomitant prescription medications, over-the-counter medications, and supplements. Baseline concomitant medications will include all concomitant therapies taken by the participant within 14 days prior to informed consent. Relevant concomitant medications will be reviewed through the End of Study.

6.5.1 SYMPTOMATIC THERAPY

Drugs administered for symptomatic treatment and initiated prior to screening may be continued as long as there is no contraindication due to drug-drug interaction with the study intervention and there is no change in dose or administration during the study period. Administration of the following therapies for each symptom cluster will be carefully documented and closely monitored during the study period because of the potential for changes in these therapies to alter study results:

Cognitive dysfunction: prescribed or illicit stimulants, amantadine, N-methyl-D-aspartate receptor antagonists (e.g., memantine, dissociative drugs).

Autonomic dysfunction: ephedrine, midodrine, amphetamine/attention deficit hyperactivity disorder medications, tricyclic antidepressants, florinef, sympathomimetics, Adderall, methylphenidate, atomoxetine, or other serotonin and norepinephrine reuptake inhibitors.

Exercise intolerance: none

6.5.2 RESCUE MEDICINE

Participants who require a rescue medication to treat a non-study-related acute condition during the study period should proceed with treatment for the acute condition, as prescribed by their treating clinician. They may continue to receive study drug as long as the rescue medication is not listed as a contraindication in any of the active study appendices. If the rescue medication is contraindicated, the participant will discontinue the use of the study intervention, but will continue to be followed per the Schedule of Procedures (refer to appendices). These participants will be considered evaluable for the analysis.

7 PARTICIPANT DISCONTINUATION/WITHDRAWAL

7.1 PARTICIPANT DISCONTINUATION FROM STUDY DRUG

Discontinuation from study intervention does not mean discontinuation from the study, and remaining study procedures should be completed as indicated by the appendix protocol. If a clinically significant finding is identified after enrollment, such as changes from baseline, the investigator or qualified designee will determine if any change in participant management is needed.

An investigator may discontinue a participant from the study intervention at their discretion, for any reason including, but not limited to, 1 of the following:

- Significant study intervention non-compliance
- If any clinical AE, laboratory abnormality, or other medical condition or situation occurs such that continued participation in the study or intervention would not be in the best interest of the participant
- Confirmed new case of acute SARS-CoV-2

Participants who discontinue due to an AE will be followed until resolution of the AE. The reason for participant discontinuation from study intervention will be recorded on the CRF. Participants who are discontinued from the study intervention but not withdrawn from the study will continue to be followed for all study procedures. If a participant discontinues the study intervention, but does not withdraw consent, they will be followed for safety for at least 28 days after the final study intervention administration.

7.2 PARTICIPANT WITHDRAWAL FROM THE STUDY

Participants are free to withdraw from participation in the study at any time upon request. The study team will attempt to determine a reason for withdrawal; however, participants are not obligated to provide a reason for withdrawal. If obtained, the reason for withdrawal will be recorded on the CRF. No further study procedures will be performed and no further data will be collected from the participant following study withdrawal. All of the data collected up until the time of withdrawal will be maintained in the study database and will be used as the participant’s data are evaluable for analysis.

7.3 LOST TO FOLLOW-UP

Participants will be considered lost to follow-up if they fail to return for any scheduled visit *and* if they are unable to be contacted after multiple attempts and methods by the study site staff and/or a central search company.

The following actions must be taken if a participant fails to return to the clinic for a required study visit:

- The site will attempt to contact the participant and reschedule the missed visit and counsel the participant on the importance of maintaining the assigned visit schedule and ascertain if the participant wishes to and/or should continue in the study.
- Before a participant is deemed lost to follow-up, the investigator or designee will make every effort to regain contact with the participant or next of kin (where possible, telephone calls and,

if necessary, a certified letter to the participant's last known mailing address or local equivalent methods). These contact attempts should be documented in the participant's medical record or study file.

- Should the participant continue to be unreachable, after exhausting all methods, the participant will be considered to have withdrawn from the study with a primary reason of lost to follow-up.

7.4 STUDY HALTING RULES

Study intervention dosing and enrollment will be temporarily suspended pending review of the Data and Safety Monitoring Board (DSMB) based on appendix-specific criteria. The DSMB recommendations will be considered by the NIH and the study Principal Investigator prior to making any decisions regarding study continuation or discontinuation.

8 STUDY ASSESSMENTS AND PROCEDURES FOR PHASE 2 STUDY DESIGNS

8.1 SCHEDULE OF PROCEDURES

Refer to appendices

8.2 Screening (Day -14 To Day 0)

Information about the study will be presented to potential participants and questions will be asked to determine potential eligibility. Study-specific screening procedures will begin only after informed consent is obtained.

Screening procedures may be done over 1 or 2 calendar days. However, in many cases (e.g., autonomic dysfunction and cognitive dysfunction symptom clusters) all the screening assessments can be done in < 24 hours, with baseline assessments, specimen collection, and the initial study product administration occurring on the same calendar day.

After the informed consent, the following assessments will be performed at Screening to determine eligibility:

- Demographics
- Medical History; including SARS-CoV-2 vaccination status and dates, SARS-CoV-2 test result date (if available), signs and symptoms, and treatment (including hospitalization, Intensive Care Unit (ICU) status, supplemental O_2 status) and PASC history (symptoms and duration)
- Collection of concomitant therapies taken within 14 days of informed consent
- CTCSQ
- Select PRO Symptom Cluster Assessment(s) relevant to the primary objective (repeated only if need to retest for the autonomic cluster). Refer to Section 8.14.4 for Symptom Cluster Assessments:
    - **Cognitive dysfunction symptom cluster:** PROMIS – cognitive 8a, ECOG2
    - **Autonomic dysfunction symptom cluster:** OHQ, COMPASS-31
    - **Exercise intolerance symptom cluster:** DSQ-PEM
- Review appendix-level eligibility criteria, refer to appendices
- ISWT; for participants who are considered for the Exercise Intolerance Symptom Cluster, the ISWT must be completed at an in-person screening visit that occurs at least 3 days before the Baseline Visit. The DSQ-PEM must be completed remotely +1 to 2 days after the visit where ISWT is performed to assess PEM.
- Pregnancy test, for individuals capable of becoming pregnant, blood or urine
- Blood collection for the following evaluations:
    - Local: Comprehensive metabolic panel (if not done within the past 3 months); prothrombin Time (PT), International normalized ratio (INR), Activated Partial Thromboplastin Time (aPTT), high-sensitivity C-reactive protein (CRP), D-Dimer, platelets, and white blood cell count

    - Send out: Mechanistic evaluation and biorepository storage for future investigation
    - Local: Safety labs, per appendix
- Nasal Swab for:
    - SARS-CoV-2 rapid test (e.g. rapid antigen test [RAT] or nucleic acid amplification test [NAAT]), completed locally
    - SARS-CoV-2 polymerase chain reaction (PCR), completed at central lab
- Stool Collection for mechanistic evaluation, processed at central lab

8.3 BASELINE (VISIT 2, DAY -2 TO DAY 0)

Baseline assessments will preferably occur on the same day as the first intervention administration and may occur on the same day as Screening. In the event that drug dosing does not begin on the same day as the Baseline visit, intervention administration must begin within 48 hours of baseline assessments. The following will occur at the Baseline visit:

- Concomitant Medication review
- PROMIS-29+2
- PASC Symptom Questionnaire
- Performance Measure Symptom Cluster Assessment(s). Refer to Section 8.14.4 for Symptom Cluster Assessments
- DSQ-PEM completed remotely +1 to 2 days after the visit to assess PEM
- If not obtained at Screening, pregnancy test, for individuals capable of becoming pregnant, blood or urine
- If not obtained at Screening, Blood Collection:
    - Send out: Mechanistic evaluation and biorepository storage for future investigation
- If not obtained at Screening, Nasal Swab for:
    - SARS-CoV-2 rapid test (e.g. rapid antigen test [RAT] or nucleic acid amplification test [NAAT]), completed locally
    - SARS-CoV-2 polymerase chain reaction (PCR), completed at central lab
- If not obtained at Screening, Stool Collection for mechanistic evaluation, processed at central lab
- Exploratory endpoint testing at select sites (Cardiopulmonary Exercise Testing [CPET], Full Autonomic Testing, peripheral blood mononuclear cells [PBMCs])
- Height and Weight
- Blood Pressure (BP) and heart rate, supine and standing
- Randomization and Study Drug Distribution of Kit 1
- Safety assessment including SAEs and suspected unexpected serious adverse reactions (SUSARs), non-serious AE from the start of study drug administration for select appendices

8.4 DRUG DOSING MIDPOINT CLINIC VISIT (VISIT 3, DAY 12 TO DAY 15)

Refer to appendices for intervention-specific dosing duration.

PROs will be reported approximately weekly during the drug dosing period, through the end of the study as indicated below and in the Schedule of Events. Additionally, at approximately the midpoint of drug dosing, a Drug Dosing Midpoint Clinic Visit will occur (Visit 3, Day 12 to Day 15). At this visit, the following will occur:

- Return IP Kit 1
- Concomitant Medication review
- Study drug distribution of IP Kit 2
- PROMIS-29+2, approximately weekly remotely during drug dosing period
- PASC Symptom Questionnaire, approximately weekly remotely during drug dosing period
- PRO Symptom Cluster Assessment(s), approximately weekly remotely during drug dosing period. Refer to Section 8.14.4 for Symptom Cluster Assessments
- Blood Collection, for the following evaluations:
    - Local: PT, INR, aPTT, high-sensitivity CRP, D-Dimer, white blood cell count, and platelets
    - Send out: Mechanistic evaluation
    - Local: Safety labs, per appendix
- Nasal Swab for SARS-CoV-2 rapid test, completed locally (e.g. RAT or NAAT), and PCR, completed at central lab
- Exploratory endpoint testing at select sites (PBMCs only)
- Safety assessment including SAEs, SUSARs, and ESIs; non-serious AEs for select appendices
- Healthcare Utilization
- Drug Adherence

8.5 END OF DOSING CLINIC VISIT (VISIT 4, DAY 25 TO DAY 28)

The End of Dosing Clinic Visit (Visit 4) will occur after the final intervention dose has been administered. The following will occur at the End of Dosing Clinic Visit (Visit 4, Day 25 to Day 28):

- Return IP Kit 2
- Concomitant Medication review
- PROMIS-29+2
- PASC Symptom Questionnaire
- CTCSQ
- PRO Symptom Cluster Assessment(s). Refer to Section 8.14.4 for Symptom Cluster Assessments
- Performance Measure Symptom Cluster Assessment(s). Refer to Section 8.14.4 for Symptom Cluster Assessments. The DSQ-PEM must be completed remotely +1 to 2 days after the visit to assess PEM.
- Blood Collection, for the following evaluations:
    - Local: PT, INR, aPTT, high-sensitivity CRP, D-Dimer, platelets, and white blood cell count
    - Send out: Mechanistic evaluation and biorepository storage for future study
    - Local: Safety labs, per appendix

- Nasal Swab for SARS-CoV-2 rapid test, completed locally (e.g. RAT or NAAT), and PCR, completed at central lab
- Exploratory endpoint testing at select sites (CPET, Full Autonomic Testing, PBMCs)
- Blood Pressure (BP) and heart rate, supine and standing
- Safety assessment including SAEs, SUSARs, and ESIs; non-serious AEs for select appendices
- Healthcare Utilization
- Drug Adherence evaluation based on doses returned at this visit

8.6 FOLLOW-UP (END OF DOSING [DAY 25 TO DAY 28] THROUGH DAY 179)

The Follow-Up Period will begin after the End of Dosing Clinic Visit (Visit 4, Day 25 to Day 28). A follow-up clinic visit will occur for all participants on Day 90 (± 5 days), Visit 7. All assessments required for the Day 90 visit will occur at the Day 90 (± 5 days) in-person visit (Visit 7); any assessments completed remotely during the week of the Day 90 visit cannot replace the in-person Day 90 assessments. All participants will receive remote follow-up approximately weekly with optional in-person visits for blood collection on Day 45 ± 5 days (Visit 5), Day 60 ± 5 days (Visit 6), and Day 120 ± 5 days (Visit 8). All assessments that are completed remotely on a weekly basis will be open to the participant to complete for 3 days. Any assessments that are not completed within that window will expire and will not be collected. The following assessments will occur as part of follow-up.

8.7 DAY 45 OPTIONAL FOLLOW-UP (VISIT 5, VISIT 45 [DAY 40 TO DAY 50])

- Send out: Mechanistic evaluation and biorepository storage for future study
- Send out: Nasal swab for PCR

8.8 DAY 60 OPTIONAL FOLLOW-UP (VISIT 6, DAY 60 [DAY 55 TO DAY 65])

- Send out: Mechanistic evaluation and biorepository storage for future study
- Send out: Nasal swab for PCR

8.9 DAY 90 (VISIT 7, DAY 90 [DAY 85 TO DAY 95])

- Concomitant Medication review
- PROMIS-29+2
- PASC Symptom Questionnaire
- CTCSQ
- PRO Symptom Cluster Assessment(s). Refer to Section 8.14.4 for Symptom Cluster Assessments
- Performance Measure Symptom Cluster Assessment(s). Refer to Section 8.14.4 for Symptom Cluster Assessments. The DSQ-PEM must be completed remotely +1 to 2 days after the visit to assess PEM.
- Blood Collection, for the following evaluations:
    - Local; PT, INR, aPTT, high-sensitivity CRP, D-Dimer, platelets, and white blood count

    - Send out: Mechanistic evaluation and biorepository storage for future study
    - Local: Safety lab tests, per appendix
- Nasal swab for SARS-CoV-2 rapid test, completed locally (e.g., RAT or NAAT)
- Nasal swab for PCR, completed at central laboratory
- Blood pressure (BP) and heart rate, supine and standing
- Exploratory endpoint testing at select sites (CPET, Full Autonomic Testing, PBMCs)
- Healthcare Utilization
- Safety assessment including SAEs, SUSARs, and ESIs

8.10 DAY 120 OPTIONAL FOLLOW-UP (VISIT 8, DAY 120 [DAY 115 TO DAY 125])

- Send out: Mechanistic evaluation and biorepository storage for future study
- Send out: Nasal swab for PCR

8.11 END OF STUDY VISIT (VISIT 9, DAY 183 ± 7 DAYS)

The End of Study Visit (Visit 9) will be a remote visit. The following will occur at the End of Study Visit:

- Concomitant Medication review
- PROMIS-29+2
- PASC Symptom Questionnaire
- CTCSQ
- PRO Symptom Cluster Assessment(s) based on participant cluster assignment. Refer to Section 8.14.4 for Symptom Cluster Assessments
- Healthcare Utilization
- Safety assessment including SAEs, SUSARs, and ESIs

8.12 OPTIONAL BLOOD COLLECTION/NASAL SWAB VISITS

Participants will be asked to attend optional Follow-up Visits (Visits 5, 6, and 8) that include blood collection and nasal swabs for PCR at Day 45 ± 5 days (Visit 5), Day 60 ± 5 days (Visit 6), and Day 120 ± 5 days (Visit 8). Participants opting to attend these optional visits will be compensated for their time and effort.

8.13 CLINICAL LABORATORY ASSESSMENTS

A comprehensive metabolic panel that includes the following will be completed at local labs according to the Schedule of Procedures:

- Sodium
- Potassium
- Chloride
- Carbon Dioxide/Bicarbonate

- Blood Urea Nitrogen
- Creatinine
- Calcium
- Glucose
- Albumin
- Total Bilirubin
- Estimated Glomerular Filtration Rate
- Total protein
- Alanine Aminotransferase (ALT)
- Alkaline Phosphatase
- Aspartate Aminotransferase (AST)

The following from the complete blood count will be completed at local labs according to the Schedule of Procedures:

- Platelets
- White Blood Cell Count

The following coagulation and other labs (D-Dimer, high-sensitivity CRP) will be collected from local labs according to the Schedule of Procedures:

- PT
- INR
- aPTT
- D-Dimer
- High-sensitivity CRP

For the baseline clinical laboratory assessments, if a participant has comprehensive metabolic panel available within 3 months of study enrollment, they do not need to be repeated as part of the study. Additional clinical laboratory assessments may be required per appendix, refer to appendices.

The SARS-CoV-2 rapid test (e.g. RAT or NAAT) will also be completed locally according to the timepoints indicated in the Schedule of Procedures.

8.14 STUDY ASSESSMENTS

8.14.1 BIOSPECIMEN COLLECTION

Because a primary focus of this protocol will be to investigate mechanisms underlying PASC and improvement with antivirals and other therapies, select biomarkers will be evaluated over time among those who receive intervention or control. These biomarkers are targeted towards the leading hypotheses for PASC pathogenesis, including viral persistence/reactivation, immune dysregulation, and dysregulation of coagulation and will be obtained from blood (~80 mL), stool, and nasal swabs.

Biomarkers will be collected at various time points that will enable the understanding of potential improvement with therapy and rebound, and will be collected at similar time points as PROs and other assessments in order to understand the relationship between the biomarkers and clinical findings.

At minimum, the following biomarkers will be assessed:

- Antigenemia (SARS-CoV-2 antigens), including the S1 subunit of spike, full length spike, and nucleocapsid in plasma

While some important biomarkers will be known at the time of participant enrollment and assays will be available, others may not. In addition to biomarkers, peripheral blood mononuclear cells will be collected at select sites at 4 timepoints: Baseline, drug dosing Midpoint Clinic Visit, End of Dosing Clinical Visit, and Day 90 Visit. Samples collected throughout the course of this study will be stored at the RECOVER Biorepository, which is designed to collect and store biospecimens for future research related to the various studies of the RECOVER Program. Samples from biorepositories have proven to be enormously important in the last 20 years, as information on the components of blood has expanded rapidly. Important insights have been gained from biorepository samples from clinical trials and the stored samples from the RECOVER Program will prove equally productive and important. This biorepository will be conducted under the coordination of the Duke Clinical Research Institute (DCRI) which serves as the CT - DCC for all RECOVER clinical trials.

Samples will be collected for storage at -80°C and will be transported and stored at the Biorepository at the Mayo Clinic in Rochester, MN for up to 7 years.

8.14.2 PROMIS-29+2 (PROPr)

Patient-Reported Outcomes Measurement Information System (PROMIS-29) global health scale: The PROMIS was developed out of the "Roadmap for Medical Research" created by the NIH in 2002 as valid, generalizable items to standardize clinical research across NIH-funded research dealing with PROs. Multiple PROMIS scales have been validated across many clinical populations.[22] The PROMIS-29 consists of 29 items that assess general domains of health and functioning, including overall physical health, mental health, social health, pain, fatigue, and overall perceived quality of life. The PROMIS global health scales have been correlated against the EuroQol 5 dimensions questionnaire (EQ-5D). [23] Additionally, PROMIS scales have been used with PASC patients.[24]

The PROMIS-29+2 is used to calculate a preference score (PROPr score) by the addition of 2 Cognitive Function Ability items. Preference-based scores provide an overall summary of HRQOL on a common metric. Preference-based scores summarize multiple domains on a metric ranging from 0 (as bad as dead) to 1 (perfect or ideal health). Scores can be used in comparisons across groups and for cost-utility analyses. [25]

8.14.3 CLUSTER TARGETED COVID-19 SYMPTOM QUESTIONS (CTCSQ)

The CTCSQ is a set of thirteen questions with the first twelve items rated none, mild, moderate, or severe. The final item is a global symptom question (participant global impression of change). Participants must report having at least 2 moderate symptom(s) from the same Symptom Cluster or 1 severe symptom in order to meet eligibility criteria. If a participant reports a moderate or severe item

#1-3, they will complete the exercise intolerance and autonomic dysfunction cluster assessments at Baseline. If a participant reports a moderate or severe item #1-8, they will complete the autonomic dysfunction cluster assessments at Baseline. If a participant reports a moderate or severe item #9-12, they will complete the cognitive dysfunction cluster assessments at Baseline. Item #13 is not associated with specific symptom clusters and will not have any influence on symptom cluster assignment. After cluster assignment, the CTCSQ will not result in any alterations to cluster assignment, but will be used as a symptom tracking tool.

Table 2. CTCSQ items #1-13 and associated symptom cluster(s)

Please rate the level of severity you experienced of the following symptoms in the last 7 days:	None	Mild	Moderate	Severe	Associated Symptom Cluster
1. Fatigue (being tired)					Exercise Intolerance/ Autonomic Dysfunction
2. Post-exertional malaise (symptoms worse after even minor physical or mental effort)					
3. Weakness in your arms and/or legs					
4. Pain in your limbs					Autonomic Dysfunction
5. Faintness (light-headedness)					
6. Gastrointestinal upset					
7. Color changes of the skin					
8. Dry mouth					
9. Problems with thinking or concentrating (brain fog)					Cognitive Dysfunction
10. Confusion					
11. Forgetfulness					
12. Difficulty focusing					
GLOBAL QUESTION (Participant Global Impression of Change)	Improved a lot	Improved somewhat	Stayed about the same	Worsened somewhat	Worsened a lot
13. Compared to the start of the study, the overall severity of your Long COVID symptoms have-					

Abbreviations: CTCSQ=Cluster Targeted Covid-19 Symptom Questions; N/A = Not applicable.

8.14.4 SYMPTOM CLUSTER ASSESSMENT

As part of screening, potential participants will answer the CTCSQ. Eligible participants will complete Symptom Cluster assessments based on their CTCSQ responses (see Table 2) and will subsequently be assigned to 1 of the 3 Symptom Clusters based on the screening assessments. Participants must meet

criteria for the PRO within a specific symptom cluster in order to be included in the cluster, see *italicized* text within each cluster for inclusion reference points. After study enrollment and initial cluster assignment, further assessments will be performed among participants who meet the symptom cluster criteria. Refer to Figure 3.

- Cognitive dysfunction:
  - PROMIS – cognitive 8a[26] (PRO assessment). Part of the PROMIS test battery, the cognitive function is a quantitative measure of current cognitive function. *A PROMIS – cognitive 8a function T-score < 40 at Screening will include a participant in this cluster and trigger other baseline assessments listed below*.
  - ECOG 2 (PRO assessment). The Everyday Cognition 2 is a self-report, 41-item questionnaire used to measure the participant's perceived capacity to perform activities related to cognitive function, which could impact major activities of daily living and independence. It has been used for patients with mild cognitive impairment, Alzheimer's Disease, and dementia. It takes approximately 5 minutes to complete.
  - Neurocognitive battery (performance measure) to assess various elements related to cognition. The neurocognitive battery consists of a cognitive assessment sequence of the following: WHO/UCLA Auditory Verbal Learning Test (WHO/UCLA AVLT) and Symbol Digit Modalities Test. The entire battery takes approximately 30 minutes to complete.
- Autonomic dysfunction:
  - COMPASS 31[27] (PRO assessment). COMPASS 31 is a measure of autonomic symptoms across multiple domains commonly seen in patients with PASC. *A COMPASS 31 score ≥ 25 in combination with OHQ requirements at Screening will include a participant in this cluster.*
  - Orthostatic Hypotension Questionnaire (OHQ) (PRO assessment). The OHQ is a measure of orthostatic intolerance, which has been the primary presentation of patients with PASC-related autonomic dysfunction. This measure includes the 6-item symptom assessment (OHSA) and the 4-item Daily Activity Scale (OHDAS). Each item is scored from 0 (none/no interference) to 10 (worst possible interference), describing the preceding week. Prior data in patients with PASC suggests that this measure well-discriminates those with symptoms compared to healthy controls. This assessment will be carried out twice (once at Screening and once at Baseline) in at least the first 100 participants to assess test-retest reliability. *A value ≥ 4 on question 1 of OHQ in combination with COMPASS 31 requirements at Screening will include a participant in this cluster.*
  - Active stand test (performance measure). The active stand test is a well-characterized measure of short-term neurologic and cardiovascular function that requires limited resources and aids in diagnosis of patient symptoms of orthostatic intolerance.
  - Full autonomic testing (exploratory performance measure) consists of 2 catecholamine collections, respiratory sinus arrhythmia, hyperventilation, Valsalva maneuver, and a head up tilt test and will be completed at select sites. The entire battery takes approximately 90 minutes to complete.

- Exercise intolerance:

  - The Modified DePaul Symptom Questionnaire Post Exertional Malaise (DSQ-PEM) PRO assessment.[28] Some patients have experienced debilitating PEM that may occur following exertion or on the day after exertion. To assess PEM, the DSQ-PEM short form will be adapted. This scale assesses symptom frequency and severity over a 6-month look back period; however, for the purposes of this study, it will be modified to assess over a 1-week look back period. It was previously validated in patients with myalgic encephalomyelitis/chronic fatigue syndrome. Frequency is rated on a 5-point Likert scale: 0 = none of the time, 1 = a little of the time, 2 = about half the time, 3 = most of the time, and 4 = all of the time. Severity is also rated on a 5-point Likert scale: 0 = symptom not present, 1 = mild, 2 = moderate, 3 = severe, 4 = very severe. Post-Exertional Malaise (PEM) will be deemed to be present for participants reporting at least 1 moderate (rated severity ≥ 2) PEM symptom for a frequency rated ≥ 2. *At least 1 of the symptoms listed in questions 1-5 with a score ≥ 2 on both severity and frequency on the DSQ-PEM at Screening will include a participant in this cluster and trigger other baseline assessments listed below.*

  - Endurance shuttle walk test (performance measure). The ESWT consists of timed walking on a 10 meter course.[29] The result is expressed as total walking time after an initial 2-minute warm-up. In order to determine walking speed, the baseline ESWT will be performed in combination with the ISWT.[30] Walking speed for the ISWT will increase every minute until the participant is too breathless or fatigued to continue walking at the required speed. The ESWT will then be performed as 85% of the maximum walking speed achieved on the ISWT.[29]

  - Cardiopulmonary exercise testing (exploratory performance measure) will be completed at select sites. This is an exercise test that measures exercise abilities. The test involves a 2 to 3-minute warm-up, followed by 8 to 12 minutes of progressively harder work on a treadmill or stationary bike. The goal is to exercise until the participant is no longer able to continue. The final outcome for this measure will be the maximum rate of oxygen consumption (VO2 max).

- All Clusters

  - The DSQ-PEM will be performed remotely approximately 1-day (+1 day) after assessment of Performance Measures. For the purposes of assessing PEM related to the specific activity, it will be modified to assess PEM related specifically to the activity that occurred the previous day (since last visit [SLV]).

Each assessment will occur according to the Schedule of Procedures (refer to appendices). Procedures may be completed during the study as part of regular standard of clinical care. Training to standardize these assessments will be provided to participating sites by the RECOVER Clinical Trial – Data Coordinating Center (CT-DCC) and protocol study teams.

8.14.5 POST-ACUTE SEQUELAE OF SARS-COV-2 INFECTION (PASC) SYMPTOM QUESTIONNAIRE

Participants will be asked to complete a questionnaire that asks about the presence of PASC symptoms. This questionnaire includes additional symptoms that are not directly related to the Symptom Clusters in this study.

9 SAFETY ASSESSMENTS AND REPORTING

9.1 ADVERSE EVENTS AND SERIOUS ADVERSE EVENTS

9.1.1 DEFINITION OF ADVERSE EVENTS (AE) AND SERIOUS ADVERSE EVENTS (SAE)

An AE is any untoward medical occurrence in humans, whether or not considered drug-related, which occurs during the conduct of a clinical trial. An AE can therefore be any change in clinical status, routine labs, x-rays, physical examinations, etc., that is considered clinically significant by the study investigator.

An SAE or serious suspected adverse reaction (SAR) or serious adverse reaction, as determined by the investigator or the sponsor, is an AE that results in any of the following serious outcomes:

- Death
- Life-threatening AE ("life-threatening" means that the study participant was, in the opinion of the investigator or sponsor, at immediate risk of death from the reaction as it occurred and required immediate intervention)
- Persistent or significant incapacity or substantial disruption of the ability to conduct normal life functions
- Inpatient hospitalization or prolongation of existing hospitalization
- Congenital abnormality or birth defect
- Important medical event that may not result in 1 of the above outcomes, but may jeopardize the health of the study participant or require medical or surgical intervention to prevent 1 of the outcomes listed in the above definition of serious event

Hospitalization for elective treatment of a preexisting condition that did not worsen from baseline does not meet the definition of an SAE. Hospitalization is defined as a stay in the hospital exceeding 24 hours.

An unexpected AE is defined as any AE, the specificity or severity of which is not consistent with the study drugs' package insert or investigators brochure.

An unanticipated problem involving risk to participants or non-participants, including an environmental exposure and exposure to a breastfeeding infant, will be reported to the sponsor and the site IRB and central IRB, as appropriate. The event occurring in the non-participant will not be entered into the electronic data capture (EDC) system. Refer to the MOP and/or Safety Management Plan (SMP) for details.

Medication errors with the study drug(s) resulting in an SAE are reportable. The medication error will be captured as a protocol deviation and the SAE captured on the SAE electronic case report form (eCRF).

9.1.2 COLLECTION PERIOD FOR AE AND SAE INFORMATION

Safety event collection will occur at the pre-specified study visits but all participants will be instructed to self-report concerns by calling the site.

Serious adverse events (SAEs) or ESIs (per appendix) will be extracted by site personnel from the participant's medical record if the participant seeks medical care or if hospitalization occurs, each of which notifies the site to conduct follow-up.

Medical occurrences that begin before the first invasive study procedure (blood collection or nasal swab), but after obtaining informed consent, will not be considered an AE. The medical occurrence or condition will be captured on the Medical History eCRF.

Non-serious AEs that result in study drug discontinuation will be identified as the reason for study drug discontinuation within the study database, reported as an AE and collected from the start of study drug administration through the end of study drug dosing. Each study drug appendix will identify whether additional non-serious AEs require collection and reporting such as spontaneously reported non-serious AEs by the participant at clinic visits that occur during the dosing period, but do not result in study drug discontinuation. There is no expectation nor plan by the site nor the DCC to reconcile AEs reported by the participant with the PRO survey.

Serious adverse events (SAEs) will be collected from the first invasive study procedure (blood collection or nasal swab) through the End of Study Clinic Visit (Day 183 ± 7 days).

Adverse events (AEs) that qualify as an ESI, even if a non-serious AE, will be collected from the start of study drug administration through the End of Study Clinic Visit (Day 183 ± 7 days).

9.1.2.1 SEVERITY OF EVENT

For reportable events, the following guidelines will be used to describe severity grades, described in the Common Terminology Criteria for Adverse Events (CTCAE) v5.0, published November 27, 2017.

Grade refers to the severity of the AE. The CTCAE displays Grades 1 through 5 with unique clinical descriptions of severity for each AE based on this general guideline:

- Grade 1 Mild; asymptomatic or mild symptoms; clinical or diagnostic observations only; intervention not indicated.
- Grade 2 Moderate; minimal, local or noninvasive intervention indicated; limiting age-appropriate instrumental activities of daily living.
- Grade 3 Severe or medically significant but not immediately life-threatening; hospitalization or prolongation of hospitalization indicated; disabling; limiting self-care activities of daily living
- Grade 4 Life-threatening consequences; urgent intervention indicated.
- Grade 5 Death related to AE.

9.1.2.2 RELATIONSHIP TO STUDY INTERVENTION

All reportable events must have their relationship to the study intervention assessed by the clinician who examines and evaluates the participant based on temporal relationship and his or her clinical

judgment. The degree of certainty about causality will be graded using the categories below. In a clinical trial, the study product must always be suspect.

- **Related** – The AE is known to occur with the study intervention, there is a reasonable possibility that the study intervention caused the AE, or there is a temporal relationship between the study intervention and event. Reasonable possibility means that there is evidence to suggest a causal relationship between the study intervention and the AE
- **Not Related** – There is not a reasonable possibility that the administration of the study intervention caused the event, there is no temporal relationship between the study intervention and event onset, or an alternate etiology has been established

9.1.3 TIME PERIOD AND FREQUENCY FOR EVENT ASSESSMENT AND FOLLOW-UP

The occurrence of an AE, events of special interest (ESI), or SAE may come to the attention of study personnel during study visits and interviews of a study participant presenting for medical care, or upon review by a study monitor.

All reportable events will be captured on the appropriate CRF. Information to be collected includes event description, date/time of onset, clinician's assessment of severity, relationship to study product (assessed only by those with the training and authority to make a diagnosis), action taken with study drug (e.g. study drug discontinuation), and date/time of resolution/stabilization of the event. All events occurring within the pre-specified reportable time windows must be documented appropriately regardless of relationship.

Any medical condition that is present at the time the participant is screened will be considered as baseline and not reported as a safety event. However, if the participant's condition deteriorates at any time during the study, it will be recorded as a safety event.

Changes in the severity of a safety event will be documented to allow an assessment of the duration of the event at each level of severity to be performed. Safety events characterized as intermittent require documentation of onset and duration of each episode.

The investigator will follow all SAEs until resolution, stabilization, or the event is otherwise explained. The DCRI Safety Surveillance Team will follow all SAEs until resolution, stabilization, or until otherwise explained.

9.1.4 REPORTING AND MONITORING OF SAES

All of the study drugs used in this platform protocol will be under an Investigational New Drug (IND) application and subject to IND regulations in 21 CFR 312 since their investigational use for treatment of PASC is not an approved indication. The IND sponsor, DSMB, or Study Medical Monitor will review aggregate safety data. The IND sponsor will be responsible for determining if the safety reporting criteria are met per 21 CFR 312.32(c)(1)(i)(C) and 21 CFR 312.32(c)(1)(iv) and will notify the CT-DCC to prepare an aggregate report for submission to the FDA. An aggregate safety report will be submitted to FDA as soon as possible, but in no case later than 15 calendar days after the IND sponsor determination. If the IND sponsor determines that an unexpected fatal or life-threatening SAR occurs markedly more frequently in a study drug arm than in the control arm, an aggregate safety report will be submitted to the FDA as soon as possible, but in no case later than 7 calendar days after the IND sponsor determination. Information on individual SAEs will be available upon request from the FDA following the submission of any aggregate reports.

Individual SAEs must be entered into the data system within 24 hours of site awareness. The DCRI Safety Surveillance team will notify pharmaceutical partners of SAEs within 1 to 2 business days of their receipt that occur involving the specific appendix of the supplied study drug/control, as required. Serious Adverse Events that are related and confirmed unlisted by the DCRI Safety Medical Monitor and IND sponsor will be reported to the FDA as suspected unexpected adverse reaction (SUSAR); as 7-day reports for unexpected fatal or life-threatening adverse reactions and 15-day reports for serious and unexpected adverse reactions. The SUSAR reports will be shared with the pharmaceutical partner of the supplied study drug according to the same timelines. If the IND sponsor, DSMB, or FDA note a clinically important increase in the rate of a SUSAR, the IND sponsor or designee will notify investigators no later than 15 calendar days after determining that the information qualifies for reporting. The investigators will notify their local IRB according to local guidelines if applicable. The CT-DCC will notify the central IRB. Refer to the Safety Management Plan for details regarding specific reporting timelines.

Investigators are not obligated to actively seek information on AEs or SAEs after the participant has concluded study participation. However, if the investigator learns of any SAE, including a death, at any time after a participant has completed the study (but before the study itself has ended), and they consider the event to be reasonably related to the study intervention, the investigator must promptly report the SAE to the sponsor via EDC entry.

9.1.5 EVENTS OF SPECIAL INTEREST (ESI)

Each study drug may have a unique list of ESIs. Refer to the relevant Appendix.

9.1.6 REPORTING OF PREGNANCY

Pregnancies of the participant or the participant's partner that occur following the participant's first study drug administration while on-study will be collected. If included in the study drug appendix that pregnant participants are excluded, the participant will be advised to discontinue study drug(s). Pregnancies will be followed until pregnancy outcome unless the participant refuses to provide consent. Pregnant participants will be asked to complete a pregnancy-specific consent form if the final outcome of the pregnancy is not reached while the participant is on-study. Pregnant partners of study participants will be asked to complete a consent form to follow pregnancy outcomes. Any pregnancy-associated ESI or SAE should be reported if information can be collected and entered into the CRF. The DCRI Safety Surveillance team will notify study drug supplying partners of a pregnancy within 1 to 2 business days after learning about the pregnancy, as required.

10 STATISTICAL CONSIDERATIONS FOR PHASE 2 DESIGN

10.1 STATISTICAL HYPOTHESES

Primary Endpoint:

- Improvement in symptom cluster-specific PRO measures, including the DSQ-PEM, PROMIS-cognitive 8a, or OHQ at Day 90 compared to baseline in intervention(s) vs. control

For each pairwise comparison between an intervention and control group, the primary null hypothesis to be tested is that the probability of meeting a specified degree of improvement in symptom cluster-specific primary outcome measures (including the DSQ-PEM, PROMIS-cognitive 8a, or OHQ) at Day 90 compared to baseline is the same for intervention and control. The alternative hypothesis is that the probability of meeting a specified degree of improvement in symptom cluster-specific primary outcome measures at Day 90 compared to baseline differs for intervention and control. This hypothesis will be tested separately for each symptom cluster. If π_D denotes the probability of improvement if randomized to intervention and π_C denotes probability of improvement if randomized to control, then the primary null and alternative hypotheses may be expressed as follows:

$$H_0: \pi_D - \pi_C = 0 \quad \text{versus} \quad H_1: \pi_D - \pi_C \neq 0.$$

Secondary:

- Improvement in performance-based outcomes, including the ESWT, active stand test, and/or neurocognitive battery in intervention(s) vs. control
- intervention(s) are safe in the PASC population

The null hypothesis to be tested is that there is no difference between intervention and control group.

Exploratory:

- Improvement in viral, immune, and other mechanistic biomarkers over the course of the study in intervention(s) vs. control

The null hypothesis to be tested is that there is no difference between intervention and control group.

10.2 PRIMARY ESTIMAND

A separate pairwise comparison between intervention and control will be performed for each combination of study appendix, dose level, and symptom cluster. For each of these, the primary estimand will be the intention-to-treat between-group difference in marginal probabilities of improvement, defined as

$$\Delta_{\text{ITT}} = P(Y = 1|A = 1) - P(Y = 1|A = 0),$$

where Y denotes the primary outcome (1 = improvement, 0 = no improvement) and A denotes randomization assignment (1 = active treatment, 0 = control). This quantity will be estimated without regard to adherence and will implicitly average over observed patterns of concomitant therapies. In the unlikely event that follow-up data collection is prevented by death or hospitalization, the participant will be classified as not improving. The trial's SAP will provide further clarification of Δ_{ITT} and may specify additional secondary and supplementary estimands, for example, the hypothetical ideal between-group difference that would be observed in a setting of high treatment adherence.

10.3 SAMPLE SIZE DETERMINATION

This study uses an adaptive platform trial design that will allow study interventions to be added or dropped from consideration based on accruing evidence of futility or efficacy. In such a design, the required sample size depends on both the number of interventions tested and the ability to pool their control arms for analysis. Initial sample size estimates are based on a single appendix with a 1:1:1 allocation to each of 2 dose levels of an active intervention and control. If additional study interventions are added later that can contribute to pooled control, the sample size will be adjusted accordingly.

The sample size is based on a design that will evaluate treatment effects separately within 3 co-primary symptom cluster populations (cognitive dysfunction, autonomic dysfunction, and exercise intolerance) with a Holm-type adjustment to control the overall type-I error across them (see Section 10.5.1 for details). The Holm procedure will initially allocate alpha equally across symptom clusters (0.0166, 0.0166, 0.0166), with a total type-I error probability of 0.05. If the null hypothesis is initially rejected for some but not all symptom clusters, the Holm procedure will allow the non-rejected hypotheses to be re-tested at an updated, less stringent significance level.

Accounting for the Holm procedure's stepwise alpha selection in power calculations is complicated because the final significance threshold is partly random and depends on the unknown configuration of treatment effects across all 3 symptom clusters considered jointly. As a simple conservative approximation, power was calculated for each symptom cluster individually assuming a specified treatment effect for that single symptom cluster and assuming 0.0166 for the Holm procedure's final significance threshold.

Analysis within each symptom cluster population will control the type-I error across multiple dose levels by analyzing them in a pre-specified hierarchical testing procedure in order from highest to lowest dose. For simplicity, the total sample size was obtained by calculating sample size for a simple 2-group comparison and multiplying by 3/2.

Sample size was chosen to provide 80% power to detect a difference of 25 percentage points in the proportion of participants who experience the binary primary outcome for each pairwise comparison between a treatment group and control within each symptom cluster, assuming a 2-sided test with alpha = 0.0166. Specifically, a sample size of 82 per group yields 80% power assuming the true outcome probabilities are 37.5% in the control group and 62.5% in the active treatment group. This sample size was inflated by 1/0.82 = 1.22 to account for potential 18% dropout/loss to follow-up.

The final required sample size per symptom cluster was calculated as 82 x 1.22 = 100 participants per intervention group (300 total per symptom cluster). Enrollment within a symptom cluster will continue until the required sample size of 100 participants is met for each intervention group.

Table 3 illustrates the magnitude of treatment effects that will be detectable with 80% or 90% power under the study's planned sample size if the primary outcome occurs with probabilities ranging from 0.05 to 0.35 in the placebo group.

Table 3. Minimum treatment effects for 80% or 90% power

Probability of Improvement with Placebo	Minimum Treatment Effect Difference for 80% Power*	Minimum Treatment Effect Difference for 90% Power*
0.05	0.17 (0.22 vs 0.05)	0.20 (0.25 vs 0.05)
0.10	0.20 (0.30 vs 0.10)	0.23 (0.33 vs 0.10)
0.15	0.22 (0.37 vs 0.15)	0.25 (0.40 vs 0.15)
0.20	0.23 (0.43 vs 0.20)	0.27 (0.47 vs 0.20)
0.25	0.24 (0.49 vs 0.25)	0.28 (0.53 vs 0.25)
0.30	0.25 (0.55 vs 0.30)	0.28 (0.58 vs 0.30)
0.35	0.25 (0.60 vs 0.35)	0.28 (0.63 vs 0.35)

* Power for pairwise comparison between single dose level of active intervention versus control assuming 100 participants per treatment group (82 participants per treatment group with non-missing outcome data) and 2-sided alpha = 0.0166.

10.4 POPULATIONS FOR ANALYSES

Modified Intent-to-Treat (mITT) Population

The primary analysis of all study data will be based on a modified intent-to-treat population (mITT), consisting of all randomized participants who receive at least part of one dose of the study intervention or control. Participants will be analyzed according to their randomly assigned intervention group. A separate mITT population will be defined for each pairwise comparison between an active intervention and control within each symptom cluster population.

Participants who were enrolled into > 1 symptom cluster, as allowed under Protocol Version 3.0, will be included in the mITT population of each cluster for which inclusion criteria are met.

Safety Population

The Safety Population will consist of all randomized participants who receive at least part of 1 dose of the study intervention or control. Participants who are randomized and treated despite being ineligible for all 3 symptom clusters will be included.

10.5 STATISTICAL ANALYSES

This section describes analysis methods for primary, secondary, and exploratory efficacy outcomes. Unless otherwise specified, efficacy analyses will be based on the mITT population. Full details will be provided in the SAP.

Because each sub-study is designed to address a separate set of questions pertaining to distinct agents, no multiplicity adjustments will be made across sub-study appendices. Plans for controlling type-I error within each sub-study are described below.

For each appendix, a separate analysis of the primary outcome will be performed in parallel for each of 3 primary symptom cluster populations. A Holm-type multiplicity adjustment will be used to control the type-I error probability at 0.05 for analysis of primary outcomes across the 3 symptom clusters. If there are multiple dose levels of an intervention, analysis of primary outcomes will be performed sequentially in order from highest to lowest dose. At each dose level, if the comparison between active intervention versus control is significant for all 3 symptom clusters, then testing of primary outcomes will proceed to the next lower dose level. Otherwise, testing will stop and primary outcome comparisons at lower dose

levels will be regarded as exploratory. Comparisons between active intervention dose levels will be performed last and will be regarded as exploratory.

Figure 4 illustrates the protocol's multiple testing strategy for an appendix with 2 active dose levels. Hypotheses are grouped into a family $\mathcal{F}_1 = \{H_{11}, H_{12}, H_{13}\}$ for the higher dose level and a family $\mathcal{F}_2 = \{H_{21}, H_{22}, H_{23}\}$ for the lower dose level. Here, H_{dc} refers to the primary outcome analysis for symptom cluster c at dose level d. Testing begins with $\mathcal{F}_1$ and initially assigns local significance level α/3 to each member of $\mathcal{F}_1$. After rejecting a hypothesis within $\mathcal{F}_1$, its local alpha is reallocated equally within $\mathcal{F}_1$. In the event that all hypotheses within $\mathcal{F}_1$ are rejected, testing proceeds to $\mathcal{F}_2$, with alpha reallocated equally among its component hypotheses. In general, an appendix with $D \geq 1$ dose levels will test families $\mathcal{F}_1, \mathcal{F}_2, \mathcal{F}_D$ in order from highest to lowest dose and will proceed to testing $\mathcal{F}_{d+1}$ if and only if all hypotheses within $\mathcal{F}_d$ have been rejected.

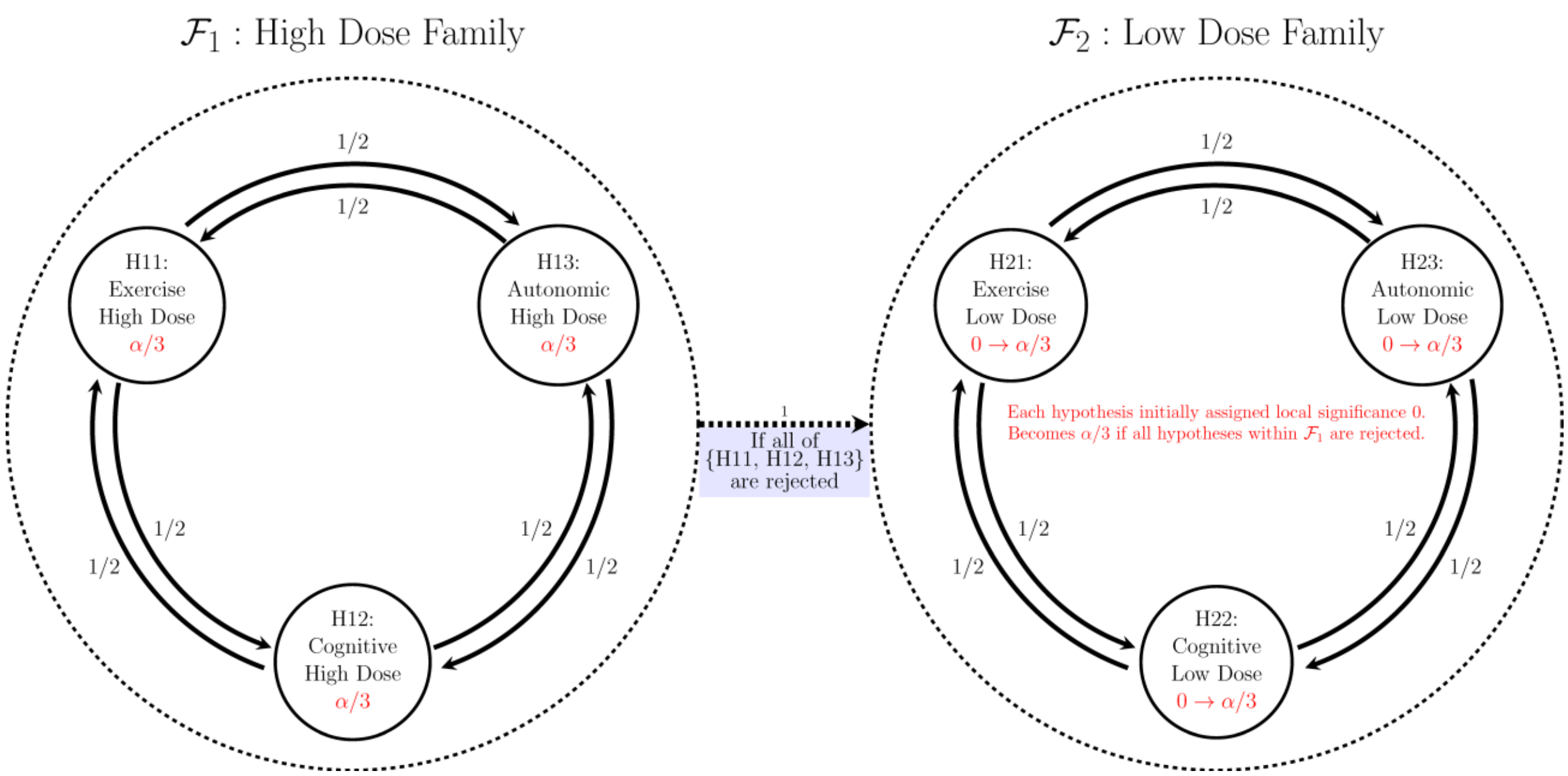

Figure 4. Multiple testing flowchart

The multiplicity procedure described above provides strong family-wise type-I error control across 3 symptom clusters and D dose levels for the primary outcomes. For secondary outcomes, the multiple testing strategy is less conservative. For each symptom cluster and dose level, if the hypothesis pertaining to the primary outcome is rejected, then the symptom cluster's major secondary outcome will be tested at the same significance level as the primary outcome. This does not strictly control type-I error across all primary and secondary outcomes, but does offer some protection by using the primary outcome as a gatekeeper for secondary outcomes within each symptom cluster. This less conservative approach is motivated by viewing the study design as a separate trial for each symptom cluster population. Of note, a finding of benefit in a particular symptom cluster population will only pertain to that symptom cluster population and will not be extrapolated to other symptom cluster populations.

10.5.1 PRIMARY EFFICACY ANALYSIS

Each of the 3 Symptom Cluster-specific primary endpoints is binary.

- For cognitive dysfunction, the primary endpoint is improvement of at least 5 T-score points on the PROMIS-cognitive 8a at Day 90 compared to baseline. To reduce variability, an average of

3 weekly assessments closest to Day 90 will be used. The proposed difference of 5 T-score points is derived from a body of literature that has specifically evaluated the minimally important difference using distribution- and anchor-based approaches across a number of disease processes (e.g., cancer, stroke, low back pain, osteoarthritis) and PROMIS measures (e.g., pain intensity, depression, physical functioning, anxiety, fatigue, etc.). These varied populations consistently report a minimally important difference of 2 to 6.[31-33] Moreover, a value of 5 represents one-half the standard deviation for PROMIS measures in the US general population and is recommended as a starting point when no empirical data are available.[34,35]

- For autonomic dysfunction, the primary endpoint is improvement as defined by a ≥ 1-point decrease in the OHQ question 1 at Day 90 compared to baseline. To reduce variability, an average of 3 weekly assessments closest to Day 90 will be used. At least 1-point change on question 1 has been determined to be clinically meaningful and resulted in previous updates to the FDA drug label for orthostatic hypotension associated with Parkinson's disease.
- For exercise intolerance, the primary endpoint is improvement in PEM, defined as having no symptoms of moderate or greater severity with 50% or more frequency as determined by the DSQ-PEM short form at Day 90.

A separate pairwise comparison between intervention and control will be performed for each combination of study appendix, dose level, and symptom cluster. Analysis will target the intention-to-treat between-group difference in marginal probabilities of improvement, defined as

$$\Delta_{\mathrm{ITT}} = P(Y = 1|A = 1) - P(Y = 1|A = 0),$$

where Y denotes the primary outcome (1 = improvement, 0 = no improvement) and A denotes randomization assignment (1 = active treatment, 0 = control).

To improve precision, the estimate of Δ_{ITT} may be adjusted for a small number of pre-specified baseline covariates using the method of Ge et al. 2011 or similar (see SAP for details).[36] Covariates will be selected based on having a suspected strong association with primary outcomes and high likelihood of complete data capture. Inference for Δ_{ITT} will be based on Wald-type test statistics and associated confidence intervals.

Significance levels for the primary analysis will be determined by the multiplicity procedure described in above Section 10.5. Briefly, alpha will initially be equally allocated across symptom clusters (0.0166, 0.0166, 0.0166), with a total type-I error probability of 0.05. If the primary outcome comparison exceeds the critical threshold for some but not all symptom clusters, the procedure will allow non-significant comparisons to be re-tested using an updated, less stringent critical threshold.

The primary endpoints for the cognitive and autonomic symptom clusters were created by dichotomizing an underlying quantitative PRO outcome. An exploratory analysis of these outcomes will also be performed using the underlying quantitative measurements. While exploratory, these continuous outcomes may be estimated with greater precision compared to binary outcomes and will provide important context for the overall results interpretation. Methods for exploratory endpoints are described below.

10.5.2 ANALYSIS OF SECONDARY ENDPOINTS

Secondary outcomes are binary and will be analyzed using methods identical to the primary outcomes.

<u>Secondary endpoint</u>:
Meeting pre-specified change from baseline to Day 90 on cluster-specific performance outcome (yes/no).

Secondary cluster-specific performance outcomes:
Cognitive dysfunction symptom cluster: neurocognitive battery
Autonomic dysfunction symptom cluster: active stand test
Exercise intolerance symptom cluster: endurance shuttle walk test (ESWT)

10.5.3 ANALYSIS OF SAFETY ENDPOINTS

Safety endpoints include the proportion of participants who experience individual SAEs and the proportion who experience any 1 or more SAEs. Each of these will be compared pairwise between each active intervention dose level and control within the appendix-specific Safety Population. Events of Special Interest (ESIs) will be summarized by study drug appendix and duration. Incidence of AEs/SAEs leading to discontinuation of study intervention will also be summarized.

10.5.4 ANALYSIS OF EXPLORATORY ENDPOINTS

Exploratory endpoints that are binary will be analyzed using methods identical to the primary outcomes.

Exploratory endpoints that are continuous will be analyzed via ANCOVA/regression with adjustment for pre-specified baseline covariates and with treatment effects expressed as differences in means. Covariates in each model will include an indicator of intervention group, the baseline value corresponding to the secondary outcome (e.g., adjust for baseline ESWT when analyzing End of Study ESWT as secondary outcome), and other pre-specified baseline factors.

10.5.5 EXPLORATORY LONGITUDINAL ANALYSES

Active intervention group comparisons of binary and continuous secondary outcomes will be assessed at multiple time points simultaneously by fitting longitudinal logistic (binary outcomes) and linear (continuous outcomes) regression models. Either hierarchical modeling with participant-specific random effects or Generalized Estimating Equations (GEE) methodology will be used to account for statistical dependence between repeated outcomes measured on the same participant over time.

Exploratory analysis will include analysis of symptom burden, PROs, exploratory performance measures at select sites, and healthcare utilization. Biomarkers will be explored to investigate mechanisms of potential intervention efficacy. Symptom burden analysis will be presented with consideration to subgroups by baseline symptoms, racial and ethnic subgroup, sex, acute COVID-19 severity, and timing after acute infection. Refer to the SAP for details.

10.5.6 EXPLORATORY BAYESIAN ANALYSES

To enhance interpretation, a subset of primary, secondary, and exploratory outcomes described above will be re-analyzed in a Bayesian statistical framework. The Bayesian statistical paradigm is an attractive framework for exploratory analyses because it provides a direct quantification of the likelihood of various hypotheses that are relevant when considering whether and how to design a subsequent phase 3 evaluation. Traditional frequentist analyses assess evidence indirectly by positing a null hypothesis and calculating the probability of observing data at least as extreme as what was observed if the null hypothesis was true. Such an approach is indirect because it doesn't directly quantify the likelihood that a hypothesis is true or the likelihood that the trial will be successful. In contrast, Bayesian analysis can answer relevant questions exactly as they are posed. In particular, it can calculate the likelihood that a treatment is sufficiently effective to merit studying in a confirmatory phase 3 study. The output of a Bayesian analysis is a posterior probability distribution describing the relative likelihood of different numerical estimates of unknown quantities. This distribution can be used to determine the post-trial probability of a clinically important treatment effect in light of the study data. An additional advantage

of Bayesian analysis is the ability to incorporate external information in the form of a prior distribution. For example, the prior may be formulated to reflect information from other RECOVER and non-RECOVER trials.

10.5.7 ANALYSIS OF TREATMENT EFFECT HETEROGENEITY

In addition to estimating the overall difference in outcome between intervention groups, exploratory analyses will assess whether and how the treatment effect differs according to a set of prospectively defined participant baseline factors, most importantly, vaccination status, duration of PASC symptoms, and selected virologic and/or inflammatory biomarkers (e.g., positive assay for antigenemia at baseline for antiviral agents). These analyses will be performed 1-at-a-time for each subgroup variable using logistic regression for binary outcomes and linear regression for continuous outcomes. A treatment-by-covariate interaction test will be used to assess whether variation in the estimated treatment effect across levels of the covariate is consistent with chance.

As a further exploratory analysis, a Bayesian multivariable modeling strategy will be used to estimate the treatment effect for selected outcomes as a function of a small number of prospectively defined baseline covariates (e.g., duration of treatment, selected biomarkers). The analysis will improve on conventional subgroup analyses by analyzing multiple subgroup factors jointly in a single multivariable model and providing treatment effect estimates tailored to a specific combination of multiple relevant covariates. The analysis will also permit estimating treatment effects as a function of dose level in sub-studies investigating multiple intervention dose levels. The Bayesian prior distribution will place low prior probability on the hypothesis of extreme treatment effect variation across subgroups (i.e., a shrinkage prior) and will thereby reduce the probability of obtaining highly noisy and extreme estimates of treatment effect heterogeneity. Before developing these models, Monte Carlo simulations based on blinded RECOVER data will be used to shed light on the maximum feasible number of model covariates and will reduce the number of candidate covariates ahead of time if simulation results suggest a high potential for noisy estimates or false discoveries.

10.5.8 MISSING DATA

A particularly strong effort will be made to prevent participant’s loss to follow-up and missing outcome data. To the extent that missing outcomes occur, a detailed evaluation of missing data patterns by intervention group will be conducted. Baseline characteristics will be compared for participants with and without missing outcome data. Analysis plans for primary and secondary endpoints will account for missing data and will be designed to minimize bias. If assessment of a primary or secondary endpoint is prevented by death or hospitalization, the participant will be classified as not improving. Sensitivity analyses will be performed under a range of missing data assumptions and may include multiple imputation, weighting, and likelihood-based methods. See SAP for details.

10.5.9 PLANNED INTERIM ANALYSIS

Interim examination of clinical endpoints and key safety events will be performed at regular intervals during the course of each sub-study/appendix. An independent, NIH-appointed, DSMB will monitor participant safety and review participant enrollment and performance of the trial. The primary objective of these interim analyses will be to ensure the safety of the participants enrolled in the trial and evaluate the accumulating endpoint data by treatment group. In addition, interim monitoring will involve a review of participant recruitment, compliance with the study protocol, status of data collection, and other factors which reflect the overall progress and integrity of the study.

There are no planned early stopping rules for efficacy in this protocol. Because PASC presentations and outcomes are highly varied, an important study objective is to estimate the effect of treatment on a

wide range of participant-relevant outcomes overall and within key pre-specified subgroups. If the study were to be stopped early with less than the full sample size, it would decrease precision for pre-specified group analyses and reduce the study's ability to characterize treatment risks and benefits based on important secondary effectiveness and safety outcomes. It would also limit the collection of data that are critical for planning future trials in similar patient populations.

Early stopping for rules for futility may be incorporated on a sub-study/appendix specific basis. Such rules will be documented in the sub-study appendices.

11 SUPPORTING DOCUMENTATION AND OPERATIONAL CONSIDERATIONS

11.1 REGULATORY, ETHICAL, AND STUDY OVERSIGHT CONSIDERATIONS

11.1.1 INFORMED CONSENT PROCESS

11.1.1.1 INSTITUTIONAL REVIEW BOARD (IRB)

The protocol, informed consent form(s) (ICF[s]), recruitment materials, and all participant materials will be submitted to the IRB(s) of record for review and approval. This approval must be obtained before any participant is enrolled. Any amendment to the protocol will require review and approval by the IRB(s) before being implemented in the study. All changes to the consent form will also be IRB-approved and a determination will be made regarding whether previously consented participants need to be re-consented.

11.1.1.2 CONSENT PROCEDURES AND DOCUMENTATION

All consenting will occur either via an electronic consent process or a paper process. Consent forms describing in detail the study intervention/control, study procedures, and risks will be given to the participant and documentation of informed consent is required prior to starting study procedures. Informed consent is a process that is initiated prior to the individual's agreement to participate in the study and continues throughout the individual's study participation. A description of risks and possible benefits of participation will be provided to the participants. Consent forms will be IRB-approved and the participant will be asked to read and review the document. The participant will be provided contact information in the event they have questions about study participation. This will allow them to communicate with the investigators (or their delegate), for further explanation of the research study and to answer any questions that may arise, as necessary. Participants will have the opportunity to carefully review the consent form and ask questions prior to signing.

The participants should have the opportunity to discuss the study and think about it prior to agreeing to participate. The participant will sign the informed consent document prior to any procedures being done specifically for the study. The participants may withdraw consent at any time throughout the course of the study. A copy of the informed consent document will be provided to the participants for their records. The rights and welfare of the participants will be protected by emphasizing to them that the quality of their medical care will not be adversely affected if they decline to participate in this study.

The study team will distinguish between the desire to discontinue study intervention(s) and the desire to withdraw consent for study follow-up.

11.1.2 STUDY DISCONTINUATION AND CLOSURE

This study may be temporarily suspended or prematurely terminated if there is sufficient reasonable cause. Written notification, documenting the reason for study suspension or termination, will be provided by the sponsor to study participants, site investigators, the central IRB, and the US FDA. Study participants will be contacted, as applicable, and be informed of changes to study visit schedule.

Circumstances that may warrant termination or suspension include, but are not limited to:

- Determination of unexpected, significant, or unacceptable risk to participants
- Demonstration of efficacy that would warrant stopping
- Insufficient compliance to protocol requirements
- Data that are not sufficiently complete and/or evaluable
- Determination that the primary endpoint has been met
- Determination of futility

Study may resume once concerns about safety, protocol compliance, and data quality are addressed, and satisfy the sponsor, IRB and/or FDA.

11.1.3 CONFIDENTIALITY AND PRIVACY

Participant confidentiality is strictly held in trust by the participating investigators, their staff, and the sponsor(s) and their agents. This confidentiality is extended to cover testing of biological samples in addition to the clinical and private information relating to participants. Therefore, the study protocol, documentation, data, and all other information generated will be held in strict confidence. No information concerning the study or the data will be released to any unauthorized third party without prior written approval of the sponsor. The study participant's contact information will be securely stored in the clinical study database.

Study participant research data, which is for purposes of statistical analysis and scientific reporting, will be transmitted to and stored at the CT-DCC. The study data entry and study management systems used by clinical sites and by research staff will be secured and password protected. At the end of the study, all study-related data storage systems will be archived according to local processes.

11.1.4 KEY ROLES AND STUDY GOVERNANCE

The RECOVER program is overseen by the RECOVER Executive Committee, the NIH RECOVER Program, and the RECOVER Senior Oversight Committee Co-Chairs. The RECOVER program also includes a Clinical Trial Steering Committee, which is a multi-stakeholder committee that oversees the study and includes patients, the CT-DCC, the NIH, the FDA, and academic and subject matter experts.

The CT-DCC is overseen by a Principal Investigator. The CT-DCC is responsible for study coordination, site management, communication, financial administration, treatment allocations, receipt and processing of data, quality control programs, and statistical analysis and reporting.

The DSMB will oversee the safety and welfare of trial participants as well as provide recommendations for continuation, discontinuation, or revision of the trial.

11.1.5 DATA AND SAFETY MONITORING BOARD (DSMB)

Safety oversight will be under the direction of the RECOVER DSMB composed of individuals with the appropriate expertise. Members of the DSMB will be independent of the study conduct and free of conflict of interest, or measures should be in place to minimize perceived conflict of interest. The DSMB will meet at least semi-annually to approve protocols, assess safety and efficacy data, and at appropriate intervals to meet requirements for the Interim Analyses on each arm of the study. The DMSB will operate under the rules of an approved charter that will be written and reviewed at the organizational meeting of the DSMB. At this time, each data element that the DSMB needs to assess will be clearly defined. The DSMB will provide its input to the NIH.

11.1.6 CLINICAL MONITORING

This study will employ a centralized risk-based approach to monitoring with routine and periodic review of site-submitted data to review the informed consent process, select eligibility criteria, medical history, identify and follow-up on missing data, inconsistent data, data outliers, etc. and ensure completion of administrative and regulatory processes. The study team will facilitate regular communication through training sessions, teleconferences, videoconferencing, email, etc. Using quality-by-design principles, steps will be taken at the study design stage to foresee and limit significant problems that might occur during the study conduct. Follow-up from the sites is expected to keep participants engaged.

11.1.7 QUALITY ASSURANCE AND QUALITY CONTROL

The study team will work in tandem to ensure that the data collected in this study are as complete and correct as possible. A 4-step, multi-functional approach to quality control will be implemented:

- Training: Prior to the start of enrollment, the clinician investigators and key study personnel at each site will be trained with the clinical protocol and data collection procedures, including how to use the EDC system. Follow-up training and training for new study personnel or new versions of the protocol will be conducted as needed.
- Monitoring: The RECOVER CT-DCC will ensure that data collection is handled properly, will provide in-service training, and will address questions from site investigators and coordinators. Electronic review of data quality and completeness will occur on a regular and ongoing basis. Any issues will be addressed.
- Managing data: After the data have been transferred for statistical summarization, data description, and data analysis, further crosschecking of the data will be performed with discrepant observations being flagged and appropriately resolved through a data query system.
- Reviewing data: Data regarding events of interest will be reviewed to ensure appropriate documents are collected for DSMB review. The CT-DCC will monitor study data and contact site study teams when events comprising the primary endpoint are not complete.

11.1.8 DATA HANDLING AND RECORD KEEPING

11.1.8.1 DATA COLLECTION AND MANAGEMENT RESPONSIBILITIES

Streamlining research activities and conducting the trial in a pragmatic manner will increase the ability to complete the trial in the face of strained clinical and research resources. Data may be collected by electronic methods, supplemented by telephone or videophone follow-up, from the electronic health record, or may be collected by paper diary.

Data will be collected directly from participants using email with a survey link, an electronic application, or a phone call. These processes are Health Insurance Portability and Accountability Act (HIPAA) compliant.

Site personnel or participants will enter study data into a secure online database. Data will be maintained in a secure online database until the time of study publication. At the time of publication, the CT-DCC will generate a de-identified version of the database for archiving. All source documents at the sites should be completed in a neat, legible manner to ensure accurate interpretation of data.

11.1.8.2 STUDY RECORDS RETENTION

Study documents should be retained for a minimum of 6 years after the study has ended. However, if required by local regulations or the US FDA, these documents should be retained for a longer period. No records will be destroyed without the written consent of the sponsor. It is the responsibility of the sponsor to inform the investigator when these documents no longer need to be retained.

11.1.9 PROTOCOL DEVIATIONS

A protocol deviation is defined as non-compliance with the clinical study protocol or GCP requirements. The non-compliance may be on the part of the participant, site investigator, or the site staff.

A major protocol deviation is a significant divergence from the protocol that may have significant effect on the participant's safety, rights, or welfare and/or on the integrity of the study data. Major protocol deviations must be sent to the study IRB and local IRB per their guidelines, recorded in source documents, and reported to the coordinating center. All protocol deviations will be documented. For this study, any missed or delayed survey completion will not be considered a major protocol deviation, unless it is a study procedure that is required for the primary endpoint.

11.1.10 PUBLICATION AND DATA SHARING POLICY

This study will comply with the NIH Public Access Policy, which ensures that the public has access to the published results of NIH funded research. It requires scientists to submit final peer-reviewed journal manuscripts that arise from NIH funds to the digital archive PubMed Central upon acceptance for publication.

11.1.11 CONFLICT OF INTEREST POLICY

The independence of this study from any actual or perceived influence, such as by the pharmaceutical industry, is critical. Therefore, any actual conflict of interest of persons who have a role in the design, conduct, analysis, publication, or any aspect of this trial will be disclosed and managed. Furthermore, persons who have a perceived conflict of interest will be required to have such conflicts managed in a way that is appropriate to their participation in the design and conduct of this trial. The study leadership in conjunction with the NIH has established policies and procedures for all study group members to disclose all conflicts of interest and will establish a mechanism for the management of all reported dualities of interest.

11.2 ABBREVIATIONS

AE	Adverse Event
ALT	Alanine Transaminase
ANCOVA	Analysis of Covariance
aPTT	Activated Partial Thromboplastin Time
AST	Aspartate Aminotransferase
BP	Blood Pressure
CFR	Code of Federal Regulations
COMPASS 31	Composite Autonomic Symptom Score 31 (form)
CONSORT	Consolidated Standards of Reporting Trials
COVID-19	Coronavirus Disease of 2019
CPET	Cardiopulmonary Exercise Testing
CRF	Case Report Form
CRP	C-reactive Protein
CTCAE	Common Terminology Criteria for Adverse Events
CTCSQ	Cluster Targeted COVID-19 Symptom Questions
CT-DCC	Clinical Trial – Data Coordinating Center
DCRI	Duke Clinical Research Institute
DILI	Drug-Induced Liver Injury
DSMB	Data Safety Monitoring Board
DSQ - PEM	DePaul Symptom Questionnaire Post Exertional Malaise
DUA	Data Use Agreement
ECOG 2	Everyday Cognition 2
eCRF	Electronic Case Report Forms
EOD	End of Dosing
ESI	Events of Special Interest
ESWT	Endurance Shuttle Walk Test
EUA	Emergency Use Authorization
FDA	Food and Drug Administration
GCP	Good Clinical Practice
HIPAA	Health Insurance Portability and Accountability Act
HIV	Human Immunodeficiency Virus
HRQOL	Health-Related Quality of Life
ICF	Informed Consent Form
ICH	International Conference on Harmonization
IND	Investigational New Drug Application
INR	International Normalized Ratio
IP	Investigational Product
IRB	Institutional Review Board
ISWT	Incremental Shuttle Walk Test
LFT	Liver Function Test
mITT	Modified Intention-To-Treat
MOP	Manual of Procedures
NAAT	Nucleic Acid Amplification Test
NCT	National Clinical Trial
NIH	National Institutes of Health
OHQ	Orthostatic Hypotension Questionnaire
OHDAS	Orthostatic Hypotension Daily Activity Scale
OHSA	Orthostatic Hypotension Symptom Assessment

PASC	Post-acute Sequelae of SARS-CoV-2 Infection
PBMC	Peripheral blood mononuclear cell
PCR	Polymerase Chain Reaction
PEM	Post-Exertional Malaise
PHI	Personal Health Information
PRO	Patient/Participant Reported Outcome
PT	Prothrombin Time
RAT	Rapid Antigen Test
RECOVER	Researching COVID to Enhance Recovery
SAE	Serious Adverse Event
SAP	Statistical Analysis Plan
SAR	Suspected Adverse Reaction
SARS-CoV-2	Severe Acute Respiratory Syndrome Coronavirus 2
SLV	Since last visit
SUSAR	Suspected Unexpected Serious Adverse Reaction
T3	Triiodothyronine
T4	Thyroxine
TSH	Thyroid Stimulating Hormone
ULN	Upper Limit of Normal
US	United States
VO2 max	Maximum Rate of Oxygen Consumption

11.3 PROTOCOL AMENDMENT HISTORY

Version	Date	Description of Change
1.0	10OCT2022	None, original protocol
2.0	09MAR2023	• Updated study description, background, and rationale;<br>• Moved biomarker objective to exploratory, added an additional biomarker exploratory objective (exploratory objective #6), and added more details around exploratory biomarker outcome measures;<br>• Adjusted sample size from 1700 to 900;<br>• Added reference to and description of potential phase 2 and phase 3 study design within the appendices;<br>• Extended follow-up to 6 months;<br>• Study Schema updated to align with study design changes;<br>• Symptom cluster assignment flow updated;<br>• Limits were placed on the number of participants with suspected and probable COVID-19 on or after May 1, 2021;<br>• Two moderate symptoms from the same Symptom Cluster or 1 severe symptom required for inclusion;<br>• End of Study has been extended to 183 days from the original 90 days;<br>• PROMIS 29+2, PASC Symptom Questionnaire, and PRO Symptom Cluster Assessment(s) will be done weekly as opposed to every 2 weeks;<br>• Stool collection added;<br>• Updated clinical laboratory assessments;<br>• Optional blood collection/nasal swab visits added to Days 45, 60, and 120; details on biospecimen collection added;<br>• Added ECOG 2 to the cognitive dysfunction PRO assessment;<br>• Non-serious AEs that result in study drug discontinuation will now be reposted separately as an AE;<br>• Statistical considerations updated to align with study design changes;<br>• PAXLOVID dosing modified to 15 days of active DOI (nirmatrelvir/ritonavir) with 10 days of control (ritonavir), 25 days of active DOI, or 25 days of control (1:1:1);<br>• Dose-related exploratory objectives have been added to Appendix A (PAXLOVID Phase 2);<br>• Thyroid function added as an Event of Special Interest to Appendix A (PAXLOVID Phase 2); and<br>• Other administrative changes throughout.
3.0	18MAY2023	• Updated schema to align with changes to study procedures;<br>• Clarified study endpoints, referred to stats section for details;<br>• Updated exploratory objectives;

Version	Date	Description of Change
		• Added the collection of non-serious AEs to the protocol, non-serious AEs will be assessed from start of study drug administration to the EOD for select appendices;<br>• Participants will have 3 days to complete remote weekly assessments;<br>• DSQ-PEM completed after performance measures will occur at 1 (+ 1 day) after performance measure;<br>• Updated statistical considerations;<br>• Removed exclusion #9 because it duplicates exclusion #8;<br>• Added a row for "Healthcare Utilization" to the Schedule of study procedures;<br>• Clarified that stool collection will be processed at a central lab;<br>• Added that peripheral blood mononuclear cells will be collected at select sites;<br>• Added additional appendix-level study assessments to Appendix A;<br>• Updated drug storage specifications for Appendix A; and<br>• Other administrative changes throughout.
4.0	07NOV2023	• Updated synopsis for clarity and to align with protocol changes;<br>• Removed Section 1.3: Key Roles, added names to cover page;<br>• Clarified estimated sample size will be approximately 100 evaluable participants per treatment group per symptom cluster (an appendix with 1 control group and 2 dosing regimens across 3 symptom clusters would have a total of 900 participants);<br>• Revised endpoints for clarity;<br>• Added concomitant medication details;<br>• Revised and clarified throughout that participants can be enrolled in ONLY 1 symptom cluster: Assessments for a symptom cluster will be performed among participants who meet the symptom cluster-specific eligibility criteria. Participants will not be enrolled into a symptom cluster cohort for which they also meet symptom cluster specific exclusion criteria. Participants will be enrolled in the cluster for which they exhibit the most severe criteria. The Principal Investigator will decide in which cluster the participant will be enrolled in based on severity of symptoms;<br>• Revision of Figure 1, Figure 2, and Figure 3 to reflect changes in procedure implemented with this amendment;<br>• Moved Schedule of Procedures from the body of the protocol to the appendix;

Version	Date	Description of Change
		• Added details regarding what clinical laboratory tests will need to be obtained;<br>• Made in-person Screening Visit required for all participants;<br>• Indicated that local rapid SARS-CoV-2 can be any rapid test, not just rapid antigen test (RAT);<br>• Symptom cluster assessment schedule included in the Schedule of Procedures within appendix;<br>• Correction to timing of weekly assessments from 3 days to 5 days; now reads: Participants will have 5 days to complete weekly assessments<br>• Clarified DSQ-PEM requirements for Exercise Intolerance Cluster;<br>• Updated collection and analysis plan for Orthostatic Hypotension Questionnaire (will be collected at both Screening and Baseline as a repeat test in the first 100 participants to assess test-retest reliability);<br>• Clarified that Patient Report Outcomes for primary endpoint must be repeated at Baseline, if Baseline occurs > 7 days after Screening;<br>• Updated missing data language for clarity;<br>• Added CTCAE for severity grading of AEs;<br>• Appendix A updates: added information on PAXLOVID FDA approval, updated most common AEs, added criteria for thyroid function ESI reporting;<br>• Other administrative changes throughout.

12 REFERENCES

1. Cabrera Martimbianco AL, Pacheco RL, Bagattini Â M, Riera R. Frequency, signs and symptoms, and criteria adopted for long COVID-19: A systematic review. *Int J Clin Pract*. Oct 2021;75(10):e14357. doi:10.1111/ijcp.14357

2. Huang C, Huang L, Wang Y, et al. 6-month consequences of COVID-19 in patients discharged from hospital: a cohort study. *Lancet*. Jan 16 2021;397(10270):220-232. doi:10.1016/s0140-6736(20)32656-8

3. Hirschtick JL, Titus AR, Slocum E, et al. Population-Based Estimates of Post-acute Sequelae of Severe Acute Respiratory Syndrome Coronavirus 2 (SARS-CoV-2) Infection (PASC) Prevalence and Characteristics. *Clin Infect Dis*. Dec 6 2021;73(11):2055-2064. doi:10.1093/cid/ciab408

4. Bell ML, Catalfamo CJ, Farland LV, et al. Post-acute sequelae of COVID-19 in a non-hospitalized cohort: Results from the Arizona CoVHORT. *PLoS One*. 2021;16(8):e0254347. doi:10.1371/journal.pone.0254347

5. Zhu N ZD, Wang W. A Novel Coronavirus from Patients with Pneumonia in China. *N Engl J Med* 2020;382:727-733.

6. Goodman BP, Khoury JA, Blair JE, Grill MF. COVID-19 Dysautonomia. *Front Neurol*. 2021;12:624968. doi:10.3389/fneur.2021.624968

7. Barizien N, Le Guen M, Russel S, Touche P, Huang F, Vallée A. Clinical characterization of dysautonomia in long COVID-19 patients. *Sci Rep*. Jul 7 2021;11(1):14042. doi:10.1038/s41598-021-93546-5

8. Stewart S, Newson L, Briggs TA, Grammatopoulos D, Young L, Gill P. Long COVID risk - a signal to address sex hormones and women's health. *Lancet Reg Health Eur*. Dec 2021;11:100242. doi:10.1016/j.lanepe.2021.100242

9. Sudre CH, Murray B, Varsavsky T, et al. Attributes and predictors of long COVID. *Nat Med*. Apr 2021;27(4):626-631. doi:10.1038/s41591-021-01292-y

10. Asadi-Pooya AA, Akbari A, Emami A, et al. Risk Factors Associated with Long COVID Syndrome: A Retrospective Study. *Iran J Med Sci*. Nov 2021;46(6):428-436. doi:10.30476/ijms.2021.92080.2326

11. Su Y, Yuan D, Chen DG, et al. Multiple early factors anticipate post-acute COVID-19 sequelae. *Cell*. Mar 3 2022;185(5):881-895.e20. doi:10.1016/j.cell.2022.01.014

12. Phetsouphanh C, Darley DR, Wilson DB, et al. Immunological dysfunction persists for 8 months following initial mild-to-moderate SARS-CoV-2 infection. *Nat Immunol*. Feb 2022;23(2):210-216. doi:10.1038/s41590-021-01113-x

13. Buonsenso D, Di Giuda D, Sigfrid L, et al. Evidence of lung perfusion defects and ongoing inflammation in an adolescent with post-acute sequelae of SARS-CoV-2 infection. *Lancet Child Adolesc Health*. Sep 2021;5(9):677-680. doi:10.1016/s2352-4642(21)00196-6

14. Daniel Chertow SS, Sabrina Ramelli, Alison Grazioli, et al. SARS-CoV-2 infection and persistence throughout the human body and brain. *PREPRINT (Version 1) available at Research Square [https://doiorg/1021203/rs3rs-1139035/v1]*. 2021;

15. Zollner A, Koch R, Jukic A, et al. Postacute COVID-19 is Characterized by Gut Viral Antigen Persistence in Inflammatory Bowel Diseases. *Gastroenterology*. May 1 2022;163(2):495-506.e8. doi:10.1053/j.gastro.2022.04.037

16. Tejerina F, Catalan P, Rodriguez-Grande C, et al. Post-COVID-19 syndrome. SARS-CoV-2 RNA detection in plasma, stool, and urine in patients with persistent symptoms after COVID-19. *BMC Infect Dis*. Mar 3 2022;22(1):211. doi:10.1186/s12879-022-07153-4

17. Cheshire WP, Freeman R, Gibbons CH, et al. Electrodiagnostic assessment of the autonomic nervous system: A consensus statement endorsed by the American Autonomic Society, American Academy of Neurology, and the International Federation of Clinical Neurophysiology. *Clin Neurophysiol*. Feb 2021;132(2):666-682. doi:10.1016/j.clinph.2020.11.024

18. Organization; PAH. Case definitions for COVID-19 surveillance. Accessed August 25, 2022, 2022. *https://www.paho.org/en/case-definitions-covid-19-surveillance*

19. Soriano JB, Murthy S, Marshall JC, Relan P, Diaz JV. A clinical case definition of post-COVID-19 condition by a Delphi consensus. *Lancet Infect Dis*. Apr 2022;22(4):e102-e107. doi:10.1016/s1473-3099(21)00703-9

20. Organization; WH. Haemoglobin concentrations for the diagnosis of anaemia and assessment of severity. Vitamin and Mineral Nutrition Information System. . *http://apps.who.int/iris/bitstream/10665/85839/3/WHO_NMH_NHD_MNM_11.1_eng.pdf?ua=1*

21. Prevention; CfDCa. Alcohol and Public Health Updated April 19, 2022. Accessed August 25, 2022. *https://www.cdc.gov/alcohol/faqs.htm#:~:text=What%20does%20moderate%20drinking%20mean,women%2C%20when%20alcohol%20is%20consumed*

22. Cella D, Yount S, Rothrock N, et al. The Patient-Reported Outcomes Measurement Information System (PROMIS): progress of an NIH Roadmap cooperative group during its first two years. *Med Care*. May 2007;45(5 Suppl 1):S3-s11. doi:10.1097/01.mlr.0000258615.42478.55

23. Hays RD, Bjorner JB, Revicki DA, Spritzer KL, Cella D. Development of physical and mental health summary scores from the patient-reported outcomes measurement information system (PROMIS) global items. *Qual Life Res*. Sep 2009;18(7):873-80. doi:10.1007/s11136-009-9496-9

24. Ganesh R, Ghosh AK, Nyman MA, et al. PROMIS Scales for Assessment of Persistent Post-COVID Symptoms: A Cross Sectional Study. *J Prim Care Community Health*. Jan-Dec 2021;12:21501327211030413. doi:10.1177/21501327211030413

25. PROMIS ADULT PROFILE INSTRUMENTS. Accessed November 16, 2022. *https://staging.healthmeasures.net/images/PROMIS/manuals/PROMIS_Adult_Profile_Scoring_Manual.pdf*

26. The Patient Reported Outcomes Measurement Information System (PROMIS): A Walk Through the First Four Years, (2009).

27. Sletten DM, Suarez GA, Low PA, Mandrekar J, Singer W. COMPASS 31: a refined and abbreviated Composite Autonomic Symptom Score. *Mayo Clin Proc*. Dec 2012;87(12):1196-201. doi:10.1016/j.mayocp.2012.10.013

28. Cotler J, Holtzman C, Dudun C, Jason LA. A Brief Questionnaire to Assess Post-Exertional Malaise. *Diagnostics (Basel)*. Sep 11 2018;8(3)doi:10.3390/diagnostics8030066

29. Goldstein RAEaRS. *Comprehensive Biomedical Physics*. vol 10. Elsevier; 2014.

30. Revill SM, Morgan MD, Singh SJ, Williams J, Hardman AE. The endurance shuttle walk: a new field test for the assessment of endurance capacity in chronic obstructive pulmonary disease. *Thorax*. Mar 1999;54(3):213-22. doi:10.1136/thx.54.3.213

31. Yost KJ, Eton DT, Garcia SF, Cella D. Minimally important differences were estimated for six Patient-Reported Outcomes Measurement Information System-Cancer scales in advanced-stage cancer patients. *J Clin Epidemiol*. May 2011;64(5):507-16. doi:10.1016/j.jclinepi.2010.11.018

32. Kroenke K, Stump TE, Chen CX, et al. Minimally important differences and severity thresholds are estimated for the PROMIS depression scales from three randomized clinical trials. *J Affect Disord*. Apr 1 2020;266:100-108. doi:10.1016/j.jad.2020.01.101

33. Terwee CB, Peipert JD, Chapman R, et al. Minimal important change (MIC): a conceptual clarification and systematic review of MIC estimates of PROMIS measures. *Qual Life Res*. Oct 2021;30(10):2729-2754. doi:10.1007/s11136-021-02925-y

34. University; N. Meaningful Change for PROMIS(R). Accessed January 20, 2023. *https://www.healthmeasures.net/score-and-interpret/interpret-scores/promis/meaningful-change*

35. Norman GR, Sloan JA, Wyrwich KW. Interpretation of changes in health-related quality of life: the remarkable universality of half a standard deviation. *Med Care*. May 2003;41(5):582-92. doi:10.1097/01.Mlr.0000062554.74615.4c

36. Ge M, Durham L, Meyer D, Xie W, Thomas N. Covariate-Adjusted Difference in Proportions from Clinical Trials Using Logistic Regression and Weighted Risk Differences. *Drug Information Journal - DRUG INF J*. 07/01 2011;45:481-493. doi:10.1177/009286151104500409

37. Benkeser D, Díaz I, Luedtke A, Segal J, Scharfstein D, Rosenblum M. Improving precision and power in randomized trials for COVID-19 treatments using covariate adjustment, for binary, ordinal, and time-to-event outcomes. *Biometrics*. Dec 2021;77(4):1467-1481. doi:10.1111/biom.13377

38. Pfizer. PAXLOVID - nirmatrelvir and ritonavir. Updated June 28, 2022. Accessed July 20, 2022. *https://labeling.pfizer.com/ShowLabeling.aspx?id=16474*

39. Hammond J, Leister-Tebbe H, Gardner A, et al. Oral Nirmatrelvir for High-Risk, Nonhospitalized Adults with Covid-19. *N Engl J Med*. Apr 14 2022;386(15):1397-1408. doi:10.1056/NEJMoa2118542

40. PAXLOVID Prescribing Information. 2023. *https://www.accessdata.fda.gov/drugsatfda_docs/label/2023/217188s000lbl.pdf*

41. Catlin NR, Bowman CJ, Campion SN, et al. Reproductive and developmental safety of nirmatrelvir (PF-07321332), an oral SARS-CoV-2 M(pro) inhibitor in animal models. *Reprod Toxicol*. Mar 2022;108:56-61. doi:10.1016/j.reprotox.2022.01.006

42. Sathish JG, Bhatt S, DaSilva JK, et al. Comprehensive Nonclinical Safety Assessment of Nirmatrelvir Supporting Timely Development of the SARS-COV-2 Antiviral Therapeutic, Paxlovid™. *Int J Toxicol*. Aug 2022;41(4):276-290. doi:10.1177/10915818221095489

43. Singh RSP, Toussi SS, Hackman F, et al. Innovative Randomized Phase I Study and Dosing Regimen Selection to Accelerate and Inform Pivotal COVID-19 Trial of Nirmatrelvir. *Clin Pharmacol Ther*. Jul 2022;112(1):101-111. doi:10.1002/cpt.2603

44. Peluso MJ, Anglin K, Durstenfeld MS, et al. Effect of Oral Nirmatrelvir on Long COVID Symptoms: 4 Cases and Rationale for Systematic Studies. *Pathog Immun*. 2022;7(1):95-103. doi:10.20411/pai.v7i1.518

45. Ford ES, Simmons W, Karmarkar EN, et al. Successful treatment of prolonged, severe COVID-19 lower respiratory tract disease in a B-cell ALL patient with an extended course of remdesivir and nirmatrelvir/ritonavir. *Clin Infect Dis*. Nov 3 2022;doi:10.1093/cid/ciac868

13 APPENDIX A (PAXLOVID PHASE 2)

This appendix describes a prospective, multi-center, double-blind, randomized controlled phase 2 trial evaluating nirmatrelvir/ritonavir (PAXLOVID) in 2 dosing durations for PASC. PAXLOVID was FDA-approved in May 2023 for the treatment of mild-to-moderate COVID-19 in adults who are at high risk for progression to severe COVID-19, including hospitalization or death. Within this study, participants will be randomized 1:1:1 to 15 days of active intervention (nirmatrelvir/ritonavir) with 10 days of control (placebo/ritonavir), 25 days of active intervention, or 25 days of control, within 3 symptom clusters. The intervention will be administered in 2 kits (15 days followed by 10 days). For this appendix, the midpoint of drug dosing is approximately 13 days; the Drug Dosing Midpoint Clinic Visit will occur at Day 15 (-5 days).

13.1 RISK ASSESSMENT

Serious and unexpected AEs may occur that have not been previously reported with PAXLOVID use.[38] The safety of nirmatrelvir and ritonavir is based on data from three Phase 2/3, controlled trials in non-hospitalized adults (refer to PAXLOVID Investigator's Brochure).[39] The most common adverse reactions (occurring at a ≥ 1% frequency in the nirmatrelvir/ritonavir group and at a greater frequency than in the placebo group) are dysgeusia and diarrhea.[40]

No PAXLOVID human data are currently available to evaluate for a drug-associated risk of major birth defects, miscarriage, adverse maternal or fetal outcomes, or lactation effects. However, 1 study is currently recruiting pregnant women with mild or moderate COVID-19 (NCT05386472), and another study is ongoing to examine PAXLOVID in healthy lactating women (NCT05441215). Pre-clinical data indicate that there are no clinically relevant risks associated with PAXLOVID administration during pregnancy and in males and females of reproductive age.[41] Published observational studies on ritonavir use in pregnant women have not identified an increased risk of major birth defects. Published studies with ritonavir are insufficient to identify a drug-associated risk of miscarriage. The benefit of taking PAXLOVID during pregnancy may be greater than the risk from the treatment; however, the risk/benefit of treatment for PASC during pregnancy has not been established.

13.2 ADDITIONAL APPENDIX-LEVEL EXPLORATORY OBJECTIVE(S)

OBJECTIVE(S)	OUTCOME MEASURES	ENDPOINTS
Exploratory		
Describe the effect of dose on efficacy of intervention within each PASC symptom cluster	**Cognitive dysfunction symptom cluster:** PROMIS – cognitive 8a<br>**Autonomic dysfunction symptom cluster:** OHQ<br>**Exercise intolerance symptom cluster:** DSQ-PEM	**Cognitive dysfunction symptom cluster:** Participants meeting pre-specified change from baseline to Day 90 on PROMIS - cognitive 8a T-score<br>**Autonomic dysfunction symptom cluster:** Participants meeting pre-specified change from baseline to Day 90 on the OHQ<br>**Exercise intolerance symptom cluster:** Participants meeting pre-specified change from baseline to Day 90 on the DSQ-PEM

OBJECTIVE(S)	OUTCOME MEASURES	ENDPOINTS
Safety		
Characterize the effect of dose on safety and tolerability of intervention in PASC	• Serious adverse events (SAEs)<br>• Events of Special Interest (ESIs)<br>• Adverse events (AEs)<br>• Number of missed doses (adherence)	• Occurrence of individual SAEs and AEs<br>• Occurrence of any 1 or more SAEs and AEs<br>• Occurrence of AEs and SAEs leading to discontinuation<br>• Occurrence of ESIs<br>• Number of missed doses (adherence)

Refer to the SAP for analysis details.

13.3 ADDITIONAL APPENDIX-LEVEL EXCLUSION CRITERIA

1. Known pregnancy[1]
2. Active or expected breastfeeding during the study
3. Known eGFR < 30 mL/min
4. Known severe hepatic impairment (Child-Pugh Class C)
5. Current use of drugs highly dependent on CYP3A for clearance[2] and for which elevated concentrations are associated with serious and/or life-threatening reactions and which cannot be interrupted during the time of study administration and within 7 days before and after study drug administration
6. Current use of potent CYP3A inducers[2] where significantly reduced nirmatrelvir or ritonavir plasma concentrations may be associated with the potential for loss of virologic response and possible resistance

[1]*A pregnancy test must be performed at the Baseline Visit for participants who are capable of becoming pregnant.*
[2]*A guide of drugs that may be contraindicated are listed in CONTRAINDICATIONS of the Full Prescribing Information for PAXLOVID.*

13.4 ADDITIONAL APPENDIX-LEVEL STUDY ASSESSMENTS

Safety Labs for appendix A are drawn in addition to platform protocol labs at the following time points and are run locally at the site. All non-serious AEs will be collected and reported from start of study drug dosing to end of study drug administration for this appendix at all timepoints that designate collection of no-serious AEs in Schedule of Procedures: Table 4, Table 5, and Table 6.

Screening Visit (Visit 1):
- Pregnancy test (for participants who are capable of becoming pregnant)
- Thyroid stimulating hormone (TSH)
- Total and free triiodothyronine (T3, total and free)
- Total and free thyroxine (T4, total and free)

Drug Dosing Midpoint Clinic Visit (Visit 3):
- Thyroid stimulating hormone (TSH)
- Total and free triiodothyronine (T3, total and free)

- Total and free thyroxine (T4, total and free)
- AST, ALT, bilirubin

End of Dosing Clinic Visit (Visit 4):

- Thyroid stimulating hormone (TSH)
- Total and free triiodothyronine (T3, total and free)
- Total and free thyroxine (T4, total and free)
- AST, ALT, bilirubin

Day 90 Visit (Visit 7):

- Thyroid stimulating hormone (TSH)
- Total and free triiodothyronine (T3, total and free)
- Total and free thyroxine (T4, total and free)
- AST, ALT, bilirubin

13.4.1 SCHEDULE OF PROCEDURES

Table 4. Schedule of Procedures – PAXLOVID, Exercise Intolerance Symptom Cluster

	Screening	Baseline	Drug Dosing Midpoint Clinic Visit	End of Dosing Clinic Visit	Follow-up Through day 179				End of Study Visit[1]
**Visit #**	**1**	**2**	**3**	**4**	**5**	**6**	**7**	**8**	**9**
**PROCEDURE**	**Day -14 to 0**	**Day -2 to 0**	**Day 15 – 3 days**	**Day 25 + 3 days**	**Day 45 ± 5 days**	**Day 60 ± 5 days**	**Day 90[3] ± 5 days**	**Day 120 ± 5 days**	**Day 183 ± 7 Days**
Informed consent	X								
Demographics	X								
Medical History	X								
Concomitant Medications	X	X	X	X	X	X	X	X	X
PROMIS-29+2		X	●——	Weekly[2]	——	——►	X	●——	Weekly[2] ——►
PASC Symptom Questionnaire		X	●——	Weekly[2]	——	——►	X	●——	Weekly[2] ——►
CTCSQ	X			X			X		X
DSQ-PEM (7-day look back)	X		●——	Weekly[2]	——	——►	X	●——	Weekly[2] ——►
DSQ-PEM (SLV look back)	X[7]	X[7]		X[7]			X[7]		
ESWT		X[7]		X[7]			X[7]		
ISWT	X[7,8]								
Cardiopulmonary test (**select sites**)		X[7]		X[7]			X[7]		
Appendix-Level Eligibility Criteria	X								
Pregnancy Test	X[9]								
Comprehensive Metabolic Panel	X[9]								
Complete Blood Count	X[9]		X	X			X		
PT, INR, aPTT, CRP, D-Dimer	X[9]		X	X			X		
Biorepository Blood	X[9]		X	X	X[5]	X[5]	X	X[5]	
Safety Blood Laboratory tests	X[9]		X[4]	X[4]			X[4]		
Nasal Swabs	X[9]		X	X	X[5]	X[5]	X	X[5]	
Stool Collection	X[9]						X		
Height and weight		X							
BP and heart rate (supine & standing)		X		X			X		
Randomization		X							
Study Drug Distribution		X	X						
AEs[6]		X	X	X					
SAEs, SUSARs		X	X	X	X	X	X	X	X
Healthcare Utilization			X	X			X		X
ESIs			X	X	X	X	X	X	X
Drug Adherence			X	X					

Underlined visits must be done in-person.

[1]End of Study Visit will occur by telephone

[2]Remote data collection approximately weekly during drug dosing and follow-up unless presenting to clinic for optional blood collection, as applicable. Participants will have 5 days to complete weekly assessments

[3]Primary endpoint assessment

[4]Safety blood laboratory assessments for Appendix A include: thyroid-stimulating hormone, free triiodothyronine (T3, total and free), total triiodothyronine, free thyroxine (FT4) testing, total thyroxine, AST, ALT, bilirubin

[5]Optional nasal swab (PCR only) and blood draws

[6]Non-serious AEs will be collected from the start of study drug administration through Day 25 + 3 days (Visit #4).

[7]The DSQ-PEM SLV must be completed remotely +1 to 2 days after any visit that includes ESWT and/or Cardiopulmonary test to assess PEM.

[8]The ISWT must be completed at an in-person screening visit that occurs at least 3 days before the Baseline Visit.

[9]Can be collected at Screening or Baseline.

Table 5. Schedule of Procedures – PAXLOVID, Cognitive Dysfunction Symptom Cluster

	Screening	Baseline	Drug Dosing Midpoint Clinic Visit	End of Dosing Clinic Visit	Follow-up Through day 179				End of Study Visit[1]
**Visit #**	**1**	**2**	**3**	**4**	**5**	**6**	**7**	**8**	**9**
**PROCEDURE**	**Day -14 to 0**	**Day -2 to 0**	**Day 15 – 3 days**	**Day 25 + 3 days**	**Day 45 ± 5 days**	**Day 60 ± 5 days**	**Day 90[3] ± 5 days**	**Day 120 ± 5 days**	**Day 183 ± 7 Days**
Informed consent	X								
Demographics	X								
Medical History	X								
Concomitant Medications	X	X	X	X	X	X	X	X	X
PROMIS-29+2		X	● Weekly[2] →				X	● Weekly[2] →	
PASC Symptom Questionnaire		X	● Weekly[2] →				X	● Weekly[2] →	
CTCSQ	X			X			X		X
PROMIS-cognitive 8a	X		● Weekly[2] →				X	● Weekly[2] →	
ECOG 2	X		● Weekly[2] →				X	● Weekly[2] →	
Neurocognitive battery		X[7]		X[7]			X[7]		
DSQ-PEM (SLV)		X[7]		X[7]			X[7]		
Appendix-Level Eligibility Criteria	X								
Pregnancy Test	X[8]								
Comprehensive Metabolic Panel	X[8]								
Complete Blood Count	X[8]		X	X			X		
PT, INR, aPTT, CRP, D-Dimer	X[8]		X	X			X		
Biorepository Blood	X[8]		X	X	X[5]	X[5]	X	X[5]	
Safety Blood Laboratories	X[8]		X[4]	X[4]			X[4]		
Nasal Swabs	X[8]		X	X	X[5]	X[5]	X	X[5]	
Stool Collection	X[8]						X		
Height and weight		X							
BP and heart rate (supine & standing)		X		X			X		
Randomization		X							
Study Drug Distribution		X	X						
AEs[6]		X	X	X					
SAEs, SUSARs		X	X	X	X	X	X	X	X
Healthcare Utilization			X	X			X		X
ESIs			X	X	X	X	X	X	X
Drug Adherence			X	X					
Underlined visits must be done in-person.									

[1]End of Study Visit will occur by telephone

[2]Remote data collection approximately weekly during drug dosing and follow-up unless presenting to clinic for optional blood collection, as applicable. Participants will have 5 days to complete weekly assessments

[3]Primary endpoint assessment

[4]Safety blood laboratory assessments for Appendix A include: thyroid-stimulating hormone, free triiodothyronine (T3, total and free), total triiodothyronine, free thyroxine (FT4) testing, total thyroxine, AST, ALT, bilirubin.
[5]Optional nasal swab (PCR only) and blood draws
[6]Non-serious AEs will be collected from the start of study drug administration through Day 25 + 3 days (Visit #4).
[7]The DSQ-PEM SLV must be completed remotely +1 to 2 days after any visit that includes the Neurocognitive Battery Test to assess PEM.
[8]Can be collected at Screening or Baseline.

Table 6. Schedule of Procedures – PAXLOVID, Autonomic Dysfunction Symptom Cluster

	Screening	Baseline	Drug Dosing Midpoint Clinic Visit	End of Dosing Clinic Visit	Follow-up Through day 179				End of Study Visit[1]
**Visit #**	**1**	**2**	**3**	**4**	**5**	**6**	**7**	**8**	**9**
**PROCEDURE**	**Day -14 to 0**	**Day -2 to 0**	**Day 15 – 3 days**	**Day 25 + 3 days**	**Day 45 ± 5 days**	**Day 60 ± 5 days**	**Day 90[3] ± 5 days**	**Day 120 ± 5 days**	**Day 183 ± 7 Days**
Informed consent	X								
Demographics	X								
Medical History	X								
Concomitant Medications	X	X	X	X	X	X	X	X	X
PROMIS-29+2		X	●———	Weekly[2]	———	——→	X	● Weekly[2]	→
PASC Symptom Questionnaire		X	●———	Weekly[2]	———	——→	X	● Weekly[2]	→
CTCSQ	X			X			X		X
COMPASS 31	X		●———	Weekly[2]	———	——→	X	● Weekly[2]	→
OHQ	X	X[8]	●———	Weekly[2]	———	——→	X	● Weekly[2]	→
Active Stand Test		X[7]		X[7]			X[7]		
Full Autonomic Testing (**select sites**)		X[7]		X[7]			X[7]		
DSQ-PEM (SLV)		X[7]		X[7]			X[7]		
Appendix-Level Eligibility Criteria	X								
Pregnancy Test	X[9]								
Comprehensive Metabolic Panel	X[9]								
Complete Blood Count	X[9]		X	X			X		
PT, INR, aPTT, CRP, D-Dimer	X[9]		X	X			X		
Biorepository Blood	X[9]		X	X	X[5]	X[5]	X	X[5]	
Safety Blood Laboratories[4]	X[9]		X[4]	X[4]			X[4]		
Nasal Swabs	X[9]		X	X	X[5]	X[5]	X	X[5]	
Stool Collection	X[9]						X		
Height and weight		X							
BP and heart rate (supine & standing)		X		X			X		
Randomization		X	X						
Study Drug Distribution		X	X						
AEs[6]		X	X	X					
SAEs, SUSARs		X	X	X	X	X	X	X	X
Healthcare Utilization			X	X			X		X
ESIs			X	X	X	X	X	X	X
Drug Adherence			X	X					
Underlined visits must be done in-person.									

[1]End of Study Visit will occur by telephone
[2]Remote data collection approximately weekly during drug dosing and follow-up unless presenting to clinic for optional blood collection, as applicable. Participants will have 5 days to complete weekly assessments

[3]Primary endpoint assessment
[4]Safety blood laboratory assessments for Appendix A include: thyroid-stimulating hormone, free triiodothyronine (T3, total and free), total triiodothyronine, free thyroxine (FT4) testing, total thyroxine, AST, ALT, Bilirubin
[5]Optional nasal swab (PCR only) and blood draws
[6]Non-serious AEs will be collected from the start of study drug administration through Day 25 + 3 days (Visit #4).
[7]The DSQ-PEM SLV must be completed remotely +1 to 2 days after any visit that includes Active Stand and/or Full Autonomic Testing to assess PEM
[8]Required as a Baseline assessment for the first 100 participants.
[9] Can be collected at Screening or Baseline.

13.5 PAXLOVID INFORMATION

In December 2021, the US FDA issued an EUA for use of the unapproved PAXLOVID, which includes nirmatrelvir and ritonavir for the treatment of mild-to-moderate COVID-19 in adults and pediatric patients (12 years of age and older weighing at least 40 kg) who test positive for SARS-CoV-2 and are at high risk for progression to severe COVID-19.[38] In May 2023, the FDA approved the use of PAXLOVID in adults with mild-to-moderate COVID-19 at a dose of 300 mg nirmatrelvir with 100 mg ritonavir twice daily for 5 days.

PAXLOVID is nirmatrelvir tablets co-packaged with ritonavir tablets. Nirmatrelvir is a SARS-CoV-2 main protease inhibitor. Ritonavir is an HIV-1 protease inhibitor and CYP3A inhibitor. Since CYP3A metabolizes nirmatrelvir, ritonavir produces higher systemic concentrations and longer half-life of nirmatrelvir.

13.5.1 PRECAUTIONS

The healthcare provider should consult other appropriate resources such as the prescribing information for the interacting drug for comprehensive information on dosing or monitoring with concomitant use of a strong CYP3A inhibitor such as ritonavir. No dosage adjustment is required when co-administered with other products containing ritonavir or cobicistat. Participants on ritonavir- or cobicistat-containing Human Immunodeficiency Virus (HIV) or Hepatitis C viral regimens should continue their treatment as indicated.

PAXLOVID use may lead to a risk of HIV-1 developing resistance to HIV protease inhibitors in individuals with uncontrolled or undiagnosed HIV-1 infection.[40]

Initiation of medications that inhibit or induce CYP3A may increase or decrease concentrations of PAXLOVID, respectively. These interactions may lead to:

- Clinically significant adverse reactions, potentially leading to severe, life-threatening, or fatal events from greater exposures of concomitant medications
- Clinically significant adverse reactions from greater exposures of PAXLOVID
- Loss of therapeutic effect of PAXLOVID and possible development of viral resistance

13.5.2 FORMULATION, APPEARANCE, PACKAGING, AND LABELING

PAXLOVID is a combination of nirmatrelvir and ritonavir tablets.

Formulation:

- Nirmatrelvir is available as immediate-release, film-coated tablets. Each tablet contains 150 mg nirmatrelvir with the following inactive ingredients: colloidal silicon dioxide, croscarmellose sodium, lactose monohydrate, microcrystalline cellulose, and sodium stearyl fumarate. The following are the ingredients in the film coating: hydroxy propyl methylcellulose, iron oxide red, polyethylene glycol, and titanium dioxide.

- Ritonavir is available as film-coated tablets. Each tablet contains 100 mg ritonavir with the following inactive ingredients: anhydrous dibasic calcium phosphate, colloidal silicon dioxide, copovidone, sodium stearyl fumarate, and sorbitan monolaurate. The film coating may include the following ingredients: colloidal anhydrous silica, colloidal silicon dioxide, hydroxypropyl cellulose, hypromellose, polyethylene glycol, polysorbate 80, talc, and titanium dioxide.

Appearance:

- Nirmatrelvir is supplied as oval, pink, film-coated tablets.
- Ritonavir is supplied as ovaloid, white, film-coated tablets.

13.5.3 DRUG DISPENSING, STORAGE, AND STABILITY

Store at 15°C to 30°C (59°F to 86°F). Participants will receive 2 kits. Kit 1 will be provided at the Baseline Visit to cover days 1 to 15. Kit 2 will be provided at the midpoint dosing visit to cover days 16 to 25.

13.5.4 DOSING AND ADMINISTRATION

PAXLOVID dosage is 300 mg nirmatrelvir (two 150 mg tablets) with 100 mg ritonavir (one 100 mg tablet). All 3 tablets (2 of nirmatrelvir and 1 of ritonavir) must be taken together and no more than 15 minutes apart. For this appendix, all 3 tablets are taken together twice daily by mouth for 25 days. PAXLOVID tablets can be taken with or without food, however, taking with food may improve tolerability. PAXLOVID tablets should be swallowed whole, not chewed, broken, or crushed.

All participants will undergo dosing for 25 days. Those randomized to PAXLOVID for 15 days will receive 15 days of active intervention (nirmatrelvir/ritonavir) and 10 days of control (placebo/ritonavir). Those randomized to PAXLOVID for 25 days will receive 25 days of active intervention.

Each participant will receive 2 kits that include a combination of blister cards and bottles. All participants will be instructed to take 2 tablets from the blister cavities and 1 tablet from the bottle for each dose. Refer to the MOP for details.

A dose reduction is necessary for participants with moderate renal impairment (eGFR ≥ 30 to < 60 mL/min): reduce to 150 mg nirmatrelvir (one 150 mg tablet) with 100 mg ritonavir (one 100 mg tablet), with both tablets taken together twice daily for the dosing period. No dose reduction is needed in participants with mild renal impairment (eGFR ≥ 60 to < 90 mL/min).

If the participant misses a dose of PAXLOVID within 8 hours of the time it is usually taken, the participant should take it as soon as possible and resume the normal dosing schedule. If the participant misses a dose by more than 8 hours, the participant should not take the missed dose but instead take the next dose at the regularly scheduled time. The participant should not double the dose to make up for a missed dose.

For this study, any dose of nirmatrelvir greater than 900 mg (6 nirmatrelvir tablets) or ritonavir greater than 300 mg (3 ritonavir tablets) within a 24-hour time period will be considered an overdose. There is no specific treatment for an overdose. Overdose is reportable to Pfizer Safety by DCRI Safety Surveillance **only when associated with an SAE**. In the event of an overdose, the investigator should:

1. Closely monitor the participant for any AEs/SAEs and laboratory abnormalities for at least 5 half-lives or 28 calendar days after the overdose of study intervention (whichever is longer).
2. Document the quantity of the excess dose as well as the duration of the overdose in the CRF.

3. If there is an associated SAE, the SAE is reported within the CRF within 24 hours of site awareness.

Decisions regarding dose interruptions or modifications will be made by the site investigator based on the clinical evaluation of the participant.

13.5.5 RATIONALE FOR SELECTION OF DOSE

Pre-clinical studies:
Nirmatrelvir has been characterized in nonclinical safety pharmacology and repeat-dose toxicity studies in the rat and non-human primate. Ritonavir was not included in pre-clinical safety studies for multiple reasons, as noted by Sathish et al.[42]:

- ritonavir is a marketed drug with a well-characterized pre-clinical and clinical safety profile, and no overlapping or additive toxicities between nirmatrelvir and ritonavir are expected;
- the exposure-enhancing effects of ritonavir seen in humans do not translate completely in animals;
- high exposures of nirmatrelvir in pre-clinical studies were achieved through use of solvate and formulations approaches.

High-dose nirmatrelvir administration (75 mg/kg per dose twice daily, by mouth) over a 5-day treatment period in cynomolgus monkeys produced cardiovascular effects limited to transient increases in BP and decreases in heart rate. Cardiovascular effects were not observed in low dose (20 mg/kg per dose twice daily, by mouth). Additionally, no adverse findings were observed in repeat dose toxicity studies up to 29 days in rats (up to 1,000 mg/kg daily, by mouth) or monkeys (up to 600 mg/kg daily, by mouth).

Clinical studies:
A first-in-human study (NCT04756531) assessed nirmatrelvir safety, tolerability, and pharmacokinetics following single-ascending doses including food effect, multiple-ascending doses, and at supra-therapeutic exposure in healthy 18 to 60-year-old patients.[43] Participants who received multiple-ascending doses were randomly assigned to control or escalating nirmatrelvir dose levels (75, 250, and 500 mg), all administered twice daily as a suspension for 10 days with ritonavir 100 mg, under fasting conditions. At all dose levels, nirmatrelvir plasma concentrations reached steady state by Day 2 and remained steady through Day 10. Across all multiple-ascending dose groups and control, treatment-related AEs included mild dysgeusia (n = 3) and mild increases in blood thyroid stimulating hormone (n = 5). No severe AEs or SAEs were reported.

Moreover, a supra-therapeutic oral dose of nirmatrelvir as a 2,250-mg suspension (dosed as 3 split doses of 750 mg administered at 0, 2, and 4 hours) enhanced with 100 mg ritonavir increased the mean plasma concentration ~ 5 times higher than that of the 500 mg nirmatrelvir multiple-ascending dose cohort on Day 1. Even with this high concentration, only 1 case of mild nausea in the treatment group was observed. No severe AEs or SAEs were reported.

In clinical studies in participants at high risk of progression to severe COVID-19 illness, PAXLOVID, as compared to placebo, was associated with an approximately 0.9 log10 copies/mL greater decline in viral RNA levels in nasopharyngeal samples through Day 5,[38] which is the approved dosing duration for acute COVID-19.[38]

To date the appropriate duration of therapy for PASC remains unknown. This study will test 2 dosing durations, 15 days and 25 days, which have been chosen to enable evaluation of dose response of

prolonged therapy within the range of dosing durations previously used in select clinical practice (e.g., immunosuppression).

No randomized clinical trial has been performed to study PAXLOVID in patients with PASC. Case studies of patients with PASC taking PAXLOVID over 5 days show equivocal improvements in symptom resolution.[44]

The planned dosing duration in this study (15 and 25 days) is nearly 3 and 5 times that typically administered and currently under EUA for administration to those with acute SARS-CoV-2 infection for prevention of severe disease. Previous clinical trials have confirmed the safety of PAXLOVID treatment up to a duration 10 days and data suggests similar safety/tolerability of 5- and 10-day treatment durations. In addition, prolonged durations of PAXLOVID (up to 20 days) have been used in heavily immunosuppressed patients with persistent SARS-CoV-2 infection,[45] and for other disease processes where viral persistence and reactivation are of concern (e.g., herpes simplex virus, especially neonatal herpes simplex virus), prolonged durations of antivirals (sometimes up to 1 year) are standard. Moreover, more prolonged durations might increase the probability of viral eradication. Viral eradication is the mechanism by which antivirals are believed to potentially mitigate PASC.

13.6 CONTROL INFORMATION

In order to maintain blinding with the distinct taste of ritonavir, the control for this appendix will be the combination of ritonavir and placebo. Data obtained from a mouse study show no evidence of an antiviral effect of ritonavir alone (see Figure 5). As such, ritonavir with placebo is expected to be an appropriate control for the PAXLOVID appendix.

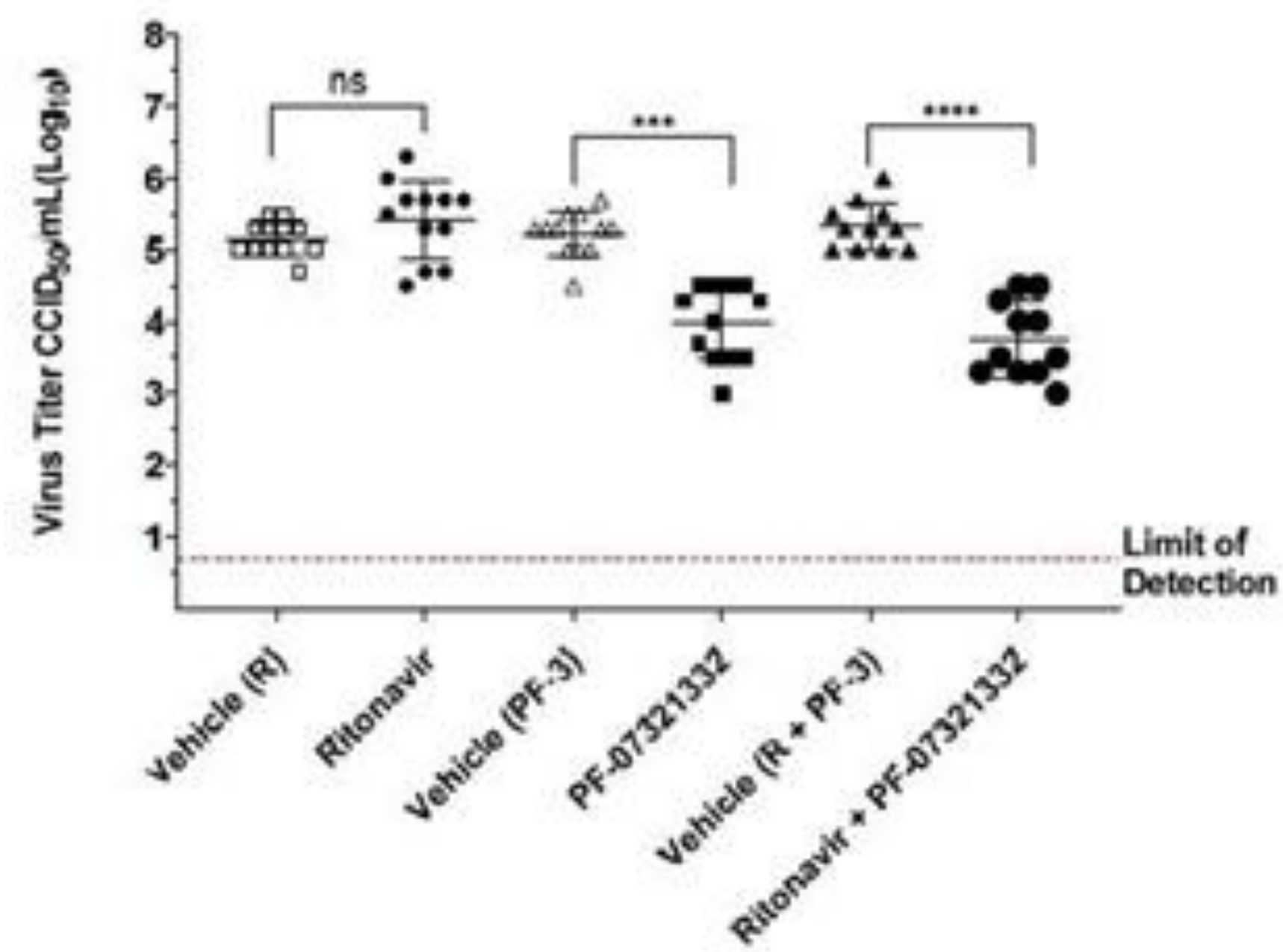

Figure 5. Effect of PAXLOVID (PF-07321332) on lung viral titers following oral administration in BALB/c mice infected with SARS-COV-2-MA10

Twice daily oral administration of PF-07321332 alone at 300 mg/kg or at 300 mg/kg in the presence of 50 mg/kg ritonavir reduced SARS-CoC-2 lung viral titer in mice. Mice (N=12) were inoculated with MA-SARS-CoV-2. ***p<0.0005; ****p<0.0001; PF-3 = PF-07321332; R = ritonavir.

13.6.1 FORMULATION, APPEARANCE, PACKAGING, AND LABELING

The control formulation includes 2 placebo tablets and 1 ritonavir tablet. The following ingredients are in a 210 mg placebo tablet: microcrystalline cellulose, NF (MC-102); pregelatinized starch, NF (Starch 1500); croscarmellose sodium, NF (Vivasol, GF Grade); colloidal silicon dioxide, NF (Aerosil 200); and magnesium stearate, NF (2257).

Control appearance will match that of all 3 PAXLOVID tablets (2 nirmatrelvir tablets and 1 ritonavir tablet):

- The placebo for the 150 mg nirmatrelvir tablet is supplied as an oval, pink, film-coated tablets.
- Ritonavir is supplied as ovaloid, white, film-coated tablets.

The control packaging matches the packaging described in Section 13.5.2.

13.6.2 CONTROL DISPENSING, STORAGE, AND STABILITY

Store at 15°C to 30°C (59°F to 86°F).

13.6.3 DOSING AND ADMINISTRATION

Control dosing and administration will occur according to Section 13.5.4 in order to maintain blinding.

13.7 EVENTS OF SPECIAL INTEREST (ESI)

Alteration in Thyroid Function

Alterations in thyroid function have been observed with patients who take PAXLOVID for longer than 10 days. Therefore, all participants in the study will undergo evaluation of thyroid stimulating hormone (TSH), total and free triiodothyronine (T3), and total and free thyroxine (T4) at baseline, drug dosing midpoint clinic visit, end of dosing (EOD), and 90 days.

The following will result in reporting an ESI:

- New *hypothyroid* occurrence, indicated by new high TSH (according to labs) with symptoms of hypothyroid.
- New *hyperthyroid* occurrence, indicated by either:
    a. High T4 with symptoms of hyperthyroid; or
    b. New High T3 with symptoms of hyperthyroid

Drug-Induced Liver Injury (DILI)

Humans exposed to a drug who show no sign of liver injury (as determined by elevations in transaminases) are termed “tolerators,” while those who show transient liver injury but adapt are termed “adaptors.” In some participants, transaminase elevations are a harbinger of a more serious potential outcome. These participants fail to adapt and therefore are “susceptible” to progressive and serious liver injury, commonly referred to as DILI. Participants who experience a transaminase elevation above 3 × upper limit of normal (ULN) should be monitored more frequently to determine if they are “adaptors” or are “susceptible.”

In the majority of DILI cases, elevations in aspartate aminotransferase (AST) and/or alanine transaminase (ALT) precede total bilirubin (T bili) elevations (> 2 × ULN) by several days or weeks. The increase in T bili typically occurs while AST/ALT is/are still elevated above 3 × ULN (i.e., AST/ALT and

T bili values will be elevated within the same laboratory sample). In rare instances, by the time T bili elevations are detected, AST/ALT values might have decreased. This occurrence is still regarded as a potential DILI. Therefore, abnormal elevations in either AST **OR** ALT in addition to T bili that meet the criteria outlined below are considered potential DILI (assessed per Hy's law criteria) cases and should always be considered important medical events (SAEs), even before all other possible causes of liver injury have been excluded.

The threshold of laboratory abnormalities for a potential DILI case depends on the participant's individual baseline values and underlying conditions. Participants who present with the following laboratory abnormalities should be evaluated further as potential DILI (Hy's law) cases to definitively determine the etiology of the abnormal laboratory values:

1. Participants with AST/ALT and T bili baseline values within the normal range who subsequently present with AST **OR** ALT values ≥ 3 × ULN **AND** a T bili value ≥ 2 × ULN with no evidence of hemolysis and an alkaline phosphatase value < 2 × ULN or not available.
2. For participants with baseline AST **OR** ALT **OR** T bili values above the ULN, the following threshold values are used in the definition mentioned above, as needed, depending on which values are above the ULN at baseline:
   a. Preexisting AST or ALT baseline values above the normal range: AST or ALT values ≥ 2 times the baseline values AND ≥ 3 × ULN; or ≥ 8 × ULN (whichever is smaller).
   b. Preexisting values of T bili above the normal range: T bili level increased from baseline value by an amount of ≥ 1 × ULN **or** if the value reaches ≥ 3 × ULN (whichever is smaller).

Rises in AST/ALT and T bili separated by more than a few weeks should be assessed individually based on clinical judgment; any case where uncertainty remains as to whether it represents a potential Hy's law case should be reviewed with the sponsor.

The participant should return to the investigator site and be evaluated as soon as possible, preferably within 48 hours from awareness of the abnormal results. This evaluation should include laboratory tests, detailed history, and physical assessment.

In addition to repeating measurements of AST and ALT and T bili for suspected Hy's law cases, additional laboratory tests should include albumin, creatinine kinase, direct and indirect bilirubin, gamma-glutamyl transferase, prothrombin time test and INR (PT/INR), total bile acids, and alkaline phosphatase. Consideration should also be given to drawing a separate tube of clotted blood and an anticoagulated tube of blood for further testing, as needed, for further contemporaneous analyses at the time of the recognized initial abnormalities to determine etiology. A detailed history, including relevant information, such as review of ethanol, acetaminophen/paracetamol (either by itself or as a coformulated product in prescription or over-the-counter medications), recreational drug, or supplement (herbal) use and consumption, family history, sexual history, travel history, history of contact with a jaundiced person, surgery, blood transfusion, history of liver or allergic disease, and potential occupational exposure to chemicals, should be collected. Further testing for acute hepatitis A, B, C, D, and E infection, liver imaging (e.g., biliary tract), and collection of serum samples for acetaminophen/paracetamol drug and/or protein adduct levels may be warranted.

All cases demonstrated on repeat testing as meeting the laboratory criteria of AST/ALT and T bili elevation defined above should be considered potential DILI (Hy's law) cases if no other reason for the LFT abnormalities has yet been found. **Such potential DILI (Hy's law) cases are to be reported as SAEs,**

irrespective of availability of all the results of the investigations performed to determine etiology of the LFT abnormalities.

A potential DILI (Hy's law) case becomes a confirmed case only after all results of reasonable investigations have been received and have excluded an alternative etiology. A confirmed DILI should be captured as an ESI and reported as an SAE in the EDC.

Elevations of AST, ALT, and T bili that do not meet the laboratory threshold definitions above will not be captured as ESIs, but will be reported as AEs.

13.8 APPENDIX HALTING RULES

Dosing and enrollment will be temporarily suspended pending review of the DSMB if 3 or more participants experience an SAE of the same type that is determined to be related to study intervention(s). The DSMB recommendations will be considered by the NIH and the study Principal Investigator prior to making any decisions regarding appendix continuation or discontinuation.

A Platform Protocol for Evaluation of Interventions for Viral Persistence, Viral Reactivation, and Immune Dysregulation in Post-Acute Sequelae of SARS-CoV-2 Infection (PASC)

Protocol: RECOVER-VITAL

STATISTICAL ANALYSIS PLAN (SAP)

Protocol Version 4.0

Protocol Version Date: 02 NOV 2023

SAP Version Number: 1.0

13 FEB 2024

Kanecia Zimmerman, MD, PhD, MPH

Professor of Pediatrics, Pediatrics-Critical Care Med

Duke Clinical Research Institute

300 W. Morgan Street

Durham, NC 27701

Tel: 919-668-8651

E-mail: kanecia.zimmerman@duke.edu

SAP Approval Page

Study Title: RECOVER-VITAL: A Platform Protocol for Evaluation of Interventions for Viral Persistence, Viral Reactivation, and Immune Dysregulation in Post-Acute Sequelae of SARS-CoV-2 Infection (PASC)

Version: 1.0

Date of Issue: 13 FEB 2024

Study Sponsor: Kanecia Zimmerman, MD, PhD, MPH
Professor of Pediatrics, Pediatrics-Critical Care Med
Duke Clinical Research Institute
300 W. Morgan Street, Durham, NC 27701

We, the undersigned, have read and approve of this SAP and agree on its content.

Electronically signed by: Kanecia Zimmerman
Reason: Approved
Date: Feb 14, 2024 16:39 EST

Sponsor Representative – Kanecia Zimmerman, MD, PhD, MPH

Sean O'Brien
Electronically signed by: Sean O'Brien
Reason: Approved
Date: Feb 15, 2024 21:05 EST

RECOVER-VITAL Reviewing Statistician – Sean O'Brien, PhD

Lindsey Baden
Electronically signed by: Lindsey Baden
Reason: Approved
Date: Feb 16, 2024 06:17 EST

RECOVER-VITAL Co-Principal Investigator – Lindsey Baden, MD

Richard Whitley
Electronically signed by: Richard Whitley
Reason: Approved
Date: Feb 16, 2024 06:32 CST

RECOVER-VITAL Co-Principal Investigator – Richard Whitley, MD

TABLE OF CONTENTS

Table of Contents 3
List of Abbreviations 5
1 Study and Document Overview 7
2 Study Objectives and Endpoints 8
2.1 Study Objectives 8
2.2 Endpoints 8
3 Statistical design 11
3.1 Power and Sample Size 11
3.2 Randomization Methods 12
4 Study Assessments and Definitions 13
4.1 Study Assessments 13
4.2 Symptom Cluster Assignment 13
4.3 Definition of Baseline 15
4.4 Efficacy Assessments 16
5 General Analysis Considerations 21
5.1 Data Sources 21
5.2 Software 22
5.3 Verification of Results 22
5.4 Populations for Analysis 22
5.5 Multiple Testing 22
5.6 Covariate Adjustment and Subgroups 24
6 Summary of Study Data 25
6.1 Subject Disposition 25
6.2 Demographics and Medical History 26
6.3 Vital Signs 26
6.4 Baseline and Concomitant Medications 26
7 Interim Analyses and Data Monitoring 26
8 Efficacy Analyses 27
8.1 Primary Efficacy Analysis 27
8.2 Handling of Missing Data 29
8.3 Analysis of Secondary Endpoints 30
8.4 Analysis of Exploratory PRO and Performance Endpoints at Days 90 and 180 31
8.5 Analysis of Weekly Longitudinal PROs 33

8.6 Analysis of Heterogeneity of Treatment Effect (HTE) ... 33
8.7 Bayesian Analyses ... 35
9 Analysis Of Medical Visits And Hospitalizations ... 39
10 Safety analyses ... 39
10.1 Clinical Laboratory Data ... 40
11 Biomarker Analyses ... 40
12 ADHERENCE ... 41
13 References ... 41
14 Appendices ... 42
Appendix 1. RECOVER-VITAL Roster of Statisticians ... 42
Appendix 2. Sensitivity analyses for missing outcomes ... 44

LIST OF ABBREVIATIONS

ADaM	Analysis Data Model
AE	Adverse Event
ALT	Alanine transaminase
ANCOVA	Analysis of Covariance
AST	Aspartate transaminase
ATC	Anatomical Therapeutic Classification
BMI	Body Mass Index
CDISC	Clinical Data Interchange Standards Consortium
CI	Confidence Interval
COVID-19	Coronavirus Disease of 2019
CRF	Case Report Form
CRP	C-Reactive Protein
CTCSQ	Cluster Targeted COVID-19 Symptom Questions
DAIDS	Division of AIDS
DCRI	Duke Clinical Research Institute
DILI	Drug-Induced Liver Injury
DSMB	Data and Safety Monitoring Board
DSQ - PEM	DePaul Symptom Questionnaire Post Exertional Malaise
ECOG 2	Everyday Cognition 2
eCRF	Electronic Case Report Form
EOD	End of Dosing
ESI	Events of Special Interest
ESWT	Endurance Shuttle Walk Test
FU	Follow-up
HIV	Human Immunodeficiency Virus
ISWT	Incremental Shuttle Walk Test
LFT	Liver Function Test
LLN	Lower Limit of Normal
MedDRA	Medical Dictionary of Regulatory Activities
MITT	Modified Intention-To-Treat
OHQ	Orthostatic Hypotension Questionnaire
OHDAS	Orthostatic Hypotension Daily Activity Scale
OHSA	Orthostatic Hypotension Symptom Assessment
PASC	Post-acute Sequelae of SARS-CoV-2 Infection
PCR	Polymerase Chain Reaction
PEM	Post-Exertional Malaise
PGIC	Patient Global Impression of Change
PRO	Patient Reported Outcome
PT	Preferred Term
Q1	25th Percentile (1st Quartile)
Q3	75th Percentile (3rd Quartile)
RAT	Rapid Antigen Test
RECOVER	Researching COVID to Enhance Recovery
SAE	Serious Adverse Event

SAP	Statistical Analysis Plan
SAR	Suspected Adverse Reaction
SARS-CoV-2	Severe Acute Respiratory Syndrome Coronavirus 2
SD	Standard Deviation
SDTM	Study Data Tabulation Model
SE	Standard Error
SUSAR	Suspected Unexpected Serious Adverse Reaction
ULN	Upper Limit of Normal

1 STUDY AND DOCUMENT OVERVIEW

RECOVER-VITAL is a platform protocol designed to be flexible so that is suitable for a wide range of settings within health care systems and community settings where it can be integrated into COVID-19 programs and subsequent treatment plans. RECOVER-VITAL is a randomized, controlled, double-blinded, multi-center, multi-arm platform trial which will evaluate different interventions organized as appendices to the protocol. Each appendix will evaluate potential mechanisms of action, efficacy, and safety of antivirals and other therapeutics in individuals with Post-acute Sequelae of SARS-CoV-2 Infection (PASC), according to the platform protocol objectives.

This document describes the planned statistical analyses that will be conducted at the completion of the RECOVER-VITAL study for the first intervention appendix – Appendix A. Once the initial unblinded data review has occurred, only the blinded statistics team members will be allowed to modify the SAP (see Appendix 1 for the list of blinded and unblinded statisticians).

Each study intervention appendix will enroll adult participants who experience at least one of three predominant symptom clusters: cognitive dysfunction, autonomic dysfunction, or exercise intolerance with post-exertional malaise (PEM). Overall enrollment for the platform protocol will depend on the number of screen failures, the number of study intervention appendices that are added, the ability to pool their control groups for analysis, and adjustments to sample size based on study data. Enrollment will begin in July 2023 with Appendix A. Study intervention appendices may be added or removed according to adaptive design and/or emerging evidence.

This study intervention Appendix A is a prospective, multi-center, double-blind, randomized controlled phase 2 trial evaluating nirmatrelvir/ritonavir (PAXLOVID) in two dosing durations for PASC. Within this study, participants will be randomized 1:1:1 to 15 days of active intervention (nirmatrelvir/ritonavir) with 10 days of control (placebo/ritonavir), 25 days of active intervention, or 25 days of control, within the three symptom clusters. The estimated sample size is approximately 100 evaluable participants per treatment group per symptom cluster (900 total participants). The intervention will be administered in two kits (15 days followed by 10 days). For this appendix, the midpoint of drug dosing is approximately 13 days; the Drug Dosing Midpoint Clinic Visit will occur at Day 15 (-5 days).

Participants will be in the study for approximately 6 months from the time of initiating study drug administration until their end of study visit. At approximately the midpoint of drug dosing, a Drug Dosing Midpoint Clinic Visit will occur. The End of Dosing Clinic Visit will occur after the final intervention dose has been administered. The Follow-Up Period will begin after the End of Dosing Clinic Visit. A follow-up clinic visit will occur for all participants on Day 90 (± 5 days). All participants will receive remote follow-up approximately weekly with optional in-person visits for blood collection on Days 45 (± 5), 60 (± 5), and 120 (± 5) days. The End of Study Visit will be a remote visit on Day 183 (± 7 days).

Dosing and enrollment will be temporarily suspended pending review of the Data and Safety Monitoring Board (DSMB) if three or more participants experience a serious adverse event (SAE) of the same type that is determined to be related to study intervention(s). The DSMB recommendations will be considered by the NIH and the study Principal Investigator prior to making any decisions regarding appendix continuation or discontinuation.

2 STUDY OBJECTIVES AND ENDPOINTS

2.1 Study Objectives

2.1.1 Primary Objective:

- To evaluate the effect of intervention(s) versus control on symptom-specific patient-reported outcome (PRO) measures within each PASC symptom cluster at Day 90.

2.1.2 Secondary Objectives:

- To evaluate the effect of intervention(s) versus control on performance-based outcomes within each PASC symptom cluster.

2.1.3 Safety Objectives:

- To characterize the safety and tolerability of intervention(s) in PASC.

2.1.4 Exploratory Objectives:

- To investigate mechanisms of potential intervention(s) efficacy.
- To compare the effect of intervention(s) on symptom burden and health related quality of life versus control.
- To evaluate the durability of intervention(s) response.
- To evaluate intervention(s) effect on healthcare utilization.
- To describe the effect and durability of intervention(s) versus control across exploratory performance-based outcomes and PROs within each PASC symptom cluster.
- To determine biomarkers associated with PASC symptom clusters and intervention-associated recovery.

2.2 Endpoints

This section summarizes protocol-defined endpoints as listed in Protocol Table 1 and documents associated key additional pre-specified definitions and implementation details. Some differences between endpoint descriptions below versus the protocol are described in Section 2.2.4 below.

2.2.1 Primary Endpoints

The primary endpoint analysis will evaluate improvement in symptom-specific patient-reported outcome measures within each PASC symptom cluster at Day 90. There are 3 joint primary endpoints corresponding to 3 symptom clusters:

- Improvement in patient-reported cognition at Day 90: Increase by at least 5 T-score points on the PROMIS-cognitive 8a at Day 90 compared to baseline in the cognitive dysfunction symptom cluster. To reduce variability, an average of 3 weekly assessments closest to Day 90 will be used.
- Improvement in patient-reported orthostatic symptoms at Day 90: Decrease by at least 1 rating scale category on modified Orthostatic Hypotension Questionnaire (OHQ) Question #1 at Day 90 compared to baseline in the autonomic dysfunction symptom cluster. To reduce variability, an average of 3 weekly assessments closest to Day 90 will be used.

- Improvement in patient-reported post-exertional malaise (PEM) at Day 90: Absence of any symptoms of moderate or greater severity occurring with 50% or more frequency as assessed by a modified version of the DePaul Symptom Questionnaire Post-Exertional Malaise (DSQ-PEM) short form at Day 90 in the exercise intolerance symptom cluster.

For each pairwise comparison between an intervention and control group, the primary null hypothesis to be tested is that the probability of meeting a specified degree of improvement in symptom cluster-specific primary outcome measures (including the DSQ-PEM, PROMIS-cognitive 8a, or OHQ) at Day 90 compared to baseline is the same for intervention and control. The alternative hypothesis is that the probability of meeting a specified degree of improvement in symptom cluster-specific primary outcome measures at Day 90 compared to baseline differs for intervention and control.

2.2.2 Secondary Endpoints

- Improvement in cognitive performance outcome at Day 90: Increase by at least 1 standard deviation (SD) in either or both of the WHO/UCLA Auditory Verbal Learning Test (AVLT) delayed recall measure and/or Symbol Digit Modalities Test number of correct substitution measure, and no decrease exceeding 0.15 SD in either of the above measures, at Day 90 in the cognitive dysfunction symptom cluster
- Improvement in orthostatic performance outcome at Day 90: Improvement by a specified threshold in at least one of the following 3 measures from the active stand test and no worsening in any of the following 3 measures from the active stand test at Day 90 compared to baseline in the autonomic dysfunction symptom cluster:
    - 10-minute heart rate change
        - Improvement: Decrease by at least 10 beats per minute from baseline to Day 90
        - Worsening: Any increase from baseline to Day 90
    - 10-minute systolic blood pressure change
        - Improvement: Increase by at least 10mmHg from baseline to Day 90
        - Worsening: Any decrease from baseline to Day 90
    - 10-minute diastolic blood pressure change
        - Improvement: Increase by at least 5mmHg from baseline to Day 90
        - Worsening: Any decrease from baseline to Day 90
- Improvement in exercise performance outcome at Day 90: Increase by at least 3 minutes in walking time on the Endurance Shuttle Walk Test (ESWT) at Day 90 compared to baseline in the exercise intolerance symptom cluster.

The null hypothesis to be tested is that there is no difference between intervention and control group.

2.2.3 Exploratory and Safety Endpoints

Efficacy

- PROMIS cognitive 8a T-score as continuous variable at days 90, 180, and longitudinally in the cognitive dysfunction symptom cluster
- OHQ Question #1 as continuous variable at days 90, 180, and longitudinally in autonomic dysfunction symptom cluster
- DSQ-PEM as continuous outcome at days 90, 180, and longitudinally in exercise intolerance symptom cluster

- Neurocognitive battery outcomes (continuous) at day 90 in the cognitive dysfunction symptom cluster: AVLT total learning score, AVLT delayed recall score, AVLT delayed recognition score, SDMT number of correct substitutions
- Change in blood pressure and heart rate on active stand test at day 90 in the autonomic symptom cluster
- ESWT walk time as continuous variable at day 90 in the exercise intolerance symptom cluster
- Frequency and severity of individual symptoms at days 90 and 180 as assessed by PASC symptom questionnaire
- PROMIS-29+2 physical function summary score at days 90, 180 and longitudinally; other PROMIS-29+2 measures at days 90 and 180
- Patient global impression of change (PGIC) at days 90 and 180
- ECOG-2 Overall score at days 90 and 180 in cognitive dysfunction symptom cluster
- COMPASS-31 overall score and subscales at days 90 and 180 in the autonomic symptom cluster
- OHQ overall score and subscales at days 90 and 180 in the autonomic symptom cluster
- Endpoints derived from full autonomic testing at select sites (details to be determined)
- Endpoints derived from cardiopulmonary exercise testing at select sites (details to be determined)
- Healthcare utilization
    - Number of cumulative medical visits in intervention(s) vs. control
    - Number of cumulative hospitalizations in intervention(s) vs. control

Safety

- Occurrence of individual SAEs
- Occurrence of any one or more SAE
- Occurrence of adverse events (AEs) and SAEs leading to discontinuation
- Occurrence of events of special interest (ESIs)

Adherence

- Number of missed doses

Biomarkers

- Viral clearance, persistence, and recurrence
- Immune dysfunction
- Coagulation
- Biologic system functioning

2.2.4 Differences in Above List Compared to Protocol

- The protocol does not specify which summary scores are to be calculated and analyzed for outcomes such as PROMIS-29, COMPASS-31, OHQ, neurocognitive battery, and active stand test. Similarly, it does not specify criteria for "meeting pre-specified change from baseline to Day 90 on cluster-specific performance outcome" for the construction of performance-based secondary endpoints. These details have been added above.

- Protocol Table 1 only lists efficacy endpoints that are to be analyzed at Day 90 and end of study. The list above pre-specifies a set of endpoints that are also to be analyzed across multiple time points longitudinally.
- The SAP uses "day 180" as short-hand for the protocol-defined end of study assessment.

3 STATISTICAL DESIGN

3.1 Power and Sample Size

This study uses an adaptive platform trial design that will allow study interventions to be added or dropped from consideration based on accruing evidence of futility or efficacy. In such a design, the required sample size depends on both the number of interventions tested and the ability to pool their control arms for analysis. Initial sample size estimates are based on a single appendix with a 1:1:1 allocation to each of two dose levels of an active intervention and control. If additional study interventions are added later that can contribute to pooled control, the sample size will be adjusted accordingly.

The sample size is based on a design that will evaluate treatment effects separately within three co-primary symptom cluster populations (cognitive dysfunction, autonomic dysfunction, and exercise intolerance) with a Holm-type adjustment to control the overall type-I error across them. The Holm procedure will initially allocate alpha equally across symptom clusters (0.0166, 0.0166, 0.0166), with a total type-I error probability of 0.05. If the null hypothesis is initially rejected for some but not all symptom clusters, the Holm procedure will allow the non-rejected hypotheses to be re-tested at an updated, less stringent significance level.

Accounting for the Holm procedure's stepwise alpha selection in power calculations is complicated because the final significance threshold is partly random and depends on the unknown configuration of treatment effects across all 3 symptom clusters considered jointly. As a simple conservative approximation, power was calculated for each symptom cluster individually assuming a specified treatment effect for that single symptom cluster and assuming 0.0166 for the Holm procedure's final significance threshold.

Analysis within each symptom cluster population will control the type-I error across multiple dose levels by analyzing them in a pre-specified hierarchical testing procedure in order from highest to lowest dose. For simplicity, the total sample size was obtained by calculating sample size for a simple two-group comparison and multiplying by 3/2.

Sample size was chosen to provide 80% power to detect a difference of 25 percentage points in the proportion of participants who experience the binary primary outcome for each pairwise comparison between a treatment group and control within each symptom cluster, assuming a two-sided test with alpha = 0.0166. Specifically, a sample size of 82 per group yields 80% power assuming the true outcome probabilities are 37.5% in the control group and 62.5% in the active treatment group. This sample size was inflated by 1/0.82 = 1.22 to account for potential 18% dropout/loss to follow-up.

The final required sample size per symptom cluster was calculated as 82 x 1.22 = 100 participants per intervention group (300 total per symptom cluster). Enrollment within a symptom cluster will continue until the required sample size of 100 participants is met for each intervention group.

Table 1 illustrates the magnitude of treatment effects that will be detectable with 80% or 90% power under the study's planned sample size if the primary outcome occurs with probabilities ranging from 0.05 to 0.35 in the placebo group.

Table 1. Minimum treatment effects for 80% or 90% power

Probability of Improvement with Placebo	Minimum Treatment Effect Difference for 80% Power*	Minimum Treatment Effect Difference for 90% Power*
0.05	0.17 (0.22 vs 0.05)	0.20 (0.25 vs 0.05)
0.10	0.20 (0.30 vs 0.10)	0.23 (0.33 vs 0.10)
0.15	0.22 (0.37 vs 0.15)	0.25 (0.40 vs 0.15)
0.20	0.23 (0.43 vs 0.20)	0.27 (0.47 vs 0.20)
0.25	0.24 (0.49 vs 0.25)	0.28 (0.53 vs 0.25)
0.30	0.25 (0.55 vs 0.30)	0.28 (0.58 vs 0.30)
0.35	0.25 (0.60 vs 0.35)	0.28 (0.63 vs 0.35)

* Power for pairwise comparison between single dose level of active intervention versus control assuming 100 participants per treatment group (82 participants per treatment group with non-missing outcome data) and two-sided alpha = 0.0166.

3.2 Randomization Methods

A two-step randomization process will be used to achieve blinding and equitable randomization probability.

At the first step, each participant will be assigned with equal probability to one of the appendices for which the participant is eligible after applying any appendix-level exclusion criteria. At the second step, if the appendix includes k dose levels of active intervention plus a control group, participants will be assigned with probability $1/(k+1)$ to one of the $(k+1)$ available treatment options.

For simplicity, the text below assumes that M_1 active appendices will have no sharing of control participants and that an additional M_2 active appendices each involve a single intervention dose level and will use a single pooled control group among them. Let k_i be the number of dose levels in the i-th appendix where no restriction is placed on $k_1, \dots, k_{M_1}$and $k_{M_1+1} = k_{M_1+2} = \cdots = k_{M_1+M_2} = 1$. Randomization will proceed as follows for a participant who is eligible for m_1 of the M_1 appendices with no sharing of controls and m_2 of the M_2 appendices with sharing of controls. At the first stage, the participant will be assigned with probability $1/(m_1 + m_2)$ to one of the appendices for which the participant is eligible. If the randomly assigned intervention is unable to use a pooled control group, the second stage randomization will assign the participant with probability $1/(k_i + 1)$ to one of the available intervention groups, where i denotes the randomly assigned appendix. If the randomly assigned intervention is one that will use a pooled control group, the second stage randomization will assign the participant to active intervention or control at an m_2: 1 ratio, where m_2 is defined above.

Sites will be informed to which study intervention appendix the participant is randomized, but not whether they are allocated to one of the active intervention dose levels or control within that appendix. The participants and investigators will be blinded throughout the study.

4 STUDY ASSESSMENTS AND DEFINITIONS

4.1 Study Assessments

Participants will be followed for approximately 6 months from time of randomization and treatment initiation. Clinic visit assessments will occur for all participants at baseline and at approximately day 13 (window, 10-15), day 25 (end of dosing visit), and day 90 (primary outcome assessment). All participants will also receive remote follow-up approximately weekly with and will be offered optional in-person visits for blood collection on Days 45 (± 5), 60 (± 5), and 120 (± 5) days. A remote end of study visit will occur on day 183 (± 7 days). The collection schedule for selected key study data is summarized in Table 2.

Table 2. Abbreviated schedule of assessment (selected items)

Visit	Screen/ Baseline	Remote: Weekly Assessments	Clinic: Dosing Midpoint	Clinic: End of Dosing	Clinic: Primary Outcome	Remote: End of Study
Target day of visit	0	Weekly	15	25	90	183
Window (days)	-2 to 0	*	10 to 15	± 3	± 5	± 7
PROMIS 8a (cognitive)	x	x	x	x	x	x
ECOG2 (cognitive)	x	x	x	x	x	x
OHQ (autonomic)	x	x	x	x	x	x
COMPASS-31 (autonomic)	x	x	x	x	x	x
DSQ-PEM (exercise)	x	x	x	x	x	x
PROMIS-29+2	x	x	x	x	x	x
PASC symptom questionnaire	x	x	x	x	x	x
CTCSQ (including PGIC)	x			x	x	x
Neuro battery (cognitive)	x			x	x	
Active stand (autonomic)	x			x	x	
ESWT (exercise)	x			x	x	
Healthcare utilization			x	x	x	x
Blood/biomarkers**	x		x	x	x	
Safety	x		x	x	x	x
Adherence			x	x		

* All assessments that are completed remotely on a weekly basis will be open to the participant to complete for 5 days. Any assessments that are not completed within that window will expire and will not be collected.
** Not shown in table: optional clinic visits for optional blood collection on days 45 (± 5), 60 (± 5), and 120 (± 5).

4.2 Symptom Cluster Assignment

Assignment of participants into symptom clusters will be based on assessments performed at the screening and baseline visit(s). An indicator of eligibility for each symptom cluster will be derived programmatically within the EDC based on the data entered. Participants may be eligible for multiple symptom clusters and will be assigned to all clusters for which eligibility criteria are met. Participants who meet criteria for a cluster will undergo symptom cluster-specific outcome assessments and will be included in symptom cluster-specific primary and secondary endpoint analyses. Screening for symptom

cluster eligibility will begin with administration of the Cluster Targeted COVID-19 Symptom Questions (CTCSQ). As detailed below, the CTCSQ assesses presence and severity of twelve symptoms with response options of none, mild, moderate, or severe. Participants who report having at least two moderate symptoms from the same symptom cluster or one severe symptom from a symptom cluster will undergo additional assessments to determine eligibility for that cluster (see Figure 1).

Figure 1. Symptom cluster assignment flow

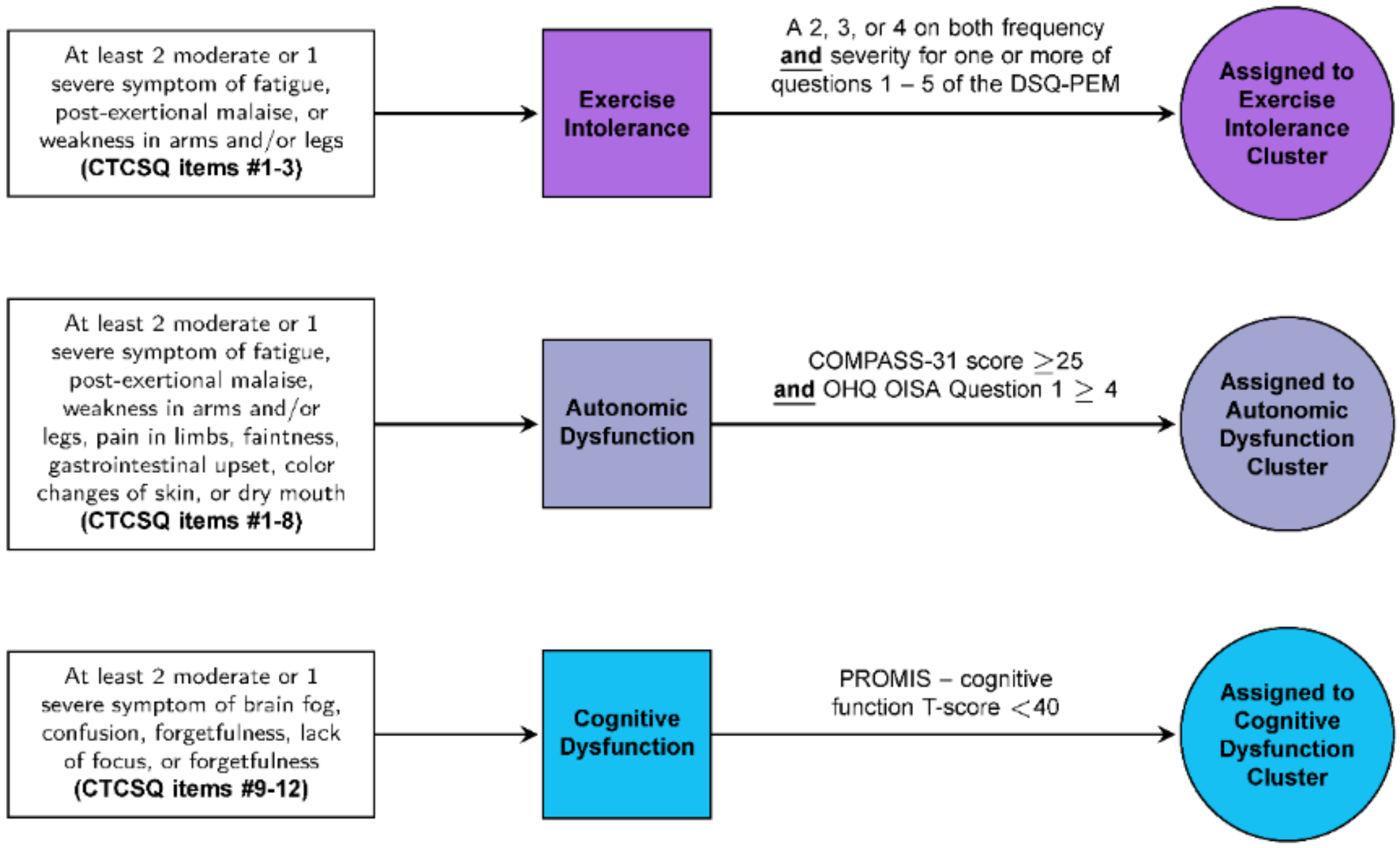

4.2.1 Cognitive dysfunction symptom cluster: eligibility

- At least two moderate or one severe rating within CTCSQ items 9-12
  - CTCSQ items 9-12: brain fog; confusion; forgetfulness; difficulty focusing
- PROMIS – cognitive 8a function T-score < 40

4.2.2 Autonomic dysfunction symptom cluster: eligibility

- At least two moderate or one severe rating within CTCSQ items 1-8
  - CTCSQ items 1-8: fatigue; post-exertional malaise; weakness in arms and/or legs; pain in limbs; faintness; gastrointestinal upset; color changes of the skin; dry mouth
- COMPASS 31 score ≥ 25
- OHQ OHSA Question #1 ≥ 4

4.2.3 Exercise intolerance symptom cluster: eligibility

- At least two moderate or one severe rating within CTCSQ items 1-3
  - CTCSQ items 1-3: fatigue; post-exertional malaise; weakness in arms and/or legs

- DSQ-PEM: at least one moderate or severe symptom occurring with at least 50% frequency

4.2.4 Cluster-Targeted COVID Symptom Questionnaire (CTCSQ)

The CTCSQ was created for RECOVER-VITAL as an eligibility screening tool for symptom clusters. The first twelve items of CTCSQ assess presence and severity of symptoms related to cognition, autonomic function, and exercise symptoms using response options of none, mild, moderate, or severe:

1. Fatigue (being tired)
2. Post-exertional malaise (symptoms worse after even minor physical or mental effort)
3. Weakness in your arms and/or legs
4. Pain in your limbs
5. Faintness (light-headedness)
6. Gastrointestinal upset
7. Color changes of the skin
8. Dry mouth
9. Problems with thinking or concentrating (brain fog)
10. Confusion
11. Forgetfulness
12. Difficulty focusing

If a participant reports a moderate or severe item #1-3, they will complete the exercise intolerance and autonomic dysfunction cluster assessments at Baseline. If a participant reports a moderate or severe item #1-8, they will complete the autonomic dysfunction cluster assessments at Baseline. If a participant reports a moderate or severe item #9-12, they will complete the cognitive dysfunction cluster assessments at Baseline.

In addition to assessing symptom cluster-specific eligibility, the above items will be used descriptively to track changes in symptoms during follow-up. After randomization, CTCSQ will be performed at the end of dosing visit, day 90 visit, and end of study visit.

The final CTCSQ item (#13) is the Patient Global Impression of Change (PCIG). The purpose of this item is fundamentally different than the others and is described in a separate section below. It is not associated with specific symptom clusters and does not play a role in symptom cluster assignment.

4.3 Definition of Baseline

Baseline assessments will preferably occur on the same day as the first study dose administration (i.e., the day of randomization) and may occur on the same day as screening. In all cases, randomization and administration of first study dose are expected to occur within 48 hours of baseline assessments. Baseline concomitant medications will include all concomitant therapies taken by the participant within 14 days prior to informed consent. Data collected at baseline will include, among others:

- Demographics
- Height and weight
- Blood pressure and pulse, supine and standing

- Medical History; including SARS-CoV-2 vaccination status and dates, SARS-CoV-2 test result date (if available), signs and symptoms, and treatment (including hospitalization, Intensive Care Unit (ICU) status, supplemental O2 status) and PASC history (symptoms and duration)
- Medications taken within 14 days prior to informed consent
- Pre-treatment values of efficacy variables (PRO and performance measures) and safety variables (labs)
- Blood and stool collection for mechanistic evaluation

4.4 Efficacy Assessments

4.4.1 PROMIS Cognitive 8a

PROMIS Cognitive 8a is a questionnaire assessing self-reported cognitive impairments over the past 7 days using 8 items. It assesses the frequency that respondents experienced the following impairments on a scale ranging from 5 (never) to 1 (very often; several times a day) for the following items:

- PC2r. My thinking has been slow
- PC35r. It has seemed like my brain was not working as well as usual
- PC36r. I have had to work harder than usual to keep track of what I was doing
- PC42r. I have had trouble shifting back and forth between different activities that require thinking
- PC8r. I have had trouble concentrating
- PC25r. I have had to work really hard to pay attention or I would make a mistake
- PC1r. I have had trouble forming thoughts
- PC5r. I have had trouble adding or subtracting numbers in my head

The total raw score is transformed into a T-score, with higher scores representing better cognitive function. The T scores are interpreted in relation to a US reference population and are scaled to have mean = 50 and SD = 10 in the reference population.

The PROMIS-cognitive 8a will be performed as part of screening for the cognitive dysfunction symptom cluster. For participants who qualify for the cognitive dysfunction cluster, it will be collected at baseline, remotely on a weekly basis from randomization until end of study, and at in-person follow-up clinic visits.

The primary endpoint for the cognitive dysfunction symptom cluster is improvement of at least 5 T-score points on the PROMIS-cognitive 8a as measured at Day 90 compared to baseline. The 5-point threshold is based on a body of literature that has specifically evaluated the minimally important difference using distribution- and anchor-based approaches across a number of disease processes (e.g., cancer, stroke, low back pain, osteoarthritis) and PROMIS measures (e.g., pain intensity, depression, physical functioning, anxiety, fatigue, etc.). These varied populations consistently report a minimally important difference of 2 to 6. [1-3] Moreover, a value of 5 represents one-half the standard deviation for PROMIS measures in the US general population and has been recommended as a starting point when no empirical data are available. [4,5]

4.4.2 Modified Orthostatic Hypotension Questionnaire (OHQ)

The Orthostatic Hypotension Questionnaire (OHQ) is a patient reported outcome designed to assess the severity and impact of orthostatic hypotension (OH), a condition characterized by a sudden drop in

blood pressure when standing. The OHQ consists of two main components: the Orthostatic Hypotension Symptom Assessment (OHSA) and the Orthostatic Hypotension Daily Activity Scale (OHDAS). The OHSA consists of 6 items measuring severity on a scale ranging from 0 (none) to 10 (worst possible) for the following symptoms:

1. Dizziness, lightheadedness, feeling faint, or feeling like you might black out.
2. Problems with vision (blurring, seeing spots, tunnel vision, etc.)
3. Weakness.
4. Fatigue.
5. Trouble concentrating.
6. Head and neck discomfort.

The OHDAS assesses the extent to which OH interferes with daily life on a scale ranging from 0 no interference) to 10 (total interference) for each of the following activities:

7. Activities that require standing for a short time.
8. Activities that require standing for a long time.
9. Activities that require walking for a short time.
10. Activities that require walking for a long time.

The OHQ questionnaire for RECOVER-VITAL has been modified slightly compared to the original OHQ. The instruction wording was changed to refer to "orthostatic intolerance" instead of "orthostatic hypotension" and "low blood pressure".

The OHQ will be performed as part of screening for the autonomic dysfunction symptom cluster. For participants who qualify for the autonomic dysfunction symptom cluster, it will be collected at baseline, remotely on a weekly basis from randomization until end of study, and at in-person follow-up clinic visits.

The primary endpoint for the autonomic dysfunction symptom cluster is improvement as defined by at least a 1-point decrease in the response to OHQ question 1 at Day 90 compared to baseline. At least 1-point change on question 1 has been determined to be clinically meaningful and resulted in previous updates to the FDA drug label for orthostatic hypotension associated with Parkinson’s disease.

Other summary measures for OHSA, OHDAS, and overall OHQ are calculated by averaging across items within OHSA, OHDAS, and overall respectively. Scores range from 0 to 10 with higher values indicating worse impairment.

Slightly different scoring rules will be used in the construction of summary measures at baseline compared to follow-up assessments. At baseline, the baseline OHSA composite score will be calculated as an average of items 1-6 and the baseline OHDAS composite score will be calculated as an average of items 7-10. The baseline overall OHQ composite score will be an average of the baseline OHSA and baseline OHDAS composites.

At follow-up, the above scoring rules will be modified by only averaging across items that were non-zero at baseline. Thus, the follow-up the follow-up OHSA composite score will be calculated by averaging across the subset of items 1-6 that were non-zero at baseline. The follow-up OHDAS composite score will be calculated by averaging across the subset of items 7-10 that were non-zero at baseline. The

follow-up overall OHQ composite score will be an average of the follow-up OHSA and follow-up OHDAS composites.

4.4.3 Modified DePaul Symptom Questionnaire Post Exertional Malaise (DSQ-PEM)

The DePaul Symptom Questionnaire Post Exertional Malaise (DSQ-PEM) is a patient-reported outcome designed to assess the frequency and severity of symptoms worsening after physical or mental exertion. The first 10 items of DSQ-PEM assess frequency and severity of the following 5 exercise-related impairments:

1. Dead, heavy feeling after starting to exercise,
2. Next day soreness or fatigue after non-strenuous, everyday activities
3. Mentally tired after the slightest effort
4. Minimum exercise makes you physically tired
5. Physically drained or sick after mild activity.

These items were modified for the current study to use a 7-day instead of 6-month look back period. Frequency is rated on a 5-point Likert scale: 0 = none of the time, 1 = a little of the time, 2 = about half the time, 3 = most of the time, and 4 = all of the time. Severity is also rated on a 5-point Likert scale: 0 = symptom not present, 1 = mild, 2 = moderate, 3 = severe, 4 = very severe.

Based on recommended scoring rules, Post-Exertional Malaise (PEM) will be deemed to be present for participants reporting at least one moderate (rated severity ≥ 2) PEM symptom for a frequency rated ≥ 2.

The DSQ-PEM will be performed as part of screening for the exercise intolerance symptom cluster. For participants who qualify for the exercise intolerance cluster, the DSQ-PEM will be assessed at baseline, remotely on a weekly basis from randomization until end of study, at in-person visits, and 1 to 2 days after each visit where performance outcomes are collected. For all other participants, it will be collected 1 to 2 days after each visit where performance outcomes are collected.

The primary endpoint for the exercise symptom cluster is improvement in PEM, defined as having no symptoms of moderate or greater severity with 50% or more frequency as determined by the DSQ-PEM short form at Day 90.

The PEM measure defined above is binary. An analogous continuous measure will be obtained by counting the number of symptoms for which PEM criteria are met, i.e., the number of symptoms with at least moderate severity occurring with at least 50% frequency.

4.4.4 Modified Everyday Cognition 2 (ECOG 2)

The Everyday Cognition 2 (ECOG 2) is a self-report, 41-item questionnaire used to measure a participant's perceived capacity to perform activities related to cognitive function, which could impact major activities of daily living and independence. It has been used for patients with mild cognitive impairment, Alzheimer's Disease, and dementia. Assessments are organized within 7 domains corresponding to memory (9 items), language (9 items), visual-spatial and perceptual Abilities (8 items), executive function – planning (5 items), executive function – organization (6 items), and executive function – divided attention (4). The original ECOG-2 asks respondents to assess abilities now compared

to 10 years ago on a scale ranging from 1 (better or no change) to 4 (consistently much worse), with an additional response option for "don't know or not applicable".

For the current study, the questionnaire has been modified to use a 7-day look back and to assess current status without reference to a specific baseline. Response options range from 1 (no difficulty) to 5 (great difficulty/unable to do), with a separate option for "not applicable". For items that are applicable, participants are asked to indicate whether this is different compared to before COVID. The final ECOG 2 summary score is calculated as an average across all questionnaire items that are marked as "applicable".

The ECOG 2 will be collected for participants in the cognitive dysfunction symptom cluster. It will be assessed at baseline, remotely on a weekly basis from randomization until end of study, and at in-person follow-up clinic visits.

4.4.5 The Composite Autonomic Symptom Score 31 (COMPASS-31)

COMPASS-31 is a patient-reported instrument designed to evaluate the presence and severity of autonomic symptoms. It encompasses six domains, including orthostatic intolerance, vasomotor, secretomotor, gastrointestinal, bladder, and pupillomotor. Each domain has a set of questions that are scored on a five-point scale, and the final score is obtained by summing weighted scores of all domains. The total score ranges from 0 to 100, with higher scores indicating a higher severity of autonomic symptoms.

COMPASS-31 will be performed as part of screening for the autonomic dysfunction symptom cluster. For participants who qualify for the autonomic dysfunction symptom cluster, it will be collected after randomization at in-person visits and remotely on a weekly basis from randomization until end of study.

4.4.6 PROMIS-29+2 Profile v2.1.

The PROMIS-29+2 Profile v2.1 is a patient-reported outcome measure designed to assess overall health and functioning. It includes 4 items per domain for each of 7 domains (physical function, anxiety, depression, fatigue, sleep disturbance, ability to participate in social roles and activities, pain interference), plus an item for pain intensity and 2 items for cognitive function. Pain intensity is assessed on scale ranging from 0 (no pain) to 10 (worst pain imaginable). All other items use Likert-type 5-point scales. Scoring of the PROMIS-29+2 will follow established guidelines [6]. Domain-specific summary scores will be expressed as T-scores with higher scores representing a worse condition for domains measuring impairment (e.g., anxiety, depression, fatigue, sleep disturbance, and pain interference) and representing a better condition for domains measuring function (e.g., physical function, ability to participate in social roles and activities). T scores are interpreted in relation to a US general reference population with population mean = 50 and SD = 10. An overall summary of health related quality of life will be derived from the PROMIS-29+2 by calculating the PROMIS Preference Score (PROPr). The PROPr summarizes results across all PROMIS-29+2 domains on a scale ranging from 0 (as bad as dead) to 1 (perfect or ideal health).

The PROMIS-29+2 Profile v2.1 will be collected at baseline, remotely on a weekly basis from randomization until end of study, and at in-person follow-up clinic visits.

4.4.7 Patient Global Impression of Change (PGIC)

The PGIC asks participants to assess how the overall severity of their long COVID symptoms have changed compared to the start of the study. The question wording is: "Compared to the start of the

study, the overall severity of your Long COVID symptoms have___". Response choices are: (5) Improved a lot, (4) Improved somewhat, (3) Stayed about the same, (2) Worsened somewhat, (1) Worsened a lot.

The PGIC has been incorporated into the CTCSQ and appears as Question #13 on the CTCSQ form. It will be collected at baseline as part of the screening CTCSQ, at the end of dosing visit, day 90 visit, and end of study visit.

4.4.8 PASC Symptom Questionnaire

The PASC Symptom Questionnaire assesses the frequency and severity of 33 individual symptoms. Frequency is assessed on Likert-type scale ranging from 1 (never) to 5 (almost constantly). Severity is assessed on a 4-level scale ranging from 1 (mild) to 4 (very severe). It will be collected at baseline, remotely on a weekly basis from randomization until end of study, and at in-person follow-up clinic visits.

4.4.9 Neurocognitive battery (AVLT/SDMT)

The Neurocognitive battery is a performance measure used to assess various elements related to cognition. The neurocognitive battery consists of a cognitive assessment sequence of the following: WHO/UCLA Auditory Verbal Learning Test (WHO/UCLA AVLT) and Symbol Digit Modalities Test (SDMT).

Three AVLT scores will be calculated:

- Total learning score: calculated as the total sum of correct responses from tests #1 through #5
- Delayed recall score: calculated as the number of correct responses from test #8
- Delayed recognition score: calculated as the number of correct recognitions minus false positives from test #9.

One SDMT score will be calculated:

- Number of correct substitutions.

The neurocognitive battery will be performed for participants in the cognitive dysfunction symptom cluster at baseline, end of dosing visit, and day 90. For analysis, the above scores will be converted into Z-scores by subtracting the overall study mean and dividing by the SD. The major secondary endpoint for the cognitive dysfunction symptom cluster is a binary endpoint defined as an increase by at least 1 SD in either or both of the AVLT delayed recall score and/or SDMT number of correct substitutions, and no decrease exceeding 0.15 SD in either of the above measures, at Day 90.

4.4.10 Active Stand Test

The active stand test is performed to assess presence of orthostatic intolerance, orthostatic hypotension, and postural orthostatic tachycardia syndrome. Participants are asked to lie down for 5 minutes and then stand for 10 minutes. The participant’s blood pressure and heart rate are recorded after 5 minutes of lying and then at minutes 1, 3, 5, and 10 after standing. The Vanderbilt Orthostatic Symptom Score, a 9 point scale of orthostatic symptoms rated on a scale of 0 (no symptom) to 10 (worst the participant has experienced), will also be obtained after 5 minutes of lying and then at 10 minutes after standing. Changes in systolic blood pressure (SBP), diastolic blood pressure (DBP), and heart rate beats per minute (HR BPM) will be assessed from lying to standing for 10 minutes. The main summary measures will be changes assessed at 10 minutes as follows:

- ΔHR = (HR BPM at 10 minutes standing) minus (HR BPM at 5 minutes lying)

- ΔSBP = (SBP at 10 minutes standing) minus (SBP at 5 minutes lying)
- ΔDBP = (DBP at 10 minutes) minus (DBP at 5 minutes lying).

The active stand test will be performed among participants in the autonomic dysfunction cluster at baseline, end of dosing visit, and Day 90.

Trial endpoints will be constructed by comparing values of ΔHR, ΔDBP, and ΔDBP at follow-up compared to baseline, i.e., by calculating differences in the above differences. The major secondary endpoint for the autonomic dysfunction cluster is a binary endpoint defined as improvement in at least one of ΔHR, ΔDBP, or ΔDBP from baseline to Day 90 (defined as an increase of at least 10mmHg in ΔSBP, an increase of at least 5mmHg on ΔDBP, or a decrease of at least 10 BPM on ΔHR) and no worsening in any of ΔHR, ΔDBP, or ΔDBP from baseline to Day 90 (defined as any decrease in ΔSBP or ΔDBP, or any increase in ΔHR).

4.4.11 Endurance Shuttle Walk Test (ESWT).

The ESWT consists of timed walking on a 10 meter course. In order to determine walking speed for the ESWT, it will be performed in combination with the incremental shuttle walk test (ISWT) at baseline. Walking speed for ESWT at baseline and follow-up will be 85% of the maximum walking speed achieved on the ISWT at baseline. The final ESWT result will be expressed as total walking time after an initial 2-minute warm-up. The ESWT will be performed at baseline, end of dosing visit, and Day 90. The major secondary endpoint for the exercise intolerance symptom cluster is an increase of at least 3 minutes in walk time at Day 90 compared to baseline.

The ESWT will be performed for participants in the exercise intolerance cluster at baseline, end of dosing visit, and Day 90.

4.4.12 Blood for clinical and mechanistic assessments

Blood for clinical laboratory assessments and mechanistic evaluation will be performed at baseline, mid-dosing visit, end of dosing visit, and day 90 visit. Optional blood draws will occur at optional visits around days 45, 60, and 120. For baseline clinical laboratory assessments, prior assessments within 3 months of study enrollment may be used. Antigenemia will be defined as detection of SARS-CoV-2 spike protein as determined by an external core lab using the Quanterix Simoa platform.

5 GENERAL ANALYSIS CONSIDERATIONS

5.1 Data Sources

At each study site, data will be entered on the electronic Case Report Forms (eCRFs) stored in Medidata Rave. Biomarker data will be collected by NIH-selected laboratories and will be integrated with the clinical database. Prior to database lock, programmed computer edit checks will be run against the database to identify discrepancies and verify reasonableness of the data. Queries to resolve discrepancies will be generated and resolved by the sites. As needed, DCRI Statistics will be able to directly download the eCRF database from Rave using SAS on Demand as Clinical Data Interchange Standards Consortium Study Data Tabulation Model (CDISC SDTM version 3.2) datasets. CDISC Analysis Data Model (ADaM version 2.1) datasets will be created by DCRI Statistics for production of final tables, figures, and listings. All planned reporting will be based off of CDISC datasets, but in the case of emergent safety data, some reporting may occur from the raw eCRF data. All programs written to create analysis datasets and perform analyses will be validated according to SOPs established by the DCRI Statistical Programming group.

5.2 Software

The statistical analyses described in this SAP, as well as production of tables, listings, and figures will be performed using SAS®, version 9.4 or higher (SAS Institute, Cary, NC). Additional statistical software may be used as needed.

5.3 Verification of Results

All tables, listings, and graphs will be verified and reviewed before considered final. The verification process will ensure that the numbers are produced by a statistically valid method and that the execution of the computations is correct. Qualified statisticians or statistical programmers employed by the DCRI who have not been previously involved in the production of the original programming will perform the verification procedures. Methods of verification include independent programming, prior to issuance of the draft statistical report, of all analysis datasets/ADaM and comparison to data listings. Tables, listings, and figures will be reviewed for accuracy, consistency with this analysis plan, consistency within tables/listings/figures, and consistency with corresponding output. Once verification is complete, all documentation of the verification process will be filed as required by the Statistical Standard Operations Procedures of the DCRI.

5.4 Populations for Analysis

The primary analysis will be based on a modified intent-to-treat population (mITT), consisting of all randomized participants who receive at least part of one dose of the study intervention or control. Participants will be analyzed according to their randomly assigned intervention group. A separate mITT population will be defined for each pairwise comparison between an active intervention and control within each symptom cluster population. Participants who were enrolled into > 1 symptom cluster, as allowed under Protocol Version 3.0, will be included in the mITT population for each cluster for which inclusion criteria are met.

Safety analyses will be based on a safety population consisting of all randomized participants who receive at least part of one dose of the study intervention or control. Participants who are randomized and treated despite being ineligible for all 3 symptom clusters will be included in safety analyses.

5.5 Multiple Testing

5.5.1 Primary Endpoints

A sequential closed testing procedure will be used to control the type-I error probability at 0.05 across primary endpoint analyses performed for each combination of 2 dose durations and 3 symptom clusters. Analyses for the two dose durations will be performed sequentially. The first set of comparisons will be between the 25-day dose duration group and control. As detailed below, a Holm-type multiplicity adjustment will be used to control the type-I error probability at 0.05 across the 3 symptom cluster-specific primary outcomes. If all 3 comparisons are significant, then testing of primary outcomes will proceed to 15-day dose duration. Otherwise, testing will stop and all 15-day analyses will be regarded as exploratory.

Figure 2 illustrates the trial's closed testing procedure for the primary outcomes. It is intended to control the type-I error probability at 0.05 across primary endpoint analyses performed for each combination of 2 dose durations and 3 symptom clusters). There are 6 primary endpoint hypotheses to be tested. They are denoted as $\{H_{11}, H_{12}, H_{13}, H_{21}, H_{22}, H_{23}\}$, where H_{dc} corresponds to dose duration d (1=25 days,

2=15 days) and symptom cluster c (1=cognitive, 2=autonomic, 3=exercise). Hypotheses are grouped into a family $\mathcal{F}_1 = \{H_{11}, H_{12}, H_{13}\}$ for the 25-day dose duration and a family $\mathcal{F}_2 = \{H_{21}, H_{22}, H_{23}\}$ for the 15-day dose duration. Testing will begin with $\mathcal{F}_1$ (25-day) and will only proceed to $\mathcal{F}_2$ (15-day) in the event that all hypotheses within $\mathcal{F}_1$ are rejected. The procedure will initially assign two-sided local significance level α/3 (≈ 0.01667) to each member of $\mathcal{F}_1$. If a hypothesis within $\mathcal{F}_1$ is rejected, its local alpha will be reallocated equally among the remaining hypotheses within $\mathcal{F}_1$ (i.e., a Holm-type multiplicity procedure). In the event that all 3 hypotheses within $\mathcal{F}_1$ have been rejected, testing will proceed to $\mathcal{F}_2$, with alpha reallocated equally among its 3 hypotheses.

Figure 2. Multiple testing flow diagram

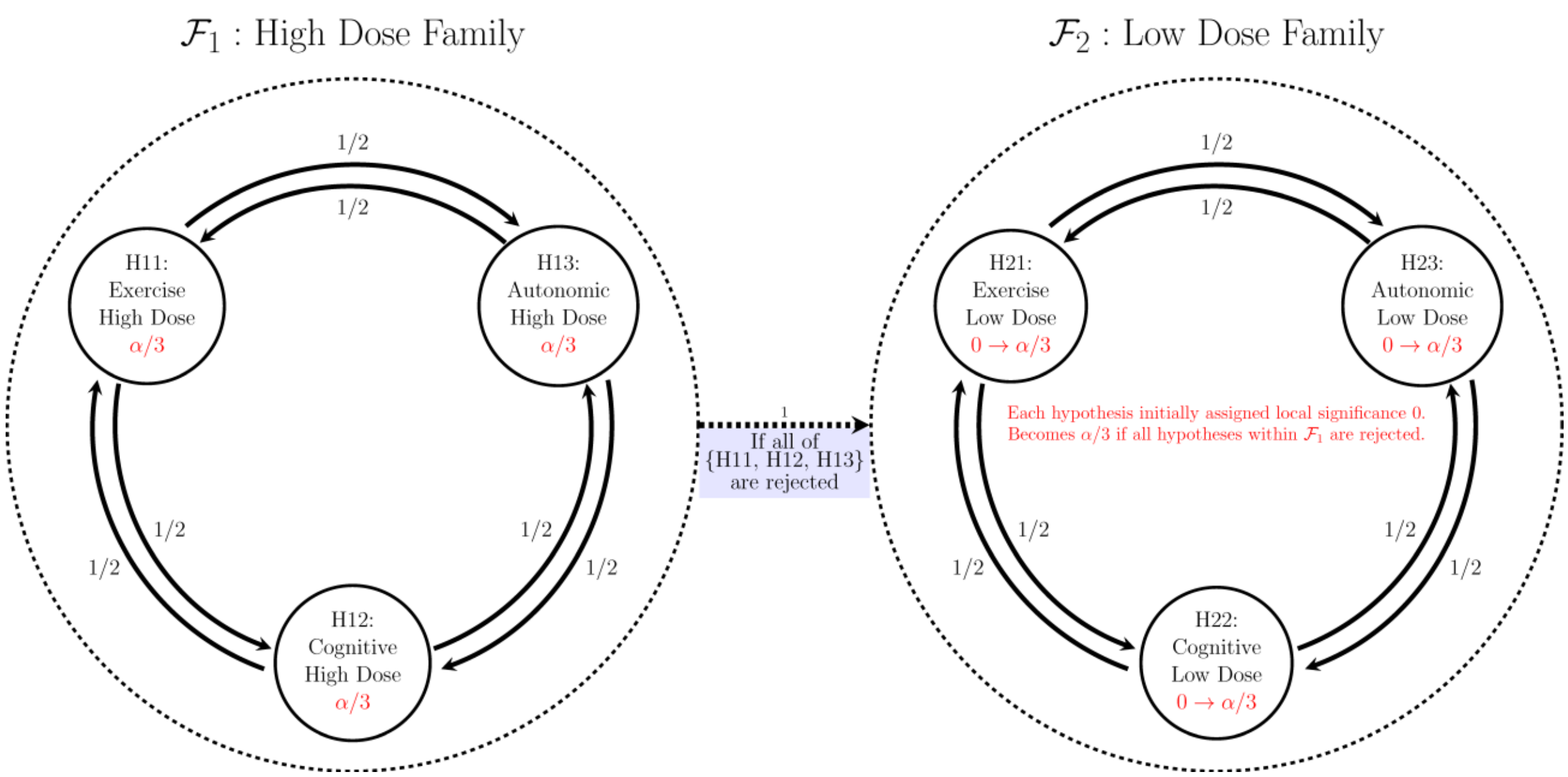

5.5.2 Secondary Endpoints

The multiplicity procedure described above provides strong type-I error control for the primary endpoints. For secondary endpoints, a less conservative approach will be adopted. For each symptom cluster and dose duration, if the hypothesis pertaining to the primary endpoint is rejected, then the symptom cluster's major secondary endpoint will be tested at the same dose duration using alpha = 0.05. This does not strictly control type-I error across all primary and secondary outcomes, but does offer some protection by using the primary outcome as a gatekeeper for secondary outcomes within each symptom cluster and dose duration. This less conservative approach is motivated by viewing the study design as a separate trial for each symptom cluster population and recognizing that a subsequent Phase 3 trial will be required for any definitive efficacy conclusions. Of note, a finding of benefit in a particular symptom cluster population will only pertain to that symptom cluster population and will not be extrapolated to other symptom cluster populations.

5.5.3 Exploratory Endpoints

No adjustment will be made for multiplicity in the analysis of exploratory endpoints.

5.6 Covariate Adjustment and Subgroups

5.6.1 Covariate adjustment and selection considerations

To account for chance covariate imbalances, all major efficacy analyses will be adjusted for pre-specified baseline covariates. Such adjustment is consistent with recommendations in two recent FDA guidance documents:

FDA's May 2021 guidance on adjusting for covariates in randomized clinical trials for drugs and biological products: "Although an unadjusted analysis is acceptable for the primary analysis, adjustment for baseline covariates will generally reduce the variability of estimation of treatment effects and thus lead to narrower confidence intervals and more powerful hypothesis testing." [7]

FDA's February 2021 guidance on developing drugs and biological products for treatment or prevention of COVID-19: "To improve the precision of treatment effect estimation and inference, sponsors should consider adjusting for prespecified prognostic baseline covariates (e.g., age, baseline severity, comorbidities, baseline medications and COVID-19 vaccination status) in the primary efficacy analysis and should propose methods of covariate adjustment. For example, for a binary endpoint, methods can be used to gain precision in the evaluation of the difference in proportions." [8]

Importantly, covariate adjustment can be implemented in a way that makes it robust to misspecification of the underlying regression models. [7] In other words, the calculated estimates and confidence intervals will not depend on correct model specification in order to be valid.

The main motivation for implementing covariate adjustment in this study protocol's efficacy assessments is to improve estimation efficiency, as discussed in FDA guidance documents. As such, pre-specified covariates were selected due to their suspected strong association with study outcomes.

An additional factor motivating covariate adjustment was a concern raised by the DMSB about potential for chance imbalances in site across treatment group. Such chance imbalances are possible because there is no stratification by site in the randomization procedure. As such, additional pre-specified covariates were selected based on their suspected strong associations with site and their potential association with study outcomes.

A final additional factor motivating covariate adjustment was the desire to potentially reduce bias that may result from missing outcome data. If analyses are not covariate-adjusted and missing outcomes occur, then treatment effect estimates will generally only be unbiased if the outcomes satisfy the condition of being missing completely at random (MCAR). If instead the efficacy analysis is covariate adjusted, then unbiased estimation can potentially be achieved if outcomes satisfy the relatively weaker condition of being missing at random (MAR), after adjusting for baseline covariates. While both assumptions (MCAR and MAR) are untestable and neither is likely to be literally true, the MAR assumption is a relatively weaker condition and therefore more likely to generate accurate estimates under a wider range of missing data scenarios. Sensitivity analyses with a range of adjustment methods are discussed in Section 8.2 and Appendix 2.

5.6.2 Pre-selected Covariates

Different pre-specified covariates will be used in different analyses for different endpoints. They are documented in relevant parts of Section 8.1.

5.6.3 Subgroups

The following subgroups have been pre-specified for exploratory analyses of treatment effect heterogeneity.

Primary interest

- Antigenemia positive at baseline (yes, no)
- Vaccination status at time of first acute COVID-19 infection (≥1 dose completed, 0 doses completed)
- Time since first acute COVID-19 infection (grouped by approximate tertiles)

Other

- Race
- Ethnicity
- Sex
- Acute COVID severity

The above designation of "primary interest" versus "other" does not appear in the protocol. It was adopted based on discussion with study principal investigators and pre-specified before first patient enrollment. The list of pre-specified subgroups was intentionally limited in order to reduce potential for spurious findings and to prioritize variables with a plausible mechanism to interact with treatment.

6 SUMMARY OF STUDY DATA

Continuous variables will be presented as n (with number of non-missing observations), mean, standard deviation, median, Q1, Q3, and minimum and maximum. Binary and categorical variables will be presented as counts and percentages (among non-missing values).

In general, summary tables will be structured with a column for each treatment group (25-day dose duration, 15-day dose duration, control) and will be annotated with the total population size relevant to that table. Summary tables will be constructed for the overall trial cohort and for each symptom cluster.

6.1 Subject Disposition

The disposition of subjects (number randomized, number who received any amount of the randomly assigned treatment, number completing study drug administration, number who withdrew consent or discontinued from study drug early, and number lost to follow-up, and number who completed the trial) will be summarized by treatment group. The number of subjects screened for inclusion and a breakdown of reasons for exclusion will be summarized. The timing and reasons for early discontinuation of study drug and/or withdrawal from the study will be summarized by treatment group. For the calculation of percentages, subjects who die will not be included in the denominators for visits/assessments beyond their death. A subject listing of analysis population eligibilities will be generated. A listing of all subjects discontinued from the study after enrollment, broken down by site and treatment group will be provided. The listing will include: reason for discontinuation, treatment group, duration of treatment, and whether or not the blind was broken. Also, for subjects who discontinued from the study after enrollment, a listing of adverse events will be provided. Subject-

specific protocol deviations will be summarized by the reason for the deviation, the deviation category, treatment group, and site for all subjects. All subject-specific protocol deviations and non-subject specific protocol deviations will be included in listings.

6.2 Demographics and Medical History

Baseline demographics and medical history will be summarized by intervention dose level within symptom cluster and overall by assigned treatment group. For continuous measures the mean and standard deviation will be summarized. Categorical variables will be described by the proportion in each category (with the corresponding sample size). Baseline characteristics will include age, sex, race, ethnicity, education level, height, weight, blood pressure, pulse, baseline SARS-CoV-2 vaccination status, signs and symptoms, treatment (including hospitalization, ICU status, and supplemental O2 status), and PASC history (symptoms and duration).

6.3 Vital Signs

Vital sign measurements include blood pressure and pulse (supine and standing).

6.4 Baseline and Concomitant Medications

Concomitant medications will be coded to the Anatomical Therapeutic Classification (ATC) using the WHO Drug Dictionary. The use of prior, concomitant medications taken, and changes in concomitant medications during the study will be recorded on the CRFs. Baseline concomitant medications will include all concomitant therapies taken by the participant within 14 days prior to informed consent. Concomitant medications will be reviewed through the End of Study. The use of concomitant medications during the study will be summarized by coded term and treatment group.

7 INTERIM ANALYSES AND DATA MONITORING

Interim examination of clinical endpoints and key safety events will be performed at regular intervals during the course of each intervention appendix. An independent, NIH-appointed, DSMB will monitor participant safety and review participant enrollment and performance of the trial. The primary objective of these interim analyses will be to ensure the safety of the participants enrolled in the trial and evaluate the accumulating endpoint data by treatment group. In addition, interim monitoring will involve a review of participant recruitment, compliance with the study protocol, status of data collection, and other factors which reflect the overall progress and integrity of the study. A complete description of the interim analyses plans with mock tables, listings, and figures is included in a separate DSMB guidance document.

There are no planned early stopping rules for efficacy in this protocol. Because PASC presentations and outcomes are highly varied, an important study objective is to estimate the effect of treatment on a wide range of participant-relevant outcomes. If the study were to be stopped early with less than the full sample size, it would decrease precision and reduce the study's ability to characterize treatment risks and benefits based on important secondary effectiveness and safety outcomes. It would also limit the collection of data that are critical for planning future trials in similar patient populations.

Early stopping for rules for futility may be incorporated on an intervention appendix specific basis. Such rules will be documented in the intervention appendices. There are currently no planned futility analyses for the Paxlovid appendix.

8 EFFICACY ANALYSES

8.1 Primary Efficacy Analysis

Each of the three symptom cluster-specific primary endpoints is a binary indicator of meeting a pre-specified degree of improvement in a patient reported outcome at 90 days.

Improvement in symptom cluster-specific patient-reported outcome measures, including the DePaul Symptom Questionnaire-Post Exertional Malaise (DSQ-PEM), PROMIS-cognitive 8a, or Orthostatic Hypotension Questionnaire (OHQ) at Day 90 compared to baseline in intervention(s) vs. control. For the PROMIS-cognitive 8a and OHQ, an average of 3 weekly assessments closest to Day 90 will be used.

A separate parallel primary efficacy analysis will be performed for each combination of 2 dose durations and 3 symptom clusters using the multiplicity adjustment procedure described in Section 5.5. First, efficacy of the 25-day dose duration will be compared to control within each symptom cluster. If all 3 cluster-specific comparisons are significant, after accounting for multiplicity, then an additional set of cluster-specific efficacy comparisons will be performed for the 15-day dose duration versus and control. Otherwise, testing will stop and all 15-day analyses will be regarded as exploratory.

The description below is generic in the sense that it pertains to any single combination of dose duration (versus control) and symptom cluster. For each cluster-specific primary endpoint comparison between an active dose duration and control, the estimand will be the between treatment-group difference in marginal probabilities of improvement, defined as

$$\Delta = P(Y = 1|A = 1) - P(Y = 1|A = 0),$$

where Y denotes the primary outcome (1 = improvement, 0 = no improvement) and A denotes randomization assignment (1 = active treatment, 0 = control). This quantity will be estimated without regard to adherence and will implicitly average over observed patterns of concomitant therapies. In the unlikely event that follow-up data collection is prevented by death or hospitalization, the participant will be classified as not improving. The null hypothesis is that the probability of improvement does not differ for active versus control groups, i.e., $\Delta = 0$. The alternative hypothesis is that the probability of improvement differs for active versus control groups, i.e., $\Delta \neq 0$.

Inference for Δ will be based on Wald-type test statistics and associated two-sided confidence intervals. The test statistic will have the form $Z = \widehat{\Delta}/\widehat{\text{se}}(\widehat{\Delta})$ where $\widehat{\Delta}$ is an estimate of Δ and $\widehat{\text{se}}(\widehat{\Delta})$ is its estimated standard error. Confidence intervals will have the form $\widehat{\Delta} \pm Z_{\alpha/2} \times \widehat{\text{se}}(\widehat{\Delta})$ where $Z_{\alpha/2}$ is the upper $1 - \alpha/2$ quantile of the standard normal distribution, e.g., $Z_{0.025} \approx 1.96$. Methods of calculating $\widehat{\Delta}$ and $\widehat{\text{se}}(\widehat{\Delta})$ are described below.

The procedure for estimating the between-group difference, $\widehat{\Delta}$, will be a special case of methodology described by Benkeser et al. 2020 [9], and also a special case of methodology described by Lunceford and Davidian 2004 [10]. It will combine logistic regression with direct standardization, also known as G-computation. To begin, a binary logistic regression model predicting the primary endpoint will be fitted to data from the pooled (active treatment group + control group) mITT cohort. The outcome variable

will be an indicator of the participant's primary endpoint status (1=improvement, 0=no improvement). Participants with missing primary endpoint status will be included but assigned weight zero during model fitting. Explanatory variables will consist of treatment group (active versus control), baseline covariates listed below, and all pairwise covariates × treatment group interactions. The inclusion of all covariate × treatment interactions is equivalent to fitting a separate outcome model for each of the two treatment groups. After estimating regression coefficients, the model's prediction equations will be applied to each participant in the pooled mITT in order to calculate the following results: (1) the participant's predicted probability of improvement if assigned to active treatment and (2) the participant's predicted probability of improvement if assigned to control. The final treatment effect estimate will be calculated by averaging these two sets of model predictions across all participants in the pooled mITT cohort and subtracting them. More concretely:

$$\widehat{\Delta} = \hat{\mu}_1 - \hat{\mu}_0, \quad \text{where} \quad \hat{\mu}_1 = \frac{1}{n}\sum_{i=1}^{n} m(x_i; \hat{\beta}_1), \quad \hat{\mu}_0 = \frac{1}{n}\sum_{i=1}^{n} m(x_i; \hat{\beta}_0),$$

$$m(x;\beta) = \exp(x'\beta)/[1 + \exp(x'\beta)\,],$$

$$\hat{\beta}_1 = \text{argmax}_\beta \sum_{i=1}^{n} c_i I(z_i = 1) \log\left[m(x_i;\beta)^{c_i y_i}\big(1 - m(x_i;\beta)\big)^{1-c_i y_i}\right]$$

$$\hat{\beta}_0 = \text{argmax}_\beta \sum_{i=1}^{n} c_i I(z_i = 0) \log\left[m(x_i;\beta)^{c_i y_i}\big(1 - m(x_i;\beta)\big)^{1-c_i y_i}\right].$$

Here, n is the number of participants in the pooled mITT cohort, z_i is an indicator of treatment assignment (1 = active, 0 = control), c_i is an indicator of whether i-th participant's primary endpoint is missing ($c_i = 0$) or non-missing ($c_i = 1$), y_i is an indicator of the i-th participant's primary endpoint status (1 = improvement, 0 = no improvement or missing), x_i is the i-th participant's vector of baseline covariates, and $I()$ is an indicator function taking the value 1 if its argument is true and taking the value 0 otherwise. An approximate two-sided $100(1-\alpha)$-level confidence interval for Δ will be calculated as $\widehat{\Delta} \pm Z_{\alpha/2}\widehat{\text{se}}(\widehat{\Delta})$ where $\widehat{\text{se}}(\widehat{\Delta})$ is based on a robust (empirical) variance estimator.

In absence of missing outcome data, the above estimator and standard error calculation will have the property of being asymptotically consistent (i.e., valid) regardless of whether or not assumptions underlying the logistic regression model are correct. Additionally, if missing outcome data occur, the calculations will continue to be valid if outcomes are missing at random (MAR), after conditioning on model covariates, and if the logistic regression model specification is correct.

Sensitivity/supplementary analyses using estimators that maintain validity under different missing data assumptions will also be implemented. Plans for these analyses are described in Section 8.2 and Appendix 2.

Pre-specified covariates for the primary efficacy analysis will be as follows:

- Cognitive symptom cluster only: Baseline PROMIS cognitive 8a T-score (continuous)
- Autonomic symptom cluster only: Baseline OHQ Question 1 (continuous)
- Exercise symptom cluster only: Number of symptoms meeting criteria for PEM (moderate severity and ≥50% frequency) on DSQ-PEM (continuous)
- Race (black, white, Asian, other) (categorical)
- Language (English, Spanish) (categorical)

- Years of education (continuous)
- US census division (New England, Atlantic, East North Central, West North Central, South Atlantic, East South Central, West South Central, Mountain, Pacific) (categorical)

Items 1-3 are symptom-specific outcomes measured at baseline. They were selected due to their suspected strong association with 90-day outcomes. Their inclusion can be viewed as adjusting for the net sum of all measured and unmeasured covariates that may randomly differ by treatment group and contribute to a participant's outcome status at baseline. Items 4-7 were added to mitigate a concern raised by the DMSB about potential for chance imbalances in site across treatment group, as discussed above.

8.2 Handling of Missing Data

8.2.1 Overview

A particularly strong effort will be made to prevent participant's loss to follow-up and missing outcome data. The frequency of missing data for key variables will be summarized by treatment group. The baseline characteristics and outcomes of participants with and without missing data will be compared. If assessment of a primary or secondary endpoint is prevented by death or hospitalization, the participant will be classified as not improving and will be considered to have non-missing outcome status.

As detailed above, the analysis strategy for the primary outcome analysis is a covariate-adjusted logistic regression combined with direct standardization. If missing outcome values occur, the estimator of the treatment effect will continue to be approximately unbiased, assuming that missingness is ignorable after conditioning on model covariates and assuming that the logistic regression model's underlying assumptions are correct. Missing values for pre-specified covariates will be handled by inclusion of missing data indicator variables [11].

8.2.2 Sensitivity Analysis #1: propensity-weighted logistic regression.

To assess sensitivity to the method of handling missing outcome data, the analysis described in Section 8.1 will be modified by incorporating inverse probability weighting (IPW) into the estimation of the outcome model. Each participant with non-missing outcome data will receive a weight proportional to the reciprocal of the participant's predicted probability of having non-missing outcome data. Inferences will be valid if outcomes are missing at random (MAR) and if at least one of the two models (outcome model or missing data model) is correctly specified.

8.2.3 Sensitivity Analysis #2: pattern mixture tipping point analysis.

To assess whether study results are robust under a range of missing not at random (MNAR) assumptions, a likelihood-based tipping point analysis will be will be conducted for the primary endpoints. The proposed approach is related to the method of Jackson et al. [12] and is a special case of pattern mixture modeling using logistic regression. Briefly, the procedure involves estimating two logistic regression models separately within each treatment group:

(1) Outcome model: a logistic regression predicting primary endpoint status as a function of covariates conditional on having non-missing primary endpoint data separately for each treatment group;
(2) Missing data model: a logistic regression predicting the probability of non-missing primary endpoint data as a function of covariates separately for each treatment group.

After estimating parameters, endpoints probabilities for participants with missing outcomes will be imputed by assuming they are shifted by a constant amount on the logit scale compared to participants with identical covariates and non-missing endpoint data within each treatment group. Treatment effects will be estimated under a range of analyst-specified shift amounts and presented with Wald-based approximate confidence intervals. Standard errors will be estimated using the delta method and will appropriately account for uncertainty arising from missing endpoint data. Tipping points will be identified as values of shift parameters that cause statistically significant treatment effect estimates to become non-significant or vice versa. Optionally, to facilitate interpretation, the above analysis may be repeated in a Bayesian statistical framework using default neutral priors for unknown logistic regression model parameters. Additional details are provided in Appendix 2.

8.3 Analysis of Secondary Endpoints

Each symptom cluster-specific secondary endpoint is a binary indicator of meeting a pre-specified degree of improvement on a performance outcome at 90 days.

Improvement in performance-based outcomes, including the endurance shuttle walk test (ESWT), active stand test, and/or neurocognitive battery at Day 90 compared to baseline in intervention(s) vs. control.

A separate analysis will be performed for each of three primary symptom cluster populations and two active dose durations.

Multiplicity considerations for secondary endpoints are discussed in Section 5.5.2. Briefly, to reduce type-I errors, analysis for a particular combination of symptom cluster and dose duration (versus control) will be conditional on having met significance for the primary endpoint for the same combination of dose duration and symptom cluster. For each symptom cluster and dose duration, if the hypothesis pertaining to the primary endpoint is rejected, then the symptom cluster's secondary endpoint will be tested at the same dose duration using alpha = 0.05. If the hypothesis pertaining to the primary endpoint is not rejected, then analysis of the secondary endpoint will be regarded as exploratory.

A covariate-adjusted analysis will be performed using pre-specified covariates as listed below. Estimation will target the between-group different in marginal probabilities of improvement for active dose versus control. Except for using different covariates, analysis methods will identical to the primary efficacy analysis.

The following covariates will be used:

- Cognitive dysfunction symptom cluster:
  - Baseline AVLT Z-score
  - Baseline SDMT Z-score
  - Demographic variables: race, language, years of education, US census division
- Autonomic dysfunction symptom cluster:
  - Baseline ΔSBP
  - Baseline ΔDBP
  - Baseline ΔHR
  - Demographic variables: race, language, years of education, US census division
- Exercise intolerance symptom cluster:
  - Baseline ESWT walk time

- Demographic variables: race, language, years of education, US census division

8.4 Analysis of Exploratory PRO and Performance Endpoints at Days 90 and 180

The PRO and performance outcomes listed in Table 3 will be analyzed at Days 90 and 180 for each pairwise comparison between active dose versus control.

Table 3. Exploratory efficacy endpoints to be analyzed at days 90 and 180

Exploratory Endpoint	Type
PROMIS Cognitive 8a T-score in cognitive cluster*	Continuous
OHQ Question #1 in autonomic cluster*	Continuous
DSQ-PEM number of criteria for PEM in exercise cluster*	Continuous
Neurocognitive battery outcomes at Day 90 in cognitive cluster*	Continuous
Change in BP and HR on active stand test at Day 90 in autonomic cluster*	Continuous
ESWT walk time at Day 90 in exercise cluster*	Continuous
PASC individual symptoms*	Continuous
PROMIS-29+2 domain scores*	Continuous
PROMIS-29+2 preference score (PROPr) *	Continuous
Patient global impression of change (PGIC)	Ordinal
ECOG-2 overall score in cognitive cluster*	Continuous
COMPASS-31 overall score in autonomic cluster*	Continuous
OHQ overall score in autonomic cluster*	Continuous

* Analysis will be adjusted for corresponding baseline value.

Except for the PGIC, all endpoints listed above will be treated as continuous variables and will be analyzed via linear regression with adjustment for the corresponding baseline measurement. PGIC will not be adjusted because PGIC directly assesses improvement in relation to baseline and thereby allows each participant to serve as his or her own control. Additional analysis details are provided below.

8.4.1 Analysis of Continuous Outcomes

For continuous outcomes, the estimand will be the between treatment-group difference in means, defined as

$$\Delta = E(Y|A = 1) - E(Y|A = 0),$$

where Y denotes the continuous outcome and A denotes randomization assignment (1 = active treatment, 0 = control). Methods of estimating Δ will be similar to the primary and secondary outcomes but will use a least squares linear regression instead of logistic model for the outcome. Pre-specified covariates will differ for each endpoint as listed in Table 3 above. The form of the estimator will be

$$\widehat{\Delta} = \hat{\mu}_1 - \hat{\mu}_0, \quad \text{where} \quad \hat{\mu}_1 = \frac{1}{n}\sum_{i=1}^{n} x_i'\hat{\beta}_1, \quad \hat{\mu}_0 = \frac{1}{n}\sum_{i=1}^{n} x_i'\hat{\beta}_0,$$

$$\hat{\beta}_1 = \text{argmin}_\beta \sum_{i=1}^{n} c_i I(z_i = 1)\,[c_i y_i - x_i'\beta\,]^2$$

$$\hat{\beta}_0 = \text{argmin}_\beta \sum_{i=1}^{n} c_i I(z_i = 0)\,[c_i y_i - x_i'\beta\,]^2,$$

where n is the number of participants in the mITT cohort, z_i is an indicator of treatment assignment (1 = active, 0 = control), c_i is an indicator of whether i-th participant's continuous outcome is missing ($c_i = 0$) or non-missing ($c_i = 1$), y_i is continuous outcome, x_i is the i-th participant's vector of baseline covariates, and $I()$ is an indicator function taking the value 1 if its argument is true and taking the value 0 otherwise. An approximate two-sided $100(1-\alpha)$-level confidence interval for Δ will be calculated as $\widehat{\Delta} \pm Z_{\alpha/2} \times \widehat{\text{se}}(\widehat{\Delta})$ where $Z_{\alpha/2}$ denotes the upper $(1-\alpha/2)$ quantile of the normal cumulative distribution function, e.g., $Z_{0.025} \approx 1.96$. The formula for estimating $\widehat{\text{se}}(\widehat{\Delta})$ will be identical to that described in Section 8.1 for the primary endpoints with the exception that $m(x;\hat{\beta})$ will be defined as $m(x;\hat{\beta}) = x'\hat{\beta}$ instead of $m(x;\hat{\beta}) = \exp(x'\hat{\beta})/[1+\exp(x'\hat{\beta})]$.

8.4.2 Analysis of PGIC (ordinal)

The PGIC is based on the question: "Compared to the start of the study, the overall severity of your Long COVID symptoms as _______." Response options are:

5) Improved a lot
4) Improved somewhat
3) Stayed about the same
2) Worsened somewhat
1) Worsened a lot

Because there are 5 possible values, the distribution of PGIC within each treatment group can be described by a set of 5 probabilities that sum to 1. Analysis will be nonparametric and will focus on comparing the distribution of category probabilities across the two treatment groups. The target estimand will be the marginal win ratio [13,14] (equivalently, the generalized odds ratio [15]), defined as:

$$\theta = \frac{P(\text{"win for active treatment"})}{P(\text{"win for control treatment"})} = \frac{P(Y^1 > Y^0)}{P(Y^1 < Y^0)},$$

where Y^1 denotes the outcome category of a randomly selected participant who is randomized to active treatment and Y^0 denotes the outcome category of a randomly selected participant who is randomized to control. Equivalently,

$$\theta = \frac{\sum_{i=1}^{5}\sum_{j=1}^{5} I(i>j)P(Y=i|Z=1)P(Y=j|Z=0)}{\sum_{i=1}^{5}\sum_{j=1}^{5} I(i<j)P(Y=i|Z=1)P(Y=j|Z=0)},$$

where Y denotes outcome category and Z denotes treatment assignment (1 = active, 0 = control). An estimate of the win ratio will be calculated as

$$\hat{\theta} = \frac{\sum_{i=1}^{5}\sum_{j=1}^{5} I(i>j)\hat{P}(Y=i|Z=1)\hat{P}(Y=j|Z=0)}{\sum_{i=1}^{5}\sum_{j=1}^{5} I(i<j)\hat{P}(Y=i|Z=1)\hat{P}(Y=j|Z=0)}$$

where

$$\hat{P}(Y=y|Z=z) = \frac{\sum_{i=1}^{n} c_i I(z_i=z) I(c_i Y_i = y)}{\sum_{i=1}^{n} c_i I(z_i = z)}, \quad y = 1,2,3,4,5, \quad z = 0,1,$$

where z_i is an indicator of the i-th participant's treatment assignment (1 = active, 0 = control), c_i is an indicator of whether i-th participant's PGIC is missing ($c_i = 0$) or non-missing ($c_i = 1$), and y_i is the i-th

participant's PGIC. An estimate greater than 1 will suggest that active treatment is superior to control, and an estimate less than 1 will suggest that control is superior to active treatment. An approximate 95% confidence interval for $\hat{\theta}$ will be calculated as

$$\text{lower limit} = \hat{\theta}/\exp\left\{Z_{0.025}\sqrt{\hat{V}}/\hat{\theta}\right\}, \quad \text{upper limit} = \hat{\theta} \times \exp\left\{Z_{0.025}\sqrt{\hat{V}}/\hat{\theta}\right\},$$

where $Z_{0.025} \approx 1.96$ and $\hat{V}$ is the variance estimator defined in Expression 4.3 of Agresti, 1980.

8.5 Analysis of Weekly Longitudinal PROs

The PRO outcomes listed in Table 4 are collected on a weekly basis and will be analyzed longitudinally for each pairwise comparison between active treatment and control.

Table 4. Endpoints to be analyzed longitudinally

Exploratory Endpoint	Type	Covariates
PROMIS Cognitive 8a T-score in cognitive cluster	Continuous	Baseline PROMIS 8a T-score
OHQ Question #1 in autonomic cluster	Continuous	Baseline OHQ Question #1
DSQ-PEM criteria for PEM in exercise cluster	Binary	Baseline no. symptoms meeting DSQ-PEM criteria
PROMIS-29 physical health summary score	Continuous	Baseline PROMIS-29 physical health summary score.

Week-specific data will be summarized as means and differences by treatment group with approximate 95% confidence intervals over weeks 1 to 26.

Differences by treatment group will be assessed by fitting generalized linear mixed models with participant-specific random effects (intercept and slope) using data from weeks 5 to 26. Weeks 1 to 4 will be excluded to reduce potential for attenuation of the treatment effect in the event that treatment effects experience a time lag. Models will incorporate a linear link for continuous outcomes (PROMIS Cognitive 8a, OHQ Question #1, PROMIS-29 physical health) and a logit link for the binary outcome (criteria for PEM). Fixed effects covariates will include treatment group, study week modeled as a restricted cubic spline, baseline values as listed in Table 4. Treatment effects will be expressed as differences in means for continuous outcomes and as odds ratios for the binary outcome. The above models omit time × by treatment interactions. The presence of time × treatment interactions will be assessed by adding interaction terms to the above models and testing whether the coefficients associated with interaction terms are equal to zero. If the hypothesis of no time × treatment interaction is rejected ($p < 0.05$), then results for models with interactions will also be presented.

8.6 Analysis of Heterogeneity of Treatment Effect (HTE)

8.6.1 Use of Pooled Active Treatment Groups

The list of pre-specified covariates for HTE analyses was intentionally kept small in order to reduce the number of comparisons and reduce potential for spurious findings. To further reduce comparisons, and

to increase sample size, HTE analyses will be performed using a pooled active treatment group that pools 15- and 25-day dose durations.

8.6.2 Endpoints for HTE Analyses

Exploratory analyses of heterogeneity of treatment effect (HTE) will be performed for the endpoints listed in Table 5.

Table 5. Endpoints for analysis of treatment effect heterogeneity

Exploratory Endpoint	Type	Covariates
PROMIS Cognitive 8a T-score in cognitive cluster	Continuous	Baseline PROMIS 8a T-score
OHQ Question #1 in autonomic cluster	Continuous	Baseline OHQ Question #1
DSQ-PEM criteria for PEM in exercise cluster	Continuous	Baseline no. symptoms meeting DSQ-PEM criteria
PROMIS-29 physical health summary score	Continuous	Baseline PROMIS-29 physical health summary score.
Patient global impression of change	Ordinal	None

8.6.3 Subgroups

Subgroups for HTE analyses are listed in Section 5.6.3. Subgroups of primary interest for HTE include:

- Antigenemia positive at baseline (yes, no)
- Vaccination status at time of first acute COVID-19 infection (≥1 dose completed, 0 doses completed)
- Time since first acute COVID-19 infection (grouped by approximate tertiles)

8.6.4 Estimation methods

Analyses of HTE will be performed one-at-a-time for each outcome and subgroup variable.

For continuous outcomes, HTE analysis will focus on estimating mean outcome differences between treatment group across levels of the subgroup variable. This will be accomplished by fitting an ANCOVA linear regression model with adjustment for treatment group (pooled active versus control), baseline measurement of the outcome variable, subgroup variable of interest, and the subgroup × treatment interaction. Estimated between treatment-group mean differences will be reported for each subgroup with associated 95% confidence intervals and with a p-value for the test of no subgroup × treatment interaction.

For the binary outcome (DSQ-PEM criteria for PEM in exercise cluster), HTE analysis will focus on estimating the difference in the probability of the outcome for pooled active versus control across levels of the specified subgroup variable. This will be accomplished by fitting a logistic regression model with adjustment for treatment group (pooled active versus control), baseline measurement of the outcome variable, subgroup variable of interest, and the subgroup × treatment interaction. After estimating regression coefficients, the model's prediction equations will be applied to each participant in the mITT cohort in order to calculate two sets of predicted probabilities per participant, namely, each participant's probability of improvement if assigned to active treatment and each participant's

probability of improvement if assigned to control. The treatment effect for a given subgroup will be estimated by averaging the two sets of model predictions across all participants belonging to the subgroup and subtracting them. Results will include estimated between-treatment group differences, associated 95% confidence intervals, and a p-value for the test of no subgroup × treatment interaction.

For PGIC (ordinal), the primary measure of treatment effect is the win ratio as defined in Section 8.4.2 above. HTE analysis will focus on estimating win ratios across each level of each pre-specified covariate. To accomplish this, the win ratio estimation methodology described in 8.4.2 above will be applied to data from each subgroup separately. Evidence of treatment × subgroup interaction will be assessed by testing the alternative hypothesis that the win ratio varies across levels of the subgroup against the null hypothesis that the win ratio is constant. For a categorical covariate with k levels (i.e., k subgrops) the assessment of a covariate × treatment interaction will be based on a chi-square test with $k-1$ degrees of freedom. Let $\hat{d}_j = \log \hat{\theta}_j$ be the estimated log-transformed win ratio statistic calculated using data from participants in the j-th of k subgroups and let $\hat{v}_j$ be the corresponding estimated variance. Define

$$\widehat{\boldsymbol{D}} = \begin{bmatrix} \hat{d}_1 \\ \hat{d}_2 \\ \vdots \\ \hat{d}_k \end{bmatrix} \quad \text{and} \quad \widehat{\boldsymbol{V}} = \begin{bmatrix} \hat{v}_1 & 0 & 0 & 0 \\ 0 & \hat{v}_2 & 0 & 0 \\ \vdots & 0 & \ddots & 0 \\ 0 & 0 & 0 & \hat{v}_k \end{bmatrix},$$

and let $\boldsymbol{C}_k$ be the $(k-1) \times k$ contrast matrix

$$\boldsymbol{C}_2 = (1 \quad -1), \quad \boldsymbol{C}_3 = \begin{pmatrix} 1 & -1 & 0 \\ 1 & 0 & -1 \end{pmatrix}, \quad \boldsymbol{C}_4 = \begin{pmatrix} 1 & -1 & 0 & 0 \\ 1 & 0 & -1 & 0 \\ 1 & 0 & 0 & -1 \end{pmatrix}, \quad \text{etc.}$$

The chi-square test statistic for interaction will be calculated as

$$X = \left(\boldsymbol{C}_k \widehat{\boldsymbol{D}}\right)' \left[\boldsymbol{C}_k \widehat{\boldsymbol{V}} \boldsymbol{C}_k'\right]^{-1} \left(\boldsymbol{C}_k \widehat{\boldsymbol{D}}\right).$$

A p-value will be obtained by calculating the probability that a chi-square random variable with $k-1$ degrees of freedom exceeds X.

8.6.5 Estimation of Covariate-specific Treatment Effects

As a further exploratory analysis, a Bayesian multivariable modeling strategy may be used to estimate the treatment effects for a subset of the above outcomes as a function of the 3 pre-specified primary interest subgroup variables jointly. This analysis will improve on conventional subgroup analyses by analyzing multiple subgroup factors jointly in a single multivariable model. Results of this analysis may inform the choice of target population for a possible future Phase 3 evaluation. Results will be shared with study investigators and NIH leadership to inform future study design but may be omitted from the final CSR. A separate analysis plan will be developed for this analysis.

8.7 Bayesian Analyses

To enhance interpretation, the endpoints listed in Table 6 below will be re-analyzed in a Bayesian statistical framework. Analysis will be performed using a range of skeptical, neutral, and enthusiastic priors as suggested by Wijeysundera et al. (2009) [13] and Zampieri et al. (2021) [17]. Results reporting will be facilitated by an R shiny app that will display results for a range of user-requested prior distributions.

8.7.1 Endpoints and statistical framework

Table 6. Endpoints for exploratory Bayesian analyses

Endpoint	Analysis methods
PROMIS Cognitive 8a T-score in cognitive cluster at day 90 (continuous)	• Analysis framework:<br>○ ANCOVA linear regression<br>• Covariates:<br>○ Baseline PROMIS Cognitive 8a T-score, re-scaled to have SD = 1.<br>○ Treatment group indicator<br>• Treatment effect estimand:<br>○ Between treatment-group mean difference as captured by regression coefficient for treatment
Presence of PEM according to DSQ-PEM in exercise cluster at day 90 (binary)	• Analysis framework:<br>• Binary logistic regression<br>• Covariates:<br>• Baseline number of symptoms meeting criteria for PEM, re-scaled to have SD = 1.<br>• Treatment group indicator<br>• Treatment effect estimand:<br>• Marginal difference in probability of PEM, calculated by averaging predicted probabilities under active and control treatments across all mITT participants
OHQ Question #1 in autonomic cluster at day 90 (continuous)	Analysis framework:<br>ANCOVA linear regression<br>Covariates:<br>Baseline OHQ Question #1, re-scaled to have SD = 1.<br>treatment group indicator<br>Treatment effect estimands:<br>Between treatment-group mean difference as captured by regression coefficient for treatment
Patient global impression of change (PGIC) in overall mITT cohort at day 90 (ordinal)	Analysis framework:<br>Nonparametric: PGIC will be assumed to follow a multinomial distribution with cell probabilities differing by treatment group<br>Covariates:<br>None, unadjusted<br>Treatment effect estimand:<br>Win ratio, as defined in Section 8.4.2 above

8.7.2 Prior Specification for Cognitive 8a

The prior will consist of independent normal distributions for regression coefficients and an inverse Gamma distribution for the residual variance. In light of limited prior information, the prior for the

baseline PROMIS Cognitive 8a coefficient will be normal with mean 0 and SD = 10. For analyses using a neutral prior, the prior for the treatment coefficient will be normal with mean 0 and SD = 10. Priors for the treatment coefficient will be as follows:

- Neutral:
  - $\beta \sim$ Normal(mean = 0, SD = 10)
  - This prior is chosen to reflect a state of uncertainty concerning the direction and magnitude of the treatment effect.
- Skeptical:
  - $\beta \sim$Normal(mean = 0, SD = 3)
  - This prior implies that:
    - The prior most likely value for the mean difference is 0 T-score points
    - The prior probability of a benefit exceeding 5 T-score points is approximately 5%
- Enthusiastic:
  - $\beta \sim$ Normal(mean = 5, SD = 7.5)
  - This prior implies that:
    - The prior most likely value for the mean difference is 5 T-score points
    - The prior probability of any benefit ($\beta > 0$) is approximately 75%.

8.7.3 Prior Specification for OHQ Question #1

The prior will consist of independent normal distributions for regression coefficients and an inverse Gamma distribution for the residual variance. In light of limited prior information, the prior for the baseline OHQ Question #1 coefficient will be normal with mean 0 and SD = 10. For analyses using a neutral prior, the prior for the treatment coefficient will be normal with mean 0 and SD = 10. Priors for the treatment coefficient will be as follows:

- Neutral:
  - $\beta \sim$ Normal(mean = 0, SD = 10)
  - This prior is chosen to reflect a state of uncertainty concerning the direction and magnitude of the treatment effect.
- Skeptical:
  - $\beta \sim$Normal(mean = 0, SD = 0.6)
  - This prior implies that:
    - The prior most likely value for the mean difference in Question #1 is 0 units
    - The prior probability of a benefit exceeding 1 unit is approximately 5%
- Enthusiastic:
- $\beta \sim$ Normal(mean = 1, SD = 1.5)
  - This prior implies that:
    - The prior most likely value for the mean difference is 5 T-score points
    - The prior probability of any benefit ($\beta > 0$) is approximately 75%.

8.7.4 Prior Specification for PEM

The prior will consist of independent normal distributions for all regression coefficients. In light of limited prior information, the prior for the baseline number of qualifying PEM symptoms coefficient will be normal with mean = 0 and SD = 10. Priors for the treatment coefficient will be as follows:

- Neutral:
  - $\beta \sim$ Normal(mean = 0, SD = 10)

    - This prior is chosen to reflect a state of uncertainty concerning the direction and magnitude of the treatment effect.
- Skeptical:
    - β ~Normal(mean = 0, SD = 0.25)
    - This prior implies that:
        - The prior most likely value of the treatment effect odds ratio is 1
        - The prior probability of a treatment effect odds ratio exceeding 1.5 is approximately 5%
- Enthusiastic:
    - β ~ Normal(mean = 0.405, SD = 0.6)
    - This prior implies that:
        - The prior most likely value for the treatment effect odds ratio is 1.5
        - The prior probability of any benefit (odds ratio > 1) is approximately 75%.

8.7.5 Prior Specification for PGIC

Full details will be developed in a later version of the SAP. Briefly, prior specification for the PGIC win ratio will follow the nonparametric marginal prior specification methodology of Kessler et al. 2015. [18] The prior for the win ratio estimand of interest will be a log-normal distribution. The conditional distribution of remaining unknown parameters (i.e., the unknown set of multinomial probabilities in the two treatment groups of interest) will be proportional to a product of two independent Dirichlet distributions. The following settings will be used.

- Neutral:
    - $\log(\text{win ratio})$ ~ Normal(mean = 0, SD = 10)
    - This prior is chosen to reflect a state of uncertainty concerning the direction and magnitude of the treatment effect.
- Skeptical:
    - $\log(\text{win ratio})$ ~Normal(mean = 0, SD = 0.25)
    - This prior implies that:
        - The prior most likely value of the treatment effect win ratio is 1
        - The prior probability of a treatment effect win ratio exceeding 1.5 is approximately 5%
- Enthusiastic:
    - $\log(\text{win ratio})$ ~ Normal(mean = 0.405, SD = 0.6)
    - This prior implies that:
        - The prior most likely value for the treatment effect win ratio is 1.5
        - The prior probability of any benefit (win ratio > 1) is approximately 75%.

8.7.6 Bayesian R Shiny App

The analyses described above are based on a small number of pre-specified prior distributions. To facilitate results reporting under a wider range of priors, an R Shiny app will be developed. It will be designed to provide interactive Bayesian analysis under an arbitrary range of user-specified subjective prior distributions. For a subset of the above endpoints, the app will calculate the posterior probability that the unknown treatment effect falls above or below a user-specified margin representing clinically important harm or benefit. For model-based analyses, the app will assign a user-specified prior to the treatment effect parameter of interest and a default reference prior to other parameters. An importance weighting strategy will be used to update posterior probabilities in real time without

needing to repeat computationally intensive posterior sampling when the user's choice of prior is varied. For PGIC, the app will implement a modification of the nonparametric marginal prior specification methodology of Kessler et al. 2015. [18] The modification involves using importance weighting to update results in near real time when the user's prior is varied. Details will be developed in a separate document.

9 ANALYSIS OF MEDICAL VISITS AND HOSPITALIZATIONS

Descriptive analyses will summarize the mean number of healthcare encounters and the proportion of participants with at least one encounter by treatment group for all types of encounters in aggregate and for specific types of encounters (inpatient hospitalization, outpatient ER visit, primary care provider visit, urgent care visit, planned hospitalization unrelated to COVID-19).

Statistical analysis will focus on comparing the cumulative mean number of all healthcare encounters and the cumulative mean number of inpatient hospitalizations per participant for each active dose group versus control at 12 weeks post-randomization and at 25 weeks post-randomization. These timepoints were chosen to roughly correspond to the beginning of the visit windows for the 90-day and end-of-study visits. The Ghosh–Lin method [19] will be used to estimate mean encounters and hospitalizations in a manner that accounts for censored observations and treats death as a competing risk for subsequent encounters and hospitalizations. Group-specific means and the between-group difference in means will be presented with 95% confidence intervals.

The Ghosh–Lin method is a modification of traditional statistical methods used to estimate the cumulative probability of experiencing an endpoint of interest within a specified time horizon. Unlike 'cumulative incidence' methods, which only consider the first events and overlook subsequent ones, Ghosh and Lin14 introduced an adaptation which incorporates recurrent events and treats death as an terminating event (informative censoring). The method is non-parametric (i.e., no distributional assumptions) and allows for estimating the average cumulative number of events (initial and recurrent) over time from randomization within each treatment group in the presence of censoring. It also accounts for the fact that patients who die can no longer contribute to the event count after their death. This is done by keeping patients who die in the risk set beyond their time of death but setting their event rate after death to zero. No assumptions are required about the relationship between recurrent events in the same patients.

10 SAFETY ANALYSES

Safety endpoints include the proportion of participants who experience individual AEs/SAEs and the proportion who experience any one or more AEs/SAEs. Each of these will be compared pairwise between each of the two intervention dose levels and control. Risk differences between each intervention dose level and control group and 95% CIs using the Miettinen-Nurminen method will be estimated. Incidence of AEs/SAEs leading to discontinuation of study intervention will also be summarized.

AEs will be coded in accordance with the current version of the Medical Dictionary for Regulatory Activities (MedDRA). AEs will be graded with regard to severity according to criteria defined in the Common Terminology Criteria for Adverse Events version 5.0 (CTCAE) and relationship to study intervention (related/not related). Each AE will be counted once only for a given participant. AEs will be presented by system organ class, preferred term (PT), duration (in days), start- and stop-date. AEs/SAEs leading to premature discontinuation from the study intervention will be presented in a listing.

For this study intervention appendix, there are two ESIs specified in the protocol: 1. Alteration in thyroid function and 2. Drug-Induced Liver Injury (DILI). ESIs will be summarized.

10.1 Clinical Laboratory Data

Blood for clinical laboratory assessments will be collected at baseline, drug dosing midpoint, end of dosing, and day 90 for all participants. Parameters evaluated include a comprehensive metabolic panel, high-sensitivity c-reactive protein, D-dimer, coagulation panel (prothrombin time, activated partial thromboplastin time, thrombin time), thyroid stimulating hormone (TSH), total and free triiodothyronine (T3), and total and free thyroxine (T4), and complete blood count with differential.

For the purposes of reporting safety laboratory assessments, the following visit windows will be used:

Table 7. Analysis Visit Windows for Laboratory Data

Visit	Target Study Day	Analysis Window Study Day	
		Low	High
Baseline	0	≤ 0	0
Drug Dosing Midpoint Clinic Visit	15	1	20
End of Dosing Clinic Visit	25	21	35
Optional Day 45	45	36	52
Optional Day 60	60	53	75
Day 90	90	76	105
Optional Day 120	120	106	End of Study

Descriptive statistics for baseline and post-baseline time points will be generated. Additionally, shift tables will be presented. The shift tables tabulate the number of lab values determined to be “Normal” and “Abnormal” at baseline and post-baseline time points. The classifications on shift tables will categorize lab values as “Normal” when they are in acceptable range. Laboratory values outside the normal range will be graded on the Labs Listing as >1x, >2x, >3x, >5x, or >10x the upper limit (or <0.1x, <0.5x, <1x the lower limit) of normal value based on appropriate increases or decreases. Laboratory data will also be summarized graphically to show the magnitude and changes in individual subject values over time relative to normal ranges. Details of any abnormalities will be provided in a listing.

11 BIOMARKER ANALYSES

Plans for biomarker analyses will be developed in a separate biomarker SAP. Briefly, markers of interest for mechanistic evaluation include antigenemia (defined as detection of SARS-CoV-2 spike protein in plasma), inflammation markers (high-sensitivity c-reactive protein, D-dimer), and coagulation markers (prothrombin time, activated partial thromboplastin time, thrombin time). Distributions of these biomarkers will be descriptively summarized and compared across treatment groups at baseline, mid-dosing visit, end of dosing visit, and days 45, 60, 90, and 120. Viral clearance will be assessed by comparing the frequency of antigenemia at post-randomization visits across treatment groups among participants who are positive for antigenemia at baseline.

12 ADHERENCE

Plans for reporting on study drug adherence will be finalized after reviewing data from the Study Drug Adherence form for the first several participants. Assuming adequate data capture, the distribution of the number of completed and missed doses will be summarized by treatment group both overall and separately for the first 15 days of the dosing period and the second 10 days of the dosing period.

13 REFERENCES

1. Yost KJ, Eton DT, Garcia SF, Cella D. Minimally important differences were estimated for six Patient-Reported Outcomes Measurement Information System-Cancer scales in advanced-stage cancer patients. J Clin Epidemiol. May 2011;64(5):507-16. doi:10.1016/j.jclinepi.2010.11.018

2. Kroenke K, Stump TE, Chen CX, et al. Minimally important differences and severity thresholds are estimated for the PROMIS depression scales from three randomized clinical trials. J Affect Disord. Apr 1 2020;266:100-108. doi:10.1016/j.jad.2020.01.101

3. Terwee CB, Peipert JD, Chapman R, et al. Minimal important change (MIC): a conceptual clarification and systematic review of MIC estimates of PROMIS measures. Qual Life Res. Oct 2021;30(10):2729-2754. doi:10.1007/s11136-021-02925-y

4. University; N. Meaningful Change for PROMIS(R). Accessed January 20, 2023. https://www.healthmeasures.net/score-and-interpret/interpret-scores/promis/meaningful-change

5. Norman GR, Sloan JA, Wyrwich KW. Interpretation of changes in health-related quality of life: the remarkable universality of half a standard deviation. Med Care. May 2003;41(5):582-92. doi:10.1097/01.Mlr.0000062554.74615.4c

6. HealthMeasures. PROMIS adult profile instruments scoring manual. September 2021. https://www.healthmeasures.net/images/PROMIS/manuals/Scoring_Manuals_/PROMIS_Adult_Profile_Scoring_Manual.pdf

7. U.S. Food and Drug Administration. "Adjusting for covariates in randomized clinical trials for drugs and biological products: guidance for industry." May 2021. https://www.fda.gov/media/148910/download

8. U.S. Food and Drug Administration. " COVID-19: Developing drugs and biological products for treatment or prevention: guidance for industry." February 2021. https://www.fda.gov/media/167274/download

9. Benkeser D, Díaz I, Luedtke A, Segal J, Scharfstein D, Rosenblum M. Improving precision and power in randomized trials for COVID-19 treatments using covariate adjustment, for binary, ordinal, and time-to-event outcomes. Biometrics. Dec 2021;77(4):1467-1481. doi:10.1111/biom.13377

10. Lunceford JK, Davidian M. Stratification and weighting via the propensity score in estimation of causal treatment effects: a comparative study. Statistics in medicine. 2004 Oct 15;23(19):2937-60.

11. Zhao A, Ding P. To adjust or not to adjust? estimating the average treatment effect in randomized experiments with missing covariates. Journal of the American Statistical Association. 2022 Oct 11:1-1.

12. Jackson D, White IR, Mason D, Sutton S. A general method for handling missing binary outcome data in randomized controlled trials. Addiction. 2014 Dec;109(12):1986-93.

13. Gasparyan SB, Folkvaljon F, Bengtsson O, Buenconsejo J, Koch GG. Adjusted win ratio with stratification: calculation methods and interpretation. Statistical Methods in Medical Research. 2021 Feb;30(2):580-611.

14. Gasparyan SB, Kowalewski EK, Folkvaljon F, Bengtsson O, Buenconsejo J, Adler J, Koch GG. Power and sample size calculation for the win odds test: application to an ordinal endpoint in COVID-19 trials. Journal of Biopharmaceutical Statistics. 2021 Nov 2;31(6):765-87.

15. Agresti A. Generalized odds ratios for ordinal data. Biometrics. 1980 Mar 1:59-67.

16. Wijeysundera DN, Austin PC, Hux JE, Beattie WS, Laupacis A. Bayesian statistical inference enhances the interpretation of contemporary randomized controlled trials. Journal of clinical epidemiology. 2009 Jan 1;62(1):13-21.

17. Zampieri FG, Casey JD, Shankar-Hari M, Harrell Jr FE, Harhay MO. Using Bayesian methods to augment the interpretation of critical care trials. An overview of theory and example reanalysis of the alveolar recruitment for acute respiratory distress syndrome trial. American journal of respiratory and critical care medicine. 2021 Mar 1;203(5):543-52.

18. Kessler DC, Hoff PD, Dunson DB. Marginally specified priors for non-parametric Bayesian estimation. Journal of the Royal Statistical Society Series B: Statistical Methodology. 2015 Jan;77(1):35-58.

19. Ghosh D, Lin DY. Nonparametric analysis of recurrent events and death. Biometrics. 2000 Jun;56(2):554-62.

14 APPENDICES

Appendix 1. RECOVER-VITAL Roster of Statisticians

Team	Name	Email	Attends DSMB Meetings
Unblinded	Frank Rockhold	frank.rockhold@duke.edu	X
	Zhen Huang	zhen.huang@duke.edu	X
	Sophia Waymyers	sophia.waymyers@duke.edu	X

Blinded	Sean O'Brien	sean.m.obrien@duke.edu	X
	Adam Silverstein	adam.silverstein@duke.edu	
	Pearl Zakroysky	pearl.zakroysky@duke.edu	X

Appendix 2. Sensitivity analyses for missing outcomes

Details of Sensitivity Analysis #2: Pattern Mixture Tipping Point Analysis

Let Y denote primary endpoint status (1=yes, 0=no), X denote a vector of baseline covariates, Z denote randomized treatment group (1=active treatment, 0=control), and R denote an indicator of the primary endpoint's missingness status (1 = non-missing, 0=missing). By randomization, the variables X and Z are assumed to be independent. The treatment effect estimand of interest is

$$\Delta = P(Y = 1|Z = 0) - P(Y = 1|Z = 0). \quad (1)$$

Without making additional assumptions or losing generality, the above estimand may be written as

$$\Delta = \left(\sum_x f_x p_x^1 m_{1x}^1 + f_x(1 - p_x^1)m_{0x}^1\right) - \left(\sum_x f_x p_x^0 m_{1x}^0 + f_x(1 - p_x^0)m_{0x}^0\right), \quad (2)$$

where $f_x = P(X = x)$, $p_x^z = P(R = 1|X = x, Z = z)$, $m_{rx}^z = P(Y = 1|X = x, Z = z, R = r)$, for $z = 0,1$, $r = 0,1$; and Σ_x denotes summation across all possible configurations of baseline covariates. The analysis will assume that functions p_x^z, m_{1x}^z, and m_{0x}^z are described by logistic regression models,

$\text{logit}\, p_{1x}^z = x'\beta_z$ (missing data model)

$\text{logit}\, m_{1x}^z = x'\alpha_z$ (outcome model, conditional on non-missing endpoint)

$\text{logit}\, m_{0x}^z = x'\alpha_z + \text{shift}_z$ (outcome model, conditional on missing endpoint)

where α_z and β_z are unknown parameters to be estimated from the data and shift_z is an analyst-specified constant that describes the hypothesized direction and magnitude of the association between Y and R within treatment group z. An estimate of Δ will be calculated as

$$\hat{\Delta} = \hat{\Delta}(\hat{\alpha}, \hat{\beta}; \text{shift}_0, \text{shift}_1) = \left(\sum_x \hat{f}_x \hat{p}_x^1 \widehat{m}_{1x}^1 + \hat{f}_x(1 - \hat{p}_x^1)\widehat{m}_{0x}^1\right) - \left(\sum_x \hat{f}_x \hat{p}_x^0 \widehat{m}_{1x}^0 + \hat{f}_x(1 - \hat{p}_x^0)\widehat{m}_{0x}^0\right)$$

where $\hat{p}_x^1$, $\widehat{m}_{1x}^1$,$\widehat{m}_{0x}^1$, $\hat{p}_x^0$, $\widehat{m}_{1x}^0$,$\widehat{m}_{0x}^0$ denote maximum likelihood estimates and $\hat{f}_x$ denotes the observed empirical covariate distribution. Details of the proposed delta method standard error formula will be documented in a subsequent version of the SAP or SAP appendix. Because it relies on large sample approximations, its accuracy will be studied via Monte Carlo simulation before performing the final analysis. If in doubt, inference will instead be performed by fitting the above models in a Bayesian statistical framework using Markov Chain Monte Carlo methods.

RECOVER VITAL SAP V1

Final Audit Report 2024-02-16

Created:	2024-02-13
By:	Pearl Zakroysky (pz33@duke.edu)
Status:	Signed
Transaction ID:	CBJCHBCAABAArTzpK3tMlWZTX7MWyRrV1aqMhndV2IFy

"RECOVER VITAL SAP V1" History

Document created by Pearl Zakroysky (pz33@duke.edu)
2024-02-13 - 7:11:13 PM GMT

Document emailed to Kanecia Zimmerman (kanecia.zimmerman@duke.edu) for signature
2024-02-13 - 7:13:43 PM GMT

Email viewed by Kanecia Zimmerman (kanecia.zimmerman@duke.edu)
2024-02-14 - 3:55:14 AM GMT

Document e-signed by Kanecia Zimmerman (kanecia.zimmerman@duke.edu)
Signing reason: Approved
Signature Date: 2024-02-14 - 9:39:33 PM GMT - Time Source: server

Document emailed to sean.m.obrien@duke.edu for signature
2024-02-14 - 9:39:34 PM GMT

Email viewed by sean.m.obrien@duke.edu
2024-02-14 - 9:40:09 PM GMT

Email viewed by sean.m.obrien@duke.edu
2024-02-16 - 2:05:06 AM GMT

Signer sean.m.obrien@duke.edu entered name at signing as Sean O'Brien
2024-02-16 - 2:05:33 AM GMT

Document e-signed by Sean O'Brien (sean.m.obrien@duke.edu)
Signing reason: Approved
Signature Date: 2024-02-16 - 2:05:35 AM GMT - Time Source: server

Document emailed to lbaden@bwh.harvard.edu for signature
2024-02-16 - 2:05:37 AM GMT

Email viewed by lbaden@bwh.harvard.edu

2024-02-16 - 3:32:35 AM GMT

Signer lbaden@bwh.harvard.edu entered name at signing as Lindsey Baden

2024-02-16 - 11:17:30 AM GMT

Document e-signed by Lindsey Baden (lbaden@bwh.harvard.edu)

Signing reason: Approved

Signature Date: 2024-02-16 - 11:17:32 AM GMT - Time Source: server

Document emailed to rwhitley@uabmc.edu for signature

2024-02-16 - 11:17:34 AM GMT

Email viewed by rwhitley@uabmc.edu

2024-02-16 - 11:17:41 AM GMT

Signer rwhitley@uabmc.edu entered name at signing as Richard Whitley

2024-02-16 - 12:32:28 PM GMT

Document e-signed by Richard Whitley (rwhitley@uabmc.edu)

Signing reason: Approved

Signature Date: 2024-02-16 - 12:32:30 PM GMT - Time Source: server

Agreement completed.

2024-02-16 - 12:32:30 PM GMT

RECOVER VITAL SAP - List of updates from V1 to V2

1. Section 1 (Study and Document Overview): Added sentence about exceeding the targeted sample size.
2. Section 2.2.3 (Exploratory and Safety Endpoints):
    a. Removed longitudinal analysis of the PROMIS-29+2
    b. Added that details will be developed in a separate SAP for full autonomic testing and cardiopulmonary exercise testing endpoints.
3. Section 3 (Statistical Design) – No updates made.
4. Section 4
    a. Table 2 (Abbreviated schedule of assessments):
        i. Added footnote about visit windows for data used in efficacy analyses
    b. 4.2 (Symptom Cluster Assignment):
        i. Added language around inclusion of participants randomized to multiple symptom clusters
    c. 4.3 (Definition of Baseline):
        i. Added language around identifying baseline records for efficacy and safety analyses.
    d. 4.4.3 (DSQ-PEM):
        i. Added language around handling of missing data for defining severity and frequency criteria used to derive improvement.
    e. 4.4.6 (PROMIS-29+2):
        i. Removed the PROPr score.
    f. 4.4.8 (PASC Symptom Questionnaire):
        i. Added language that there are 2 versions of the PASC.
    g. 4.4.9 (Neurocognitive battery):
        i. Updated wording of the definition of the binary endpoint so that it is more clear. The actual definition did not change.
    h. 4.4.11 (ESWT):
        i. Removed sentence about when performed since already mentioned below.
5. Section 5
    a. 5.4 (Populations for Analysis):
        i. Added that participants must be eligible, enrolled, and randomized to be included into the symptom cluster population.
        ii. Added definition of the overall mITT population.
        iii. Added the high study drug adherence population.
        iv. Updated wording for safety analyses.
    b. 5.6.3 (Subgroups):
        i. Updated "time since first acute COVID-19 infection" subgroup to "time from first COVID infection that led to current PASC symptoms to randomization".
6. Section 6
    a. 6.2 (Demographics and Medical History):
        i. Updated wording for better clarity.
        ii. Added continuous statistics that will be presented.
        iii. Updated list of baseline characteristics to be consistent with data collected and available.

   b. 6.4 (Baseline and Concomitant Medications):
      i. Added language that concomitant medications will be limited to those taken by 2% or more of the population at both baseline and day 90.
7. Section 7 (Interim Analyses and Data Monitoring) – No updates made.
8. Section 8
   a. 8.1 (Primary Efficacy Analysis):
      i. Added language that the analysis window will be between Day 76 and Day 104.
      ii. Added language that an average of all available assessments will be used if fewer than 3 assessments are completed within the window.
      iii. The remainder of this section was heavily edited to better describe between-group comparisons and the binary logistic regression model.
   b. 8.2.1 (Overview):
      i. Added details around how death and hospitalization will be ascertained for determining if the primary endpoint will stay missing or set to no improvement.
   c. 8.2.2 (Consideration for primary endpoint analysis): New section added for handling missing baseline and/or the primary endpoint data.
   d. 8.2.3 (Sensitivity Analysis: propensity-weighted logistic regression):
      i. Updated language
      ii. Removed sensitivity analysis #2 (pattern mixture tipping point analysis).
   e. 8.3 (Analysis of Secondary Endpoints):
      i. Added sentence about analysis window
      ii. Removed language as a covariate
      iii. Updated US census division to US region
   f. 8.4 (Analysis of Exploratory PRO and Performance Endpoints at Days 90 and 180):
      i. Removed PASC and PROMIS-29+2 PROPr from Table 3
      ii. Added language about presentation of the PASC as descriptive
   g. 8.4.1 (Analysis of Continuous Outcomes):
      i. Updated details so that analysis is identical to primary and secondary outcomes
      ii. Removed analysis of PGIC
   h. 8.5 (Analysis of Weekly Longitudinal PROs)
      i. Updated DSQ-PEM endpoint from binary to continuous
      ii. Removed PROMIS-29 physical health summary score
   i. 8.6.2 (Endpoints for HTE Analyses)
      i. Updated DSQ-PEM endpoint from binary to continuous
      ii. Removed PROMIS-29 physical health summary score and PGIC
   j. 8.6.4 (Estimation methods):
      i. Removed methods for DSQ-PEM (since no longer binary) and PGIC (since no longer analyzing for HTE)
   k. 8.7.1 (Endpoints and statistical framework):
      i. Added note that the PGIC will be shared with study investigators but not included in the final CSR.
      ii. Details for PGIC deleted from this section and moved to Appendix 2.
9. Section 9 (Analysis of Medical Visits and Hospitalizations):
   a. Removed Ghosh-Lin method and updated to presentation of only descriptive statistics.

10. Section 10 (Safety Analyses):
    a. Removed listing of AEs/SAEs leading to premature intervention discontinuation since don't have this information easily available.
11. Section 11 (Biomarker Analyses) – No updates made.
12. Section 12 (Adherence):
    a. Added details around what information will be presented and how it will be summarized.
13. Section 13 (References):
    a. Updated to remove references deleted from body of the SAP and no longer applicable
14. Section 14 (Appendices):
    a. Appendix 1
        i. Added Sam Broderick to the roster
    b. Appendix 2 - moved from body of the SAP.

A Platform Protocol for Evaluation of Interventions for Viral Persistence, Viral Reactivation, and Immune Dysregulation in Post-Acute Sequelae of SARS-CoV-2 Infection (PASC)

Protocol: RECOVER-VITAL

STATISTICAL ANALYSIS PLAN (SAP)

Protocol Version 4.0

Protocol Version Date: 02 NOV 2023

SAP Version Number: 2.0

3 APR 2025

Kanecia Zimmerman, MD, PhD, MPH

Professor of Pediatrics, Pediatrics-Critical Care Med Duke

Clinical Research Institute

300 W. Morgan Street Durham,

NC 27701

Tel: 919-668-8651

E-mail: kanecia.zimmerman@duke.edu

SAP Approval Page

Study Title: RECOVER-VITAL: A Platform Protocol for Evaluation of Interventions for Viral Persistence, Viral Reactivation, and Immune Dysregulation in Post-Acute Sequelae of SARS-CoV-2 Infection (PASC)

Version: 2.0

Date of Issue: 3 APR 2025

Study Sponsor: Kanecia Zimmerman, MD, PhD, MPH
Professor of Pediatrics, Pediatrics-Critical Care Med
Duke Clinical Research Institute
300 W. Morgan Street, Durham, NC 27701

We, the undersigned, have read and approve of this SAP and agree on its content.

Electronically signed by: Kanecia Zimmerman
Reason: Approve Date: Apr 3, 2025 12:02 EDT

____ __________

Sponsor Representative – Kanecia Zimmerman, MD, PhD, MPH

Sean O'Brien Electronically signed by: Sean O'Brien Reason: Approve Date: Apr 3, 2025 15:45 EDT

____

RECOVER-VITAL Reviewing Statistician – Sean O'Brien, PhD

Electronically signed by: Lindsey Baden
Lindsey Baden Reason: Approve Date: Apr 3, 2025 12:58 PDT

____

RECOVER-VITAL Co-Principal Investigator – Lindsey Baden, MD

Electronically signed by: Richard Whitley
Richard Whitley Reason: Approve Date: Apr 3, 2025 16:22 CDT

____

RECOVER-VITAL Co-Principal Investigator – Richard Whitley, MD

TABLE OF CONTENTS

Table of Contents 2
List of Abbreviations 5

1 Study and Document Overview 7
2 Study Objectives and Endpoints 8
2.1 Study Objectives 8
2.2 Endpoints 8
3 Statistical design 12
3.1 Power and Sample Size 12
3.2 Randomization Methods 14
4 Study Assessments and Definitions 15
4.1 Study Assessments 15
4.2 Symptom Cluster Assignment 16
4.3 Definition of Baseline 18
4.4 Efficacy Assessments 18
5 General Analysis Considerations 24
5.1 Data Sources 24
5.2 Software 24
5.3 Verification of Results 24
5.4 Populations for Analysis 24
5.5 Multiple Testing 25
5.6 Covariate Adjustment and Subgroups 26
6 Summary of Study Data 28
6.1 Subject Disposition 28
6.2 Demographics and Medical History 28
6.3 Vital Signs 29
6.4 Baseline and Concomitant Medications 29
7 Interim Analyses and Data Monitoring 29
8 Efficacy Analyses 29
8.1 Primary Efficacy Analysis 29
8.2 Handling of Missing Data 32
8.3 Analysis of Secondary Endpoints 34
8.4 Analysis of Exploratory PRO and Performance Endpoints at Days 90 and 180 34
8.5 Analysis of Weekly Longitudinal PROs 35
8.6 Analysis of Heterogeneity of Treatment Effect (HTE) 36
8.7 Bayesian Analyses 37
9 Analysis Of Medical Visits And Hospitalizations 40

10 Safety analyses 40
10.1 Clinical Laboratory Data 40
11 Biomarker Analyses 41
12 Adherence 41
13 References 42
14 Appendices 43
Appendix 1. RECOVER-VITAL Roster of Statisticians 43
Appendix 2. Prior Specification for Bayesian Analysis of the PGIC 44

LIST OF ABBREVIATIONS

ADaM	Analysis Data Model
AE	Adverse Event
ALT	Alanine transaminase
ANCOVA	Analysis of Covariance
AST	Aspartate transaminase
ATC	Anatomical Therapeutic Classification
BMI	Body Mass Index
CDISC	Clinical Data Interchange Standards Consortium
CI	Confidence Interval
COVID-19	Coronavirus Disease of 2019
CRF	Case Report Form
CRP	C-Reactive Protein
CTCSQ	Cluster Targeted COVID-19 Symptom Questions
DAIDS	Division of AIDS
DCRI	Duke Clinical Research Institute
DILI	Drug-Induced Liver Injury
DSMB	Data and Safety Monitoring Board
DSQ - PEM	DePaul Symptom Questionnaire Post Exertional Malaise
ECOG 2	Everyday Cognition 2
eCRF	Electronic Case Report Form
EOD	End of Dosing
ESI	Events of Special Interest
ESWT	Endurance Shuttle Walk Test
FU	Follow-up
HIV	Human Immunodeficiency Virus
ISWT	Incremental Shuttle Walk Test
LFT	Liver Function Test
LLN	Lower Limit of Normal
MedDRA	Medical Dictionary of Regulatory Activities
MITT	Modified Intention-To-Treat
OHQ	Orthostatic Hypotension Questionnaire
OHDAS	Orthostatic Hypotension Daily Activity Scale
OHSA	Orthostatic Hypotension Symptom Assessment
PASC	Post-acute Sequelae of SARS-CoV-2 Infection
PCR	Polymerase Chain Reaction
PEM	Post-Exertional Malaise

PGIC	Patient Global Impression of Change
PRO	Patient Reported Outcome
PT	Preferred Term
Q1	25th Percentile (1st Quartile)
Q3	75th Percentile (3rd Quartile)
RAT	Rapid Antigen Test
RECOVER	Researching COVID to Enhance Recovery
SAE	Serious Adverse Event
SAP	Statistical Analysis Plan
SAR	Suspected Adverse Reaction
SARS-CoV-2	Severe Acute Respiratory Syndrome Coronavirus 2
SD	Standard Deviation
SDTM	Study Data Tabulation Model
SE	Standard Error
SUSAR	Suspected Unexpected Serious Adverse Reaction
ULN	Upper Limit of Normal

1 STUDY AND DOCUMENT OVERVIEW

RECOVER-VITAL is a platform protocol designed to be flexible so that is suitable for a wide range of settings within health care systems and community settings where it can be integrated into COVID-19 programs and subsequent treatment plans. RECOVER-VITAL is a randomized, controlled, double-blinded, multi-center, multi-arm platform trial which will evaluate different interventions organized as appendices to the protocol. Each appendix will evaluate potential mechanisms of action, efficacy, and safety of antivirals and other therapeutics in individuals with Post-acute Sequelae of SARS-CoV-2 Infection (PASC), according to the platform protocol objectives.

This document describes the planned statistical analyses that will be conducted at the completion of the RECOVER-VITAL study for the first intervention appendix – Appendix A. Once the initial unblinded data review has occurred, only the blinded statistics team members will be allowed to modify the SAP (see Appendix 1 for the list of blinded and unblinded statisticians).

Each study intervention appendix will enroll adult participants who experience at least one of three predominant symptom clusters: cognitive dysfunction, autonomic dysfunction, or exercise intolerance with post-exertional malaise (PEM). Overall enrollment for the platform protocol will depend on the number of screen failures, the number of study intervention appendices that are added, the ability to pool their control groups for analysis, and adjustments to sample size based on study data. Enrollment will begin in July 2023 with Appendix A. Study intervention appendices may be added or removed according to adaptive design and/or emerging evidence.

This study intervention Appendix A is a prospective, multi-center, double-blind, randomized controlled phase 2 trial evaluating nirmatrelvir/ritonavir (PAXLOVID) in two dosing durations for PASC. Within this study, participants will be randomized 1:1:1 to 15 days of active intervention (nirmatrelvir/ritonavir) with 10 days of control (placebo/ritonavir), 25 days of active intervention, or 25 days of control, within the three symptom clusters. The originally targeted sample size of 900 was intentionally exceeded in order to ensure at least 100 participants in each treatment group within symptom cluster. The intervention will be administered in two kits (15 days followed by 10 days). For this appendix, the midpoint of drug dosing is approximately 13 days; the Drug Dosing Midpoint Clinic Visit will occur at Day 15 (-5 days).

Participants will be in the study for approximately 6 months from the time of initiating study drug administration until their end of study visit. At approximately the midpoint of drug dosing, a Drug Dosing Midpoint Clinic Visit will occur. The End of Dosing Clinic Visit will occur after the final intervention dose has been administered. The Follow-Up Period will begin after the End of Dosing Clinic Visit. A follow-up clinic visit will occur for all participants on Day 90 (± 5 days). All participants will receive remote followup approximately weekly with optional in-person visits for blood collection on Days 45 (± 5), 60 (± 5), and 120 (± 5) days. The End of Study Visit will be a remote visit on Day 183 (± 7 days).

Dosing and enrollment will be temporarily suspended pending review of the Data and Safety Monitoring Board (DSMB) if three or more participants experience a serious adverse event (SAE) of the same type that is determined to be related to study intervention(s). The DSMB recommendations will be considered by the NIH and the study Principal Investigator prior to making any decisions regarding appendix continuation or discontinuation.

2 STUDY OBJECTIVES AND ENDPOINTS

2.1 Study Objectives

2.1.1 Primary Objective:

- To evaluate the effect of intervention(s) versus control on symptom-specific patient-reported outcome (PRO) measures within each PASC symptom cluster at Day 90.

2.1.2 Secondary Objectives:

- To evaluate the effect of intervention(s) versus control on performance-based outcomes within each PASC symptom cluster.

2.1.3 Safety Objectives:

- To characterize the safety and tolerability of intervention(s) in PASC.

2.1.4 Exploratory Objectives:

- To investigate mechanisms of potential intervention(s) efficacy.
- To compare the effect of intervention(s) on symptom burden and health related quality of life versus control.
- To evaluate the durability of intervention(s) response.
- To evaluate intervention(s) effect on healthcare utilization.
- To describe the effect and durability of intervention(s) versus control across exploratory performance-based outcomes and PROs within each PASC symptom cluster.
- To determine biomarkers associated with PASC symptom clusters and intervention-associated recovery.

2.2 Endpoints

This section summarizes protocol-defined endpoints as listed in Protocol Table 1 and documents associated key additional pre-specified definitions and implementation details. Some differences between endpoint descriptions below versus the protocol are described in Section 2.2.4 below.

2.2.1 Primary Endpoints

The primary endpoint analysis will evaluate improvement in symptom-specific patient-reported outcome measures within each PASC symptom cluster at Day 90. There are 3 joint primary endpoints corresponding to 3 symptom clusters:

- Improvement in patient-reported cognition at Day 90: Increase by at least 5 T-score points on the PROMIS-cognitive 8a at Day 90 compared to baseline in the cognitive dysfunction symptom cluster. To reduce variability, an average of 3 weekly assessments closest to Day 90 will be used.
- Improvement in patient-reported orthostatic symptoms at Day 90: Decrease by at least 1 rating scale category on modified Orthostatic Hypotension Questionnaire (OHQ) Question #1 at Day 90

compared to baseline in the autonomic dysfunction symptom cluster. To reduce variability, an average of 3 weekly assessments closest to Day 90 will be used.

- Improvement in patient-reported post-exertional malaise (PEM) at Day 90: Absence of any symptoms of moderate or greater severity occurring with 50% or more frequency as assessed by a modified version of the DePaul Symptom Questionnaire Post-Exertional Malaise (DSQ-PEM) short form at Day 90 in the exercise intolerance symptom cluster.

For each pairwise comparison between an intervention and control group, the primary null hypothesis to be tested is that the probability of meeting a specified degree of improvement in symptom clusterspecific primary outcome measures (including the DSQ-PEM, PROMIS-cognitive 8a, or OHQ) at Day 90 compared to baseline is the same for intervention and control. The alternative hypothesis is that the probability of meeting a specified degree of improvement in symptom cluster-specific primary outcome measures at Day 90 compared to baseline differs for intervention and control.

2.2.2 Secondary Endpoints

- Improvement in cognitive performance outcome at Day 90: Increase by at least 1 standard deviation (SD) in either or both of the WHO/UCLA Auditory Verbal Learning Test (AVLT) delayed recall measure and/or Symbol Digit Modalities Test number of correct substitution measure, and no decrease exceeding 0.15 SD in either of the above measures, at Day 90 in the cognitive dysfunction symptom cluster
- Improvement in orthostatic performance outcome at Day 90: Improvement by a specified threshold in at least one of the following 3 measures from the active stand test and no worsening in any of the following 3 measures from the active stand test at Day 90 compared to baseline in the autonomic dysfunction symptom cluster:
  - ○ 10-minute heart rate change
    - Improvement: Decrease by at least 10 beats per minute from baseline to Day 90
    - Worsening: Any increase from baseline to Day 90 ○ 10-minute systolic blood pressure change
    - Improvement: Increase by at least 10mmHg from baseline to Day 90
    - Worsening: Any decrease from baseline to Day 90 ○ 10-minute diastolic blood pressure change
    - Improvement: Increase by at least 5mmHg from baseline to Day 90
    - Worsening: Any decrease from baseline to Day 90
- Improvement in exercise performance outcome at Day 90: Increase by at least 3 minutes in walking time on the Endurance Shuttle Walk Test (ESWT) at Day 90 compared to baseline in the exercise intolerance symptom cluster.

The null hypothesis to be tested is that there is no difference between intervention and control group.

2.2.3 Exploratory and Safety Endpoints

Efficacy

-
- PROMIS cognitive 8a T-score as continuous variable at days 90, 180, and longitudinally in the cognitive dysfunction symptom cluster
- OHQ Question #1 as continuous variable at days 90, 180, and longitudinally in autonomic dysfunction symptom cluster
- DSQ-PEM as continuous outcome at days 90, 180, and longitudinally in exercise intolerance symptom cluster

  Neurocognitive battery outcomes (continuous) at day 90 in the cognitive dysfunction symptom cluster: AVLT total learning score, AVLT delayed recall score, AVLT delayed recognition score, SDMT number of correct substitutions
- Change in blood pressure and heart rate on active stand test at day 90 in the autonomic symptom cluster
- ESWT walk time as continuous variable at day 90 in the exercise intolerance symptom cluster
- Frequency and severity of individual symptoms at days 90 and 180 as assessed by PASC symptom questionnaire
- PROMIS-29+2 physical function summary score at days 90 and 180; other PROMIS-29+2 measures at days 90 and 180
- Patient global impression of change (PGIC) at days 90 and 180
- ECOG-2 Overall score at days 90 and 180 in cognitive dysfunction symptom cluster
- COMPASS-31 overall score and subscales at days 90 and 180 in the autonomic symptom cluster
- OHQ overall score and subscales at days 90 and 180 in the autonomic symptom cluster
- Endpoints derived from full autonomic testing at select sites (details to be developed in a separate SAP)
- Endpoints derived from cardiopulmonary exercise testing at select sites (details to be developed in a separate SAP)
- Healthcare utilization ○ Number of cumulative medical visits in intervention(s) vs. control ○ Number of cumulative hospitalizations in intervention(s) vs. control

Safety

- Occurrence of individual SAEs
- Occurrence of any one or more SAE
- Occurrence of adverse events (AEs) and SAEs leading to discontinuation
- Occurrence of events of special interest (ESIs)

Adherence

- Number of missed doses

Biomarkers

- Viral clearance, persistence, and recurrence
- Immune dysfunction
- Coagulation
- Biologic system functioning

•

2.2.4 Differences in Above List Compared to Protocol

- The protocol does not specify which summary scores are to be calculated and analyzed for outcomes such as PROMIS-29, COMPASS-31, OHQ, neurocognitive battery, and active stand test. Similarly, it does not specify criteria for "meeting pre-specified change from baseline to Day 90 on cluster-specific performance outcome" for the construction of performance-based secondary endpoints. These details have been added above.

  Protocol Table 1 only lists efficacy endpoints that are to be analyzed at Day 90 and end of study. The list above pre-specifies a set of endpoints that are also to be analyzed across multiple time points longitudinally.
- The SAP uses "day 180" as short-hand for the protocol-defined end of study assessment.

3 STATISTICAL DESIGN

3.1 Power and Sample Size

This study uses an adaptive platform trial design that will allow study interventions to be added or dropped from consideration based on accruing evidence of futility or efficacy. In such a design, the required sample size depends on both the number of interventions tested and the ability to pool their control arms for analysis. Initial sample size estimates are based on a single appendix with a 1:1:1 allocation to each of two dose levels of an active intervention and control. If additional study interventions are added later that can contribute to pooled control, the sample size will be adjusted accordingly.

The sample size is based on a design that will evaluate treatment effects separately within three coprimary symptom cluster populations (cognitive dysfunction, autonomic dysfunction, and exercise intolerance) with a Holm-type adjustment to control the overall type-I error across them. The Holm procedure will initially allocate alpha equally across symptom clusters (0.0166, 0.0166, 0.0166), with a total type-I error probability of 0.05. If the null hypothesis is initially rejected for some but not all symptom clusters, the Holm procedure will allow the non-rejected hypotheses to be re-tested at an updated, less stringent significance level.

Accounting for the Holm procedure's stepwise alpha selection in power calculations is complicated because the final significance threshold is partly random and depends on the unknown configuration of treatment effects across all 3 symptom clusters considered jointly. As a simple conservative approximation, power was calculated for each symptom cluster individually assuming a specified treatment effect for that single symptom cluster and assuming 0.0166 for the Holm procedure's final significance threshold.

Analysis within each symptom cluster population will control the type-I error across multiple dose levels by analyzing them in a pre-specified hierarchical testing procedure in order from highest to lowest dose.

•

For simplicity, the total sample size was obtained by calculating sample size for a simple two-group comparison and multiplying by 3/2.

Sample size was chosen to provide 80% power to detect a difference of 25 percentage points in the proportion of participants who experience the binary primary outcome for each pairwise comparison between a treatment group and control within each symptom cluster, assuming a two-sided test with alpha = 0.0166. Specifically, a sample size of 82 per group yields 80% power assuming the true outcome probabilities are 37.5% in the control group and 62.5% in the active treatment group. This sample size was inflated by 1/0.82 = 1.22 to account for potential 18% dropout/loss to follow-up.

The final required sample size per symptom cluster was calculated as 82 x 1.22 = 100 participants per intervention group (300 total per symptom cluster). Enrollment within a symptom cluster will continue until the required sample size of 100 participants is met for each intervention group.

Table 1 illustrates the magnitude of treatment effects that will be detectable with 80% or 90% power under the study's planned sample size if the primary outcome occurs with probabilities ranging from 0.05 to 0.35 in the placebo group.

Table 1. Minimum treatment effects for 80% or 90% power

Probability of Improvement with Placebo	Minimum Treatment Effect Difference for 80% Power*	Minimum Treatment Effect Difference for 90% Power*
0.05	0.17 (0.22 vs 0.05)	0.20 (0.25 vs 0.05)
0.10	0.20 (0.30 vs 0.10)	0.23 (0.33 vs 0.10)
0.15	0.22 (0.37 vs 0.15)	0.25 (0.40 vs 0.15)
0.20	0.23 (0.43 vs 0.20)	0.27 (0.47 vs 0.20)
0.25	0.24 (0.49 vs 0.25)	0.28 (0.53 vs 0.25)
0.30	0.25 (0.55 vs 0.30)	0.28 (0.58 vs 0.30)
0.35	0.25 (0.60 vs 0.35)	0.28 (0.63 vs 0.35)

* Power for pairwise comparison between single dose level of active intervention versus control assuming 100 participants per treatment group (82 participants per treatment group with non-missing outcome data) and two-sided alpha = 0.0166.

3.2 Randomization Methods

A two-step randomization process will be used to achieve blinding and equitable randomization probability.

At the first step, each participant will be assigned with equal probability to one of the appendices for which the participant is eligible after applying any appendix-level exclusion criteria. At the second step, if the appendix includes k dose levels of active intervention plus a control group, participants will be assigned with probability $1/(k+1)$ to one of the $(k+1)$ available treatment options.

For simplicity, the text below assumes that M_1 active appendices will have no sharing of control participants and that an additional M_2 active appendices each involve a single intervention dose level and will use a single pooled control group among them. Let k_i be the number of dose levels in the i-th appendix where no restriction is placed on $k_1, \ldots, k_{M1}$ and $k_{M1+1} = k_{M1+2} = \cdots = k_{M1+M2} = 1$. Randomization will proceed as follows for a participant who is eligible for m_1 of the M_1 appendices with no sharing of controls and m_2 of the M_2 appendices with sharing of controls. At the first stage, the participant will be assigned with probability $1/(m_1 + m_2)$ to one of the appendices for which the participant is eligible. If the randomly assigned intervention is unable to use a pooled control group, the second stage randomization will assign the participant with probability $1/(k_i + 1)$ to one of the available intervention groups, where i denotes the randomly assigned appendix. If the randomly assigned intervention is one that will use a pooled control group, the second stage randomization will assign the participant to active intervention or control at an m_2:1 ratio, where m_2 is defined above.

Sites will be informed to which study intervention appendix the participant is randomized, but not whether they are allocated to one of the active intervention dose levels or control within that appendix. The participants and investigators will be blinded throughout the study.

4 STUDY ASSESSMENTS AND DEFINITIONS

4.1 Study Assessments

Participants will be followed for approximately 6 months from time of randomization and treatment initiation. Clinic visit assessments will occur for all participants at baseline and at approximately day 13 (window, 10-15), day 25 (end of dosing visit), and day 90 (primary outcome assessment). All participants will also receive remote follow-up approximately weekly with and will be offered optional in-person visits for blood collection on Days 45 (± 5), 60 (± 5), and 120 (± 5) days. A remote end of study visit will occur on day 183 (± 7 days). The collection schedule for selected key study data is summarized in Table 2.

Table 2. Abbreviated schedule of assessments (selected items)

**Visit**	**Screen/ Baseline**	**Remote: Weekly Assessments**	**Clinic: Dosing Midpoint**	**Clinic: End of Dosing**	**Clinic: Primary Outcome**	**Remote: End of Study**
Target day of visit	0	Weekly	15**	25**	90**	183**
Window (days)	-2 to 0	*	10 to 15	± 3	± 5	± 7
PROMIS 8a (cognitive)	x	x	x	x	x	x
ECOG2 (cognitive)	x	x	x	x	x	x
OHQ (autonomic)	x	x	x	x	x	x
COMPASS-31 (autonomic)	x	x	x	x	x	x
DSQ-PEM (exercise)	x	x	x	x	x	x
PROMIS-29+2	x	x	x	x	x	x
PASC symptom questionnaire	x	x	x	x	x	x
CTCSQ (including PGIC)	x			x	x	x
Neuro battery (cognitive)	x			x	x	
Active stand (autonomic)	x			x	x	
ESWT (exercise)	x			x	x	
Healthcare utilization			x	x	x	x
Blood/biomarkers***	x		x	x	x	
Safety	x		x	x	x	x
Adherence			x	x		

* All assessments that are completed remotely on a weekly basis will be open to the participant to complete for 5 days. Any assessments that are not completed within that window will expire and will not be collected.
** For the purposes of efficacy analyses, we will use data from the following visit windows: day 15 (± 3), day 25 (± 3), day 90 (± 14), day 183 (± 28).
*** Not shown in table: optional clinic visits for optional blood collection on days 45 (± 5), 60 (± 5), and 120 (± 5).

4.2 Symptom Cluster Assignment

Assignment of participants into symptom clusters will be based on assessments performed at the screening and baseline visit(s). An indicator of eligibility for each symptom cluster will be derived programmatically within the EDC based on the data entered. Prior to Protocol version 4.0, participants who were eligible for multiple symptom clusters were enrolled into to all clusters for which eligibility criteria were met. Beginning with Protocol version 4.0, participants who meet eligibility criteria for multiple clusters will be enrolled in the cluster for which they exhibit the most severe criteria. Screening for symptom cluster eligibility will begin with administration of the Cluster Targeted COVID-19 Symptom Questions (CTCSQ). As detailed below, the CTCSQ assesses presence and severity of twelve symptoms with response options of none, mild, moderate, or severe. Participants who report having at least two moderate symptoms from the same symptom cluster or one severe symptom from a symptom cluster will undergo additional assessments to determine eligibility for that cluster (see Figure 1). *Figure 1. Symptom cluster assignment flow*

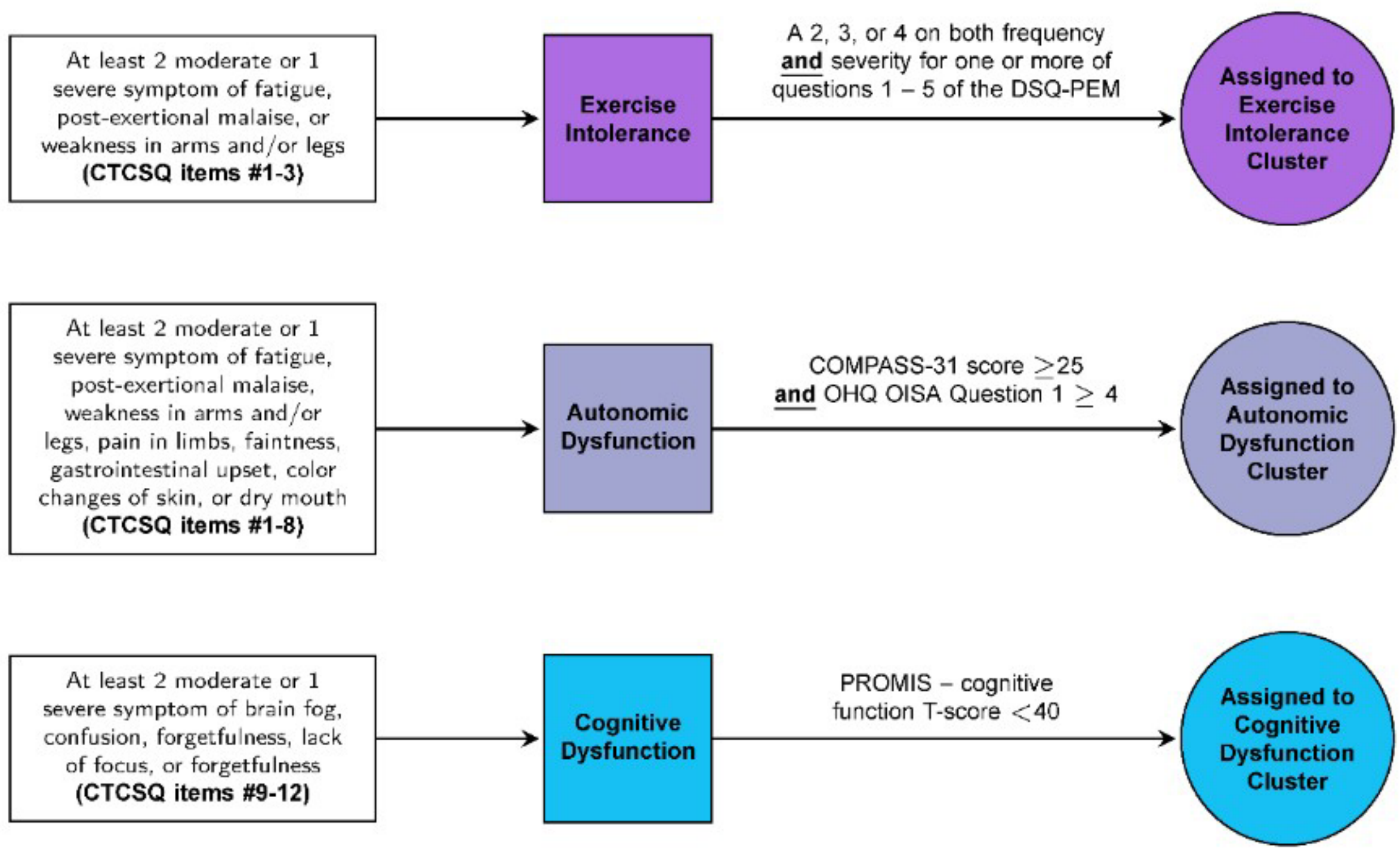

4.2.1 Cognitive dysfunction symptom cluster: eligibility

- At least two moderate or one severe rating within CTCSQ items 9-12 ○ CTCSQ items 9-12: brain fog; confusion; forgetfulness; difficulty focusing
- PROMIS – cognitive 8a function T-score < 40

4.2.2 Autonomic dysfunction symptom cluster: eligibility

- At least two moderate or one severe rating within CTCSQ items 1-8 ○ CTCSQ items 1-8: fatigue; post-exertional malaise; weakness in arms and/or legs; pain in limbs; faintness; gastrointestinal upset; color changes of the skin; dry mouth
- COMPASS 31 score ≥ 25
- OHQ OHSA Question #1 ≥ 4

4.2.3 Exercise intolerance symptom cluster: eligibility

- At least two moderate or one severe rating within CTCSQ items 1-3 ○ CTCSQ items 1-3: fatigue; post-exertional malaise; weakness in arms and/or legs
- DSQ-PEM: at least one moderate or severe symptom occurring with at least 50% frequency

4.2.4 Cluster-Targeted COVID Symptom Questionnaire (CTCSQ)

The CTCSQ was created for RECOVER-VITAL as an eligibility screening tool for symptom clusters. The first twelve items of CTCSQ assess presence and severity of symptoms related to cognition, autonomic function, and exercise symptoms using response options of none, mild, moderate, or severe:

1. Fatigue (being tired)
2. Post-exertional malaise (symptoms worse after even minor physical or mental effort)
3. Weakness in your arms and/or legs
4. Pain in your limbs
5. Faintness (light-headedness)
6. Gastrointestinal upset
7. Color changes of the skin
8. Dry mouth
9. Problems with thinking or concentrating (brain fog)
10. Confusion
11. Forgetfulness
12. Difficulty focusing

If a participant reports a moderate or severe item #1-3, they will complete the exercise intolerance and autonomic dysfunction cluster assessments at Baseline. If a participant reports a moderate or severe item #1-8, they will complete the autonomic dysfunction cluster assessments at Baseline. If a participant reports a moderate or severe item #9-12, they will complete the cognitive dysfunction cluster assessments at Baseline.

In addition to assessing symptom cluster-specific eligibility, the above items will be used descriptively to track changes in symptoms during follow-up. After randomization, CTCSQ will be performed at the end of dosing visit, day 90 visit, and end of study visit.

The final CTCSQ item (#13) is the Patient Global Impression of Change (PGIC). The purpose of this item is fundamentally different than the others and is described in a separate section below. It is not associated with specific symptom clusters and does not play a role in symptom cluster assignment.

4.3 Definition of Baseline

Baseline assessments will preferably occur on the same day as the first study dose administration (i.e., the day of randomization) and may occur on the same day as screening. In all cases, randomization and administration of first study dose are expected to occur within 48 hours of baseline assessments. For efficacy analyses of the primary, secondary, and exploratory endpoints, baseline will be defined as the closest observation on or prior to the date of randomization. Such assessments may be recorded during the Screening or Baseline clinic visits, or remotely via Medidata's electronic clinical outcome assessment (eCOA) platform. For safety analyses, baseline will be defined as the closest observation on or prior to the date of first study dose administration.

Baseline concomitant medications will include all concomitant therapies taken by the participant within 14 days prior to informed consent. Data collected at baseline will include, among others:

- Demographics
- Height and weight
- Blood pressure and pulse, supine and standing
- Medical History; including SARS-CoV-2 vaccination status and dates, SARS-CoV-2 test result date (if available), signs and symptoms, and treatment (including hospitalization, Intensive Care Unit (ICU) status, supplemental O2 status) and PASC history (symptoms and duration)
- Medications taken within 14 days prior to informed consent
- Pre-treatment values of efficacy variables (PRO and performance measures) and safety variables (labs)
- Blood and stool collection for mechanistic evaluation

4.4 Efficacy Assessments

4.4.1 PROMIS Cognitive 8a

PROMIS Cognitive 8a is a questionnaire assessing self-reported cognitive impairments over the past 7 days using 8 items. It assesses the frequency that respondents experienced the following impairments on a scale ranging from 5 (never) to 1 (very often; several times a day) for the following items:

- PC2r. My thinking has been slow
- PC35r. It has seemed like my brain was not working as well as usual
- PC36r. I have had to work harder than usual to keep track of what I was doing
- PC42r. I have had trouble shifting back and forth between different activities that require thinking
- PC8r. I have had trouble concentrating
- PC25r. I have had to work really hard to pay attention or I would make a mistake
- PC1r. I have had trouble forming thoughts
- PC5r. I have had trouble adding or subtracting numbers in my head

The total raw score is transformed into a T-score, with higher scores representing better cognitive function. The T scores are interpreted in relation to a US reference population and are scaled to have mean = 50 and SD = 10 in the reference population.

The PROMIS-cognitive 8a will be performed as part of screening for the cognitive dysfunction symptom cluster. For participants who qualify for the cognitive dysfunction cluster, it will be collected at baseline, remotely on a weekly basis from randomization until end of study, and at in-person follow-up clinic visits.

The primary endpoint for the cognitive dysfunction symptom cluster is improvement of at least 5 Tscore points on the PROMIS-cognitive 8a as measured at Day 90 compared to baseline. The 5-point threshold is based on a body of literature that has specifically evaluated the minimally important difference using distribution- and anchor-based approaches across a number of disease processes (e.g., cancer, stroke, low back pain, osteoarthritis) and PROMIS measures (e.g., pain intensity, depression, physical functioning, anxiety, fatigue, etc.). These varied populations consistently report a minimally important difference of 2 to 6. [1-3] Moreover, a value of 5 represents one-half the standard deviation for PROMIS measures in the US general population and has been recommended as a starting point when no empirical data are available. [4,5]

4.4.2 Modified Orthostatic Hypotension Questionnaire (OHQ)

The Orthostatic Hypotension Questionnaire (OHQ) is a patient reported outcome designed to assess the severity and impact of orthostatic hypotension (OH), a condition characterized by a sudden drop in blood pressure when standing. The OHQ consists of two main components: the Orthostatic Hypotension Symptom Assessment (OHSA) and the Orthostatic Hypotension Daily Activity Scale (OHDAS). The OHSA consists of 6 items measuring severity on a scale ranging from 0 (none) to 10 (worst possible) for the following symptoms:

1. Dizziness, lightheadedness, feeling faint, or feeling like you might black out.
2. Problems with vision (blurring, seeing spots, tunnel vision, etc.) 3. Weakness.
4. Fatigue.
5. Trouble concentrating.
6. Head and neck discomfort.

The OHDAS assesses the extent to which OH interferes with daily life on a scale ranging from 0 no interference) to 10 (total interference) for each of the following activities:

7. Activities that require standing for a short time.
8. Activities that require standing for a long time.
9. Activities that require walking for a short time.
10. Activities that require walking for a long time.

The OHQ questionnaire for RECOVER-VITAL has been modified slightly compared to the original OHQ. The instruction wording was changed to refer to "orthostatic intolerance" instead of "orthostatic hypotension" and "low blood pressure".

The OHQ will be performed as part of screening for the autonomic dysfunction symptom cluster. For participants who qualify for the autonomic dysfunction symptom cluster, it will be collected at baseline, remotely on a weekly basis from randomization until end of study, and at in-person follow-up clinic visits.

The primary endpoint for the autonomic dysfunction symptom cluster is improvement as defined by at least a 1-point decrease in the response to OHQ question 1 at Day 90 compared to baseline. At least 1point change on question 1 has been determined to be clinically meaningful and resulted in previous updates to the FDA drug label for orthostatic hypotension associated with Parkinson's disease.

Other summary measures for OHSA, OHDAS, and overall OHQ are calculated by averaging across items within OHSA, OHDAS, and overall respectively. Scores range from 0 to 10 with higher values indicating worse impairment.

Slightly different scoring rules will be used in the construction of summary measures at baseline compared to follow-up assessments. At baseline, the baseline OHSA composite score will be calculated as an average of items 1-6 and the baseline OHDAS composite score will be calculated as an average of items 7-10. The baseline overall OHQ composite score will be an average of the baseline OHSA and baseline OHDAS composites.

At follow-up, the above scoring rules will be modified by only averaging across items that were non-zero at baseline. Thus, the follow-up the follow-up OHSA composite score will be calculated by averaging across the subset of items 1-6 that were non-zero at baseline. The follow-up OHDAS composite score will be calculated by averaging across the subset of items 7-10 that were non-zero at baseline. The follow-up overall OHQ composite score will be an average of the follow-up OHSA and follow-up OHDAS composites.

4.4.3 Modified DePaul Symptom Questionnaire Post Exertional Malaise (DSQ-PEM)

The DePaul Symptom Questionnaire Post Exertional Malaise (DSQ-PEM) is a patient-reported outcome designed to assess the frequency and severity of symptoms worsening after physical or mental exertion. The first 10 items of DSQ-PEM assess frequency and severity of the following 5 exercise-related impairments:

1. Dead, heavy feeling after starting to exercise,
2. Next day soreness or fatigue after non-strenuous, everyday activities
3. Mentally tired after the slightest effort 4. Minimum exercise makes you physically tired 5. Physically drained or sick after mild activity.

These items were modified for the current study to use a 7-day instead of 6-month look back period. Frequency is rated on a 5-point Likert scale: 0 = none of the time, 1 = a little of the time, 2 = about half the time, 3 = most of the time, and 4 = all of the time. Severity is also rated on a 5-point Likert scale: 0 = symptom not present, 1 = mild, 2 = moderate, 3 = severe, 4 = very severe.

Based on recommended scoring rules, Post-Exertional Malaise (PEM) will be deemed to be present for participants reporting at least one moderate (rated severity ≥ 2) PEM symptom for a frequency rated ≥ 2.

The DSQ-PEM will be performed as part of screening for the exercise intolerance symptom cluster. For participants who qualify for the exercise intolerance cluster, the DSQ-PEM will be assessed at baseline, remotely on a weekly basis from randomization until end of study, at in-person visits, and 1 to 2 days after each visit where performance outcomes are collected. For all other participants, it will be collected 1 to 2 days after each visit where performance outcomes are collected.

The primary endpoint for the exercise symptom cluster is improvement in PEM, defined as having no symptoms of moderate or greater severity with 50% or more frequency as determined by the DSQ-PEM short form at Day 90. We will use a conservative approach to defining “no symptoms of moderate or greater severity” and “no symptoms with 50% or more frequency”. If the frequency or severity of any of the 5 symptoms are missing, improvement in PEM will be considered missing.

The PEM measure defined above is binary. An analogous continuous measure will be obtained by counting the number of symptoms for which PEM criteria are met, i.e., the number of symptoms with at least moderate severity occurring with at least 50% frequency.

4.4.4 Modified Everyday Cognition 2 (ECOG 2)

The Everyday Cognition 2 (ECOG 2) is a self-report, 41-item questionnaire used to measure a participant’s perceived capacity to perform activities related to cognitive function, which could impact major activities of daily living and independence. It has been used for patients with mild cognitive impairment, Alzheimer’s Disease, and dementia. Assessments are organized within 7 domains corresponding to memory (9 items), language (9 items), visual-spatial and perceptual Abilities (8 items), executive function – planning (5 items), executive function – organization (6 items), and executive function – divided attention (4). The original ECOG-2 asks respondents to assess abilities now compared to 10 years ago on a scale ranging from 1 (better or no change) to 4 (consistently much worse), with an additional response option for "don't know or not applicable".

For the current study, the questionnaire has been modified to use a 7-day look back and to assess current status without reference to a specific baseline. Response options range from 1 (no difficulty) to 5 (great difficulty/unable to do), with a separate option for "not applicable". For items that are applicable, participants are asked to indicate whether this is different compared to before COVID. The final ECOG 2 summary score is calculated as an average across all questionnaire items that are marked as "applicable".

The ECOG 2 will be collected for participants in the cognitive dysfunction symptom cluster. It will be assessed at baseline, remotely on a weekly basis from randomization until end of study, and at in-person follow-up clinic visits.

4.4.5 The Composite Autonomic Symptom Score 31 (COMPASS-31)

COMPASS-31 is a patient-reported instrument designed to evaluate the presence and severity of autonomic symptoms. It encompasses six domains, including orthostatic intolerance, vasomotor, secretomotor, gastrointestinal, bladder, and pupillomotor. Each domain has a set of questions that are scored on a five-point scale, and the final score is obtained by summing weighted scores of all domains. The total score ranges from 0 to 100, with higher scores indicating a higher severity of autonomic symptoms.

COMPASS-31 will be performed as part of screening for the autonomic dysfunction symptom cluster. For participants who qualify for the autonomic dysfunction symptom cluster, it will be collected after randomization at in-person visits and remotely on a weekly basis from randomization until end of study.

4.4.6 PROMIS-29+2 Profile v2.1.

The PROMIS-29+2 Profile v2.1 is a patient-reported outcome measure designed to assess overall health and functioning. It includes 4 items per domain for each of 7 domains (physical function, anxiety, depression, fatigue, sleep disturbance, ability to participate in social roles and activities, pain

interference), plus an item for pain intensity and 2 items for cognitive function. Pain intensity is assessed on scale ranging from 0 (no pain) to 10 (worst pain imaginable). All other items use Likert-type 5-point scales. Scoring of the PROMIS-29+2 will follow established guidelines [6]. Domain-specific summary scores will be expressed as T-scores with higher scores representing a worse condition for domains measuring impairment (e.g., anxiety, depression, fatigue, sleep disturbance, and pain interference) and representing a better condition for domains measuring function (e.g., physical function, ability to participate in social roles and activities). T scores are interpreted in relation to a US general reference population with population mean = 50 and SD = 10.

The PROMIS-29+2 Profile v2.1 will be collected at baseline, remotely on a weekly basis from randomization until end of study, and at in-person follow-up clinic visits.

4.4.7 Patient Global Impression of Change (PGIC)

The PGIC asks participants to assess how the overall severity of their long COVID symptoms have changed compared to the start of the study. The question wording is: "Compared to the start of the study, the overall severity of your Long COVID symptoms have___". Response choices are: (5) Improved a lot, (4) Improved somewhat, (3) Stayed about the same, (2) Worsened somewhat, (1) Worsened a lot.

The PGIC has been incorporated into the CTCSQ and appears as Question #13 on the CTCSQ form. It will be collected at baseline as part of the screening CTCSQ, at the end of dosing visit, day 90 visit, and end of study visit.

4.4.8 PASC Symptom Questionnaire

Version 1 of the PASC Symptom Questionnaire assesses the frequency and severity of 33 individual symptoms. Frequency is assessed on Likert-type scale ranging from 1 (never) to 5 (almost constantly). Severity is assessed on a 4-level scale ranging from 1 (mild) to 4 (very severe). Version 2 of the PASC Symptom Questionnaire was introduced in July 2024; 6 severity questions and 1 frequency question were removed, wording of some of the questions was updated, and frequency and severity scales were updated. The questionnaire is collected at baseline, remotely on a weekly basis from randomization until end of study, and at in-person follow-up clinic visits.

4.4.9 Neurocognitive battery (AVLT/SDMT)

The Neurocognitive battery is a performance measure used to assess various elements related to cognition. The neurocognitive battery consists of a cognitive assessment sequence of the following: WHO/UCLA Auditory Verbal Learning Test (WHO/UCLA AVLT) and Symbol Digit Modalities Test (SDMT).

Three AVLT scores will be calculated:

- Total learning score: calculated as the total sum of correct responses from tests #1 through #5
- Delayed recall score: calculated as the number of correct responses from test #8
- Delayed recognition score: calculated as the number of correct recognitions minus false positives from test #9.

One SDMT score will be calculated:

- Number of correct substitutions.

The neurocognitive battery will be performed for participants in the cognitive dysfunction symptom cluster at baseline, end of dosing visit, and day 90. For analysis, the above scores will be converted into Z-scores by subtracting the overall study mean and dividing by the SD. The major secondary endpoint for the cognitive dysfunction symptom cluster is a binary endpoint defined as an increase by at least 1 point in either or both of the AVLT delayed recall Z-score and/or SDMT number of correct substitutions Zscore, and no decrease exceeding 0.15 in either of these measures, at Day 90 compared to baseline.

4.4.10 Active Stand Test

The active stand test is performed to assess presence of orthostatic intolerance, orthostatic hypotension, and postural orthostatic tachycardia syndrome. Participants are asked to lie down for 5 minutes and then stand for 10 minutes. The participant's blood pressure and heart rate are recorded after 5 minutes of lying and then at minutes 1, 3, 5, and 10 after standing. The Vanderbilt Orthostatic Symptom Score, a 9 point scale of orthostatic symptoms rated on a scale of 0 (no symptom) to 10 (worst the participant has experienced), will also be obtained after 5 minutes of lying and then at 10 minutes after standing. Changes in systolic blood pressure (SBP), diastolic blood pressure (DBP), and heart rate beats per minute (HR BPM) will be assessed from lying to standing for 10 minutes. The main summary measures will be changes assessed at 10 minutes as follows:

- ΔHR = (HR BPM at 10 minutes standing) minus (HR BPM at 5 minutes lying)
- ΔSBP = (SBP at 10 minutes standing) minus (SBP at 5 minutes lying) •
  ΔDBP = (DBP at 10 minutes) minus (DBP at 5 minutes lying).

The active stand test will be performed among participants in the autonomic dysfunction cluster at baseline, end of dosing visit, and Day 90.

Trial endpoints will be constructed by comparing values of ΔHR, ΔDBP, and ΔDBP at follow-up compared to baseline, i.e., by calculating differences in the above differences. The major secondary endpoint for the autonomic dysfunction cluster is a binary endpoint defined as improvement in at least one of ΔHR, ΔDBP, or ΔDBP from baseline to Day 90 (defined as an increase of at least 10mmHg in ΔSBP, an increase of at least 5mmHg on ΔDBP, or a decrease of at least 10 BPM on ΔHR) and no worsening in any of ΔHR, ΔDBP, or ΔDBP from baseline to Day 90 (defined as any decrease in ΔSBP or ΔDBP, or any increase in ΔHR).

4.4.11 Endurance Shuttle Walk Test (ESWT).

The ESWT consists of timed walking on a 10 meter course. In order to determine walking speed for the ESWT, it will be performed in combination with the incremental shuttle walk test (ISWT) at baseline. Walking speed for ESWT at baseline and follow-up will be 85% of the maximum walking speed achieved on the ISWT at baseline. The final ESWT result will be expressed as total walking time after an initial 2minute warm-up. The major secondary endpoint for the exercise intolerance symptom cluster is an increase of at least 3 minutes in walk time at Day 90 compared to baseline.

The ESWT will be performed for participants in the exercise intolerance cluster at baseline, end of dosing visit, and Day 90.

4.4.12 Blood for clinical and mechanistic assessments

Blood for clinical laboratory assessments and mechanistic evaluation will be performed at baseline, middosing visit, end of dosing visit, and day 90 visit. Optional blood draws will occur at optional visits

around days 45, 60, and 120. For baseline clinical laboratory assessments, prior assessments within 3 months of study enrollment may be used. Antigenemia will be defined as detection of SARS-CoV-2 spike protein as determined by an external core lab using the Quanterix Simoa platform.

5 GENERAL ANALYSIS CONSIDERATIONS

5.1 Data Sources

At each study site, data will be entered on the electronic Case Report Forms (eCRFs) stored in Medidata Rave. Biomarker data will be collected by NIH-selected laboratories and will be integrated with the clinical database. Prior to database lock, programmed computer edit checks will be run against the database to identify discrepancies and verify reasonableness of the data. Queries to resolve discrepancies will be generated and resolved by the sites. As needed, DCRI Statistics will be able to directly download the eCRF database from Rave using SAS on Demand as Clinical Data Interchange Standards Consortium Study Data Tabulation Model (CDISC SDTM version 3.2) datasets. CDISC Analysis Data Model (ADaM version 2.1) datasets will be created by DCRI Statistics for production of final tables, figures, and listings. All planned reporting will be based off of CDISC datasets, but in the case of emergent safety data, some reporting may occur from the raw eCRF data. All programs written to create analysis datasets and perform analyses will be validated according to SOPs established by the DCRI Statistical Programming group.

5.2 Software

The statistical analyses described in this SAP, as well as production of tables, listings, and figures will be performed using SAS®, version 9.4 or higher (SAS Institute, Cary, NC). Additional statistical software may be used as needed.

5.3 Verification of Results

All tables, listings, and graphs will be verified and reviewed before considered final. The verification process will ensure that the numbers are produced by a statistically valid method and that the execution of the computations is correct. Qualified statisticians or statistical programmers employed by the DCRI who have not been previously involved in the production of the original programming will perform the verification procedures. Methods of verification include independent programming, prior to issuance of the draft statistical report, of all analysis datasets/ADaM and comparison to data listings. Tables, listings, and figures will be reviewed for accuracy, consistency with this analysis plan, consistency within tables/listings/figures, and consistency with corresponding output. Once verification is complete, all documentation of the verification process will be filed as required by the Statistical Standard Operations Procedures of the DCRI.

5.4 Populations for Analysis

The primary analysis will be based on a separate modified intent-to-treat (mITT) population defined for each symptom cluster population. Each cluster-specific mITT population will consist of all randomized participants who receive at least part of one dose of the study intervention or control and who are eligible, enrolled, and randomized into that symptom cluster population. Participants will be analyzed according to their randomly assigned intervention group. Participants who were enrolled into > 1 symptom cluster, as allowed under Protocol Version 3.0, will be included in the mITT population for

each cluster for which inclusion criteria were met. The overall mITT population will consist of participants included in at least one cluster-specific mITT population.

The primary analysis will be repeated in the high study drug adherence population consisting of all mITT participants who missed no greater than 3 planned doses during their entire drug dosing period.

Safety analyses will be based on a safety population consisting of all randomized participants who receive at least part of one dose of the study intervention or control. Participants who are randomized and treated will be included safety analyses irrespective of their eligibility for efficacy analyses.

5.5 Multiple Testing

5.5.1 Primary Endpoints

A sequential closed testing procedure will be used to control the type-I error probability at 0.05 across primary endpoint analyses performed for each combination of 2 dose durations and 3 symptom clusters. Analyses for the two dose durations will be performed sequentially. The first set of comparisons will be between the 25-day dose duration group and control. As detailed below, a Holm-type multiplicity adjustment will be used to control the type-I error probability at 0.05 across the 3 symptom clusterspecific primary outcomes. If all 3 comparisons are significant, then testing of primary outcomes will proceed to 15-day dose duration. Otherwise, testing will stop and all 15-day analyses will be regarded as exploratory.

Figure 2 illustrates the trial's closed testing procedure for the primary outcomes. It is intended to control the type-I error probability at 0.05 across primary endpoint analyses performed for each combination of 2 dose durations and 3 symptom clusters). There are 6 primary endpoint hypotheses to be tested. They are denoted as $\{H_{11}, H_{12}, H_{13}, H_{21}, H_{22}, H_{23}\}$, where H_{dc} corresponds to dose duration d (1=25 days, 2=15 days) and symptom cluster c (1=cognitive, 2=autonomic, 3=exercise). Hypotheses are grouped into a family $\mathcal{F}_1 = \{H_{11}, H_{12}, H_{13}\}$ for the 25-day dose duration and a family $\mathcal{F}_2 = \{H_{21}, H_{22}, H_{23}\}$ for the 15day dose duration. Testing will begin with $\mathcal{F}_1$ (25-day) and will only proceed to $\mathcal{F}_2$ (15-day) in the event that all hypotheses within $\mathcal{F}_1$ are rejected. The procedure will initially assign two-sided local significance level α/3 (≈ 0.01667) to each member of $\mathcal{F}_1$. If a hypothesis within $\mathcal{F}_1$ is rejected, its local alpha will be reallocated equally among the remaining hypotheses within $\mathcal{F}_1$ (i.e., a Holm-type multiplicity procedure). In the event that all 3 hypotheses within $\mathcal{F}_1$ have been rejected, testing will proceed to $\mathcal{F}_2$, with alpha reallocated equally among its 3 hypotheses.

Figure 2. Multiple testing flow diagram

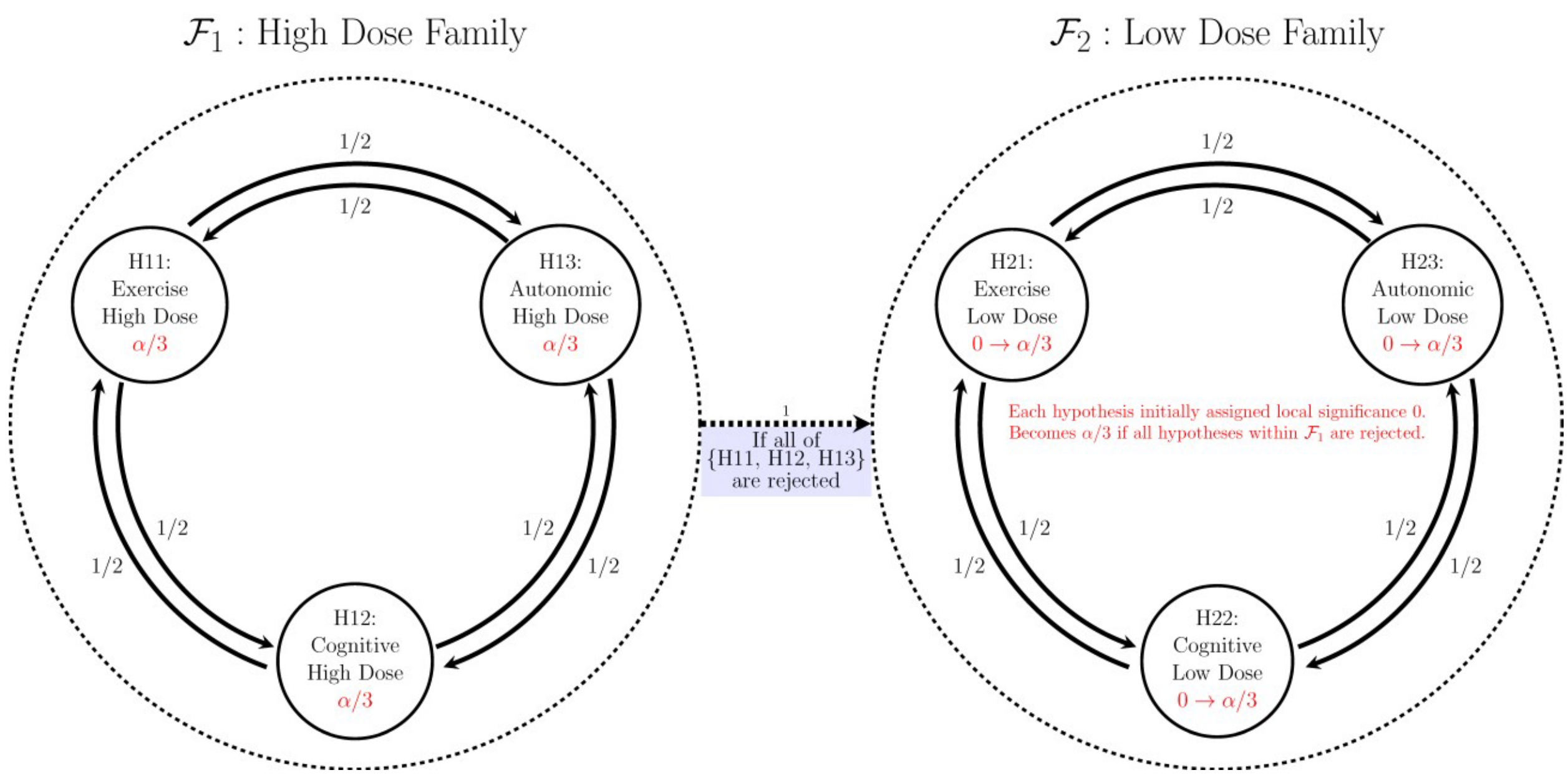

5.5.2 Secondary Endpoints

The multiplicity procedure described above provides strong type-I error control for the primary endpoints. For secondary endpoints, a less conservative approach will be adopted. For each symptom cluster and dose duration, if the hypothesis pertaining to the primary endpoint is rejected, then the symptom cluster's major secondary endpoint will be tested at the same dose duration using alpha = 0.05. This does not strictly control type-I error across all primary and secondary outcomes, but does offer some protection by using the primary outcome as a gatekeeper for secondary outcomes within each symptom cluster and dose duration. This less conservative approach is motivated by viewing the study design as a separate trial for each symptom cluster population and recognizing that a subsequent Phase 3 trial will be required for any definitive efficacy conclusions. Of note, a finding of benefit in a particular symptom cluster population will only pertain to that symptom cluster population and will not be extrapolated to other symptom cluster populations.

5.5.3 Exploratory Endpoints

No adjustment will be made for multiplicity in the analysis of exploratory endpoints.

5.6 Covariate Adjustment and Subgroups

5.6.1 Covariate adjustment and selection considerations

To account for chance covariate imbalances, all major efficacy analyses will be adjusted for pre-specified baseline covariates. Such adjustment is consistent with recommendations in two recent FDA guidance documents:

FDA's May 2021 guidance on adjusting for covariates in randomized clinical trials for drugs and biological products: "Although an unadjusted analysis is acceptable for the primary analysis, adjustment for baseline covariates will generally reduce the variability of estimation of treatment effects and thus lead to narrower confidence intervals and more powerful hypothesis testing." [7]

FDA's February 2021 guidance on developing drugs and biological products for treatment or prevention of COVID-19: "To improve the precision of treatment effect estimation and inference, sponsors should consider adjusting for prespecified prognostic baseline covariates (e.g., age, baseline severity, comorbidities, baseline medications and COVID-19 vaccination status) in the primary efficacy analysis and should propose methods of covariate adjustment. For example, for a binary endpoint, methods can be used to gain precision in the evaluation of the difference in proportions." [8]

Importantly, covariate adjustment can be implemented in a way that makes it robust to misspecification of the underlying regression models. [7] In other words, the calculated estimates and confidence intervals will not depend on correct model specification in order to be valid.

The main motivation for implementing covariate adjustment in this study protocol's efficacy assessments is to improve estimation efficiency, as discussed in FDA guidance documents. As such, prespecified covariates were selected due to their suspected strong association with study outcomes.

An additional factor motivating covariate adjustment was a concern raised by the DMSB about potential for chance imbalances in site across treatment group. Such chance imbalances are possible because there is no stratification by site in the randomization procedure. As such, additional pre-specified covariates were selected based on their suspected strong associations with site and their potential association with study outcomes.

A final additional factor motivating covariate adjustment was the desire to potentially reduce bias that may result from missing outcome data. If analyses are not covariate-adjusted and missing outcomes occur, then treatment effect estimates will generally only be unbiased if the outcomes satisfy the condition of being missing completely at random (MCAR). If instead the efficacy analysis is covariate adjusted, then unbiased estimation can potentially be achieved if outcomes satisfy the relatively weaker condition of being missing at random (MAR), after adjusting for baseline covariates. While both assumptions (MCAR and MAR) are untestable and neither is likely to be literally true, the MAR assumption is a relatively weaker condition and therefore more likely to generate accurate estimates under a wider range of missing data scenarios. Sensitivity analyses with a range of adjustment methods are discussed in Section 8.2 and Appendix 2.

5.6.2 Pre-selected Covariates

Different pre-specified covariates will be used in different analyses for different endpoints. They are documented in relevant parts of Section 8.1.

5.6.3 Subgroups

The following subgroups have been pre-specified for exploratory analyses of treatment effect heterogeneity.

Primary interest

- Antigenemia positive at baseline (yes, no)
- Vaccination status at time of first acute COVID-19 infection (≥1 dose completed, 0 doses completed)
- Time from first COVID-19 infection that led to current PASC symptoms to randomization (grouped by approximate tertiles) Other

- Race
- Ethnicity
- Sex
- Acute COVID severity

The above designation of "primary interest" versus "other" does not appear in the protocol. It was adopted based on discussion with study principal investigators and pre-specified before first patient enrollment. The list of pre-specified subgroups was intentionally limited in order to reduce potential for spurious findings and to prioritize variables with a plausible mechanism to interact with treatment.

6 SUMMARY OF STUDY DATA

Continuous variables will be presented as n (with number of non-missing observations), mean, standard deviation, median, Q1, Q3, and minimum and maximum. Binary and categorical variables will be presented as counts and percentages (among non-missing values).

In general, summary tables will be structured with a column for each treatment group (25-day dose duration, 15-day dose duration, control) and will be annotated with the total population size relevant to that table. Summary tables will be constructed for the overall trial cohort and for each symptom cluster.

6.1 Subject Disposition

The disposition of subjects (number randomized, number who received any amount of the randomly assigned treatment, number completing study drug administration, number who withdrew consent or discontinued from study drug early, and number lost to follow-up, and number who completed the trial) will be summarized by treatment group. The number of subjects screened for inclusion and a breakdown of reasons for exclusion will be summarized. The timing and reasons for early discontinuation of study drug and/or withdrawal from the study will be summarized by treatment group. For the calculation of percentages, subjects who die will not be included in the denominators for visits/assessments beyond their death. A subject listing of analysis population eligibilities will be generated. A listing of all subjects discontinued from the study after enrollment, broken down by site and treatment group will be provided. The listing will include: reason for discontinuation, treatment group, duration of treatment, and whether or not the blind was broken. Also, for subjects who discontinued from the study after enrollment, a listing of adverse events will be provided. Subjectspecific protocol deviations will be summarized by the reason for the deviation, the deviation category, treatment group, and site for all subjects. All subject-specific protocol deviations and non-subject specific protocol deviations will be included in listings.

6.2 Demographics and Medical History

Baseline demographics and medical history will be summarized by assigned treatment group within symptom cluster and over all symptom clusters. For continuous measures the mean, standard deviation, median, Q1, Q3, min, and max will be summarized. Categorical variables will be described by the proportion in each category (with the corresponding sample size). Baseline characteristics will include, but are not limited to: age, sex, race, ethnicity, years of education, height, weight, blood pressure, pulse,

baseline SARS-CoV-2 vaccination status, treatment of symptoms for first PASC COVID infection, and medical history.

6.3 Vital Signs

Vital sign measurements include blood pressure and pulse (supine and standing).

6.4 Baseline and Concomitant Medications

Concomitant medications will be coded to the Anatomical Therapeutic Classification (ATC) using the WHO Drug Dictionary. The use of prior, concomitant medications taken, and changes in concomitant medications during the study will be recorded on the CRFs. Baseline concomitant medications will include all concomitant therapies taken by the participant within 14 days prior to informed consent. Concomitant medications will be reviewed through the End of Study. The use of concomitant medications that were taken by 2% or more of the population at both of the baseline and Day 90 visits will be summarized by coded term and treatment group.

7 INTERIM ANALYSES AND DATA MONITORING

Interim examination of clinical endpoints and key safety events will be performed at regular intervals during the course of each intervention appendix. An independent, NIH-appointed, DSMB will monitor participant safety and review participant enrollment and performance of the trial. The primary objective of these interim analyses will be to ensure the safety of the participants enrolled in the trial and evaluate the accumulating endpoint data by treatment group. In addition, interim monitoring will involve a review of participant recruitment, compliance with the study protocol, status of data collection, and other factors which reflect the overall progress and integrity of the study. A complete description of the interim analyses plans with mock tables, listings, and figures is included in a separate DSMB guidance document.

There are no planned early stopping rules for efficacy in this protocol. Because PASC presentations and outcomes are highly varied, an important study objective is to estimate the effect of treatment on a wide range of participant-relevant outcomes. If the study were to be stopped early with less than the full sample size, it would decrease precision and reduce the study's ability to characterize treatment risks and benefits based on important secondary effectiveness and safety outcomes. It would also limit the collection of data that are critical for planning future trials in similar patient populations.

Early stopping for rules for futility may be incorporated on an intervention appendix specific basis. Such rules will be documented in the intervention appendices. There are currently no planned futility analyses for the Paxlovid appendix.

8 EFFICACY ANALYSES

8.1 Primary Efficacy Analysis

Each of the three symptom cluster-specific primary endpoints is a binary indicator of meeting a prespecified degree of improvement in a patient reported outcome at 90 days.

The Day 90 analysis window for all three primary endpoints will be between Day 76 and Day 104. For the PROMIS-cognitive 8a and OHQ, the average of the 3 weekly assessments closest to Day 90 will be used. If fewer than 3 assessments are completed within the Day 90 analysis window, the average of all available assessments will be used.

A separate comparison will be performed for each combination of 2 dose durations and 3 symptom clusters using the multiplicity adjustment procedure described in Section 5.5. First, efficacy of the 25day dose duration will be compared to control within each symptom cluster. If all 3 cluster-specific comparisons are significant, after accounting for multiplicity, then an additional set of cluster-specific efficacy comparisons will be performed for the 15-day dose duration versus and control. Otherwise, testing will stop and all 15-day analyses will be regarded as exploratory. Comparisons between the 25day and 15-day dose durations will be performed and regarded as exploratory.

For each between-group treatment comparison, the estimand of interest is the between-group difference in marginal probabilities of improvement. In probability notation, the estimands of interest are:

$$\Delta_{1,3} = P(Y = 1|\mathrm{TRT} = 1) - P(Y = 1|\mathrm{TRT} = 3), \qquad \text{(25 day versus control)}$$

$$\Delta_{2,3} = P(Y = 1|\mathrm{TRT} = 2) - P(Y = 1|\mathrm{TRT} = 3), \qquad \text{(15 day versus control)}$$

$$\Delta_{1,2} = P(Y = 1|\mathrm{TRT} = 1) - P(Y = 1|\mathrm{TRT} = 2), \qquad \text{(25 day versus 15 day)}$$

where 1 = PAXLOVID 25 days, 2 = PAXLOVID 15 days, 3 = Control.

The above quantities are defined without regard to adherence and implicitly average over observed patterns of concomitant therapies.

If primary endpoint assessment is prevented by death or hospitalization, the participant will be classified as not improving and considered to have non-missing outcome status.

For the comparison between treatment groups j and k, the null hypothesis is:

$$H_0: \Delta_{j,k} = 0.$$

The alternative hypothesis is:

$$H_0: \Delta_{j,k} \neq 0.$$

Treatment effects for all three dose groups will be estimated simultaneously, rather than through separate pairwise analyses. This ensures consistency of estimates across all 3 pairwise comparisons.

Estimation of $\Delta_{j,k}$ will follow the covariate adjustment approach of Benkeser et al. (2020) [9], which combines regression estimation with direct standardization (also known as G-computation). While Benkeser et al. describe covariate adjustment in the context of a two-arm trial, the approach extends naturally to settings with more than two treatment groups by estimating treatment effects for all groups simultaneously and comparing predicted outcomes across all pairwise contrasts.

To begin, a binary logistic regression model predicting the primary endpoint will be fitted to data from mITT cohort. The outcome variable will be an indicator of the participant's primary endpoint status

(1=improvement, 0=no improvement). Participants with missing primary endpoint status will be included but assigned weight zero during model fitting.

This model will be used to generate predicted probabilities of improvement under each treatment condition. The following covariates will be included:

- Treatment group (TRT)
- Primary PRO at baseline (continuous variable):
    - Cognitive cluster: Baseline PROMIS-Cognitive 8a T-score ○ Autonomic cluster: Baseline OHQ Question 1
    - Exercise cluster: Baseline number of symptoms meeting criteria for PEM
- Interaction of TRT x primary PRO at baseline
- Race
- Years of education
- US region (Northeast, Midwest, South, West, with Puerto Rico included in the South)

The inclusion of treatment x covariate interactions for some but not all covariates is intended to provide adequate flexibility while mitigating computational issues associated with sparse categorical covariates.

After estimating regression coefficients, the model's prediction equations will be applied to each participant in the pooled mITT population in order to calculate predicted probabilities of improvement for each participant under each treatment condition.

For the comparison of treatment group j versus k, the treatment effect will be estimated as:

$$\hat{\Delta}_{j,k} = \frac{1}{n}\sum_{i=1}^{n} \hat{P}_i^{(j)} - \frac{1}{n}\sum_{i=1}^{n} \hat{P}_{i(k)},$$

where

- $\hat{P}_i^{(j)}$ is the predicted probabilty of improvement for participant i under treatment j,
- $\hat{P}_i^{(j)}$ is the predicted probabilty of improvement for participant i under treatment k,
- n is the number of participants in the cluster-specific primary analysis population across all three treatment groups.

In absence of missing outcome data, this estimator is asymptotically consistent, regardless of whether the logistic regression model is correctly specified. If missing outcome data occur, the estimator remains consistent under the missing at random (MAR) assumption, provided the logistic regression model is correctly specified. Sensitivity/supplementary analyses using estimators that maintain validity under different missing data assumptions are described in Section 8.2 and Appendix 2.

Inference for $\Delta_{j,k}$ will be based on Wald-type test statistics with empirical (sandwich) standard errors:

$$Z = \frac{\hat{\Delta}_{j,k}}{\hat{se}\,(\hat{\Delta}_{j,k})}.$$

Approximate 95% confidence intervals will be constructed as

$$\hat{\Delta}_{j,k} \pm Z_{0.025} \times \hat{se}\,(\hat{\Delta}_{j,k})$$

where $Z_{\alpha/2}$ is the upper $1-\alpha/2$ quantile of the standard normal distribution (e.g., $Z_{0.025} \approx 1.96$). Empirical (sandwich) standard errors will be calculated as:

$$\hat{se}\,(\hat{\Delta}_{j,k}) = \sqrt{\frac{1}{n^2}\sum_{i=1}^{n} \left\{ \hat{P}^{(j)} + \frac{Z_{j,i} M_i \,[Y_i - \hat{P}_i^{(j)}]}{\bar{Z}_j} - \hat{P}^{(k)} - \frac{Z_{k,i} M_i \,[Y_i - \hat{P}_i^{(k)}]}{\bar{Z}_k} - \hat{\Delta}_{j,k} \right\}^2},$$

where:

n = number of participants in the mITT population,

$$Z_{t,i} = \begin{cases} 1 & \text{if } i\text{-th participant was randomized to group } t \\ 0 & \text{otherwise} \end{cases}, \quad t = j, k,$$

$$\bar{Z}_t = \frac{1}{n}\sum_{i=1}^{n} Z_{t,i}, \quad t = j, k,$$

Y_i = primary outcome of i-th participant,

$$M_i = \begin{cases} 0 & \text{if } Y_i \text{ is non-missing} \\ 1 & \text{if } Y_i \text{ is missing} \end{cases},$$

with the convention that a missing value multiplied by zero is zero.

8.2 Handling of Missing Data

8.2.1 Overview

A particularly strong effort will be made to prevent participant's loss to follow-up and missing outcome data. The frequency of missing data for key variables will be summarized by treatment group. The baseline characteristics and outcomes of participants with and without missing data will be compared. If assessment of a primary or secondary endpoint is prevented by death or hospitalization, the participant will be classified as not improving and will be considered to have non-missing outcome status. A listing of participants with missing primary endpoint data and who died or were hospitalized will be provided to the CRAs for adjudication. The CRAs will review the listing and determine if the primary endpoint collection was prevented by death or hospitalization. If the resulting adjudication is that primary endpoint collection was prevented, the primary endpoint will be set to no improvement. If the resulting adjudication is that primary endpoint collection was not prevented, the primary endpoint will stay missing.

8.2.2 Considerations for primary endpoint analysis

The primary analysis was designed to minimize bias and maximize use of available data from participants with missing primary endpoint values. It involves:

(1) fitting an outcome model; and
(2) averaging predicted values across participants.

The following missing data rules will be implemented:

(1) Outcome model fitting will only use participants with non-missing primary endpoint data.
(2) Predicted values will be generated and averaged across all participants in the mITT population, including those with missing outcomes, with the following exception:
    - Participants who have missing data for **both** the primary endpoint **and** the baseline PRO used to define it will be excluded.

This exclusion is necessary because the primary endpoint is defined relative to the baseline PRO. In the absence of a baseline value, the primary endpoint is undefined. Moreover, when both the baseline PRO and the primary endpoint are missing, the model cannot reliably estimate predicted values, as no similar participants with observed outcomes exist. Therefore, participants with jointly missing baseline PRO and primary endpoint will be excluded from the standardization step of the primary endpoint analysis.

These participants will not be excluded from the mITT population and this restriction applies only to the primary endpoint analysis. Secondary analyses of outcomes not defined relative to baseline will use the full mITT population.

Missing baseline covariates will be handled by median imputation, with possible inclusion of corresponding missing data indicators in the outcome model (Zhao and Ding 2022) [10].

8.2.3 Sensitivity Analysis: propensity-weighted logistic regression.

To assess sensitivity to the method of handling missing outcome data, the analysis described in Section 8.1 will be modified to incorporate inverse probability weighting (IPW) into the estimation of the outcome model. This approach is doubly robust, in the sense that valid inference can be obtained if either the outcome model or the model for missing data is correctly specified.

To implement this approach, we first fit three separate logistic regression models, one for each treatment group. For the j-th model, the outcome is an indicator for the joint occurrence of two events:

(1) randomization to treatment group j; and (2)
availability of a non-missing primary endpoint.

Each model estimates the probability that a participant's counterfactual outcome under treatment j, denoted $Y^{(j)}$, would be observed, conditional on covariates. Participants with non-missing outcome data are then assigned weights inversely proportional to their estimated probability of having an observed outcome under their assigned treatment group. These weights are applied when fitting the outcome model, which is otherwise identical to the primary analysis.

As in the primary analysis, predicted probabilities of improvement under each treatment will be generated for all participants (excluding those with both missing outcome and missing baseline PRO, as described in Section 8.2.2). These predicted values will then be averaged across the cluster-specific mITT population to estimate marginal treatment effects.

8.3 Analysis of Secondary Endpoints

Each symptom cluster-specific secondary endpoint is a binary indicator of meeting a pre-specified degree of improvement on a performance outcome at 90 days. The Day 90 analysis window for all three secondary endpoints will be between Day 76 and Day 104.

A separate analysis will be performed for each of three primary symptom cluster populations and two active dose durations.

Multiplicity considerations for secondary endpoints are discussed in Section 5.5.2. Briefly, to reduce type-I errors, analysis for a particular combination of symptom cluster and dose duration (versus control) will be conditional on having met significance for the primary endpoint for the same combination of dose duration and symptom cluster. For each symptom cluster and dose duration, if the hypothesis pertaining to the primary endpoint is rejected, then the symptom cluster's secondary endpoint will be tested at the same dose duration using alpha = 0.05. If the hypothesis pertaining to the primary endpoint is not rejected, then analysis of the secondary endpoint will be regarded as exploratory.

A covariate-adjusted analysis will be performed using pre-specified covariates as listed below. Estimation will target the between-group different in marginal probabilities of improvement for active dose versus control. Except for using different covariates, analysis methods will identical to the primary efficacy analysis.

The following covariates will be used:

- Cognitive dysfunction symptom cluster:
  - Baseline AVLT Z-score ○ Baseline SDMT Z-score
  - Demographic variables: race, years of education, US region
- Autonomic dysfunction symptom cluster:
  - Baseline ΔSBP ○ Baseline ΔDBP ○ Baseline ΔHR
  - Demographic variables: race, years of education, US region
- Exercise intolerance symptom cluster:
  - Baseline ESWT walk time
  - Demographic variables: race, years of education, US region

8.4 Analysis of Exploratory PRO and Performance Endpoints at Days 90 and 180

The PRO and performance outcomes listed in Table 3 will be analyzed at Days 90 and 180 for each pairwise comparison between active dose versus control.

Table 3. Exploratory efficacy endpoints to be analyzed at days 90 and 180

**Exploratory Endpoint**
PROMIS Cognitive 8a T-score in cognitive cluster
OHQ Question #1 in autonomic cluster
DSQ-PEM number of criteria for PEM in exercise cluster
Neurocognitive battery outcomes at Day 90 in cognitive cluster
Change in BP and HR on active stand test at Day 90 in autonomic cluster
ESWT walk time at Day 90 in exercise cluster

PROMIS-29+2 domain scores
Patient global impression of change (PGIC)
ECOG-2 overall score in cognitive cluster
COMPASS-31 overall score and subscales in autonomic cluster
OHQ overall score and subscales in autonomic cluster

All endpoints listed above will be treated as continuous variables and will be analyzed via linear regression. All endpoints, except for the PGIC, will include adjustment for the corresponding baseline measurement. PGIC will not be adjusted because PGIC directly assesses improvement in relation to baseline and thereby allows each participant to serve as his or her own control. Additional analysis details are provided below.

We will present each version of the PASC Symptom Questionnaire at days 90 and 180. Frequency and severity of individual symptoms will be treated as continuous and only descriptive statistics will be presented.

8.4.1 Analysis of Continuous Outcomes

For continuous outcomes, the estimand will be the between treatment-group difference in means. For the comparison of group j versus k, the estimand is:

$$\Delta_{j,k} = E(Y|\mathrm{TRT} = j) - E(Y|\mathrm{TRT} = k)$$

where Y denotes the continuous outcome and TRT denotes randomization assignment. Methods of estimating $\Delta_{j,k}$ and calculating standard errors will be identical to the primary and secondary outcomes but will use a least squares linear regression instead of logistic model for the outcome. Pre-specified covariates will differ for each endpoint as listed in Table 3 above.

8.5 Analysis of Weekly Longitudinal PROs

The PRO outcomes listed in Table 4 are collected on a weekly basis and will be analyzed longitudinally for each pairwise comparison between active treatment and control.

Table 4. Endpoints to be analyzed longitudinally

**Exploratory Endpoint**	**Type**	**Covariates**
PROMIS Cognitive 8a T-score in cognitive cluster	Continuous	Baseline PROMIS 8a T-score
OHQ Question #1 in autonomic cluster	Continuous	Baseline OHQ Question #1
Number of symptoms meeting DSQ-PEM criteria in exercise cluster	Continuous	Baseline number of symptoms meeting DSQ-PEM criteria

Week-specific data will be summarized as means and differences by treatment group over weeks 1 to 26.

Differences by treatment group will be assessed by fitting generalized linear mixed models with participant-specific random effects (intercept and slope) using data from weeks 5 to 26. Weeks 1 to 4 will be excluded to reduce potential for attenuation of the treatment effect in the event that treatment effects experience a time lag. Models will incorporate a linear link. Fixed effects covariates will include treatment group, study week modeled as a restricted cubic spline, baseline values as listed in Table 4. Treatment effects will be expressed as differences in means. The above models omit time × by treatment interactions. The presence of time × treatment interactions will be assessed by adding interaction terms to the above models and testing whether the coefficients associated with interaction terms are equal to zero. If the hypothesis of no time × treatment interaction is rejected ($p < 0.05$), then results for models with interactions will also be presented and treatment effects will be estimated at Day 90.

8.6 Analysis of Heterogeneity of Treatment Effect (HTE)

8.6.1 Use of Pooled Active Treatment Groups

The list of pre-specified covariates for HTE analyses was intentionally kept small in order to reduce the number of comparisons and reduce potential for spurious findings. To further reduce comparisons, and to increase sample size, HTE analyses will be performed using a pooled active treatment group that pools 15- and 25-day dose durations.

8.6.2 Endpoints for HTE Analyses

Exploratory analyses of heterogeneity of treatment effect (HTE) will be performed for the endpoints listed in Table 5.

Table 5. Endpoints for analysis of treatment effect heterogeneity

Exploratory Endpoint	Type	Covariates
PROMIS Cognitive 8a T-score in cognitive cluster	Continuous	Baseline PROMIS 8a T-score
OHQ Question #1 in autonomic cluster	Continuous	Baseline OHQ Question #1
Number of symptoms meeting DSQ-PEM criteria in exercise cluster	Continuous	Baseline Number of symptoms meeting DSQ-PEM criteria

8.6.3 Subgroups

Subgroups for HTE analyses are listed in Section 5.6.3. Subgroups of primary interest for HTE include:

- Antigenemia positive at baseline (yes, no)
- Vaccination status at time of first acute COVID-19 infection (≥1 dose completed, 0 doses completed)
- Time from first COVID-19 infection that led to current PASC symptoms to randomization (grouped by approximate tertiles)

8.6.4 Estimation methods

Analyses of HTE will be performed one-at-a-time for each outcome and subgroup variable.

HTE analysis will focus on estimating mean outcome differences between treatment group across levels of the subgroup variable. This will be accomplished by fitting an ANCOVA linear regression model with adjustment for treatment group (pooled active versus control), baseline measurement of the outcome variable, subgroup variable of interest, and the subgroup × treatment interaction. Estimated between treatment-group mean differences will be reported for each subgroup with associated 95% confidence intervals and with a p-value for the test of no subgroup × treatment interaction.

8.6.5 Estimation of Covariate-specific Treatment Effects

As a further exploratory analysis, a Bayesian multivariable modeling strategy may be used to estimate the treatment effects for a subset of the above outcomes as a function of the 3 pre-specified primary interest subgroup variables jointly. This analysis will improve on conventional subgroup analyses by analyzing multiple subgroup factors jointly in a single multivariable model. Results of this analysis may inform the choice of target population for a possible future Phase 3 evaluation. Results will be shared with study investigators and NIH leadership to inform future study design but may be omitted from the final CSR. A separate analysis plan will be developed for this analysis.

8.7 Bayesian Analyses

To enhance interpretation, the endpoints listed in Table 6 below will be re-analyzed in a Bayesian statistical framework. Analysis will be performed using a range of skeptical, neutral, and enthusiastic priors as suggested by Wijeysundera et al. (2009) [12] and Zampieri et al. (2021) [13]. Results reporting will be facilitated by an R shiny app that will display results for a range of user-requested prior distributions.

8.7.1 Endpoints and statistical framework

Table 6. Endpoints for exploratory Bayesian analyses

**Endpoint**	**Analysis methods**
PROMIS Cognitive 8a Tscore in cognitive cluster at day 90 (continuous)	• Analysis framework: ○ ANCOVA linear regression<br>• Covariates: ○ Baseline PROMIS Cognitive 8a T-score, re-scaled to have SD = 1.<br>○ Treatment group indicator<br>• Treatment effect estimand: ○ Between treatment-group mean difference as captured by regression coefficient for treatment
Presence of PEM according to DSQ-PEM in exercise cluster at day 90 (binary)	• Analysis framework: ○ Binary logistic regression<br>• Covariates: ○ Baseline number of symptoms meeting criteria for PEM, re-scaled to have SD = 1.

	○ Treatment group indicator<br>• Treatment effect estimand: ○ Marginal difference in probability of PEM, calculated by averaging predicted probabilities under active and control treatments across all mITT participants
OHQ Question #1 in autonomic cluster at day 90 (continuous)	Analysis framework:<br>• ANCOVA linear regression Covariates:<br>• Baseline OHQ Question #1, re-scaled to have SD = 1.<br>• treatment group indicator Treatment effect estimands:<br>Between treatment-group mean difference as captured by regression coefficient for treatment
Patient global impression of change (PGIC) in overall mITT cohort at day 90 (ordinal)*	*This analysis will be shared with study investigators, but will not be included in the final CSR.<br>Analysis framework:<br>• Nonparametric; PGIC will be assumed to follow a multinomial distribution with cell probabilities differing by treatment group. See Appendix 2 for details.<br>Covariates:<br>• None, unadjusted<br>Treatment effect estimand:<br>• Win Ratio (See Appendix 2 for definition).

8.7.2 Prior Specification for Cognitive 8a

The prior will consist of independent normal distributions for regression coefficients and an inverse Gamma distribution for the residual variance. In light of limited prior information, the prior for the baseline PROMIS Cognitive 8a coefficient will be normal with mean 0 and SD = 10. For analyses using a neutral prior, the prior for the treatment coefficient will be normal with mean 0 and SD = 10. Priors for the treatment coefficient will be as follows:

- Neutral:
  - $\beta \sim$ Normal(mean = 0, SD = 10)
  - This prior is chosen to reflect a state of uncertainty concerning the direction and magnitude of the treatment effect.
- Skeptical:
  - $\beta \sim$Normal(mean = 0, SD = 3) ○ This prior implies that:
    - The prior most likely value for the mean difference is 0 T-score points
    - The prior probability of a benefit exceeding 5 T-score points is approximately 5%

- Enthusiastic:
    - $\beta \sim$ Normal(mean = 5, SD = 7.5) ○ This prior implies that:
        - The prior most likely value for the mean difference is 5 T-score points
        - The prior probability of any benefit ($\beta > 0$) is approximately 75%.

8.7.3 Prior Specification for OHQ Question #1

The prior will consist of independent normal distributions for regression coefficients and an inverse Gamma distribution for the residual variance. In light of limited prior information, the prior for the baseline OHQ Question #1 coefficient will be normal with mean 0 and SD = 10. For analyses using a neutral prior, the prior for the treatment coefficient will be normal with mean 0 and SD = 10. Priors for the treatment coefficient will be as follows:

- Neutral:
    - $\beta \sim$ Normal(mean = 0, SD = 10)
    - This prior is chosen to reflect a state of uncertainty concerning the direction and magnitude of the treatment effect.
- Skeptical:
    - $\beta \sim$Normal(mean = 0, SD = 0.6) ○ This prior implies that:
        - The prior most likely value for the mean difference in Question #1 is 0 units The prior probability of a benefit exceeding 1 unit is approximately 5%
- Enthusiastic:
- $\beta \sim$ Normal(mean = 1, SD = 1.5) ○ This prior implies that:
        - The prior most likely value for the mean difference is 5 T-score points The prior probability of any benefit ($\beta > 0$) is approximately 75%.

8.7.4 Prior Specification for PEM

The prior will consist of independent normal distributions for all regression coefficients. In light of limited prior information, the prior for the baseline number of qualifying PEM symptoms coefficient will be normal with mean = 0 and SD = 10. Priors for the treatment coefficient will be as follows:

- Neutral:
    - $\beta \sim$ Normal(mean = 0, SD = 10)
    - This prior is chosen to reflect a state of uncertainty concerning the direction and magnitude of the treatment effect.
- Skeptical:
    - $\beta \sim$Normal(mean = 0, SD = 0.25) ○ This prior implies that:
        - The prior most likely value of the treatment effect odds ratio is 1
        - The prior probability of a treatment effect odds ratio exceeding 1.5 is approximately 5%
- Enthusiastic:
    - $\beta \sim$ Normal(mean = 0.405, SD = 0.6) ○ This prior implies that:
        - The prior most likely value for the treatment effect odds ratio is 1.5
        - The prior probability of any benefit (odds ratio > 1) is approximately 75%.

8.7.5 Bayesian R Shiny App

The analyses described above are based on a small number of pre-specified prior distributions. To facilitate results reporting under a wider range of priors, an R Shiny app will be developed. It will be designed to provide interactive Bayesian analysis under an arbitrary range of user-specified subjective prior distributions. For a subset of the above endpoints, the app will calculate the posterior probability that the unknown treatment effect falls above or below a user-specified margin representing clinically important harm or benefit. For model-based analyses, the app will assign a user-specified prior to the treatment effect parameter of interest and a default reference prior to other parameters. An importance weighting strategy will be used to update posterior probabilities in real time without needing to repeat computationally intensive posterior sampling when the user's choice of prior is varied.

9 ANALYSIS OF MEDICAL VISITS AND HOSPITALIZATIONS

Descriptive analyses will summarize the mean number of healthcare encounters by treatment group for all types of encounters in aggregate and for specific types of encounters (inpatient hospitalization, outpatient ER visit, primary care provider visit, urgent care visit, planned hospitalization unrelated to COVID-19). We will present the cumulative mean number of healthcare encounters per participant overall and by type for each active dose group versus control at 12 weeks post-randomization and at 25 weeks post-randomization. These timepoints were chosen to roughly correspond to the beginning of the visit windows for the 90-day and end-of-study visits.

10 SAFETY ANALYSES

Safety endpoints include the proportion of participants who experience individual AEs/SAEs and the proportion who experience any one or more AEs/SAEs. Each of these will be compared pairwise between each of the two intervention dose levels and control. Risk differences between each intervention dose level and control group and 95% CIs using the Miettinen-Nurminen method will be estimated. Incidence of AEs/SAEs leading to discontinuation of study intervention will also be summarized.

AEs will be coded in accordance with the current version of the Medical Dictionary for Regulatory Activities (MedDRA). AEs will be graded with regard to severity according to criteria defined in the Common Terminology Criteria for Adverse Events version 5.0 (CTCAE) and relationship to study intervention (related/not related). Each AE will be counted once only for a given participant. AEs will be presented by system organ class, preferred term (PT), duration (in days), start- and stop-date.

For this study intervention appendix, there are two ESIs specified in the protocol: 1. Alteration in thyroid function and 2. Drug-Induced Liver Injury (DILI). ESIs will be summarized.

10.1 Clinical Laboratory Data

Blood for clinical laboratory assessments will be collected at baseline, drug dosing midpoint, end of dosing, and day 90 for all participants. Parameters evaluated include a comprehensive metabolic panel, high-sensitivity c-reactive protein, D-dimer, coagulation panel (prothrombin time, activated partial thromboplastin time, thrombin time), thyroid stimulating hormone (TSH), total and free triiodothyronine (T3), and total and free thyroxine (T4), and complete blood count with differential.

For the purposes of reporting safety laboratory assessments, the following visit windows will be used:

Table 7. Analysis Visit Windows for Laboratory Data

Visit	Target Study Day	Analysis Window Study Day	
		Low	High
Baseline	0	≤ 0	0
Drug Dosing Midpoint Clinic Visit	15	1	20
End of Dosing Clinic Visit	25	21	35
Optional Day 45	45	36	52
Optional Day 60	60	53	75
Day 90	90	76	105
Optional Day 120	120	106	End of Study

Descriptive statistics for baseline and post-baseline time points will be generated. Additionally, shift tables will be presented. The shift tables tabulate the number of lab values determined to be "Normal" and "Abnormal" at baseline and post-baseline time points. The classifications on shift tables will categorize lab values as "Normal" when they are in acceptable range. Laboratory values outside the normal range will be graded on the Labs Listing as >1x, >2x, >3x, >5x, or >10x the upper limit (or <0.1x, <0.5x, <1x the lower limit) of normal value based on appropriate increases or decreases. Laboratory data will also be summarized graphically to show the magnitude and changes in individual subject values over time relative to normal ranges. Details of any abnormalities will be provided in a listing.

11 BIOMARKER ANALYSES

Plans for biomarker analyses will be developed in a separate biomarker SAP. Briefly, markers of interest for mechanistic evaluation include antigenemia (defined as detection of SARS-CoV-2 spike protein in plasma), inflammation markers (high-sensitivity c-reactive protein, D-dimer), and coagulation markers (prothrombin time, activated partial thromboplastin time, thrombin time). Distributions of these biomarkers will be descriptively summarized and compared across treatment groups at baseline, middosing visit, end of dosing visit, and days 45, 60, 90, and 120. Viral clearance will be assessed by comparing the frequency of antigenemia at post-randomization visits across treatment groups among participants who are positive for antigenemia at baseline.

12 ADHERENCE

The distribution of the number of completed and missed doses will be summarized by treatment group and within symptom cluster. Reasons why the full course of treatment was not completed will be summarized by treatment group and within symptom cluster.

13 REFERENCES

1. Yost KJ, Eton DT, Garcia SF, Cella D. Minimally important differences were estimated for six Patient-Reported Outcomes Measurement Information System-Cancer scales in advanced-stage cancer patients. J Clin Epidemiol. May 2011;64(5):507-16. doi:10.1016/j.jclinepi.2010.11.018

2. Kroenke K, Stump TE, Chen CX, et al. Minimally important differences and severity thresholds are estimated for the PROMIS depression scales from three randomized clinical trials. J Affect Disord. Apr 1 2020;266:100-108. doi:10.1016/j.jad.2020.01.101

3. Terwee CB, Peipert JD, Chapman R, et al. Minimal important change (MIC): a conceptual clarification and systematic review of MIC estimates of PROMIS measures. Qual Life Res. Oct 2021;30(10):2729-2754. doi:10.1007/s11136-021-02925-y

4. University; N. Meaningful Change for PROMIS(R). Accessed January 20, 2023. https://www.healthmeasures.net/score-and-interpret/interpret-scores/promis/meaningfulchange

5. Norman GR, Sloan JA, Wyrwich KW. Interpretation of changes in health-related quality of life: the remarkable universality of half a standard deviation. Med Care. May 2003;41(5):582-92. doi:10.1097/01.Mlr.0000062554.74615.4c

6. HealthMeasures. PROMIS adult profile instruments scoring manual. September 2021. https://www.healthmeasures.net/images/PROMIS/manuals/Scoring_Manuals_/PROMIS_Adult_Profile_Scoring_Manual.pdf

7. U.S. Food and Drug Administration. "Adjusting for covariates in randomized clinical trials for drugs and biological products: guidance for industry." May 2021. https://www.fda.gov/media/148910/download

8. U.S. Food and Drug Administration. " COVID-19: Developing drugs and biological products for treatment or prevention: guidance for industry." February 2021. https://www.fda.gov/media/167274/download

9. Benkeser D, Díaz I, Luedtke A, Segal J, Scharfstein D, Rosenblum M. Improving precision and power in randomized trials for COVID-19 treatments using covariate adjustment, for binary, ordinal, and time-to-event outcomes. Biometrics. Dec 2021;77(4):1467-1481. doi:10.1111/biom.13377

10. Zhao A, Ding P. To adjust or not to adjust? estimating the average treatment effect in randomized experiments with missing covariates. Journal of the American Statistical Association. 2022 Oct 11:1-1.

11. Jackson D, White IR, Mason D, Sutton S. A general method for handling missing binary outcome data in randomized controlled trials. Addiction. 2014 Dec;109(12):1986-93.

12. Wijeysundera DN, Austin PC, Hux JE, Beattie WS, Laupacis A. Bayesian statistical inference enhances the interpretation of contemporary randomized controlled trials. Journal of clinical epidemiology. 2009 Jan 1;62(1):13-21.

13. Zampieri FG, Casey JD, Shankar-Hari M, Harrell Jr FE, Harhay MO. Using Bayesian methods to augment the interpretation of critical care trials. An overview of theory and example reanalysis of the alveolar recruitment for acute respiratory distress syndrome trial. American journal of respiratory and critical care medicine. 2021 Mar 1;203(5):543-52.

14. Kessler DC, Hoff PD, Dunson DB. Marginally specified priors for non-parametric Bayesian estimation. Journal of the Royal Statistical Society Series B: Statistical Methodology. 2015 Jan;77(1):35-58.

14 APPENDICES

Appendix 1. RECOVER-VITAL Roster of Statisticians

Team	Name	Email	Attends DSMB Meetings

Unblinded	Frank Rockhold	frank.rockhold@duke.edu	X
	Zhen Huang	zhen.huang@duke.edu	X
	Sophia Waymyers	sophia.waymyers@duke.edu	X
Blinded	Sean O'Brien	sean.m.obrien@duke.edu	X
	Adam Silverstein	adam.silverstein@duke.edu	
	Pearl Zakroysky	pearl.zakroysky@duke.edu	X
	Sam Broderick	samuel.broderick@duke.edu	

Appendix 2. Prior Specification for Bayesian Analysis of the PGIC

Endpoint Description

The Patient Global Impression of Change (PGIC) is based on the question:

"Compared to the start of the study, the overall severity of your Long COVID symptoms have become _______."

Response options are:

1) Improved a lot
2) Improved somewhat
3) Stayed about the same
4) Worsened somewhat
5) Worsened a lot

Because there are 5 possible values, the distribution of PGIC within each treatment group can be described by a set of 5 probabilities that sum to 1. The following description applies to each pairwise comparison between a treatment and control group.

Target Estimand: Win Ratio

The primary estimand is the nonparametric win ratio, also known as the generalized odds ratio (Agresti, 1980). The win ratio is a model-free, interpretable summary of treatment effect, defined as the probability that a randomly selected participant from the treatment group reports a better PGIC outcome than a randomly selected participant from the control group, divided by the probability of the reverse:

$$\text{win ratio} = \frac{P(Y^1 < Y^0)}{P(Y_0 < Y_1)},$$

where Y^1 and Y^0 are PGIC outcomes of participants randomly selected from the the treatment and control groups, respectively. A win ratio estimate greater than 1 will suggest that active treatment is superior to control and an estimate less than 1 will suggest the opposite.

Notation

The following notation will be used:

- $\boldsymbol{\alpha}_1 = (\alpha_{11},\alpha_{12},\alpha_{13},\alpha_{14},\alpha_{15})$ = unknown category probabilities in the treatment group
- $\boldsymbol{\alpha}_0 = (\alpha_{01},\alpha_{02},\alpha_{03},\alpha_{04},\alpha_{05})$ = unknown category probabilities in the control group
- $\boldsymbol{Y}_1 = (Y_{11},Y_{12},Y_{13}, Y_{14},Y_{15})$ = observed counts in the treatment group
- $\boldsymbol{Y}_0 = (Y_{01},Y_{02},Y_{03}, Y_{04},Y_{05})$ = observed counts in the control group
- $n_1 = Y_{11} + Y_{12} + Y_{13} + Y_{14} + Y_{15}$ = number of participants in treatment group
- $n_0 = Y_{01} + Y_{02} + Y_{03} + Y_{04} + Y_{05}$ = number of participants in control group.

Here, α_{ij} is the probability that a participant from group i reports a PGIC outcome in category j and Y_{ij} is the number of participants from group i that report a PCIG outcome in category j, for $i = 0,1$.

Using the above notation, the win ratio is defined as a function of $\boldsymbol{\alpha}_1$ and $\boldsymbol{\alpha}_0$, namely:

$$\text{WR}(\boldsymbol{\alpha}_1,\boldsymbol{\alpha}_0) = \frac{\sum_{i=1}^{4}\sum_{j=i+1}^{5} \alpha_{1i}\alpha_{0j}}{\sum_{i=1}^{4}\sum_{j=i+1}^{5} \alpha_{0i}\alpha_{1j}}.$$

Likelihood model

The distribution of observed counts will be modeled as independent multinomial distributions:

$$Y_1 \sim \text{Multinomial}(n_1, \boldsymbol{\alpha}_1) \text{ and } Y_0 \sim \text{Multinomial}(n_0, \boldsymbol{\alpha}_0).$$

The likelihood function is:

$$l(\boldsymbol{\alpha}_1, \boldsymbol{\alpha}_0; \boldsymbol{Y}_1, \boldsymbol{Y}_0) = \text{dmultinom}(\boldsymbol{Y}_1; \boldsymbol{\alpha}_1) \times \text{dmultinom}(\boldsymbol{Y}_0; \boldsymbol{\alpha}_0).$$

Prior Distribution

The marginal prior for the win ratio will be a log-normal distribution:

$$\log(\text{WR}) \sim \text{Normal}(\text{mean} = \mu, \text{SD} = \sigma).$$

Three prior settings for $\log(\text{WR})$ will be used:

- Neutral prior: $\mu = 0, \sigma = 10$
- Skeptical prior: $\mu = 0, \sigma = 0.25$
- Enthusiastic prior: $\mu = \log(1.5), \sigma = 0.6$

To induce the desired normal prior on $\log(\text{WR})$, the joint prior on $(\boldsymbol{\alpha}_1, \boldsymbol{\alpha}_0)$ will be constructed as a reweighted version of a reference prior. The reference prior will be the product of two independent Dirichlet distributions:

$$p_{\text{ref}}(\boldsymbol{\alpha}_1, \boldsymbol{\alpha}_0) = \text{Dir}(\boldsymbol{\alpha}_1 | \boldsymbol{a}_1) \times \text{Dir}(\boldsymbol{\alpha}_0 | \boldsymbol{a}_0),$$

where $\boldsymbol{a}_1$ and $\boldsymbol{a}_0$ are each set to (0.1, 0.1, 0.1, 0.1, 0.1), reflecting absence of strong prior information. The re-weighted prior will take the form:

$$p(\boldsymbol{\alpha}_1, \boldsymbol{\alpha}_0) \propto p_{\text{ref}}(\boldsymbol{\alpha}_1, \boldsymbol{\alpha}_0) \times w(\boldsymbol{\alpha}_1, \boldsymbol{\alpha}_0)$$

where the weight function $w(\cdot)$ is chosen to induce the desired normal prior on log-WR:

$$w(\boldsymbol{\alpha}_1, \boldsymbol{\alpha}_0) = \frac{\phi(\log(\text{WR}(\boldsymbol{\alpha}^1, \boldsymbol{\alpha}^0)) \, ; \mu, \sigma^2)}{g(\log(\text{WR}(\boldsymbol{\alpha}_1, \boldsymbol{\alpha}_0)))}.$$

Here, $\phi(; \mu, \sigma^2)$ denotes the density of a normal distribution with mean μ and variance σ^2; and $g(.)$ denotes the marginal density of $\log(\text{WR}(\boldsymbol{\alpha}_1, \boldsymbol{\alpha}_0))$ under the reference prior. This weighting approach adjusts for the discrepancy between the marginal distribution of $\log(\text{WR})$ under the reference prior and the desired prior distribution.

Because a closed form expression for $g()$ is not readily available, it will be estimated empirically using a kernel density estimator applied to 5,000,000 Monte Carlo samples drawn from p_{ref}. The resulting numerical approximation of $g(\cdot)$ is illustrated in Figure A1 below.

Figure A1. Marginal prior density of log win ratio under reference prior as estimated via Monte Carlo simulations with 5 million independent samples.

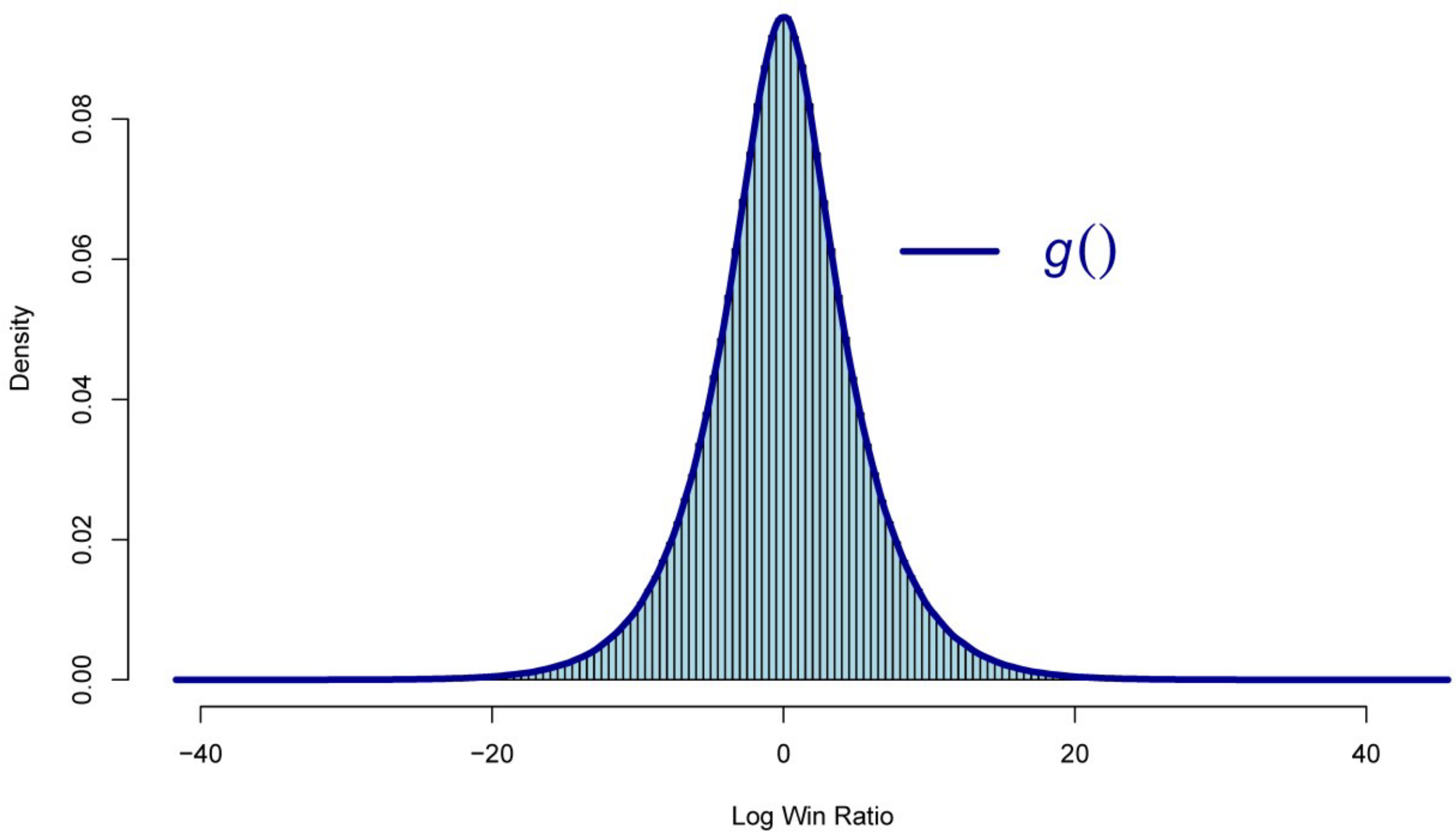

Posterior Computation

Posterior inference for WR will be based on Monte Carlo importance weighting. This approach enables estimation of posterior quantities without requiring direct sampling from the reweighted posterior distribution. Instead, samples will be drawn from the more computationally convenient reference posterior, which combines the reference prior with the multinomial likelihood. Because of conjugacy, the reference posterior has a simple closed form:

$$\boldsymbol{\alpha}_1 | \boldsymbol{Y}_1 \sim \text{Dirichlet}(\boldsymbol{a}_1 + \boldsymbol{Y}_1), \quad \boldsymbol{\alpha}_0 | \boldsymbol{Y}_0 \sim \text{Dirichlet}(\boldsymbol{a}_0 + \boldsymbol{Y}_0).$$

At least 1,000,000 samples will be drawn using the rdirichlet function from the **MCMCpack** R package.

Each sampled pair $(\boldsymbol{\alpha}_1^{(i)}, \boldsymbol{\alpha}_0^{(i)})$ will be assigned an importance weight $w_i = w\,(\boldsymbol{\alpha}_1^{(i)}, \boldsymbol{\alpha}_0^{(i)})$ where $w(\cdot)$ is the function defined above. The posterior mean of WR will then be estimated as:

$$\sum_{i=1}^{M} \tilde{w}_i \, \text{WR}\left(\boldsymbol{\alpha}_1^{(i)}, \boldsymbol{\alpha}_0^{(i)}\right), \quad \text{where} \quad \tilde{w}_i = w_i / \sum_{i=1}^{M} w_i,$$

and M is the number of posterior samples. Equal-tailed 95% credible intervals will be obtained from the weighted empirical distribution of sampled WR values.

RECOVER_VITAL_SAP_3APR2025_V2_Clean

Final Audit Report 2025-04-03

Created:	2025-04-03 (Eastern Daylight Time)
By:	Pearl Zakroysky (pz33@duke.edu)
Status:	Signed
Transaction ID:	CBJCHBCAABAALHQsG1yJ379iGx8WHyHUoiikBJw9n-wj

"RECOVER_VITAL_SAP_3APR2025_V2_Clean" History

Document created by Pearl Zakroysky (pz33@duke.edu)
2025-04-03 - 10:06:05 AM EDT- IP address: 136.226.107.7

Document emailed to Kanecia Zimmerman (kanecia.zimmerman@duke.edu) for signature
2025-04-03 - 10:07:54 AM EDT

Email viewed by Kanecia Zimmerman (kanecia.zimmerman@duke.edu)
2025-04-03 - 10:19:13 AM EDT- IP address: 146.75.232.0

Document e-signed by Kanecia Zimmerman (kanecia.zimmerman@duke.edu)
Signing reason: Approved
Signature Date: 2025-04-03 - 12:02:33 PM EDT - Time Source: server- IP address: 174.245.118.119

Document emailed to sean.m.obrien@duke.edu for signature
2025-04-03 - 12:02:35 PM EDT

Email viewed by sean.m.obrien@duke.edu
2025-04-03 - 3:45:22 PM EDT- IP address: 136.226.2.252

Signer sean.m.obrien@duke.edu entered name at signing as Sean O'Brien
2025-04-03 - 3:45:57 PM EDT- IP address: 136.226.2.252

Document e-signed by Sean O'Brien (sean.m.obrien@duke.edu)
Signing reason: Approved
Signature Date: 2025-04-03 - 3:45:59 PM EDT - Time Source: server- IP address: 136.226.2.252

Document emailed to lbaden@bwh.harvard.edu for signature
2025-04-03 - 3:46:03 PM EDT

Email viewed by lbaden@bwh.harvard.edu

2025-04-03 - 3:57:48 PM EDT- IP address: 165.85.62.144

Adobe Acrobat Sign

Signer lbaden@bwh.harvard.edu entered name at signing as Lindsey Baden
2025-04-03 - 3:58:33 PM EDT- IP address: 165.85.62.144

Document e-signed by Lindsey Baden (lbaden@bwh.harvard.edu)
Signing reason: Approved
Signature Date: 2025-04-03 - 3:58:35 PM EDT - Time Source: server- IP address: 165.85.62.144

Document emailed to rwhitley@uabmc.edu for signature
2025-04-03 - 3:58:40 PM EDT

Email viewed by rwhitley@uabmc.edu
2025-04-03 - 4:07:23 PM EDT- IP address: 172.225.216.122

Signer rwhitley@uabmc.edu entered name at signing as Richard Whitley
2025-04-03 - 5:22:34 PM EDT- IP address: 138.26.219.57

Document e-signed by Richard Whitley (rwhitley@uabmc.edu)
Signing reason: Approved
Signature Date: 2025-04-03 - 5:22:36 PM EDT - Time Source: server- IP address: 138.26.219.57

Agreement completed.

2025-04-03 - 5:22:36 PM EDT

Adobe Acrobat Sign
